# Functional, Aromatic, and Fluorinated Monothiosemicarbazones: Investigations into Their Structures and Activity toward the Gallium-68 Incorporation by Microwave Irradiation

**DOI:** 10.1021/acsomega.1c07396

**Published:** 2022-04-11

**Authors:** Sophia Sarpaki, Fernando Cortezon-Tamarit, Rüdiger Maria Exner, Kexin Song, Sara Raquel Mota Merelo de Aguiar, Haobo Ge, Charareh Pourzand, Stephen James Paisey, Gabriele Kociok-Köhn, Jonathan Robin Dilworth, Laurence Carroll, Sofia Ioana Pascu

**Affiliations:** † Department of Chemistry, University of Bath, Claverton Down, Bath BA2 7AY, United Kingdom; ‡ Department of Pharmacy and Pharmacology, University of Bath, Claverton Down, Bath BA2 7AY, United Kingdom; § Centre of Therapeutic Innovations, University of Bath, Claverton Down, Bath BA2 7AY, United Kingdom; ∥ Wales Research and Diagnostic PET Imaging Centre, School of Medicine, University of Cardiff, Cardiff CF10 3AT, United Kingdom; ⊥ Department of Chemistry, Chemistry Research Laboratory, University of Oxford, Oxford OX2 3TA, United Kingdom; # Department of Medicine, Imperial College, Du Cane Road, London W12 0NN, United Kingdom; ∇ Russell H. Morgan Department of Radiology and Radiological Sciences, Johns Hopkins Medical Institutions, Baltimore, Maryland 21287, United States

## Abstract

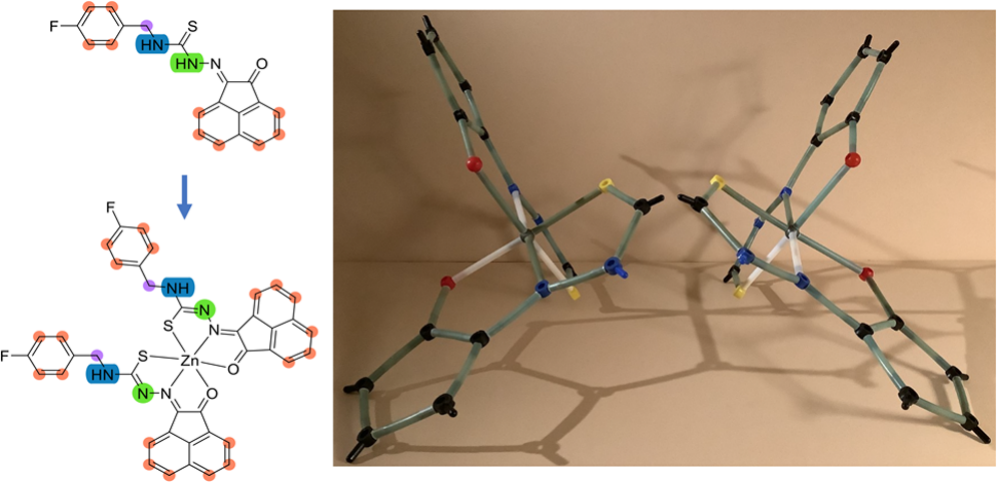

We report on the synthesis and spectroscopic characterization of a new series of coordinating monothiosemicarbazones incorporating aromatic backbones, featuring O/N/S donor centers monosubstituted with different aliphatic, aromatic, fluorinated, and amine-functionalized groups at their N centers. Their ability to bind metal ions such as Zn(II) and Ga(III) was explored, and the formation of two different coordination isomers of the Zn(II) complex was demonstrated by X-ray diffraction studies using synchrotron radiation. These studies showed the planar geometry for the coordinated mono(thiosemicarbazone) ligand and that the metal center can adopt either a heavily distorted tetrahedral Zn center (placed in an N/S/S/N environment, with CN = 4) or a pseudo-octahedral geometry, where the Zn(II) center is in the O/N/S/S/N/O environment, and CN = 6. Furthermore, 2-(4,5-dimethyl-2-thiazolyl)-3,5-diphenyl-2*H*-tetrazolium bromide (MTT) assays and cellular imaging in living cells were subsequently performed in two different cancer cell lines: PC-3 (a standard cell line derived from a bone metastasis of a stage IV prostate cancer) and EMT6 (a commercial murine mammary carcinoma cell line). The radiolabeling of new functional and aromatic monothiosemicarbazones with either gallium-68 (under pH control) or fluorine-18 is discussed. The potential of this class of compounds to act as synthetic scaffolds for molecular imaging agents of relevance to positron emission tomography was evaluated *in vitro*, and the cellular uptake of a simultaneously fluorinated and [^68^Ga]-labeled mono(thiosemicarbazone) was investigated and is reported here.

## Introduction

Positron emission tomography (PET) is a noninvasive imaging technique relying on positron-emitting radiotracers and offers various advantages over more traditional diagnostic techniques, including high specificity and sensitivity to probe physiological processes *in vivo*. Among other processes and metabolic adaptations, imaging of tumor hypoxia has been a dynamic field of research,^1−6^ due to the overall importance of prognosis. Investigations into the chemistry and chemical biology of thiosemicarbazones and of their metal complexes^7−9^ have been pursued from the perspective of their molecular imaging applications for targeting hypoxic tumors.^10−16^ Furthermore, this class of ligands and their metal complexes have received tremendous attention over the years due to their broad range of potential therapeutic targets.^17,18^

Earlier reports on simple acenaphthenequinone-anchored thiosemicarbazone ligands and their corresponding Fe(II), Ni(II), Cu(II), and Zn(II) metal complexes have shown that they can act as either bidentate or tridentate ligands and can inhibit cancer cell proliferation either as free ligands or, in the case of iron derivatives, as metal complexes.^19^ There is significant interest in the chemistry of bis(thiosemicarbazone) ligands featuring different radioactive metals as theranostic agents, and their potential as imaging agents for hypoxia has been investigated.^20^ The recent developments in the commercial availability of Gallium-68 from ^68^Ge/^68^Ga generators provide a reliable source of this positron-emitting radionuclide. The advantageous decay properties of Gallium-68 (*E*_max_ = 1.9 MeV, β^+^ 90%) coupled with a half-life of 68 min make this radionuclide attractive for imaging applications based on small molecules and/or biomolecules with relatively fast uptake and clearance rates.^21−25^ Additionally, the γ emitting gallium-67 isotope (*t*_1/2_ = 78 h) incorporated on the same synthetic platforms may well be of use in SPECT imaging mode, where longer imaging time frames are needed. The incorporation of an intrinsically fluorescent aromatic ligand backbone provided using synthetic precursors such as acenaphthenequinone was expected to confer structural rigidity and a higher kinetic stability compared to the corresponding complexes for gallium and indium anchored onto bis(thiosemicarbazones) with aliphatic backbones.^26^ We reported earlier the synthesis of Gallium-68 complexes featuring acenaphthenequinone bis(thiosemicarbazone) ligands that proved to be kinetically stable and showed a considerable hypoxia retention response compared to [^68^Ga]GaCl_3_ and [^64^Cu]Cu-ATSM.^26^

Here, we report on the synthesis and spectroscopic characterization of a new series of coordinating mono(thiosemicarbazone) acenaphthenequinone ligands incorporating aromatic backbones, featuring O/N/S donor centers monosubstituted with different aliphatic, aromatic, fluorinated, and amine-functionalized moieties at their nitrogen centers. Furthermore, 2-(4,5-dimethyl-2-thiazolyl)-3,5-diphenyl-2*H*-tetrazolium bromide (MTT) assays and cellular imaging were subsequently performed in two different cancer cell lines: PC-3 (derived from a bone metastasis of a stage IV prostate cancer) and EMT6 (a murine mammary carcinoma cell line). Their ability to bind metal ions such as Zn(II) and Ga(III) was explored, and the formation of two different coordination isomers of the Zn(II) complex was demonstrated by X-ray diffraction studies using synchrotron radiation. The potential of these compounds to act as future molecular imaging agents for positron emission tomography was evaluated by radiolabeling with gallium-68, and subsequently the cellular uptake of the resulting [^68^Ga]-labeled mono(thiosemicarbazone) was also investigated and is reported upon hereby.

## Results and Discussion

### Synthesis of Functional Monothiosemicarbazones

**Scheme 1:**
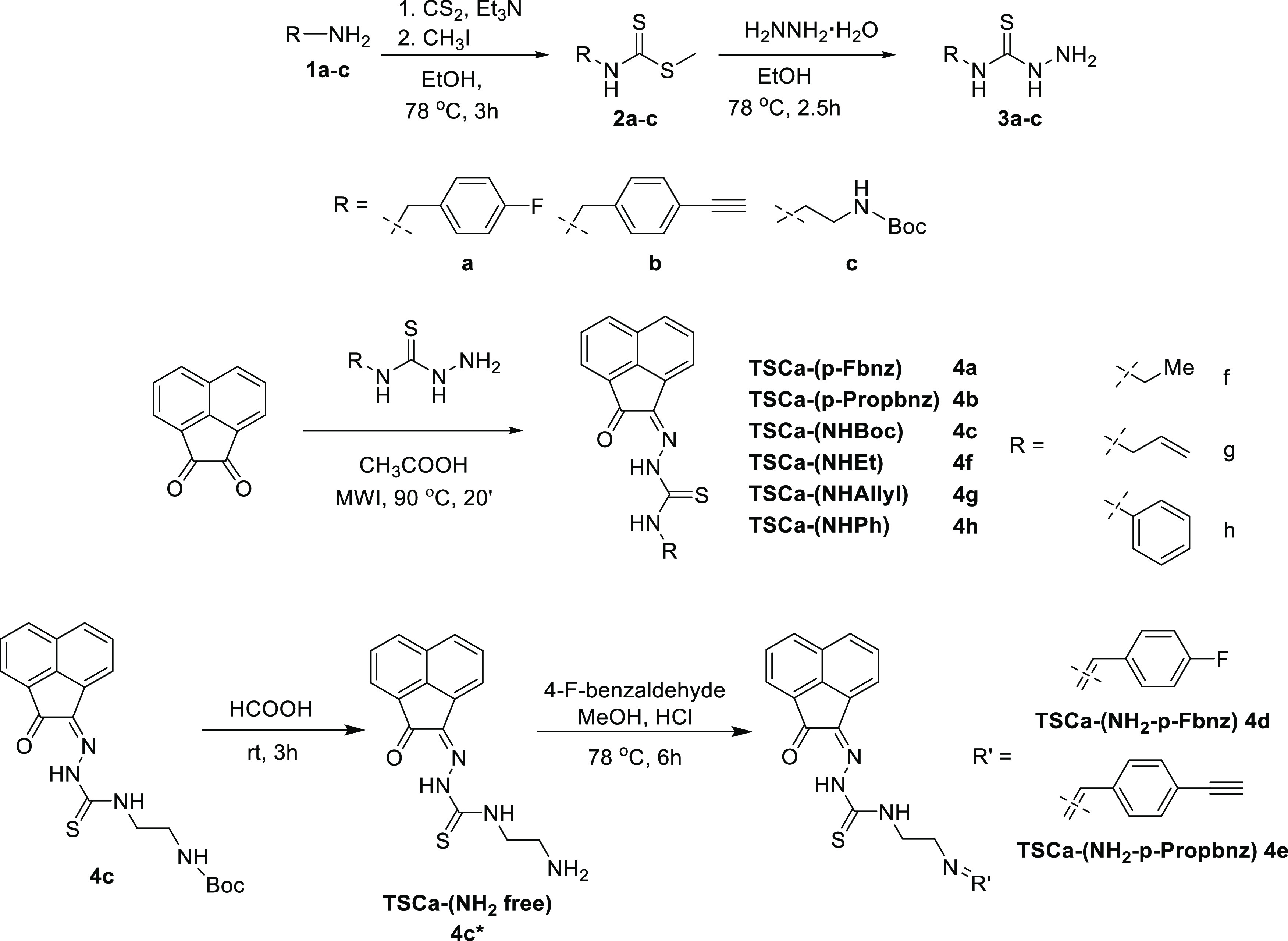
Synthetic Routes to Novel Thiosemicarbazide Precursors and Their Respective Acenaphthenequinone Mono(thiosemicarbazone) Ligands Using a Combination of Conventional Heating and Microwave Technology

The first step toward the synthesis of the new thiosemicarbazone ligands was the preparation of the corresponding functional thiosemicarbazide precursors, which was accomplished following adapted methods of previously described procedures^13^ involving the condensation reactions of commercially available amines with carbon disulfide in ethanol (Scheme 1). The addition of methyl iodide led to the respective thiocarbamate intermediates (**2a**–**c**). Subsequent hydrazinolysis of **2a**–**c** yielded the desired thiosemicarbazides (**3a**–**c**). The synthesis of the acenaphthenequinone-based mono(thiosemicarbazone) ligands was achieved following a modification of the previously described methodology,^20^ whereby the suspension of acenaphthenequinone and the thiosemicarbazide were reacted in acetic acid under microwave irradiation under a variety of conditions, with the most promising reaction being carried out for 20 min at 90 °C.

**Scheme 2:**
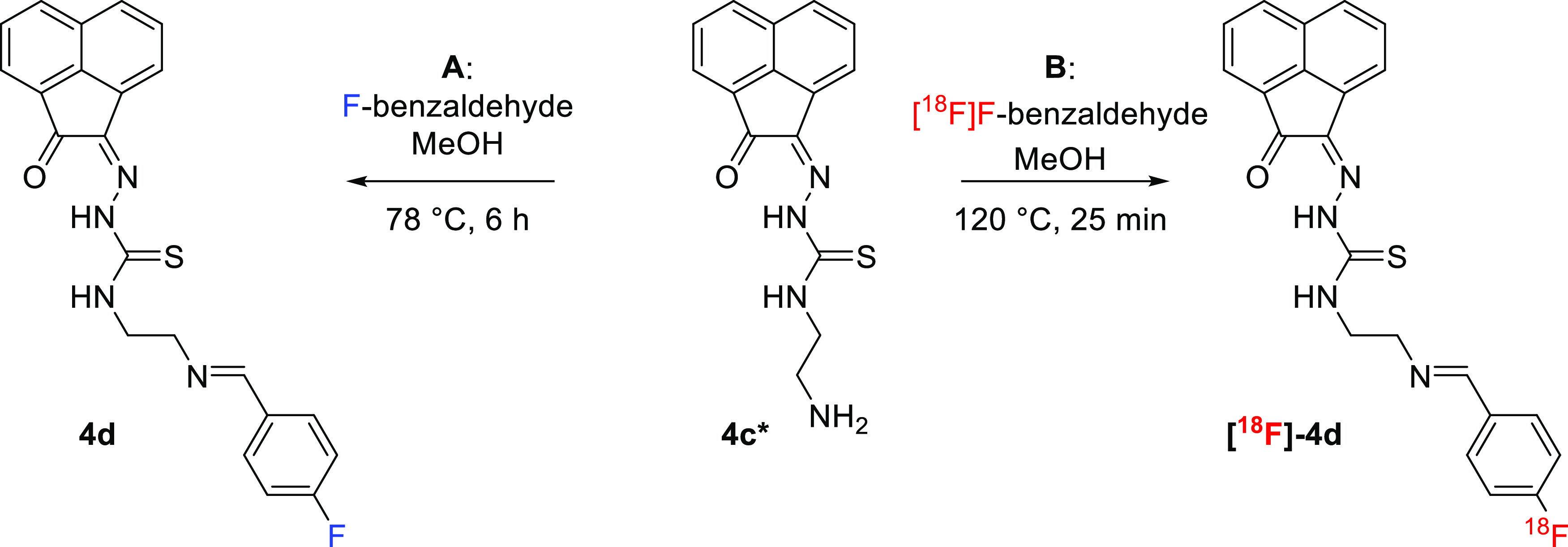
Coupling Reaction between Compound 4c* Featuring a Terminal Primary Amine and Either “Cold” Fluorobenzaldehyde (FBA, Route **A**, Thermodynamic Control) or [^18^F]F-Fluorobenzaldehyde (Route **B**, Kinetic Control, Proposed Structure for the Expected Product)

This method allowed for a rapid and efficient synthesis of the thiosemicarbazones denoted **4a** (shorthand: TSCA-(*p*-Fbnz)), **4b** (shorthand: TSCa-(*p*-Propbnz)), and **4c** (shorthand: TSCA-(NHBoc)). Synthetic approaches using microwave irradiation rather than conventional heating gave improved yields for the synthesis of compounds **4a**–**c**. Compound **4c** was further treated with an excess of formic acid to remove the protecting group, thus giving rise to the thiosemicarbazone ligand with a terminal 2-aminoethyl functional group (4c*) (Scheme 2). To obtain mono(thiosemicarbazone) compounds functionalized with a 4-(fluorobenzylidene)aminoethyl group, the intermediate amine terminal species 4c* was treated with 4-fluoro-benzaldehyde under “cold” chemistry conditions (thermodynamic control) that resemble a recent radiofluorination protocol (which has been carried out under kinetic control),^27−29^ giving rise to compound **4d**. An analogous method was applied for the formation of the *p*-propyl-benzoyl derivative **4e**, a new monothiosemicarbazone functionalized with a terminal alkyne group. This functional compound could open the possibility for further “click” derivatization reactions in future investigations. For the synthesis of simpler, analogous compounds (denoted in shorthand, for simplicity TSCA-Et (**4f**), TSCA-Allyl (**4g**), and TSCA-Ph (**4h**)), adapted methods with respect to our earlier published methods^20,26^ were applied and extensively optimized hereby. Reactions were performed under either microwave irradiation or conventional heating and no significant differences in the reaction yields, identities, or purities of the final compounds were observed. All compounds were fully characterized spectroscopically, as described below and in the Supporting Information (SI), and in most cases, the single-crystal X-ray diffraction unequivocally demonstrated the structure identity for these functional thiosemicarbazones, as well as the presence of several different polymorphs. For the case of **4h**, the structures of two different polymorphs were collected and the corresponding structural data are reported in the SI. The monothiosemicarbazones used hereby were investigated for their potential to undergo radiolabeling experiments with gallium-68. The compounds used for radiolabeling assays were generally those synthesized via the condensation reaction between one equivalent of acenaphthenequinone and one equivalent of thiosemicarbazide, with catalytic amounts of hydrochloric acid, in an ethanolic solution heated to 90 °C under microwave irradiation for ca. 10 min.

### Coupling Reaction of the NH_2_-Functionalized Monothiosemicarbazone 4c* with Fluorobenzaldehyde

**Figure 1:**
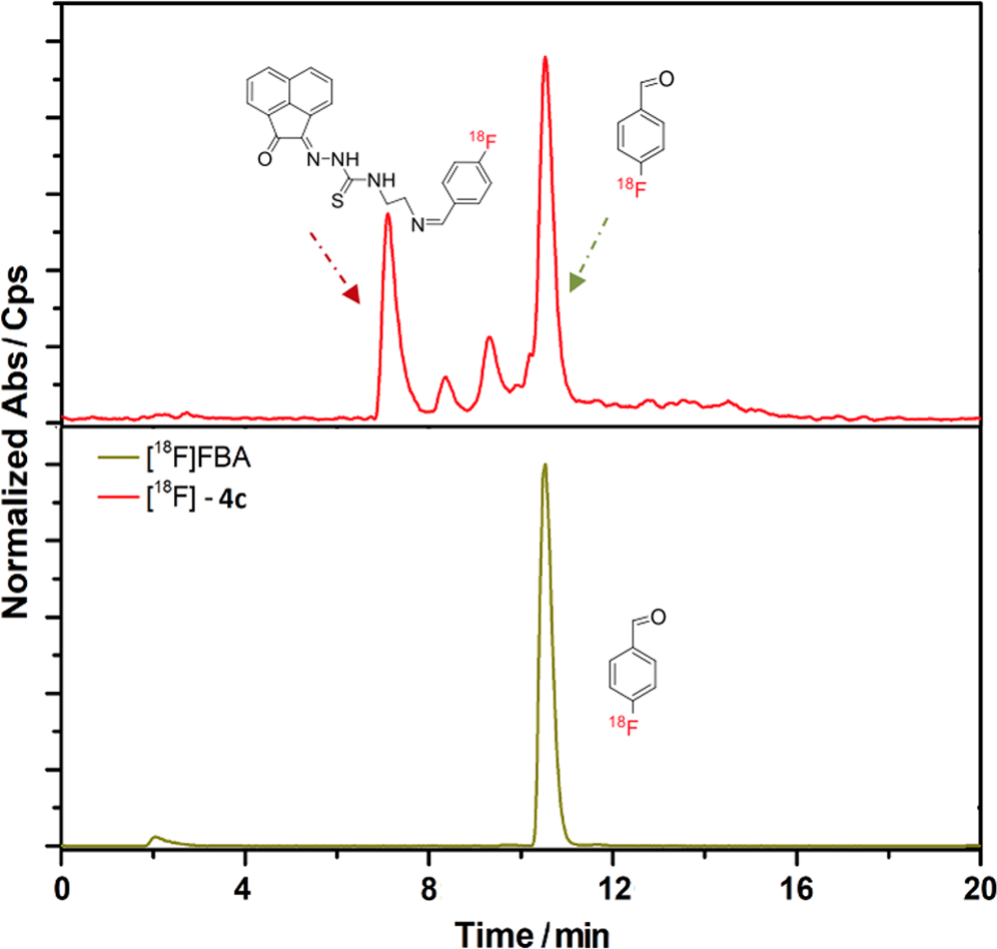
Radio-HPLC traces for radiolabeling of the NH_2_-terminated compound 4c* using [^18^F]FBA (rf. ca. 10.3 min). The occurrence of the desired [^18^F]-labeled compound proposed in Scheme 2 was indicated by the peak with retention time (rt) of ca. 7.1 min (red trace). Further minor bands assignable to hydrolysis of the desired imine under the radio-HPLC conditions and/or imine *E*/*Z* isomerization and side products formed in the presence of DMF and TFA were observed between 8 and 10 min.

The reaction between compound 4c* and a simple fluorinated aldehyde precursor, the (nonradioactive) fluorobenzaldehyde (FBA, route **A**, Scheme 2 and SI) was performed. This process was successful under thermodynamic control albeit under a protocol that necessitated over 6 h reaction time, as described in the Experimental Section. This straightforward method for fluorine incorporation was based on the condensation reaction between an aldehyde and a primary amine with the expected formation of the corresponding imine, and with the possible formation of *E*/*Z* isomers. To explore the potential for the radiolabeling of aromatic monothiosemicarbazones, the incorporation of fluorine-18 was investigated hereby for the first time. Preliminary experiments (under kinetic control) were carried out for the radiolabeling of the free-amine mono(substituted) compound 4c* with the no-carried added reagent [^18^F]fluorobenzaldehyde ([^18^F]FBA, route **B**). Challenges presented by this radiolabeling route remain, despite promising earlier reports^27−29^ and in our hands, these mainly resulted from the fact that the desired imines hydrolyzed to some extent under the aqueous reversed-phase high-performance liquid chromatography (RP-HPLC) conditions when traces of trifluoroacetic acid (TFA) are present. Additionally, to achieve the optimum yield for the reaction under thermodynamic control, route **A** necessitated rather harsh conditions under conventional heating (Scheme 2) or microwave irradiation (Experimental Section): these methods were not found to be compatible with the radiochemistry protocols, which were then adapted to make use of a combination of automated and manual labeling methods. Therefore, the synthesis of the [^18^F]fluorobenzaldehyde reagent was first conducted following an adapted protocol^29^ using an automated procedure on the FASTlab platform. In the first step, the [^18^F]fluoride (available from a cyclotron) was dried and then trapped on a Sep-Pak QMA-carbonate Light Cartridge before being eluted into the reactor using an eluent consisting of Kryptofix K_222_ and KHCO_3_ in acetonitrile: water (4:1). The content of the reactor was evaporated at 120 °C under reduced pressure and then under a very low flow of nitrogen. The “dried” and supported ^18^F-fluoride was then dissolved with anhydrous acetonitrile and transferred to a vial, and then the fluorination step was performed manually. Aliquots of dried fluoride were added by syringe to a v-bottom vial containing the precursor 4-trimethylammonium benzaldehyde triflate, a well-established precursor for the synthesis of no-carrier-added [^18^F]FBA by the nucleophilic substitution with [^18^F]fluoride.^27−29^ Then the vial was heated at 90 °C for 15 min resulting in the formation of consistently >98% radiochemical purity [^18^F]FBA according to radio-HPLC (10.3 min, radio-HPLC Method C), and used further for the labeling reactions of 4c*. To optimize the conditions for the ^18^F incorporation into compound 4c*, a variety of tests were conducted to assess the influence of temperature and solvents on labeling efficiency. For example, when the radiolabeling was attempted using MeOH at 90 °C the radiochemical incorporation (ROI) was very low (3%). When the reaction temperature was increased to 120 °C, a modest increase in the ROI followed, which prompted the evaluation of the use of a solvent more suitable for high-temperature conditions: as such, the use of DMF was employed and the reaction was allowed to proceed at 120 °C for 25 min. While this resulted in an ROI of ca. 30% (as shown in Figure 1) the potential of this solvent to interfere with the reaction and lead to side products should not be overlooked—indeed several peaks have been found in the radio-HPLC within 2–5 min retention time of the expected of the desired compound, whereas the analogous coupling reaction was successfully carried out under thermodynamic control, using ca. 6 h reaction time and a stoichiometric amount of the nonradioactive fluorobenzaldehyde (FBA, route **A**, Scheme 2 and SI).

### Spectroscopic and Structural Investigations

The new thiosemicarbazones and their corresponding pro-ligands were fully characterized by a combination of ^1^H,^13^C{^1^H}, and ^19^F{^1^H} NMR spectroscopy, IR spectroscopy, and mass spectrometry (see SI). Typically, the ^1^H NMR spectroscopy of the acenaphthenequinone monothiosemicarbazones investigated gave rise to two characteristic low-field resonances, between 12.7 and 12.1 ppm and between 9.3 and 10.0 ppm assignable to the hydrazinic proton and the amine proton, respectively. The ^19^F{^1^H} NMR spectroscopy of complexes of **4a** and **4d** gave rise to singlets at −115.74 and −109.86 ppm, respectively. The optical properties of all mono(thiosemicarbazones) were investigated using UV–vis absorption and fluorescence spectroscopy (SI), whereby generally they show weak fluorescence emission in dimethyl sulfoxide (DMSO) (*vide infra*). Interestingly, in the case of the imine conjugates **4d** and **4e**, the presence of geometric (*E*/*Z*) isomers may be possible due to different configurations of the C=N bond. However, we were unable to observe any direct evidence by NMR spectroscopy for the two possible isomers under the room temperature solution studies. Here, density functional theory (DFT) geometry optimizations (gas phase) indicated that the energy differences were small overall (SI): the calculated total bond energies of the optimized geometries for Isomers **4f-I** and **4f-II** were −205.8204 and −205.6919 eV, respectively, i.e., 2.96 kcal/mol difference between **4f-I** and **4f-II** and 7.36 kcal/mol between **4f-II** and **4f-III** were found by DFT calculations in the gas phase (SI).

**Figure 2:**
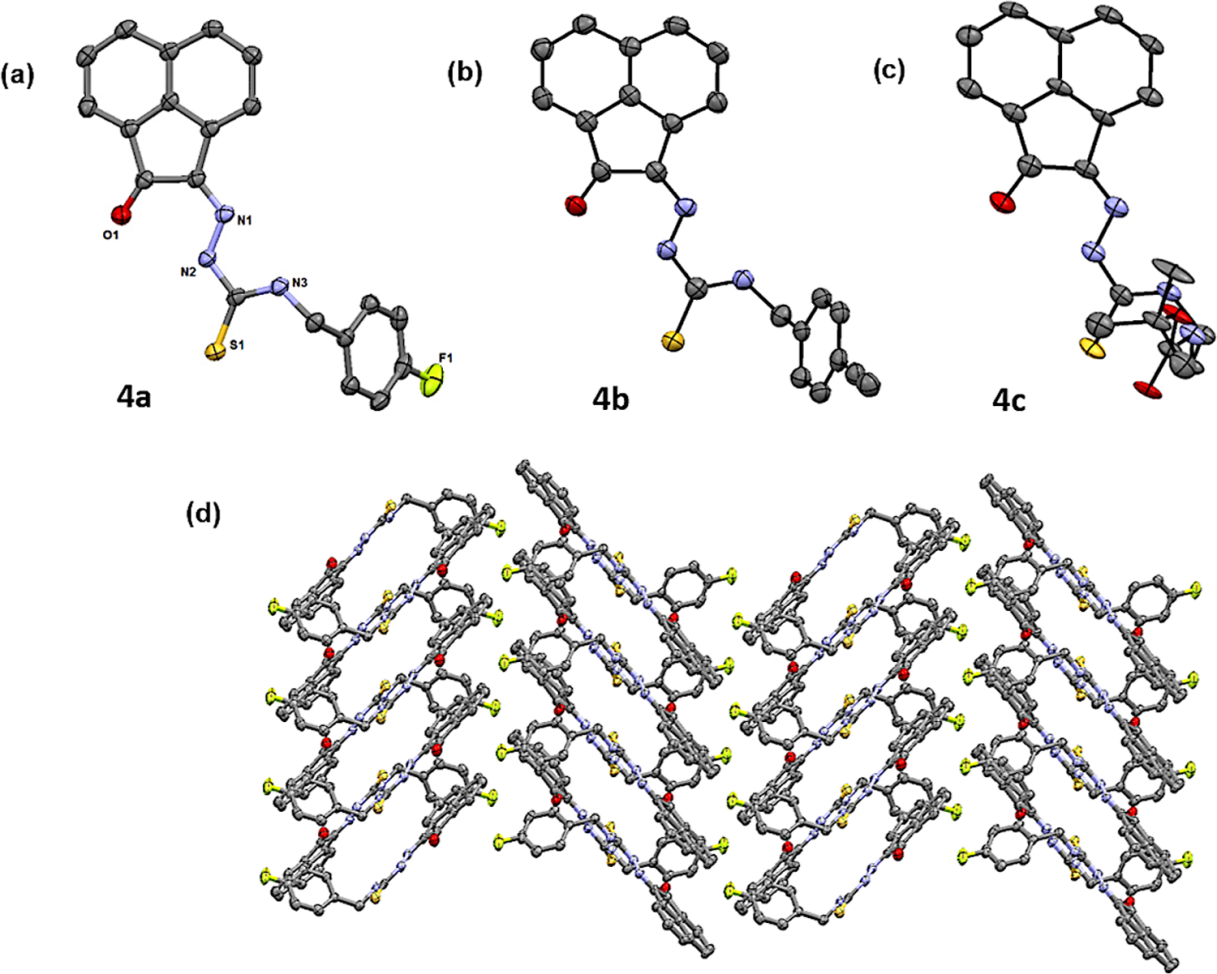
Single-crystal X-ray diffraction structure of 4-fluorobenzyl-3-thiosemicarbazone-acenaphthenequinone (**4a**, **4b**, and **4c**) showing 50% thermal ellipsoids (H atoms omitted for clarity). (a–c) Packing diagram showing a section of the unit cell, viewed along the axis *a* (d). Colors: N blue; O red; S yellow; C gray; F green.

**Figure 3:**
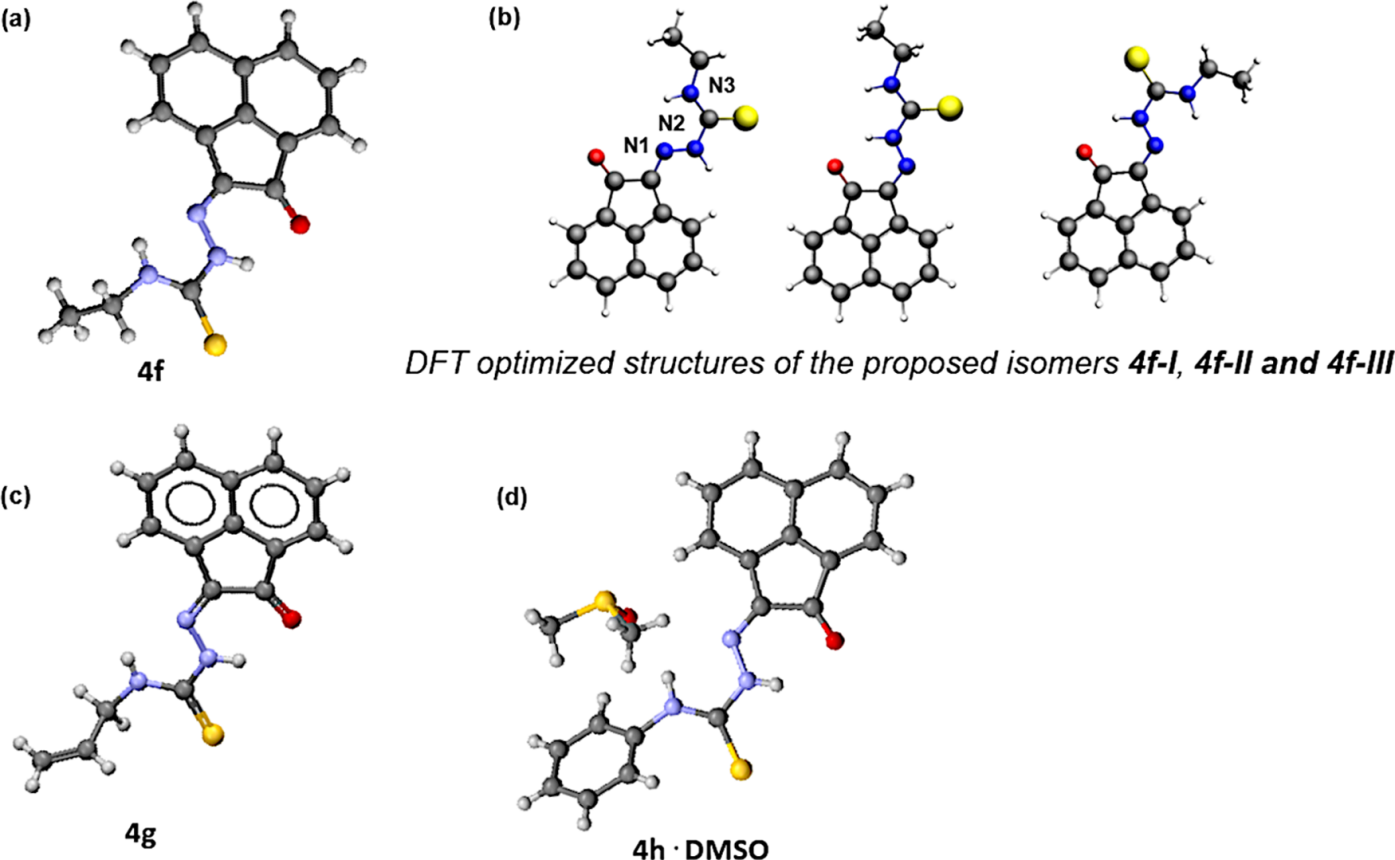
Overview of structural representations: (a) molecular structure of **4f** from X-ray diffraction analysis; (b) DFT-optimized geometries (BLYP/TZP, gas phase) for the model isomers denoted **4f-I**, **4f-II**, and **4f-III** (SI); (c) molecular structure of **4g** from X-ray diffraction analysis; and (d) molecular structure of **4h** determined by single-crystal X-ray diffraction as co-crystallized with one molecule of DMSO, H-bonded. Colors: N, blue; O, red; S, yellow; C, gray; F, green.

The molecular structures of **4a**, **4b**, and **4c**, also of the simpler monothiosemicarbazones **4f**–**h** were determined by single-crystal X-ray diffraction (Figures 2 and 3, Table 1), as were the structures of thiocarbamate and thiosemicarbazide precursors **2a** and **3a** (SI). The structural representation of **4a** is depicted in Figure 2 and all other crystal structures of these ligands and starting materials are given in the SI.

**Table 1:** Comparison of Some of the Relevant Molecular Parameters in Compounds **4f**–**h** (SI)

distance (Å)/angle (deg)	**4f**	**4g**	**4h**
**O1-C1**	1.215(3)	1.217(6)	1.24(1)
**C1-C2**	1.517(3)	1.514(6)	1.50(1)
**C2-N1**	1.294(3)	1.297(6)	1.28(1)
**C3-S**	1.675(2)	1.671(3)	1.6739(18)
**O1-N2**	2.789(3)	2.787(5)	2.729(9)
**O1-C1-C2**	125.9(2)	124.8(5)	123.8(8)
**C1-C2-N1**	128.3(2)	128.7(4)	130.3(7)
**O1-C1-C2-N1**	–1.3(4)	0.8(8)	0(1)

### Investigation into the Formation of Metal-Coordinated Species

The coordination chemistry of the mono(substituted) thiosemicarbazone ligands with Zn(II) and Ga(III) in undried organic solvents was one of the main aims of our investigations. The reactions of the ligands to Zn(II) and Ga(III) were studied to explore the formation of ML_2_-type complexes, where L^–^ represents the mono-anionic, deprotonated monothiosemicarbazone acting as a ligand. A variety of synthetic approaches for the formation of Zn(II) complexes of these monothiosemicarbazones, acting as ligands, were investigated and are described in the Experimental Section and in the SI: general methods involved either microwave irradiation methodologies or conventional heating, and the ligands **4f**, **4g**, and **4h** have been prepared using adapted methods from previous synthetic routes.^20,26^ Notwithstanding the efficacy differences in terms of sustainable chemistry, which favor microwave methods, no significant advantages were found with respect to yield or purity in the case of metal complex formation. Each of the ligands investigated was expected to act either as a tridentate O/N/S donor or as a bidentate N/S donor toward Lewis acids such as Zn(II) or Ga(III). In line with the literature observations,^19,30−32^ the as-synthesized compounds were anticipated to present distorted octahedral geometries around the metal center in the corresponding ML_2_-type complexes.

For these monothiosemicarbazones, a number of features are expected to pose challenges to their effective metalation reactions:

(a) The enhanced electron delocalization of the acenaphthenequinone unit, which would likely noticeably reduce the nucleophilicity of the carbonyl group;
(b) A notable feature common to the molecular structures of all of the monosubstituted ligands investigated is the short O(1)-(H-N(2)) intramolecular hydrogen bond which may reduce the nucleophilicity of the carbonyl and introduce an energetic hurdle to be surpassed for the complexation of the CO group to the metal center. Such monothiosemicarbazones, i.e., incorporating rigid, aromatic frameworks capable of extensive electronic delocalization involving exocyclic heteroatoms and featuring a number of intramolecular as well as intermolecular H bonds, have the potential to act as ligands toward Lewis acids such as Zn(II) or Ga(III) yet their coordination chemistry^31^ has not been fully investigated.

**Figure 4:**
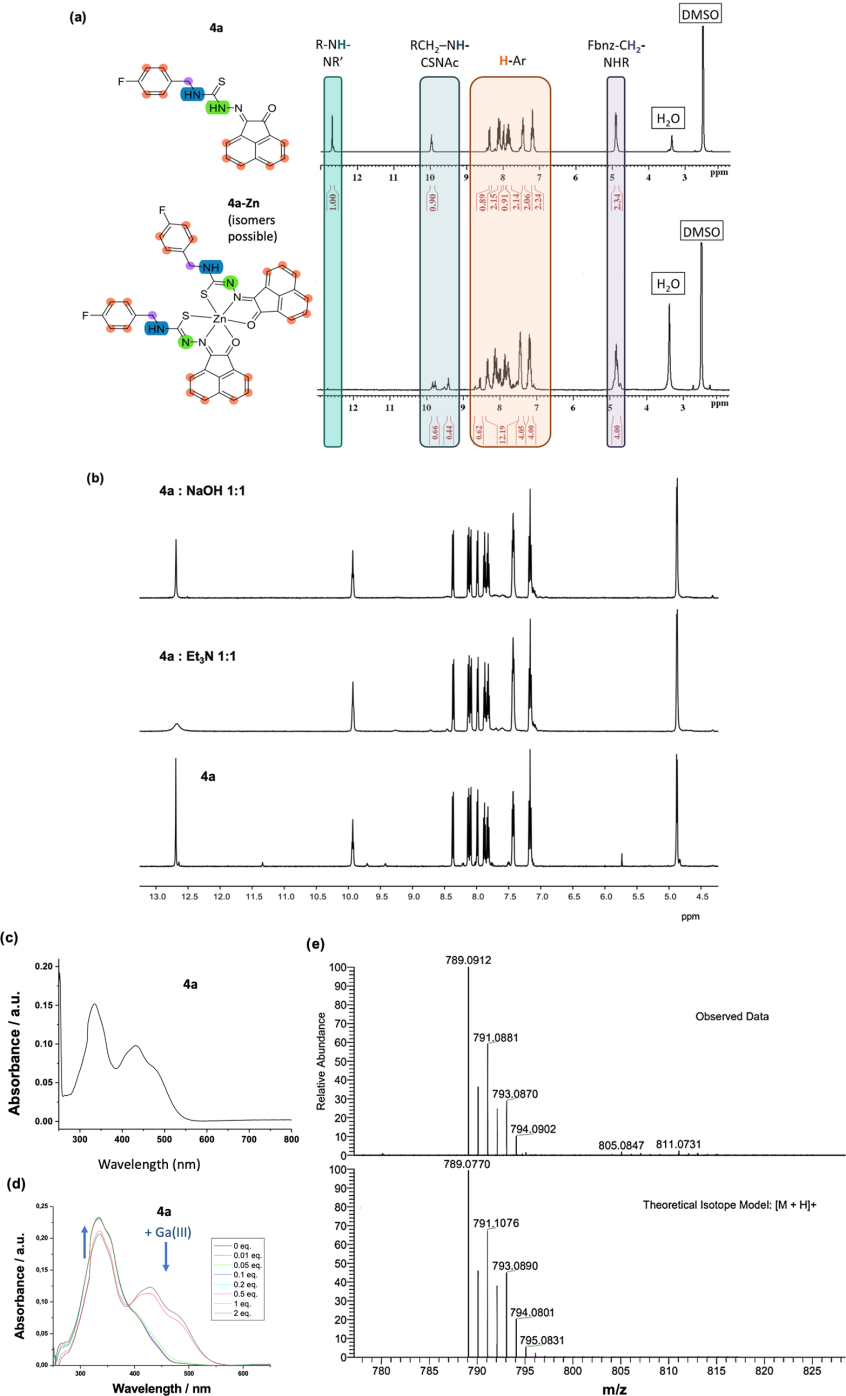
(a) ^1^H NMR spectroscopy (500 MHz, *d*_6_-DMSO, 293 K) of compounds **4a** and **4a-Zn**. (b) ^1^H NMR spectroscopy at the treatment of **4a** with different reagents in aqueous DMSO. (c) UV–vis absorption spectrum for compound **4a** at a concentration of 0.01 mM in DMSO. (d) UV–vis spectroscopy of **4a** with the addition of GaCl_3_ in DMSO (titration at a constant concentration of **4a**). Spectra were recorded after each addition of GaCl_3 solution aliquots_. (e) Mass spectrometry (ESI-MS, positive mode) of **4a-Zn**.

**Figure 5:**
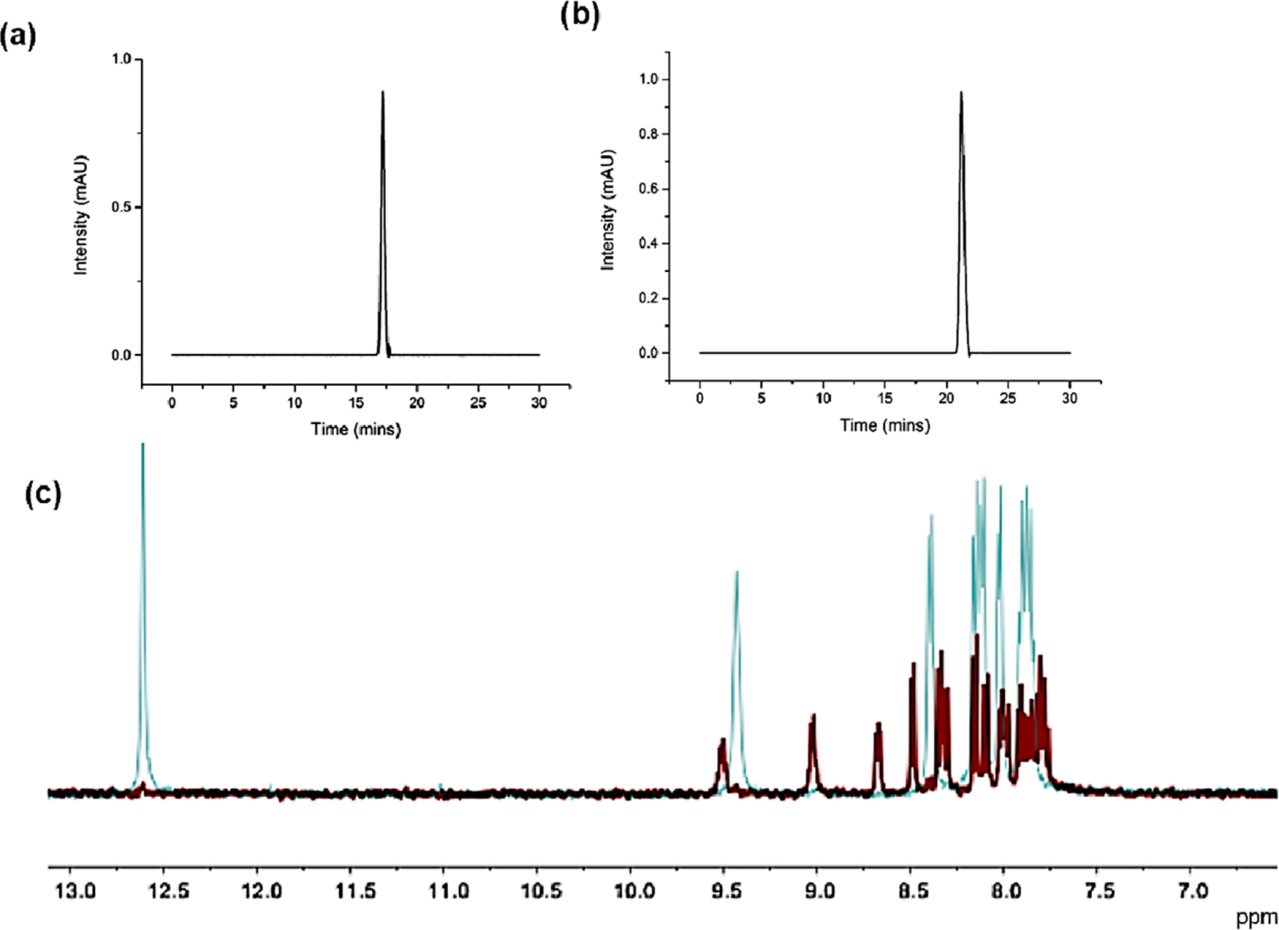
(a, b) HPLC traces of **4f** (top left) and **4f-Zn** (top right) recorded using UV detection (295 nm, Method B, Dionex C18 Acclaim column; 5 μm, 4.6 mm × 150 mm; 30 min reversed-phase, gradient method using MeCN/H_2_O containing 0.1% TFA as mobile phases). (c) Overlay of the ^1^H NMR spectrum (400 MHz, *d*_6_-DMSO, 293 K) of the monothiosemicarbazone ligand **4f** (blue line) overlaid onto the corresponding **4f-Zn** complex (red line), showing the doubling of some of the ligand-backbone assignable ^1^H resonances, which may be indicative of coordination isomers or be caused by the asymmetry of the ligand coordination with respect to the Zn(II) center. Full ^1^H NMR assignment is given in the Experimental Section.

**Scheme 3:**
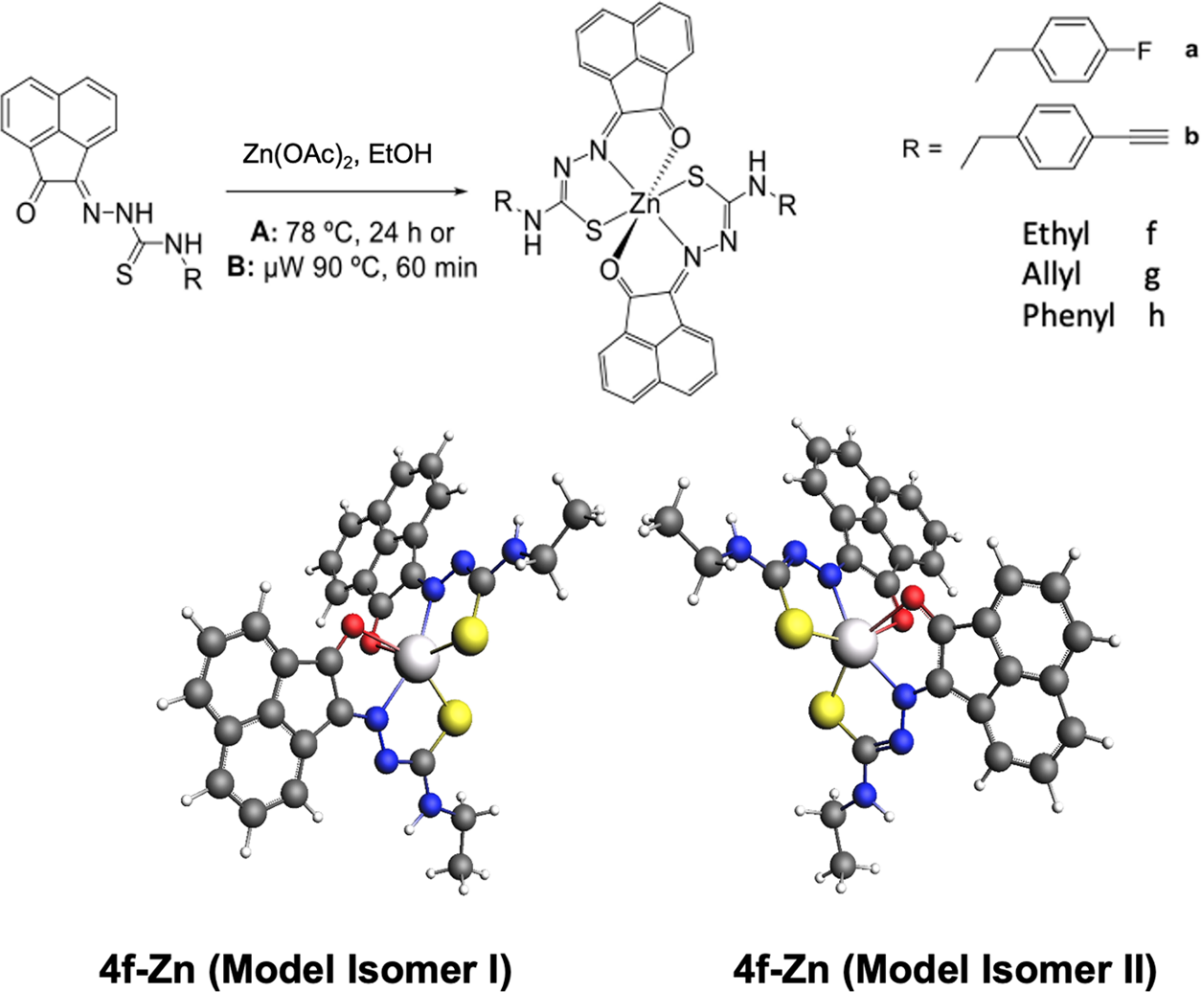
Synthetic Routes to the Zn(II) Complexes Reported Hereby (Top Row), Involving Either Microwave Irradiation (Route **A**) or Conventional Heating (Route **B**) ML_2_-type complexes (bottom row, e.g., illustrated for **4f-Zn**) were isolated regardless of the [Zn]:[ligand] ratio involved in the reactions carried out for the compounds studied. DFT calculations indicated the possibility of optical isomers for these pseudo-octahedral ML_2_ complexes. Colors: N, blue; O, red; S, yellow; C, gray; Zn, light gray.

In our hands, the Zn(II) metalation of the new functional monothiosemicarbazones of interest was carried out successfully in MeOH or EtOH as the solvent of choice under both conventional heating and microwave-assisted radiation (Scheme 3). These reactions appear to have led exclusively to the formation of the ML_2_-type species, as shown in Scheme 3. Key spectroscopic investigations are given in Figures 4 and 5 and the SI. The formation of ML(OAc)_2_ species was not observed, when reactions were carried out using either the 1:1 or 1:2 M/L ratios of Zn(II) precursor to monothiosemicarbazone ligands. The crude products were further purified *via* filtration and washed with methanol, yielding the desired products as orange solids in advanced purity as indicated by HPLC. These were fully characterized spectroscopically, and in the case of the ethyl-substituted monothiosemicarbazone, the Zn(II)-coordination product was characterized by X-ray diffraction, showing the possibility of the formation of isomers (*vide infra* and SI).

The ^1^H NMR spectroscopy confirmed the formation of the Zn(II)-complex from ligand **4a**. Figure 4 shows the ^1^H NMR spectroscopy of compound **4a** as well as the ^1^H NMR of this compound after the treatment with different bases in aqueous *d*_6_-DMSO: these experiments indicated only very subtle changes to the nature of the ligand in the presence of amines such as Et_3_N, i.e., the broadening of the NN*H* assignable proton resonance at 12.6 ppm was observed in this case. In contrast, virtually no deprotonation seemed to occur in the presence of NaOH.

A comparison with the ^1^H NMR of **4a** after treatment with Zn(OAc)_2_ and isolation of its Zn(II) derivative **4a-Zn** is also included whereby ^1^H NMR spectroscopy indicated the increased complexity in the Zn(II) complex spectrum and doubling of most ligand-backbone assignable ^1^H resonances in the presence of this Lewis acid, likely due to isomerism. Notably, the disappearance of the resonance at 12.6 ppm (Figure 4a) provided strong evidence of full deprotonation of the hydrazone position (R-N*H*-NR′).

Additionally, the secondary amine resonance related to the fluorobenzylamine (RCH_2_-N*H*-CSNA) appeared to be duplicated, as seen in Figure 4b. This may be the result of the previously mentioned isomerism: the diagnostic resonance for the presence of the neutral monothiosemicarbazone is the NH peak at 9.0–10.0 ppm. However, complexity in the ^1^H NMR spectra of these complexes can also be due to the asymmetry of the ligand coordination with respect to the Zn(II) center.

Similar features can be observed also in the NMR spectrum of **4a** in the region between 7.0 and 8.6 ppm where the resonances from aromatic protons (*H*-Ar) are observed, and at ca. 4.9 ppm where the resonance from the benzyl group appears (see the Experimental Section for details). These results strongly suggest the coordination of mono(substituted) ligands in two different environments and the coordination of **4a** to Zn(II) was also confirmed by mass spectrometry. Similar NMR experiments were conducted for compounds **4a**, **4b**, **4f**, **4g**, and **4h**, and the full assignment of the corresponding Zn(II) complexes is given in the Experimental Section and the SI. Interestingly, no significant changes in the ^1^H NMR spectroscopy of the Zn(II) complexes were observed over 72 h in wet DMSO, indicating the kinetic stability of these species in organic solvents. Kinetic stability challenges in DMSO conducted in the presence of glutathione suggested a significantly lower kinetic stability with full decomposition to free ligands over 24 h, by UV–vis spectroscopy (SI).

**Figure 6:**
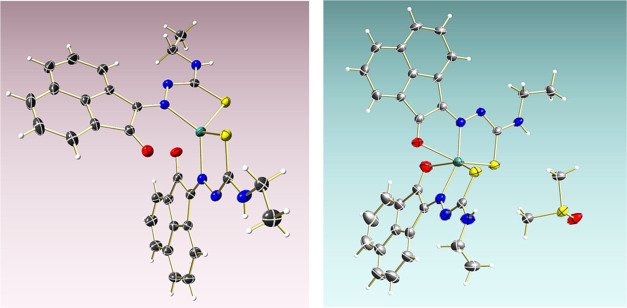
X-ray diffraction studies of compound **4f-Zn** showing the formation of two coordination isomers, with Zn(II) in heavily distorted Td vs distorted Oh environments. Crystals suitable for X-ray diffraction were obtained when complex **4f-Zn** was synthesized using conventional heating protocols. Colors: N, blue; O, red; S, yellow; C, gray; H, white; Zn, green.

Determination of the molecular structure of **4f-Zn** by single-crystal X-ray diffraction indicated that for this complex, two coordination isomers are possible, and these single crystals grew, and were mechanically separated, from the NMR tube. Structural investigations performed on the **4f-Zn** complex indicated a pseudo-octahedral geometry with the C.N = 6 around the Zn(II) center in the [O/N/S]_2_ environment, although the coordination isomer for **4f-Zn**, with the metal center in CN 4 and a distorted tetrahedral geometry (in the N/S/N/S environment) is also possible. However, HPLC studies (Figure 5) did not indicate any differences in solution. Sequential recrystallization did not lead to the isolation of pure isomers; however, crystallography studies indicated that the two structural isomers present different coordination geometries around the zinc center, as well as different orientations of one of the N-Et chains (Figure 6 and Table 2). DFT calculations in the gas phase indicated that for the pseudo-Oh geometry (SI), there is also the possibility of optical isomer formation (SI).

**Table 2:** Selected Bond Lengths and Angles for the Structures of the **4f-Zn** Determined by X-ray Diffraction^a^

**4f-Zn** (distorted Td geometry)			
bond lengths [Å]		bond angles [deg]	
Zn-N(3)	2.0445(17)	N(3)-Zn-N(6)	128.25(7)
Zn-N(6)	2.0686(17)	N(3)-Zn-S(1)	85.34(5)
Zn-S(1)	2.3323(6)	N(6)-Zn-S(1)	125.43(5)
Zn-S(2)	2.3340(6)	N(3)-Zn-S(2)	122.81(5)
Zn-O(2)	2.7189(16)	N(6)-Zn-S(2)	84.91(5)
Zn-O(1)	2.8296(15)	S(1)-Zn-S(2)	113.87(2)
S(1)-C(3)	1.738(2)	N(3)-Zn-O(2)	74.15(6)
S(2)-C(18)	1.718(2)	N(6)-Zn-O(2)	70.83(6)
N(1)-C(3)	1.334(3)	S(1)-Zn-O(2)	82.71(4)
N(1)-C(2)	1.466(3)	S(2)-Zn-O(2)	155.70(4)
N(1)-H(1)	0.822(17)	N(3)-Zn-O(1)	70.07(5)
N(2)-C(3)	1.346(3)	N(6)-Zn-O(1)	74.55(6)
N(2)-N(3)	1.350(2)	S(1)-Zn-O(1)	155.31(3)
N(3)-C(4)	1.300(2)	S(2)-Zn-O(1)	79.42(3)
N(4)-C(18)	1.332(3)	O(2)-Zn-O(1)	92.37(5)
N(4)-C(17)	1.454(3)	C(3)-S(1)-Zn	92.59(7)
		C(18)-S(2)-Zn	93.19(7)

**4f-Zn** (distorted Oh geometry)			
bond lengths [Å]		bond angles [deg]	
Zn-N(3)	2.069(4)	N(3)-Zn-N(6)	156.37(13)
Zn-N(6)	2.071(3)	N(3)-Zn-S(2)	110.53(10)
Zn-S(2)	2.3814(12)	N(6)-Zn-S(2)	81.41(10)
Zn-S(1)	2.3816(12)	N(3)-Zn-S(1)	82.31(10)
Zn-O(2)	2.434(3)	N(6)-Zn-S(1)	114.33(9)
Zn-O(1)	2.527(3)	S(2)-Zn-S(1)	107.57(4)
S(1)-C(1)	1.722(4)	N(3)-Zn-O(2)	87.30(12)
S(2)-C(16)	1.743(4)	N(6)-Zn-O(2)	75.37(11)
O(1)-C(14)	1.236(5)	S(2)-Zn-O(2)	153.34(8)
O(2)-C(29)	1.221(5)	S(1)-Zn-O(2)	93.98(7)
N(1)-C(1)	1.348(6)	N(3)-Zn-O(1)	74.17(11)
N(1)-C(2)	1.445(6)	N(6)-Zn-O(1)	86.06(11)
N(1)-H(1)	0.846(19)	S(2)-Zn-O(1)	89.15(8)
N(2)-N(3)	1.336(5)	S(1)-Zn-O(1)	154.89(8)
N(2)-C(1)	1.352(5)	O(2)-Zn-O(1)	76.48(10)
N(3)-C(4)	1.318(5)	C(1)-S(1)-Zn	93.68(14)
N(4)-C(16)	1.325(5)	C(16)-S(2)-Zn	94.51(14)
N(4)-C(17)	1.463(5)		

a The main structural differences are found in the Zn–O distances.

**Figure 7:**
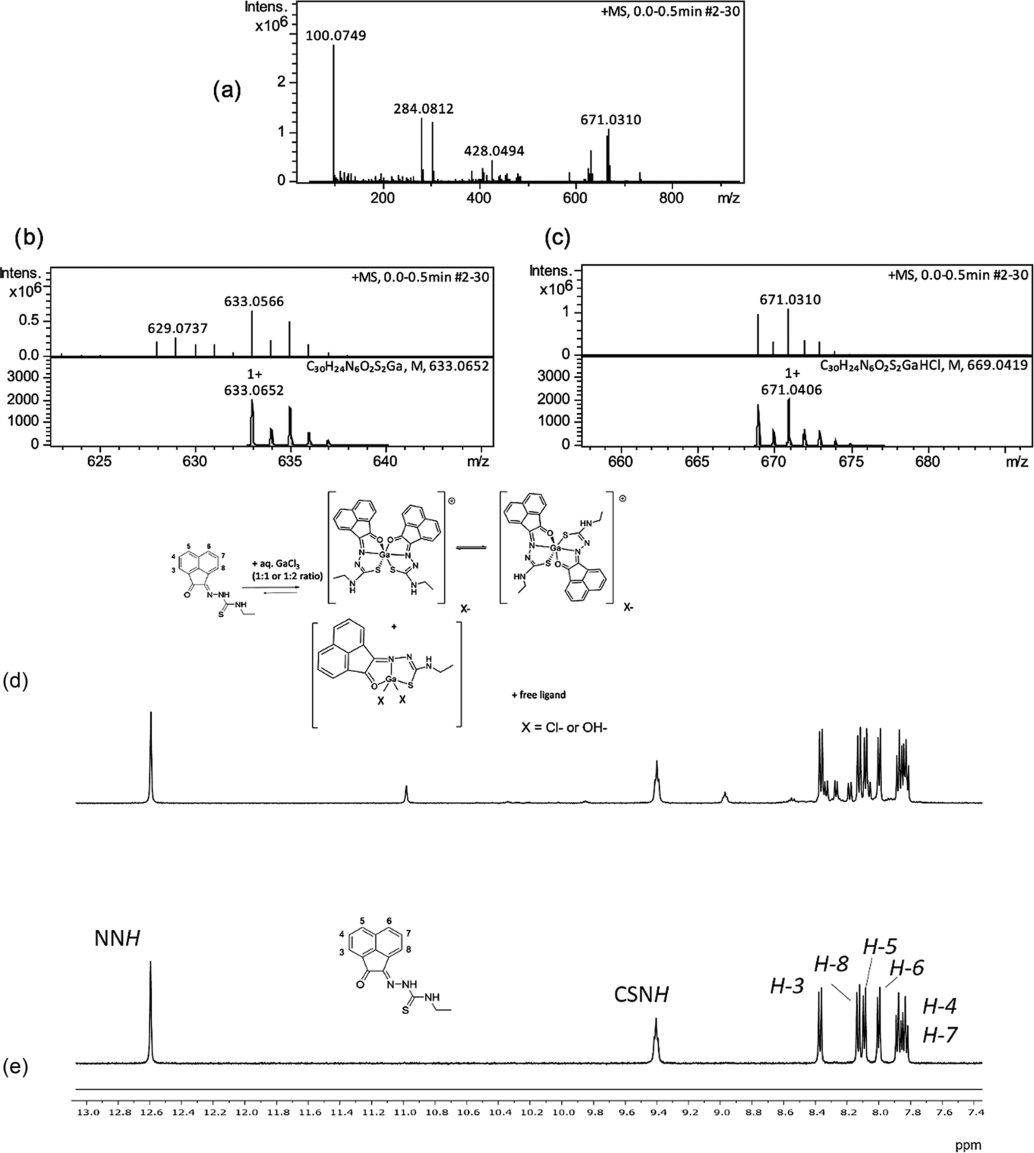
Formation of Ga(III) complexes. (a) Mass spectrometry of Ga-4f (ESI-MS) positive mode showing full spectrum, (b) relevant *m*/*z* peaks indicative of [M]+ and (c) [M + H + Cl]+, where [M] corresponds to 1:2 [Ga]:[**4f**] complex ion formation; and (d) proposed products for the complexation reactions carried out in THF containing trace amounts of water (1:1 or 1:2 reaction, X = OH^–^ or Cl^–^). (e) ^1^H NMR spectroscopy (DMSO-*d*_6_, 400 MHz) of the NMR-scale reaction between compound **4f** and GaCl_3_ (1:1 ratio).

Given the intrinsic planarity of the ligand frameworks upon complexation with a metal center, as shown for **4f-Zn**, two possible coordination isomers (pseudo-Oh, with likely optical isomerism, and pseudo-Td) were predicted for **4a-Zn** and one might expect to observe different ^19^F resonances in solution for each of these. However, in our hands, observation of different ^19^F signals has not been forthcoming in the experiments carried out at room temperature. Nevertheless, after complexation experiments carried out toward the formation of corresponding cold zinc(II) and gallium(III) complexes (see SI) multiple pairs of ligand-assignable peaks were observed by ^1^H NMR and interpreted as a possible indication of the formation of inseparable isomers. These, in turn are likely to impact the ^68^Ga radiolabeling reactions, which are processes carried out under kinetic control (*vide infra*). Gas-phase DFT investigations on the **4f-Zn** complex indicated that the ligand framework is generally planar and substituents resemble a mer-like orientation with the S-Zn-S angle of ca. 100° (compared to 107.57(4)°) in the pseudo-Oh **4f-Zn** X-ray structure, and significantly smaller than the 113.87(2)° found for the pseudo-Td geometry of the second **4f-Zn** polymorph. These observations are in line with earlier work by Cowley et al.^32^ who reported related homoleptic Re complexes supported on N/S ligands. For the Re analogues reported, no evidence for any isomers could be found. Similar synthetic approaches to those described for the Zn(II) complexes in this series did not lead to Ga(III)-coordinated monothiosemicarbazones: for all species in the series, the thermodynamic product of the Ga(III) complexation was not isolated, and during purification attempts their decomposition to free ligand emerged, regardless of the different conditions applied. Attempts at transmetallation from Zn(II) to Ga(III), performed for the case of compound **4a-Zn**, **4f-Zn**, or **4g-Zn** either using microwave irradiation or conventional heating did not lead to pure compounds either, although extensive and conclusive mass spectrometry did provide evidence for the formation of the desired gallium-substituted monothiosemicarbazonato species denoted **4f-Ga** or **4g-Ga**, and these proposed structures are shown in Figure 7 (see the SI). Mass spectrometry (MALDI-TOF as well as ESI^+^) show extensive fragmentation and HPLC characterization and separation proved challenging. Gas-phase DFT calculations indicated that the monocationic complexes whereby Ga(III) is hexa-coordinated in the [ONS/SNO] environment are thermodynamically stable and they show optical isomerism (SI).

The ^1^H NMR titrations of compound **4f** with GaCl_3_ carried out in DMSO in 10 mM conc. and followed by UV–vis titrations (in DMSO, in diluted solutions) indicated that the gallium–ligand association does occur in dilute solutions. The UV–vis absorption spectra for titration of **4a** (0.01 mM in DMSO, host) with aliquots of GaCl_3_ (titration at constant volume of “host”) indicated significant changes with respect to the free ligand UV–vis spectroscopy, indicating metal–ligand association, e.g., an isosbestic point that indicates a metal insertion process was observed, and these spectra are given in the SI. We conclude that in our hands, gallium incorporation reactions carried out under thermodynamic control led to mixtures of gallium-containing products but the separation and full characterization of the thermodynamic product has been elusive thus far, unlike the case of their corresponding Zn(II) complexes. A previous report in monothiosemicarbazones indicated that the formation of GaL_2_X_2_ (where X = Cl or OH) as well as [GaL_2_]^+^ complex cations is likely.^30,31^

**Figure 8:**
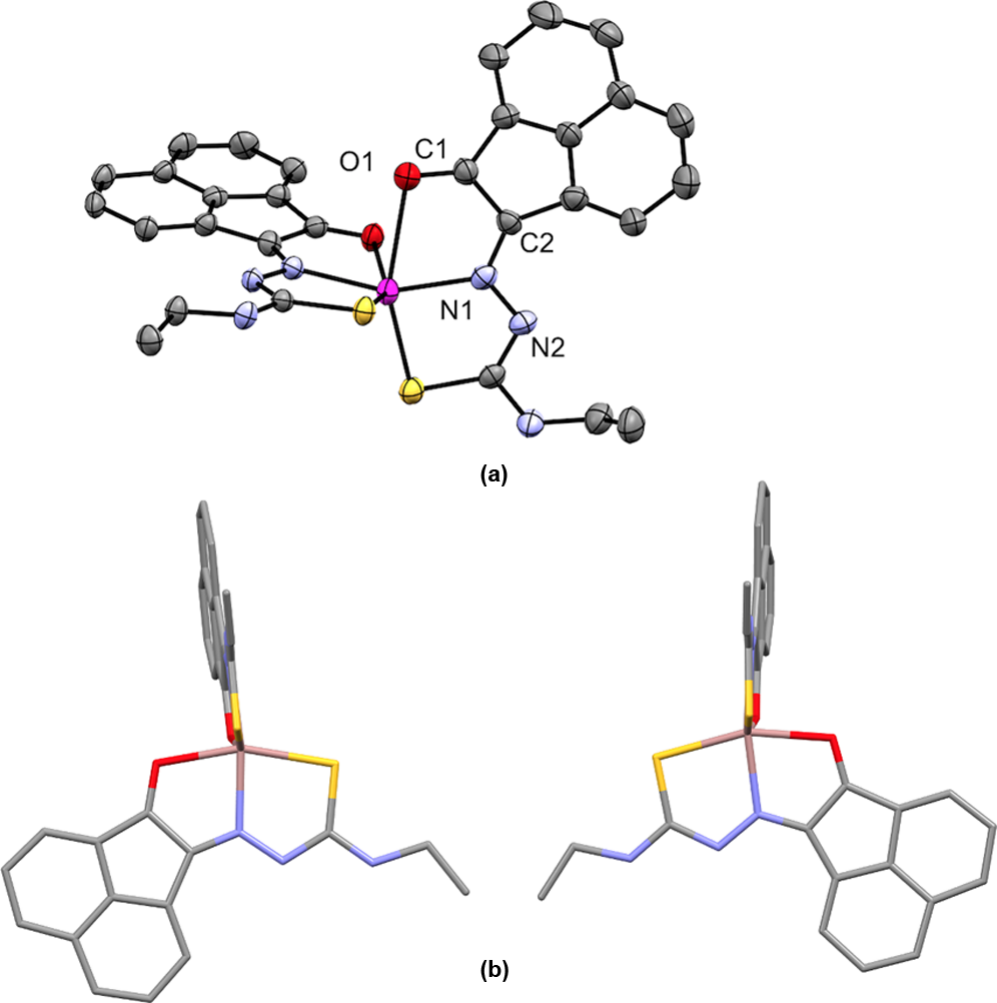
Comparison of the coordination geometries: (a) Pseudo-Oh geometry of the corresponding **4f-Zn** isomer determined experimentally from the single-crystal X-ray diffraction analysis is given for a comparison with the (b) gas-phase DFT calculations BLYP/TZ2P level: geometry optimizations in gas phase for the proposed pseudo-octahedral species **4f-Ga** of type ML_2_^+^, where M = Ga(III) and L^–^ = mono-deprotonated ligand **4f**. The molecular parameters, bond energies, and corresponding xyz files for the optimized geometries of the isomers proposed are given in the SI. H atoms were omitted for clarity. Colors: C, gray; O, red; S, yellow; Zn, magenta; Ga, brown.

To the best of our knowledge, there are only a small number of structurally related thiosemicarbazone derivatives, for example, those incorporating the 2-acetylpyridine 4N-alkyl scaffolds which were characterized structurally and currently reported. Their Ga(III) and In(III) complexes which have been prepared and characterized in solution and solid state, demonstrate a preference for [ML_2_]^+^ for the gallium complexes while the less common [MLX_2_] is also reported for a simple gallium-4*N*-alkyl thiosemicarbazone derivative.^30^ Therefore, the general trend from the very limited available structures reported in the CSD seems to indicate that gallium complexes of thiosemicarbazones are of the type [ML_2_]^+^ and [MLX_2_] (where X = OH– or OMe–, or Cl−) which falls in line with the MS fragments seen in this work (SI).^30^ Gas-phase DFT calculations show that thermodynamically the formation of the [GaL_2_]^+^ complex (as two optical isomers with respect to the metal center in the CN = 6) may well be possible; however, a detailed investigation into the nature of bonding within these complexes was not carried out hereby. The difference between the calculated bond energies of the optimized geometries ML_2_^+^ for the modeled isomers denoted **4f-Ga I** and **4f-Ga II** were found to be of only −0.01 kcal/mol difference in the gas phase, and for the Zn(II) species of type ML_2_, **4f-Zn** I and **4f-Zn II** of only 0.01 kcal/mol, therefore interconversion, likely through the formation of distorted Td intermediates in solution, with breaking of the metal-oxygen interactions is highly likely (Figure 8). A previous report on monothiosemicarbazone with less rigid backbones complexations indicated that the formation of [GaLX_2_] (where X = OH– or OMe–, or Cl−) as well as [GaL_2_]^+^ complex cations is feasible.^30^ Therefore, the formation of gallium complexes under kinetic control using aqueous ^68^Ga(III) ions (available from a generator or cyclotron produced, *vide infra*) is highly likely, and they are not expected to be readily separated by HPLC.

### Radiolabeling Experiments with Gallium-68

The radiosynthesis of several gallium-68 labeled acenaphthenequinone mono(thiosemicarbazonato) complexes has been performed using our previously optimized microwave-driven or conventional heating protocols.^20,33^ It appears that the reactions carried out under kinetic control for the radiolabeling of this class of monothiosemicarbazones with gallium-68 ions have been more promising than the similar reactions under thermodynamic control described above. To establish a radiolabeling protocol for this family of compounds, a number of challenges in the formulation of the gallium precursor were undertaken using adapted methods from our previous investigations into ^68^Ga aqueous chemistry.^33^

Overall, the radiolabeling of the mono(substituted) thiosemicarbazone acenaphthoquinone complexes was achieved as follows. The [^68^Ga]GaCl_3(aq)_ was eluted from the generator and trapped in a CXS4 cartridge. It was then washed with 20 mL of H_2_O before being eluted with a THF/0.02M HCl (98%) solution. Additional washing of the cartridge with water was found to be crucial to keep the ROI high. The eluted [^68^Ga]GaCl_3_ (aq) was subsequently dried for 10–15 min under a nitrogen stream at 95 °C. Pure methanol was then used to resuspend [^68^Ga]GaCl_3_, and the mono(substituted) ligand was added (2 mg/mL in DMSO). This was heated under microwave-assisted (μW) radiation at 95 °C for 30 min and injected into a radio-HPLC. The highest radiochemical incorporations were achieved with ligand **4a** (FbnzTSCA). Despite the various conditions applied, some of which include adjusting the pH of the reaction or changing the ratio between the monothiosemicarbazone substrate and the [^68^Ga]GaCl_3_, it was not possible to surpass the ROI achieved for **4a** when the rest of the ligands in the series were used.

Key points influencing the outcome of the radiolabeling seem to be the elution of the [^68^Ga]GaCl_3(aq)_, the drying procedure of [^68^Ga]Ga(III), the pH of the reaction, and the ratio between the precursor (mono(substituted) ligand) and the [^68^Ga]GaCl_3_. Three different eluents were used to optimize the reaction. Acetone/0.02M HCl (98%) and THF/0.02M HCl (98%) were suggested from previous similar experiments,^34^ and celite/0.02M HCl (98%) was used to ameliorate the pH adjustment of the reaction. For the experiments carried out with the first two eluents, [^68^Ga]GaCl_3_ (aq) was first dried under a stream of nitrogen at 110 °C for 15 min. Despite [^68^Ga]GaCl_3_(aq) being carefully dried in both cases, the eluent of THF/0.02M HCl (98%) appeared to result in a much higher radiochemical incorporation (ROI) compared to elution with acetone. For the experiments carried out with celite/0.02M HCl (98%), both reactions in dry [^68^Ga]GaCl_3_ or in solution were attempted; however, none of them were successful.

The use of a buffer was also investigated; however, this did not improve the ROI of the reaction. Tables 3 and 4 summarize some of the conditions applied to optimize the reactions performed using [^68^Ga]GaCl_3_ eluted with THF/0.02M HCl as an ca. 98% solution. The reaction was optimized using the ethyl-substituted monothiosemicarbazone **4f** (2 mg/mL in DMSO) and then translated to the other related compounds in the series.

**Table 3:** Optimized [^68^Ga]Ga(III) Radiochemical Incorporation for a Diverse Library of Monothiosemicarbazones under Microwave Radiation Conditions

	monothiosemicarbazone precursors	total radiochemical incorporation (combined ROI)
**4a**	TSCa-(*p*-Fbnz)	98%
**4b**	TSCa-(*p*-Propbnz)	55%
**4f**	TSCa-Et	75%
**4g**	TSCa-Allyl	67%
**4h**	TSCa-Ph	70%

**Table 4:** Summary of the Experiments Carried Out on the **4f** Mono(Substituted) Ligand (EtTSCA) for Optimization of the Radiolabeling Reaction of the Ligand with [^68^Ga]GaCl_3_ through Different Conditions^a^

solvent	concentration of **4f** (mM)	NaOAc buffer	final pH	*T* (°C) (μW)	time (min)	combined ROI
EtOH	0.42	pH 4.5	4.5	95 °C	30–60	
EtOH	0.42	pH 5.0	5.2–6.4	95 °C	30–60	
EtOH	0.34	no buffer use	4.5–6.5	95 °C	30–60	
MeOH	0.34	pH 4.5	4.8	95 °C	30–60	
MeOH	0.34	pH 5.0	4.8–6.4	95 °C	30–60	
MeOH	0.34	no buffer use	5.8	95 °C	30–60	75%
MeOH	0.34	no buffer use	∼2	95 °C	30–60	
MeOH	0.34	no buffer use	3.8–6.4	95 °C	30	55–65%

a When a range of pH values are stated, this indicates that more than one experiment was carried out at this pH range. All experiments were repeated at least three times.

**Figure 9:**
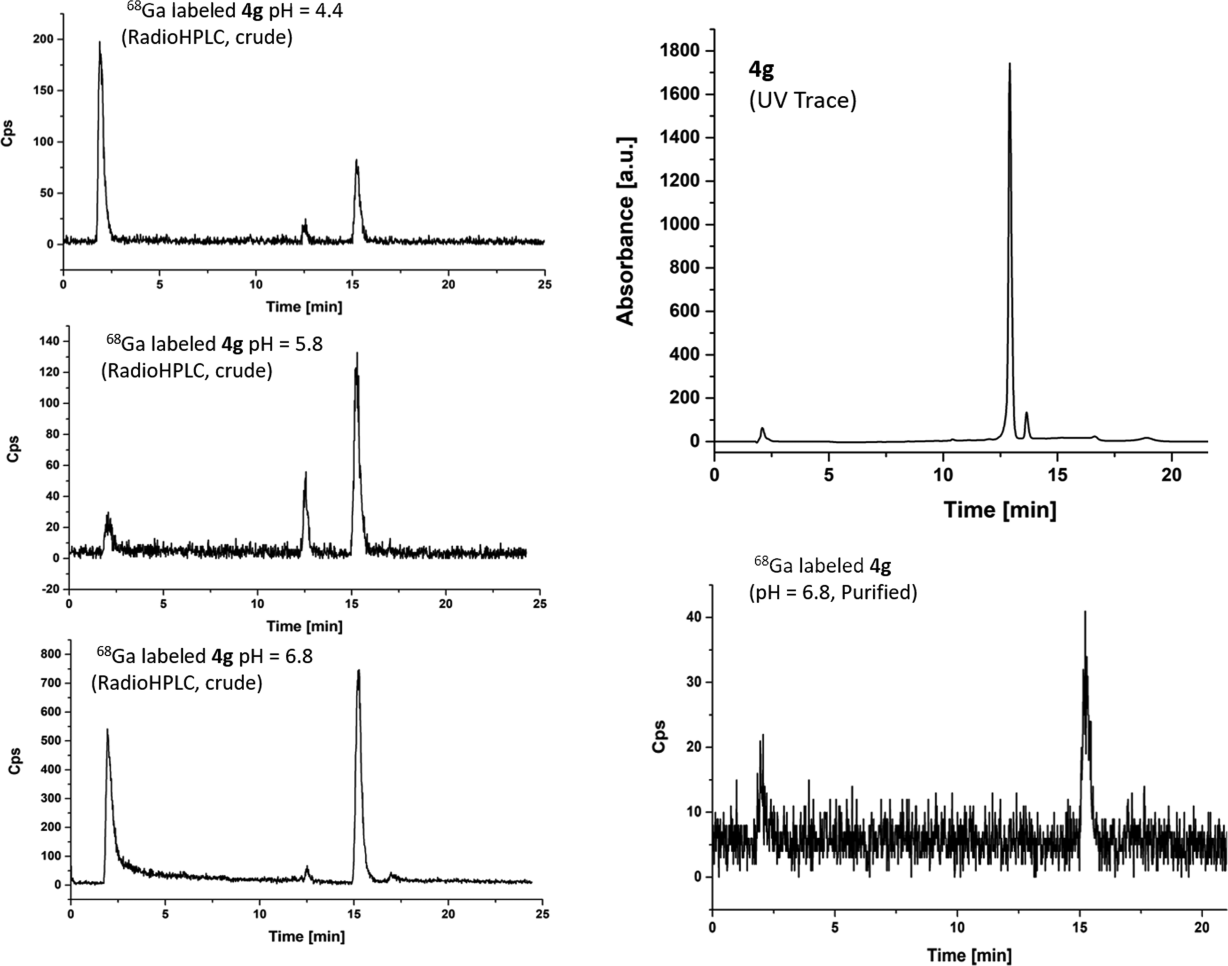
Analytical data for the optimized radiochemistry assays carried out under pH control for **4g** (HPLC Method C, Experimental Section). Radio-HPLC traces as well as the HPLC of **4g** (cold ligand precursor, UV detection) and of the purified ^68^Ga-labeled **4g** (major species, HPLC Method C, Experimental Section) are shown.

**Figure 10:**
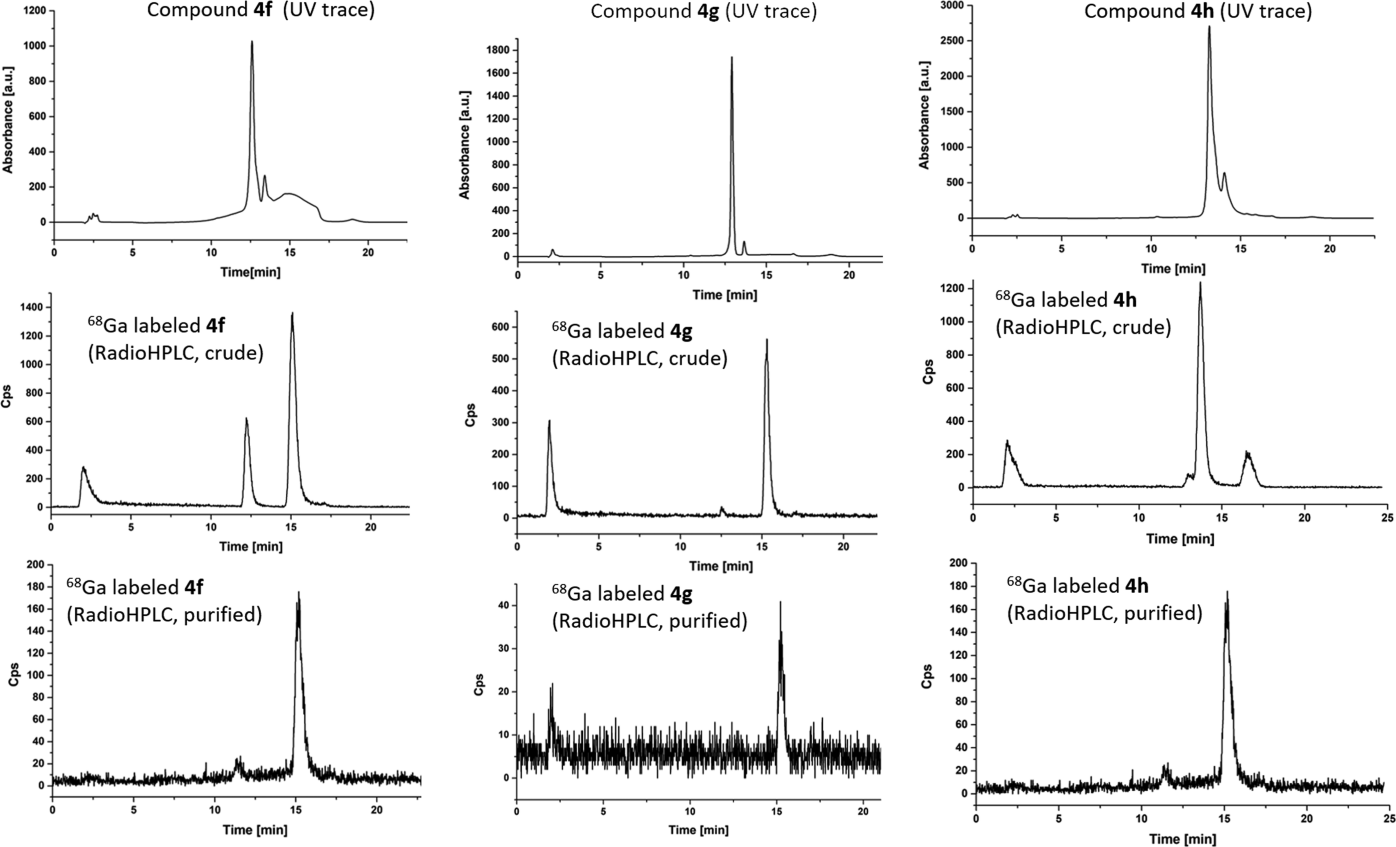
Overview of the radio-HPLC at the labeling of **4f**, **4g**, and **4h** (pH maintained between 4 and 7). Additional radio-HPLC traces are given in the SI.

Validating the nature of the species emerging from Ga-68 radiolabeling of monothiosemicarbazones of this family of compounds proved challenging, especially since the analogous cold gallium(III) coordination chemistry, whereby reactions between these ligands and GaCl_3_ or GaNO_3_ were carried out under thermodynamic control (at the ratio of ligands to metal of either 1:1 or 2:1) did not appear to proceed efficiently and resulted in inseparable mixtures. Thus, the cold standards for analytical chemistry comparisons were unavailable for Ga(III), while the Zn(II) complexation proved to invariably lead to the preferential formation of ML_2_-type derivatives. Radiolabeling with [^68^Ga] proceeds under kinetic control and it has been observed that this is a key point influencing the outcome of the reactions between all ligands mentioned above and in Scheme 3 and the formulation of gallium-68. The main steps mentioned above seemed to be the initial elution and drying procedure for the [^68^Ga]GaCl_3_, the pH of the reaction, and the ratio between the ligand and the radiolabel metal precursor. Under optimized conditions, the aqueous ^68^Ga(III) precursor likely consists of a mixture of [^68^Ga]GaCl_3_ along with different gallium-68 complexes featuring chloride, hydroxy, and aqua species as ligands, after the initial elution from the generator with 0.6 M HCl and prior to being reformulated into a solution containing anhydrous THF and 0.02M HCl. This resulting aqueous [^68^Ga]GaCl_3_ solution was subsequently dried in a borosilicate ampoule or test tube for 10–15 min under a nitrogen stream at 95 °C to remove the traces of solvents and acid that could interfere with the thiosemicarbazone complex formation. Pure methanol was then used to resuspend the gallium-68 residue which was anchored onto the walls of the borosilicate glass test tube thus allowing subsequent derivatization. Following this, the corresponding thiosemicarbazone ligand was added to the reaction in dry DMSO (2 mg/ mL). The borosilicate glass tube was then heated under microwave irradiation (MWI) at 95 °C for 30 min providing an overall successful incorporation of the unlabeled [^68^Ga]GaCl_3_. The radiochemical incorporation (ROI) estimated from the radio-HPLC for each thiosemicarbazone ligand (Figures 9 and 10, Tables 3 and 4, and SI) demonstrated overall radiochemical incorporations exceeding 65% for the 4-fluorobenzyl-3-thiosemicarbazone-acenaphthenequinone ligand; the fluorinated ligand **4a** synthesized in this work achieved an overall combined 98% radiochemical incorporation.

**Figure 11:**
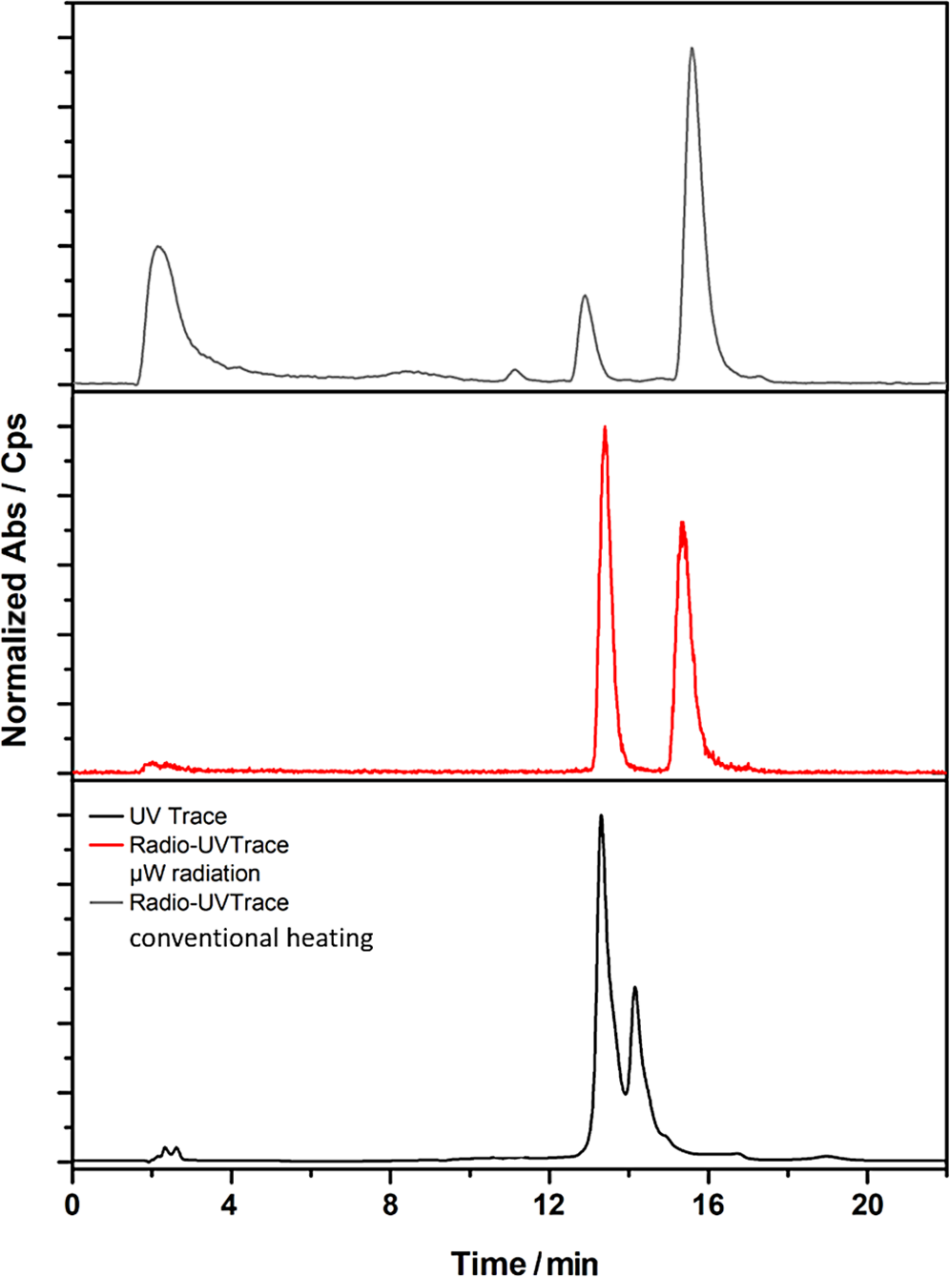
Representative UV-HPLC trace for the cold compounds synthesized (black) and radio-HPLC trace for radiolabeling of the free ligand **4a** with gallium-68 through microwave-assisted (μW) radiation (red line) or conventional heating (black line), giving rise to the (proposed) complexes [^68^Ga]GaL and [^68^Ga]GaL_2_, assignable to the two different retention times. Reinjection of an isolated fraction of the later eluting species of the gallium-68 labeled mono(thiosemicarbazone) derivatives showed a similar distribution of products, including some “free” [^68^Ga]GaCl_3_. These results may indicate that the resulting gallium complexes have limited kinetic stability under HPLC conditions, which could explain the difficulties encountered in separating the corresponding cold Ga complexes.

Allowing the reaction to proceed for a longer time provides the desired compounds when using conventional heating; however, radiochemical incorporation dropped significantly, compared to heating by microwave irradiation. This was evident from observation of the large band in radio-HPLC for the early eluting species, corresponding to free [^68^Ga]GaCl_3(aq)_ (Figure 11).

Isolation of the slowly eluting, major fraction by semipreparative HPLC allowed for the reinjection of the substance, which led to further loss of gallium-68. Interestingly, the ratio of these peaks is heavily influenced by the radiolabeling pH. For compound **4g**, the allyl-substituted monothiosemicarbazone ligand, a pH-dependent radiolabeling experiment showed that the optimum pH of 6.4 leads to optimized conditions and more easily separable components. Overall, the resulting radio-HPLC traces showed a similar distribution of species, suggesting that the Ga-68 complexes form with a high degree of reproducibility regardless of the nature of the substituent involved; however, they may be of limited kinetic stability over time.

These radio-reactions were also successfully carried out with conventional heating by allowing for a longer reaction time. For example, Figure 11 shows the radio-HPLC (red and gray lines) for the radiolabeling of mono(substituted) ligand (**4a**) with [^68^Ga]GaCl_3_. The HPLC indicated that the conversion of the mono(substituted) ligand to the respective gallium-68 complex had occurred under both conventional heating, and microwave irradiation. However, the presence of [^68^Ga]GaCl_3_ signal was also found indicating that radiolabeling did not proceed to completion: the reaction in which microwave radiation (Figure 11, red trace) was used had the greatest ROI as opposed to the reaction which underwent conventional heating (Figure 11, gray trace).

In our hands, these radiolabeling protocols were previously tested side-by-side with gallium-68 extracted by either a generator or directly from a cyclotron and both of these approaches produced similar results with highly comparable ROIs. Here the labeling of **4a** was also successfully carried out also with gallium-68 produced by a cyclotron via the ^68^Zn(p,n)^68^Ga reaction in aqueous solution. The procedure involved the additional step whereby the eluted [^68^Ga]GaCl_3_ (aq) was then trapped in a CXS4 cartridge and the labeling procedure followed was as stated earlier resulted in a radiolabeled complex with a similar ROI to the one stated above. A purification procedure was developed to extract only the radiolabeled compound through the use of a C18 cartridge. The cartridge was first primed with EtOH and then washed with H_2_O before loading the as-obtained compound. This was further washed with H_2_O, to remove the remaining, free, [^68^Ga]Ga(III) before further elution by treatment with a small amount of EtOH. Independent of the methodology employed, the radio-HPLC traces systematically indicated the presence of two distinctly different retention times from species with similar properties by radio-HPLC which are separable as they are eluting within ca. 2–3 min of each other. Separation and reinjection of an isolated fraction of the later eluting species of the gallium-68 labeled mono(thiosemicarbazone) derivative showed a similar distribution of products, including some free [^68^Ga]GaCl_3_. These results may indicate that the resulting gallium complexes have limited kinetic stability by HPLC, which could explain the difficulties encountered in separating the cold Ga analogues.

**Scheme 4:**
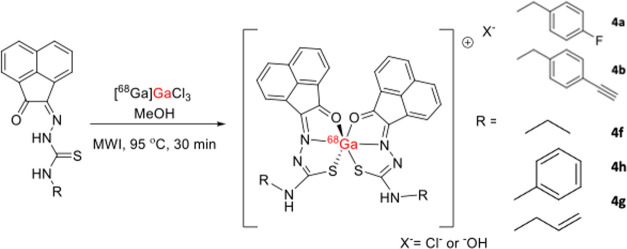
Incorporation of Gallium-68 within Acenaphthenequinone Thiosemicarbazones in Aqueous Media through the Formation of [GaL_2_]^+^ Complex Cations Formation of coordination isomers is possible, and the possibility of the formation of a 1:1 [^68^Ga(III)]:[monothiosemicarbazone] species of type GaLX_2_ (X = OH– or Cl−) cannot be discounted.

We suggest that in each case the two signals correspond to two distinct complexes of gallium(III) in different coordination environments and which we hypothesize to feature the earlier eluting, presumably an **MLX**_**2**_-type compound together with the **[ML**_**2**_**]**^**+**^**X**^–^-type complex (where X^–^ = OH^–^ or Cl^–^), as depicted in Scheme 4 and the SI.

### Cellular Uptake of a Gallium-68 Radiolabeled Thiosemicarbazone

**Figure 12:**
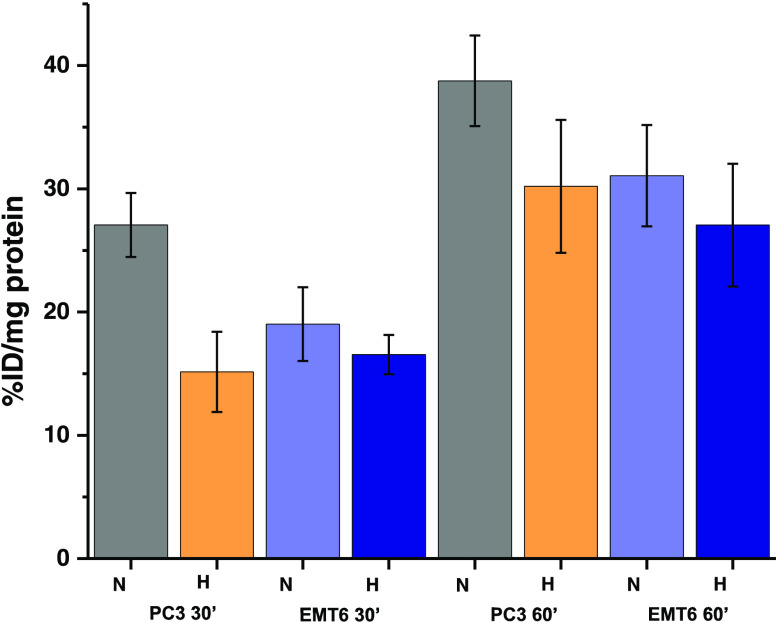
Investigations into the cellular uptake of [^68^Ga]Ga radiolabeled compound **4a**, denoted **4a-**^**68**^**Ga**. The retention in PC-3 and EMT6 cells under normoxic (N) and hypoxic (H) conditions measured 30 and 60 min after addition of tracer and expressed in % of internalized dose/mg of protein. Error bar stands for standard error (±SE), calculated from six repeated measurements.

For the gallium-68 labeled 4-(fluorobenzyl)-3-thiosemicarbazone-acenaphthenequinone **4a-**^**68**^**Ga** (determined as a mixture of complexes type [^68^Ga]Ga(L)_2_X and [^68^Ga]GaLX_2_ likely in dynamic exchange and with the presence of free gallium species and uncoordinated ligand, even after the HPLC separation) the level of uptake in living cancer cells cultured under normoxia or chemically induced hypoxia was estimated using a well-established γ-counting method. Uptake was investigated after incubation with [^68^Ga]-**4a-Ga**. The Ga-68 radiolabeled thiosemicarbazone **4a-**^**68**^**Ga** was tested for its uptake in different cell lines under both normoxic and chemically induced hypoxic conditions (under the CoCl_2_·6H_2_O assay conditions, see Experimental Section). To gain a handle on any level of selectivity for this gallium-68 radiolabeled species under hypoxic conditions, the compound was further tested at the time points of 30–60 min. The results suggest that notable differences in cellular uptake and localization can be seen between cell lines (Figure 12). Experiments appear to show that although as a general trend uptake seems to decrease somewhat under hypoxia in each example, this variation did not show any statistical significance.

These observations are noteworthy since PC-3 (human prostatic small cell neuroendocrine carcinoma) cell line has not been reported to have a highly oxidative phenotype.^35^ Therefore, treatment with the compound might trigger a metabolic transition phase (30 min) followed by an adjustment state (60 min). On the other hand, the EMT-6 (mouse breast mammary adenocarcinoma) cell line has been shown to have an increased sensitivity upon exposure to hypoxic conditions,^36^ which can be reflected in the reduced cellular uptake of the gallium-68 labeled monothiosemicarbazone. Generally, in all cases, association levels suggest that uptake is likely to be considerable, however in related cold cellular uptake (see SI) only a weak fluorescence emission was observed, indicating that the metal complexes very likely dissociate within cellular media/environment. Stability assays with glutathione indicated a rapid change in the UV/Vis spectra of the Zn(II) thiosemicarbazone complex **4f-Zn**; however, elucidation of the underlying cause has so far not been pursued. Due to the entirely different concentration regimes, the cellular uptake methods are not directly comparable, and further investigations are necessary to draw a parallel regarding the kinetic stability of gallium complexes in cells. Further investigations into the [^18^F]-labeled **4d** derivative and into its cold analogues are underway in our laboratories.

### Cellular Viability Assays

Prior to the application of the asymmetric complexes in biological experiments, preliminary cytotoxicity studies were deemed necessary to determine the working concentration suitable for avoiding concomitant cell damage. A variety of techniques are used for the measurement of cell metabolic activity. The use of tetrazolium salts and more specifically the use of 2-(4,5-dimethyl-2-thiazolyl)-3,5-diphenyl-2*H*-tetrazolium bromide (MTT) has become a gold standard for the assessment of alterations in metabolic activity in cells.^37−39^ The exact mechanism for the action of MTT is not well understood, but the accepted mechanism suggests that the reduction of MTT dye is caused to a large degree by nicotinamide species (i.e., NAD(P)H) as cofactors, in combination with oxidoreductases, although additional reduction mechanisms, e.g., enzyme-free reduction in lipidic structures, have been proposed.

In this work, MTT assays were performed in a range of different concentrations to calculate the IC_50_ values for selected monothiosemicarbazones. The viability assays were carried out in both PC-3 and EMT-6 cell lines under normoxic and chemically induced hypoxic conditions (CoCl_2_·6H_2_O). The cells were plated in 96-well plates and incubated with the selected compounds in serum medium (1% DMSO) and at eight different concentrations for 48 h at 37 °C (SI). In the following steps, the cells were washed with phosphate-buffered saline (PBS) one to three times to remove compounds, and then the MTT dye was added and incubated for 2 h. MTT reagents were removed and the insoluble formazan species that resulted from this procedure were then solubilized in DMSO prior to absorbance measurement using a standard plate reader.

**Figure 13:**
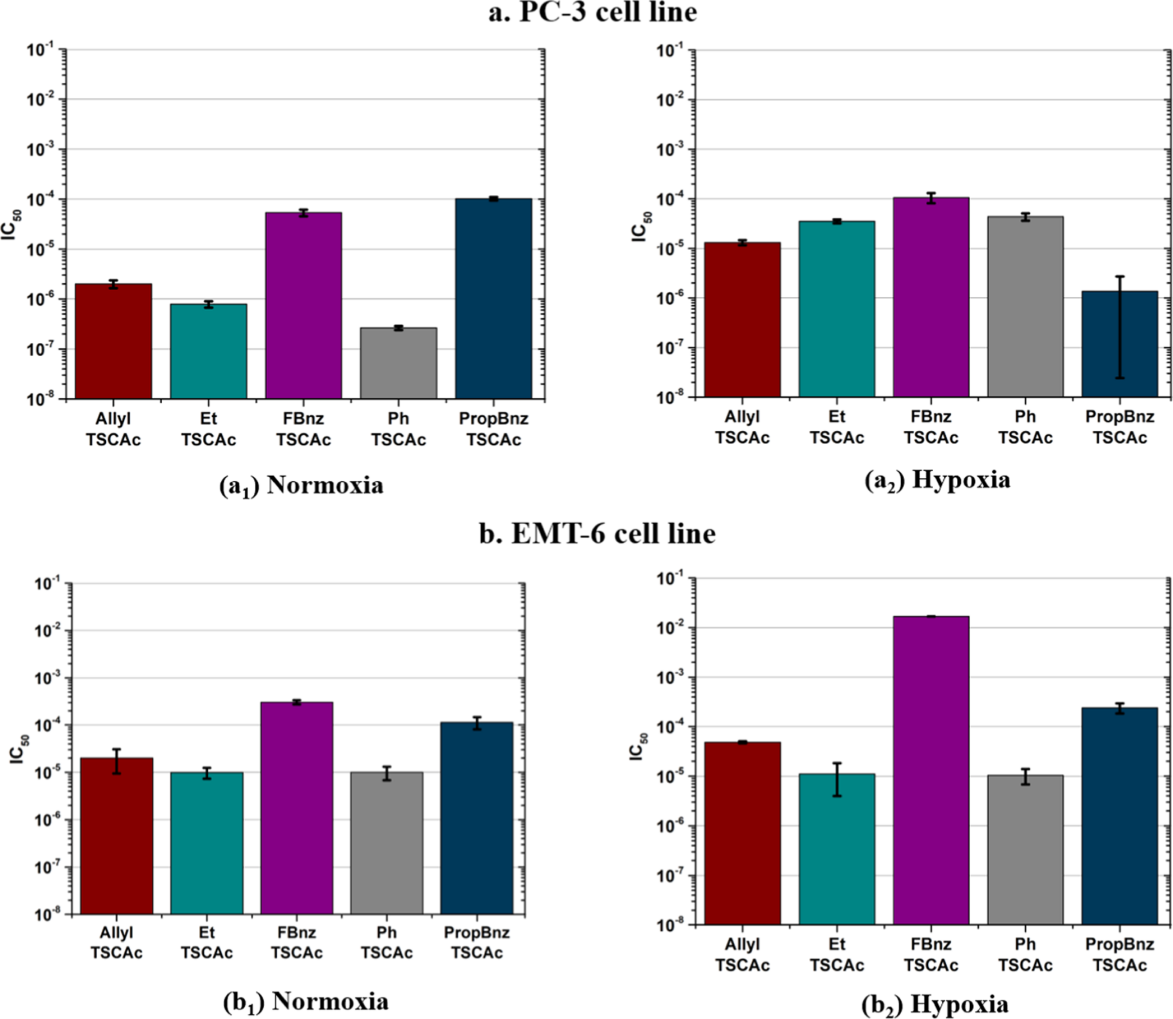
Estimation of IC_50_ values from MTT assays with PC-3 (a) and EMT-6 (b) cell lines under both normoxic (a_1_, b_1_) and hypoxic (a_2_, b_2_) conditions for a variety of mono(substituted) ligands. Error bars stand for standard error (±SE), calculated from six repeated measurements.

The IC_50_ values (i.e., the concentration of the compound tested where its response is reduced by 50%^40^) of the compounds investigated are within the range of 10–50 μM for the simpler substituted species (Et, Allyl, Ph) but variations occur with significant lack of cytotoxicity for compound **4a**, which is fluorinated, as shown in Figure 13. Compounds 4c*, **4d** and **4e** were found to have inhibitory effects, with IC_50_ values in the region of 1 mM. It can be noted that some of the mono(substituted) ligands present high cytotoxicity in the PC-3 cell line in normoxic conditions at 48 h. Nevertheless, the response seems to be only moderately cell-dependent and only a small variation in the cytotoxicity effects was observed in both cell lines used. The compound **4a** (FbnzTSCA) also shows a considerable reduction of cytotoxicity when incubated in the cell line EMT-6 under acute hypoxia conditions (chemically induced) compared to the other conditions tested. The compound **4a** (FbnzTSCA) shows a reduction of cytotoxicity when incubated in the cell line EMT-6 under chemically induced hypoxia compared to the other conditions tested. Further *in vitro* studies are planned to elucidate the potential of these compounds as imaging agents.

### Optical Properties and Cellular Imaging Assays

The understanding of the optical properties of the ligand in solution allows for the choice of an appropriate biological assay and microscopy conditions. The excitation–emission mapping for solutions of concentrations ranging between 100 μM and 1 mM in DMSO was measured to enable the optimum selection of lasers for cellular imaging work; however, these high concentrations could cause aggregation to occur. Unlike the aromatic bis(thiosemicarbazone) ligands previously investigated, the mono(thiosemicarbazone) ligands showed very weak fluorescence, with quantum yield for the compound **4f** estimated to be ca. 0.1%. Similarly, unlike the bis(thiosemicarbazone) ligands and complexes, Zn(II) complexes of mono(thiosemicarbazone) ligands only showed weak fluorescence emission and low solubility in aqueous media. The monothiosemicarbazone compounds were imaged in PC-3 (prostate carcinoma) using standard confocal fluorescence microscopy with one photon excitation at 405 or 488 nm. Experiments were carried out to ascertain if the weak fluorescence emission of these compounds was sufficient to render this traceable *in vitro*. As the Zn(II) compounds showed limited solubility and precipitation from cellular media, this precluded detailed investigations into these metal complexes.

**Figure 14:**
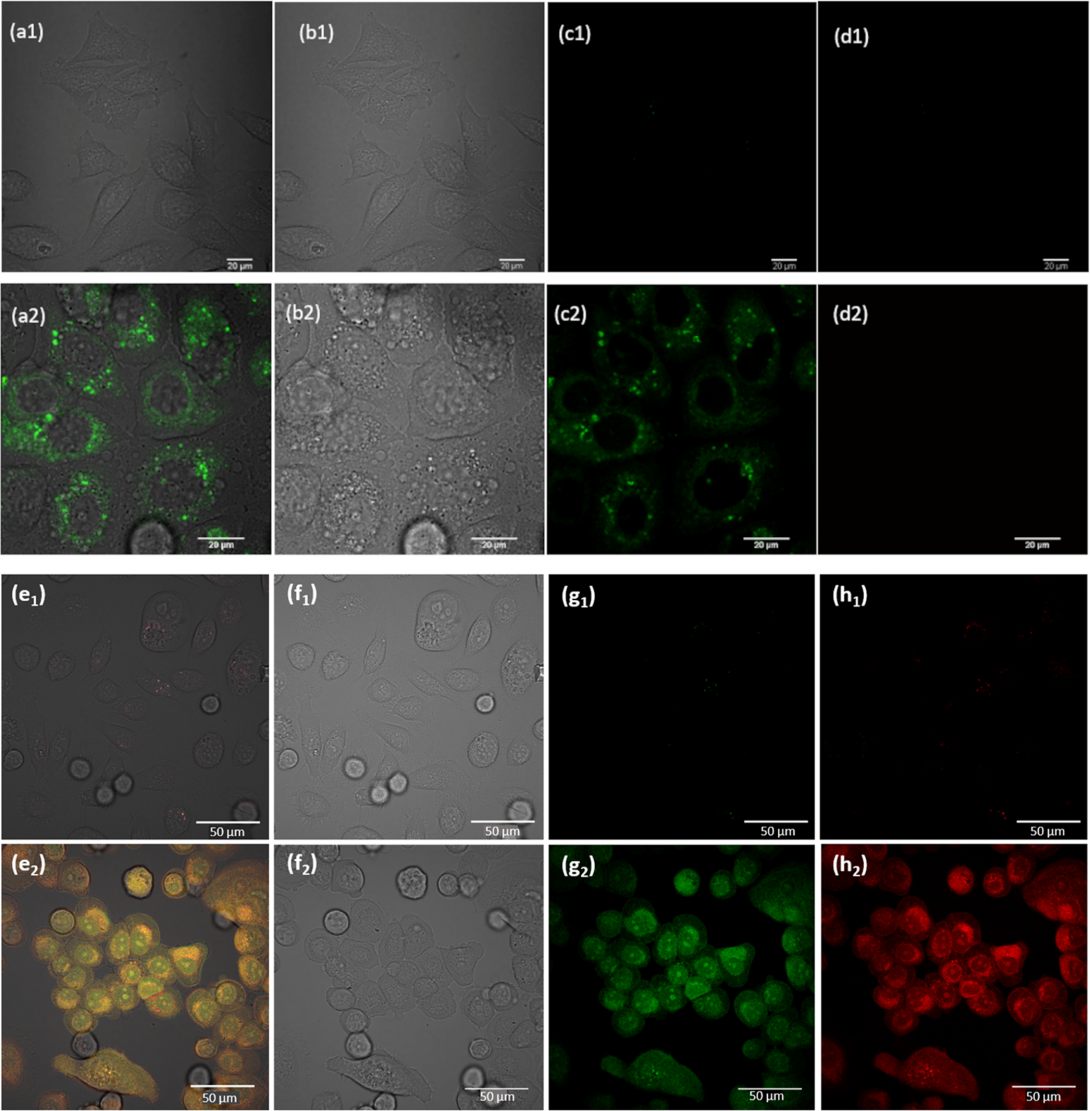
(a1–d1) Single-photon confocal microscopy images of PC-3-control experiments relevant to: (a2–d2) single-photon confocal microscopy images of compound **4a** in PC-3 cells (at 37 °C after 20 min incubation, 50 μM, in 1:99 DMSO/serum-free medium), where (a1) overlay of the blue, green, and red emission channels all at λ_ex_ = 405 nm (scale bar 20 μm); (b1) DIC channel, (c1) green emission channel, and (d1) red emission channel; (e1–h1) Single-photon confocal microscopy images of PC-3-control experiments relevant to (e2–h2): single-photon confocal microscopy images of amine derivative 4c* in PC-3 cells at 37 °C after 20 min incubation (50 μM, in 1:99 DMSO/serum-free medium), where (e2) overlay of the blue, green, and red channels all at λ_ex_ = 405 nm; (f2) DIC channel, (g2) green emission channel, and (h2) red emission channel (scale bar 20 μm). Further control experiments and additional fluorescence imaging assays are given in the SI.

The *in vitro* imaging was performed using confocal fluorescence microscopy (Figure 14) aiming to observe any changes in cell morphologies that could provide preliminary evidence of toxicity and to probe whether or not the complexes are traceable by fluorescence imaging in living PC-3 cells. In confocal microscopy, one photon excitation at 405 nm was most effective, alongside the 488 nm excitation, with the emission long-pass filtered at 515 nm. The cells were cultured using standard protocols as described in the Experimental Section and SI. Control experiments prior to incubation of cells with a compound of interest were obtained by confocal fluorescence imaging to ensure that the cell morphology remained unaltered before the imaging experiments, and to obtain a baseline for autofluorescence. No changes in cell morphology were observed by optical microscopy after 20 min incubation in control experiments.

Cellular imaging studies were performed using concentrations of mono(thiosemicarbazonato) compounds ranging from 25 to 100 μM in a DMSO/RPMI (Royal Park Memorial Institute) cell medium 1:99 solvent mix, whereby the final DMSO concentration on the imaging plate was generally kept lower than 1%. Then, the cells were carefully washed initially with prewarmed PBS (37 °C) and then with fetal calf serum (FCS)-free medium. The latter was used to remove any remaining noninternalized compound before the fluorescence imaging took place. All of the imaging studies were carried out in the absence of serum. Absence of serum is required to avoid potential background fluorescence and to ensure the suitability of the compound. Optimal imaging conditions for this class of compounds were found to be under the 405 nm laser line excitation rather than at 488 nm excitation and the corresponding emission was observed in the green channel for all compounds investigated. All of the compounds of interest were imaged in normoxic conditions, cultured as described in the Experimental Section. Some very subtle changes in cell morphology were observed by optical microscopy after 15−20 min incubation with the compounds with respect to the control, as evident from Figure 14 and the SI.

The viability of the cells was monitored by optical imaging prior to and during the imaging studies and PC-3 (prostate carcinoma) cells were cultured using standard protocols, analogous to earlier cellular investigations on thiosemicarbazones.^41^ The necessity to use concentrations as high as 50 μM in these studies was a result of the rather weak fluorescence emissions by comparison with organic, commercial dyes such as BODIPY or FITC.^42,43^

In previous experiments thiosemicarbazone-based ligands and complexes incubated in HeLa cells were found to possess a good colocalization with lysotracker, suggesting that these types of compounds are likely to enter the lysosome. Further investigations with colocalization dyes demonstrated that this simple compound did not localize in the mitochondria or nucleus. Previous experiments also showed that bis(substituted) ligand related to the family of compounds reported hereby possessed weak uptake in HeLa cells and which was barely detectable when incubated in FEK-4 cells, under similar conditions. The latter observations suggested that there could be a preference for thiosemicarbazones to enter cancerous cell lines over noncancerous cells.^33,34^ The uptake of compound **4a** followed an analogous pattern (Figure 14, micrographs a2–d2), and a typical lysosomal localization was observed in the PC3 cell line. Micrographs depicted in Figure 14e2–h2 represent typical confocal fluorescence microscopy images in PC-3 cells for the 4c*; interestingly, this −NH_2_ substituted compound was the only compound in the series that showed fluorescence emission. Uptake experiments in living cells seemed to indicate that for 4c*, a compound exhibiting a free −NH_2_ group, (Figure 14, micrographs e2–h2) the fluorescence emission was distributed throughout the cytoplasm. In previous experiments, related thiosemicarbazones were incubated with HeLa cells were found to accumulate in lysosomes by colocalization experiments with lysotracker dyes.^33,34^ However, conducting similar experiments including a wider panel of cancer cell lines would be needed to explore the potential of these compounds as theranostics under hypoxia^44^ and could provide more information regarding the compounds’ utility in cells in future investigations. Therefore further *in vitro* studies are ongoing in our laboratories to elucidate the potential of these compounds as theranostic agents.

## Conclusions

A new class of functional monothiosemicarbazones was successfully synthesized and characterized by ESI-MS, NMR spectroscopy, and single X-ray diffraction, and the investigation of their structural properties was accomplished. For all of the new functional thiosemicarbazones **4a**–**e** and their simpler analogues **4f**–**h**, the coordination properties toward zinc(II) have been probed. Significantly several novel functional thiosemicarbazides have been synthesized from the corresponding protected diamines under thermodynamic as well as under kinetic control, leading to a novel F-18 labeled monothiosemicarbazone. Several different derivatives were reacted with Zn(II), and the complexes were obtained in reduced times with respect to conventional heating under microwave irradiation conditions. The mono(substituted) ligands obtained with novel aromatic groups have been used for the synthesis of mono(thiosemicarbazonato) metal complexes of Zn(II) and Ga(III). Additional work is underway to expand the chemistry of these ligands to other metals, such as, for example, Cu(II). Selected examples have been radiolabeled for the first time with Gallium-68. *In vitro* studies indicated uptake in living cancer cells, however, unlike many other thiosemicarbazones, they generally appear to have little toxicity toward cancerous cell lines. Radiolabeling attempts indicated that the resulting gallium-68 labeled complexes can form in noteworthy radio-incorporation yields that are pH-dependent, giving rise to new complexes with limited kinetic stability. Unlike previously described bis(thiosemicarbazone) complexes, the monothiosemicarbazones described hereby showed no hypoxia selectivity under the conditions tested. Cytotoxicity assays have confirmed that the compounds in the series are showing increased toxicity in PC-3 vs EMT-6 cell lines. Their fluorescence emission properties in cells were analyzed showing uptake in living PC3 cells. Furthermore, the cell uptake experiments performed on the Ga-68 radiolabeled variant of compound **4a** revealed a variable cellular association under various incubation periods within cell lines with different genetic backgrounds. Optimization of the cellular uptake assays is needed regarding the incubation times, as well as the strategies for inducing hypoxia. Future studies will involve the exploration of alternative metal complexes in the series coupled with explorations toward gaining deeper understanding of the capabilities of these ligands to efficiently form kinetically stable metal complexes and of their potential for applications as theranostics.

## Experimental Section

All reagents and solvents were obtained from Aldrich Chemical Co. (Gillingham, U.K.), Fluoro Chem (Hadfield, U.K.), and Fisher (Acros; Geel Belgium) and used without further purification unless otherwise stated. Solvents with high purity or HPLC grade were obtained from Aldrich Chemical Co. (Gillingham, U.K.) and/or VWR (Radnor, PA). Milli-Q water was obtained from a Millipore Milli-Q purification system and anhydrous solvents from a PS-400-7 Innovative technologies SPS system.

Microwave reactions were conducted in a Biotage (Uppsala, Sweden) Initiator 2.5 reactor (0–450W depending on T) in stirred capped vials. The reaction mixtures were prestirred for 30 s and heated to the desired temperature by applying 400 W power.

Thin-layer chromatography (TLC) was carried out on Merck silica gel 60 F254 analytical plates (Matrix silica gel with aluminum support and fluorescent indicator 254, 0.2 mm thickness) and visualized by ultraviolet (UV) fluorescence (λ = 254, 366 nm) both: by charring with 10% KMnO_4_ in 1 M H_2_SO_4_ or by charring with 5% Na_2_SO_4_ in EtOH. The elution conditions for TLC were varied and are quoted for each compound.

NMR spectroscopy was performed using 300 MHz, 500 MHz Bruker (Banner Lane, U.K.) Advance NMR spectrometer and/or a 500 MHz Agilent automated system. Bruker and Agilent 500 Spectra were acquired at 500 MHz for ^1^H NMR, at 125 MHz for ^13^C{^1^H}NMR, and at 470 MHz for ^19^F{^1^H} NMR. All spectra were acquired at 298 K unless otherwise stated. Chemical shifts δ are reported in ppm and coupling constants (*J*) are reported in Hertz (Hz) with a possible discrepancy ≥0.2 Hz. Chemical shifts of solvent residues were identified as follows: CDCl_3_^1^:H, δ = 7.26,^13^C, δ = 77.0; DMSO-*d*_6_^1^:H, δ = 2.50^13^;C, δ = 39.5; D_2_O^1^:H, δ = 4.79. Peak multiplicities are referred to as follows: s, singlet; d, doublet; t, triplet; q, quartet; m, multiplet; br, broad.

Accurate mass spectrometry was carried out at the EPSRC National Mass Spectrometry Centre of Swansea University, U.K., using MALDI, ESI, and EI modes, as well as atmospheric solids analysis probe (ASAP) using the API ionization method.

Analytical HPLC was performed either on a Dionex Ultimate 3000 series HPLC system (Sunnyvale, California.) or on an Agilent 1100 series HPLC system (Agilent Technologies, Stockport, U.K.).

The Dionex system was equipped with a UV–vis diode array detector (measuring at eight wavelengths from 200 to 800 nm), using a Phenomenex Gemini C_18_ or a Waters Symmetry C_18_ column (250 mm × 4.6 mm, 110 Å or 100 Å, respectively) at a flow rate of 0.8 mL/min. The gradient elution was 0.1% TFA in Milli-Q water as solvent A and 0.1% TFA in MeCN as solvent B. A reverse gradient was applied starting with A at 95%, going to 5% of A for 7.5 min, then an isocratic step until 15 min and a gradient until 95% A, then kept for 18 min (Method A).

Alternatively, an HPLC method (B) using the Dionex Ultimate 3000 HPLC instrument with a UV–vis diode array detector measuring at eight wavelengths between 200 and 800 nm was applied: analytical HPLC chromatograms were acquired in RP mode using a Dionex C_18_ Acclaim column (5 μm, 4.6 mm × 150 mm). A 30 min gradient method using MeCN/H_2_O containing 0.1% TFA as mobile phases was applied: The gradient elution was 0.8 mL/min, with 0.1% TFA Milli-Q water as solvent A and 0.1% TFA MeCN as solvent B, as follows: start 95% A, reverse gradient until 5% A at 15 min, isocratic until 22.5 min, reverse gradient from 22.6 min 95% A, then hold to 30 min.

The Agilent 1100 series HPLC system (Agilent Technologies, Stockport, U.K.) was also applied. This was equipped with a UV detector (254 nm) and a LabLogic Flow-count radio-detector, using a Phenomenex Gemini C_18_ or a Waters Symmetry C_18_ column (250 mm × 4.6 mm, 110 Å or 100 Å, respectively) and Laura 3 software (LabLogic, Sheffield, U.K.) at a flow rate of 1 mL/min. The gradient elution was 0.1% TFA in Milli-Q water as solvent A and 0.1% TFA in MeCN as solvent B. A reverse gradient was applied starting with A at 95% for 2 min, going up to 5% A at 12 min, then an isocratic step until 14 min and gradient until 95% A at 16 min, then hold to 25 min (Method C).

IR spectroscopy was carried out on a PerkinElmer (Waltham, Massachusetts) frontier FTIR machine equipped with an attenuated total reflectance (ATR) module.

UV–vis spectroscopy was performed in 1 cm quartz cuvettes on a PerkinElmer (Waltham, Massachusetts) Lambda 35 UV–vis spectrometer controlled by UV-Winlab software.

Fluorescence spectroscopy was performed in 1 cm quartz cuvettes on a PerkinElmer (Waltham, Massachusetts) LS55 luminescence spectrometer controlled by FL-Winlab 4.0 software.

### Radiochemistry Methods

Radio-HPLC was performed either on an Agilent 1100 series HPLC system (Agilent Technologies, Stockport, U.K.) equipped with a γ-RAM Model 3 γ-detector (IN/US Systems, Inc., Florida) and Laura 3 software (LabLogic, Sheffield, U.K.). The gradient elution was 0.1% TFA in Milli-Q water as solvent A and 0.1% TFA in MeCN as solvent B. A reverse gradient was applied starting with A at 95% for 2 min, going up to 5% A at 12 min, isocratic level until 14 min and gradient until 95% A at 16 min, then hold to 25 min (Method C). Alternatively, characterization was carried out on an Agilent 1100 series HPLC system (Agilent Technologies, Stockport, U.K.) equipped with a γ-RAM Model 3 γ-detector (IN/US Systems, Inc., Florida) and Laura 3 software (LabLogic, Sheffield, U.K.). The gradient elution was 0.1% TFA in Milli-Q water as solvent A and 0.1% TFA in MeCN as solvent B. A reverse gradient was applied starting with A at 95% for 2 min, going up to 5% A at 12 min, isocratic level until 14 min and gradient until 95% A at 16 min, then hold to 25 min (Method D).

Radio-TLC was performed on a LabLogic PET/SPECT radio-TLC Scanner system (LabLogic, Sheffield, U.K.) using Laura software (LabLogic, Sheffield, U.K.). The radio-TLC was developed on Whatman 3MM with 0.35 M ethylenediaminetetraacetic acid (EDTA) as the mobile phase.

The positron-emitting radiotracer gallium-68 was extracted either from a SnO_2_-based column matrix ^68^Ge/^68^Ga generator (Department of Surgery and Cancer of Imperial College in London) using a 0.6 M HCl solution or produced through a cyclotron (PETIC, Cardiff, U.K., via the ^68^Zn(p,n)^68^Ga reaction) and extraction of [^68^Ga]GaCl_3_ from the target proceeded using a 0.1 M HCl solution.^20^

For the generator-produced ^68^Ga, the eluted aqueous ^68^Ga(III) was purified as follows; the activity was trapped in an SCX cartridge, which was already activated with 1 mL of HCl solution 0.1M and washed with 10 mL of water. Then, the ^68^GaCl_3_ was eluted from the cartridge with 0.8 mL of a THF/HCl (0.02M) solution (98%) or acetone/HCl (0.02M) solution (98%) and was further dried under nitrogen atmosphere.

The positron-emitting radiotracer [^18^F]fluoride was produced through a cyclotron (18O(p,n)18F, at Imanova, London, U.K.). Synthesis of [^18^F]fluorobenzaldehyde was performed, by Chris Barnes at Imperial College London, on the FASTlab *via* an established automated procedure.^31^ The [^18^F]fluoride was first dried and then was trapped on a Sep-Pak QMA-carbonate Light Cartridge. Then, it was eluted into the reactor using an eluent consisting of Kryptofix K_222_ and KHCO_3_ in acetonitrile: water (4:1). The content of the reactor was evaporated at 120 °C under vacuum and a low flow of nitrogen. The dried fluoride was then dissolved with 600 μL of anhydrous acetonitrile before being transferred to a Wheaton vial. The fluorination step was then performed manually. The anhydrous fluoride (400 μL) was added via syringe to a v-bottom vial containing 3 mg of the precursor. The vial was then heated at 90 °C for 15 min, resulting in consistently >98% radiochemical purity according to radio-HPLC, and it was then used to further labeling reactions. All of the radiolabeling experiments were repeated at least twice.

Microwave techniques for radiochemistry reactions involved the use of a Biotage (Uppsala, Sweden) Initiator 2.5 reactor (0–450 depending on T) in stirred capped vials. The reaction mixtures were prestirred for 30 s and heated to the desired temperature by applying the power of 400 W that was reduced and kept constant once the target temperature was reached.

### General Radiochemistry Procedures

A stock solution of the free monothiosemicarbazone was prepared as either 1 or 2 mg/mL in DMSO. Gallium experiments applied protocols optimized at Hammersmith Hospital, Imperial College London. In the optimized procedures, 10 mL of 0.1 M HCl was used to elute batches with activities ranging between 150 and 222 MBq (i.e., max 6 mCi) of ^68^Ga^3+^ from the generator and was subsequently trapped on a 30 mg/mL Strata X-C cartridge. This was eluted with 700 μL of 0.02M HCL/98% acetone and dried for 15 min under a stream of nitrogen at 110 °C. Next, 25 μL of 2 mg/mL if ligand or corresponding Zn(II) complex (for transmetallation approaches) in DMSO and 2 mL of HPLC-grade ethanol. The solution was heated for 30 min at 90 °C for conventional heating approaches, or microwave technologies were applied and optimized as described in the SI.

### *In Vitro* Experiments

#### General Cells Culturing Methods

Cells were cultured at 37 °C in a 5% CO_2_ incubator and harvested once >70% confluence had been reached. Both PC-3 (human prostatic small cell neuroendocrine carcinoma) and EMT-6 (mouse breast mammary adenocarcinoma) cell lines were cultured in phenol-free RPMI (Roswell Park Memorial Institute) in 1640 serum medium. The media contained 10% fetal calf serum (FCS), 0.5% penicillin/streptomycin (10,000 IU/mL/10,000 mg/mL), and 1% 200 mM l-glutamine. All steps were performed in the absence of phenol red.

The supernatant containing dead cell matter and excess protein was aspirated. The live adherent cells were then washed with 10 mL of phosphate-buffered saline (PBS) solution twice to remove any remaining media containing FCS. The cells were incubated in 3 mL of trypsin solution (0.25% trypsin in PBS) for 5 to 7 min at 37 °C. After trypsinization, 6 mL of medium containing 10% serum was added to inactivate the trypsin and the solution was centrifuged for 5 min (1000 rpm, 25 °C) to precipitate cells. The supernatant liquid was aspirated, and 5 mL of serum medium (10% FCS) was added to the cell matter left behind. The cells were counted using a hemocytometer and then seeded as appropriate.

#### Protocols for Cellular Assays under Chemically Induced Hypoxia

A stock solution of CoCl_2_ was prepared prior to each assay. COCl_2_·6H_2_O (4.76 mg, 237.9 g/mol) was dissolved in 1 mL of Milli-Q water to make a 20 mM CoCl_2_ stock solution.

##### Cytotoxicity Assays

Cells were prepared in a similar manner to that previously described. After cell subculturing, the cells were divided into aliquots (7000 cells per well), seeded in a 96-well plate, and cultured at 37 °C for 24 h in a conventional incubator (37 °C; 5% CO_2_) prior to the addition of CoCl_2_ stock solution (1 μL). The cells were incubated for a further 24 h. Then, the compounds were loaded and the cells were continuously cultured at 37 °C for a time period relative to each experiment (24, 48, or 72 h).

##### Fluorescence Assays

Cells were prepared in a similar manner as previously described. After cell subculturing, the cells were divided into aliquots (0.15 × 10^6^ cells per glass-bottom dish), seeded on a glass-bottom dish, and cultured at 37 °C for 48 h in a conventional incubator (37 °C; 5% CO_2_) prior to the addition of CoCl_2_ stock solution (10 μL/dish). The cells were incubated for a further 24 h and then treated as described in the fluorescence microscopy assay protocol.

#### Cellular Viability by MTT Assays

Cells (5–7 × 10^3^ cells per well) were seeded on a sterile 96-well plate and incubated for 48 h to adhere. All of the monosubstituted ligands were subsequently loaded at different concentrations (as mentioned earlier in Section S7.1.3) into the wells and cultured for another 48 h. Subsequently, the cells were washed three times with PBS and 3-(4,5-dimethylthiazol-2-yl)-2,5-diphenyltetrazolium bromide (MTT) was added [0.5 mg/mL, 90% serum-free medium (SFM)] + 10% PBS followed by a 2 h incubation. After aspiration, 100 μL of DMSO was added and the 96-well plates were read by an ELISA plate reader, Molecular Devices Versa Max (BN02877). The absorption wavelength was at 570 nm, and 630 nm wavelength was used as a reference.

#### General Fluorescence Microscopy Assays

PC3 cells were cultured in normoxia environment as previously described.^20^ The cells were then seeded in a single-well plate at least 48 h prior to the microscopy experiment (10,000 cells per well plate) where they were washed twice with PBS and incubated at 37 °C. Control fluorescence images were recorded before the addition of the compound. For both confocal and epi-fluorescence microscopy experiments, the desired compound was loaded as a DMSO/RPMI 1:99 ratio, solution mixture (100 μM) into the wells and the cells were incubated for 15 or 20 min at 37 °C. They were then carefully washed with phosphate-buffered saline (PBS) prewarmed to 37 °C, and then it was replaced by FCS-free medium to remove the noninternalized fluorescent dispersion prior to fluorescence imaging.

The intracellular radioactivity at the uptake assays using gallium-labeled compounds was counted in an LKB Wallac 1282 Compugamma Laboratory γ counter (PerkinElmer), while for GO nanocomposites, it was calculated at a Wizard2 2480 1-Detector γ counter (PerkinElmer). On bicinchoninic acid (BCA) assays, the cells were counted at a Sunrise absorbance reader (Tecan Trading AG, Switzerland). All of the obtained results were analyzed through the scientific two-dimensional (2D) graphing and statistics software GraphPad Prism (GraphPad Software, California).

#### Radioactive Cellular Assays

PC-3 and EMT6 cells (3 × 10^3^ cells) were seeded in six-well plates and incubated in normoxia and hypoxia environments. After treatments, the plates were aspirated and washed twice with warm PBS. Each well plate was then loaded with 1000 μL of [^68^Ga]Ga-labeled **4a** (**[**^**68**^**Ga]-4a**) in DMSO:PBS solution mixture (0.5:95.5) (3 MBq/mL; 81.1 μCi) and incubated for 1 h. During posterior incubation, the reaction was stopped by washing the wells with ice-cold PBS twice, followed by the addition of 1 mL of ice-cold, 0.1% Triton X-100, and 0.1 M NaOH Lysates. A homogeneous mixture was obtained by blending the components with up/down pipetting. Each dissolved cell (800 μL) was then transferred and capped into counting tubes for γ counting. The stock [^68^Ga]Ga-treated **4a** (**[**^**68**^**Ga]-4a**) solution was separated into three aliquots, each 10 μL, and placed in the counting tubes as standards. The intracellular radioactivity was immediately counted using an LKB Wallac 1282 Compugamma Laboratory γ counter (PerkinElmer). Lastly, protein concentration determination by BCA was carried out. This normalization of decay-corrected radioactivity counts per minute (CPM) to protein concentration, was required to give a measure of radiotracer uptake as % ID/mg of protein = CPM in 1 mL/(standard in mL·protein concentration in mg)·100%.

#### Synthesis of Thiosemicarbazones and Relevant Precursors

##### Methyl-(4-fluorobenzyl)carbamodithioate (**2a**)

Carbon disulfide (3.2 mL, 52.56 mmol) was added dropwise to a solution of 4-fluorobenzylamine (5 mL, 43.75 mmol) and triethylamine (7.4 mL, 52.56 mmol) in EtOH (60 mL) under stirring. The obtained slurry was allowed to react for 1.5 h at 25 °C, and then iodomethane (3.3 mL, 52.56 mmol) was added into the mixture and stirred for 1.45 h. Afterward, the excess solvent was removed under vacuum. The resulting residue was resuspended in EtAc and washed with 1 M HCl (100 mL), saturated NaHCO_3_ solution (200 mL), and distilled H_2_O (300 mL). The organic phase was then dried over MgSO_4_, and the excess solvent was removed under reduced pressure to afford 6.2706 g of methyl-*N*-(2-*tert*-butoxycarbonylaminoethyl)dithiocarbamate: this product was obtained as a yellowish powder in 67% yield. ^**1**^**H NMR** (δ, DMSO-*d*_6_, 25 °C): 10.40 (s, 1H, H4) 7.35–7.27 (m, 2H, H2/2′), 7.15 (appt, 2H, ^3^*J* = 8.9 Hz, H1/1′), 4.79 (d, 2H, ^3^*J* = 3.3 Hz, H3), 2.51 (s, 3H, H5). ^**13**^**C{**^**1**^**H} NMR** (δ, DMSO-*d*_6_, 25 °C): 198.3, 161.7 (d, *J*_C–F_ = 242.8 Hz), 134.0 (d, *J*_C–F_ = 3.1 Hz), 130.0 (d, *J*_C–F_ = 8.2 Hz), 115.5 (d, *J*_C–F_ = 21.3 Hz), 49.1, 17.8. ^**19**^**F{**^**1**^**H} NMR** (δ, DMSO-*d*_6_, 25 °C): −118.05. **Mass spectrum**: ESI-MS calcd for C_9_H_10_FNS_2_ [M + H]^+^: 216.0311 found 216.0312.
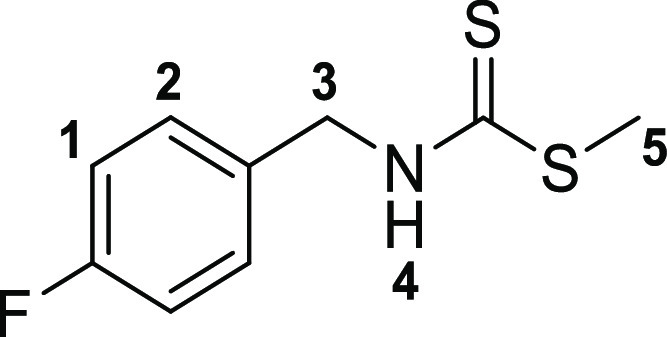


##### Methyl-(4-ethynyl benzyl)carbamodithioate (**2b**)

To a solution of ethynyl phenyl methenamine (0.6000 g, 4.56 mmol) and triethylamine (0.76 mL, 5.46 mmol) in EtOH (10 mL), carbon disulfide (0.33 mL, 5.46 mmol) was added dropwise under stirring. The obtained slurry was allowed to react for 1.5 h at 25 °C. Then, iodomethane (0.34 mL, 5.46 mmol) was added into the mixture and stirred for 1.45 h. Afterward, the excess solvent was removed under vacuum. The resulting residue was resuspended in EtAc and washed with 1 M HCl (50 mL), saturated NaHCO_3_ solution (50 mL), and distilled H_2_O (100 mL). The organic phase was then dried over MgSO_4_, and the excess solvent was removed under reduced pressure to afford 0.7404 g of methyl (4-ethynyl benzyl)carbamodithioate.
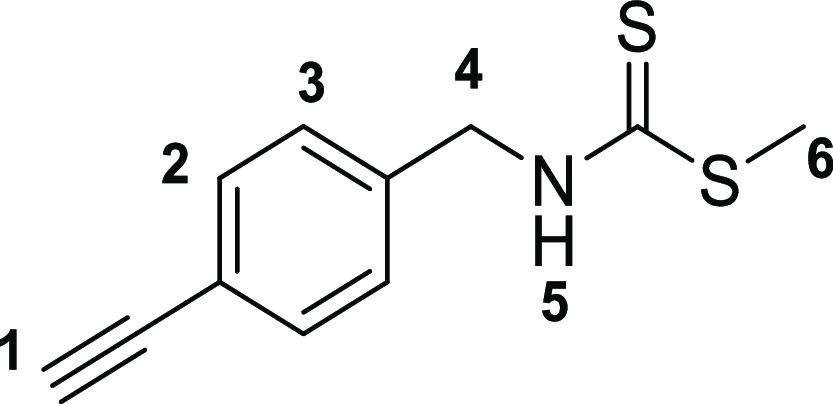


The product was obtained as a yellowish powder in 73% yield. ^**1**^**H NMR** (δ, DMSO-*d*_6_, 25 °C): 10.42 (s, 1H, H5), 7.43 (d, 2H, ^3^*J* = 8.2 Hz, H3/3′), 7.26 (d, 2H, ^3^*J* = 8.4 Hz, H2/2′), 4.84 (d, 2H, ^3^*J* = 4.8 Hz, H4), 4.16 (s, 1H, H1), 2.53 (s, 3H, H6). ^**13**^**C{**^**1**^**H} NMR** (δ, DMSO-*d*_6_, 25 °C): 198.3, 137.8, 133.2, 127.6, 121.1, 85.4, 80.2, 52.0, 18.1. **Mass spectrum**: ESI-MS calcd for C_11_H_11_NS_2_ [M + H]^+^: 222.0406 found 222.040.

##### Methyl-*N*-(2-*tert*-butoxycarbonylaminoethyl)dithiocarbamate (**2c**)

To a solution of *N*-Boc-ethylenediamine (1.72 mL, 11 mmol) and triethylamine (1.65 mL, 12 mmol) in EtOH (20 mL), carbon disulfide (0.72 mL, 12 mmol) was added slowly, dropwise, and under stirring. The formed slurry was allowed to react for 1.5 h at 25 °C. Then, iodomethane (0.8508 g, 6.0 mmol) was added into the mixture and stirred for 1.45 h. Afterward, the excess solvent was removed under reduced pressure. The resulting residue was resuspended in EtOAc and washed with 1 M HCl (50 mL), saturated NaHCO_3_ solution (50 mL), and distilled H_2_O (50 mL). The organic phase was then dried over MgSO_4_ and the excess solvent was removed under reduced pressure to afford 1.6312 g of methyl-*N*-(2-*tert*-butoxycarbonylaminoethyl)dithiocarbamate as a light yellow solid.
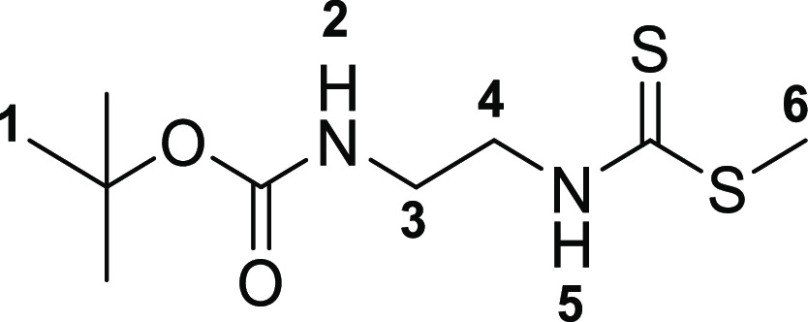


The product was obtained as a yellowish powder in 81% yield. ^**1**^**H NMR** (δ, CDCl_3_, 25 °C): 8.43 (s, 1H, H5), 5.16 (s, 1H, H2), 3.76 (d, ^3^*J* = 5.0 Hz, 2H, H4), 3.39 (d, ^3^*J* = 5.0 Hz, 2H, H3), 2.55 (s, 3H, H6), 1.41 (s, 9H, H1). ^**13**^**C{**^**1**^**H} NMR** (δ, CDCl_3_, 25 °C): 199.2, 157.8, 80.4, 49.4, 39.1, 28.4, 18.0. **Mass spectrum**: ESI-MS calcd for C_9_H_19_N_2_O_2_S_2_ [M + H]^+^: 251.0888; found: 251.0875.

##### *N*-(4-Fluorobenzyl)hydrazinecarbothioamide (**3a**)

To a solution of methyl (4-fluorobenzyl)carbamodithioate (6.2700 g, 29.12 mmol) in EtOH (60 mL), hydrazine monohydrate (1.8 mL, 36.45 mmol) was added dropwise, under stirring. The obtained slurry was allowed to react for 5 h under reflux (78 °C). Then, the excess solvent was removed under reduced pressure and the resulting residue was resuspended in chloroform. It was further purified by recrystallization from MeOH to afford 4.12 g of 4-*N*-(2-*tert*-*N*-(4-fluorobenzyl)hydrazinecarbothioamide) as white crystals. This product was obtained as white crystals in 71% yield. ^**1**^**H NMR** (δ, DMSO-*d*_6_, 25 °C): 8.76 (s, 1H, H5), 8.35 (brs, 1H, H4), 7.39–7.30 (m, 2H, H2/2′), 7.16–7.06 (m, 2H, H1/1′), 4.67 (d, 2H, ^3^*J* = 6.1 Hz, H3), 4.49 (appq, 2H, H6). ^**13**^**C{**^**1**^**H} NMR** (δ, DMSO-*d*_6_, 25 °C): 181.8, 161.4 (d, *J*_C–F_ = 241.7 Hz), 136.5 (d, *J*_C–F_ = 3.0 Hz), 129.7 (d, *J*_C–F_ = 8.0 Hz), 115.1 (d, *J*_C–F_ = 21.1 Hz), 45.7. ^**19**^**F{**^**1**^**H} NMR** (δ, DMSO-*d*_6_, 25 °C): −113.85. **Mass spectrum**: ESI-MS calcd for C_8_H_10_FN_3_S [M + H]^+^: 200.0652 found 200.0650.
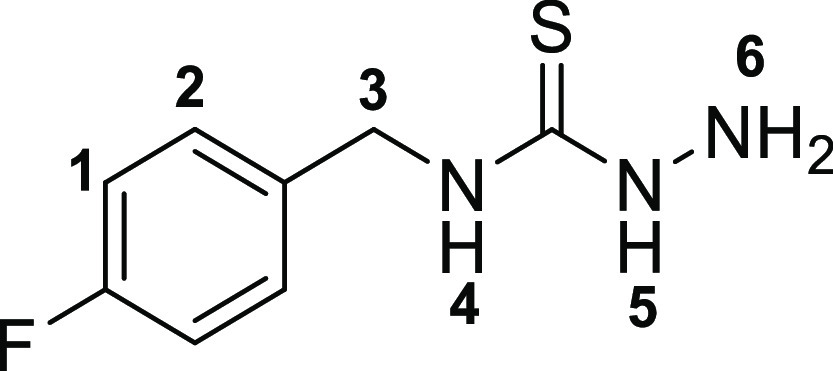


##### *N*-(4-Ethynyl benzyl)hydrazinecarbothioamide (**3b**)

Hydrazine monohydrate (0.21 mL, 4.16 mmol) was added dropwise to a solution of methyl (4-ethynyl benzyl)carbamodithioate (0.7400 g, 3.32 mmol) in EtOH (20 mL) under stirring. The obtained slurry was allowed to react for 5 h under reflux (78 °C). Then, the excess solvent was removed under reduced pressure and the resulting residue was resuspended in chloroform. It was further purified using a silica plug, eluting with CHCl_3_ (50 mL) and subsequently MeOH (100 mL). The methanolic fraction was concentrated under reduced, and then the compound crashed out from this conc. MeOH solution to afford 0.5452 g of *N*-(4-ethynyl benzyl)hydrazinecarbothioamide as an off-white solid. The product was obtained as a white powder in 80% yield. ^**1**^**H NMR** (δ, DMSO-*d*_6_, 25 °C): 8.81 (s, 1H, H6), 8.41 (brs, 1H, H5), 7.42 (appd, 2H, ^3^*J* = 8.1 Hz, H3/3′), 7.30 (appd, 2H, ^3^*J* = 8.1 Hz, H2/2′), 4.72 (d, 2H, ^3^*J* = 6.0 Hz, H4), 4.53 (bs, 2H, H7), 4.14 (s, 1H, H1). ^**13**^**C{**^**1**^**H} NMR** (δ, DMSO-*d*_6_, 25 °C): 181.9, 141.6, 127.9, 126.2, 120.2, 83.9, 80.9, 46.2. **Mass spectrum**: ESI-MS calcd for C_10_H_11_N_3_S [M + H]^+^: 206.0752 found 206.0749.
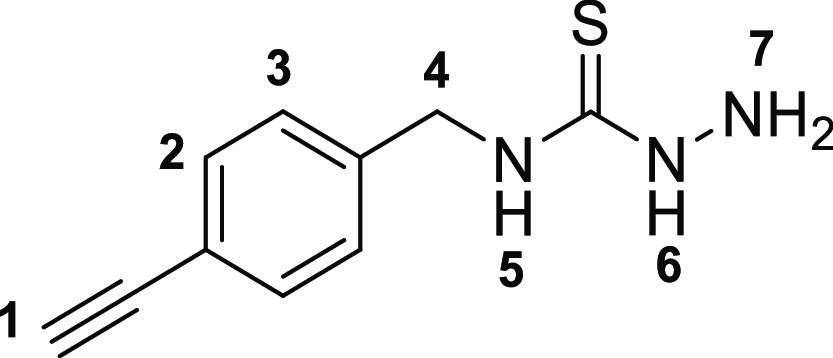


##### 4-*N*-(2-*tert*-Butoxycarbonylaminoethyl)-3-dithiocarbamate (**3c**)

Hydrazine monohydrate (0.39 mL, 8.09 mmol) was added dropwise to a solution of methyl-*N*-(2-*tert*-butoxycarbonylaminoethyl)dithiocarbamate (1.6206 g, 6.47 mmol) in EtOH (15 mL) under stirring. The obtained slurry was allowed to react for 2.5 h under reflux (78 °C). Then, the excess solvent was removed under reduced pressure and the resulting residue was resuspended in chloroform. It was further purified using a silica plug, washed with CHCl_3_ (50 mL) and MeOH (100 mL), and the methanolic fraction was concentrated under vacuum. Further purification was needed, and flash column chromatography was carried out using CHCl_3_/MeOH solution (1–10%) to afford 1.0312 g of 4-*N*-(2-*tert*-butoxycarbonylaminoethyl)-3-dithiocarbamate: this product was obtained as a white solid in 68% yield. ^**1**^**H NMR** (δ, DMSO-*d*_6_, 25 °C): 8.68 (brs, 1H, H6), 7.94 (brs, 1H, H5), 4.99 (brs, 1H, H2), 4.44 (s, 2H, H7), 3.49 (appq, 2H, ^3^*J* = 6.0 Hz, H3), 3.38 (appq, 2H, ^3^*J* = 5.9 Hz, H4), 1.38 (s, 9H, H1). ^**13**^**C{**^**1**^**H} NMR** (δ, DMSO-*d*_6_, 25 °C): 197.9, 156.1, 78.2, 46.4, 38.5, 28.6. **Mass spectrum**: ESI-MS calcd for C_8_H_18_N_4_O_2_S [M + H]^+^: 235.1221 found 235.1223.
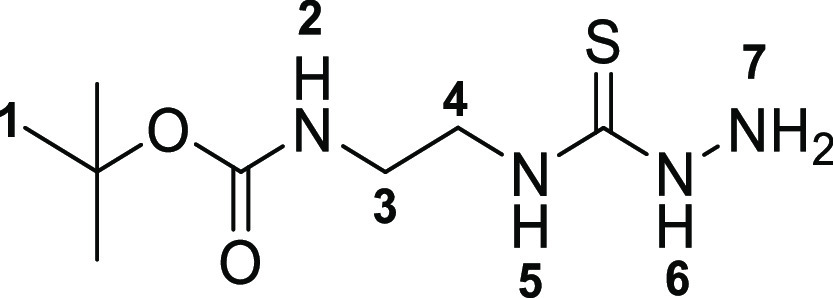


##### Mono(substituted)-4-F-benzyl-3-thiosemicarbazone-acenaphthenequinone (**4a**)

A microwave tube was filled with acenaphthenoquinone (0.5000 g, 2.74 mmol), 4-fluorobenzylamine thiosemicarbazide (**3a**, 0.5450 g, 2.74 mmol), and 15 mL of acetic acid. The mixture was reacted at 90 °C in the microwave for 20 min. The slurry was then allowed to cool, filtrated, and washed with Et_2_O. The precipitate was collected to afford 0.9564 g of the desired compound as a yellow solid (88%). No further purification was necessary. Crystals suitable for X-ray diffraction were obtained from DMSO after 2 days at room temperature.
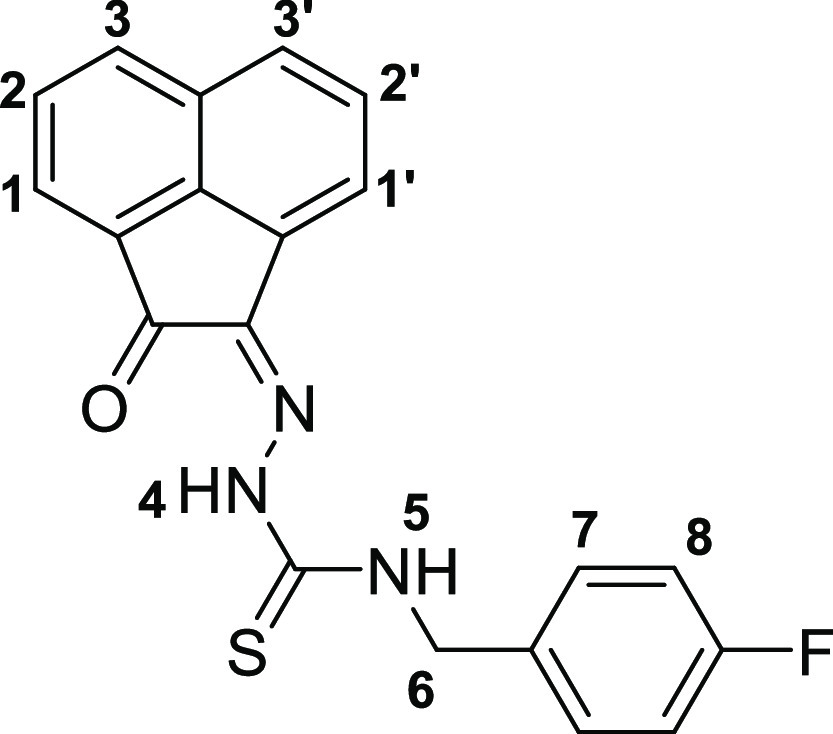


The product was obtained as a yellow powder in 88% yield. ^**1**^**H NMR** (δ, DMSO-*d*_6_, 25 °C): 12.68 (s, 1H, H4), 9.93 (t, 1H, ^3^*J* = 6.3 Hz, H5), 8.36 (d, 1H, ^3^*J* = 8.3 Hz, H3), 8.12 (d, 1H, ^3^*J* = 8.3 Hz, H3′), 8.08 (d, 1H, ^3^*J* = 6.9 Hz, H1), 7.98 (d, 1H, ^3^*J* = 6.9 Hz, H1′), 7.87 (t, 1H, *J* = 7.6 Hz, H2), 7.81 (t, 1H, J = 7.7 Hz, H2′), 7.43 (dd, 2H, *J* = 8.4 Hz, 5.5 Hz, H7/7′), 7.17 (t, 2H, *J* = 8.8 Hz, H8/8′), 4.88 (d, ^3^*J* = 6.3 Hz, H6). ^**13**^**C{**^**1**^**H} NMR** (δ, DMSO-*d*_6_, 25 °C): 189.0, 178.4, 161.8 (d, *J* = 242.5 Hz), 139.7, 138.0, 135.0 (d, *J* = 2.9 Hz), 133.2, 130.9, 130.5, 130.4, 129.9 (d, *J* = 8.1 Hz), 129.3, 129.1, 127.6, 123.0, 118.8, 115.6 (d, *J* = 21.4 Hz), 47.0. ^**19**^**F{**^**1**^**H} NMR** (δ, DMSO-*d*_6_, 25 °C): −115.74. **Mass spectrum**: ESI-MS calcd for C_20_H_14_FN_3_OS [M + H]^+^: 364.0920 found 364.0915. **IR** (ATR, cm^–1^): 3320, 3269, 1692, 1607, 1520, 1453, 1082, 1026, 853, 775. **HPLC (Method C)**: *R*_t_ = 11.33 min

##### Mono(substituted)-4-ethynyl benzyl-3-thiosemicarbazone-acenaphthenequinone (**4b**)

A microwave tube was filled with acenaphthenoquinone (0.2500 g, 1.37 mmol), 4-ethynyl-benzylamine thiosemicarbazide (0.2802 g, 1.37 mmol), and 10 mL of acetic acid. The mixture was reacted at 90 °C in the microwave for 20 min. The slurry was then allowed to cool, filtrated, and washed with Et_2_O. The precipitate was collected to afford 0.9468 g of the desired compound as a yellow solid (86%). Single crystals suitable for X-ray diffraction were obtained from DMSO after 2 days at room temperature.
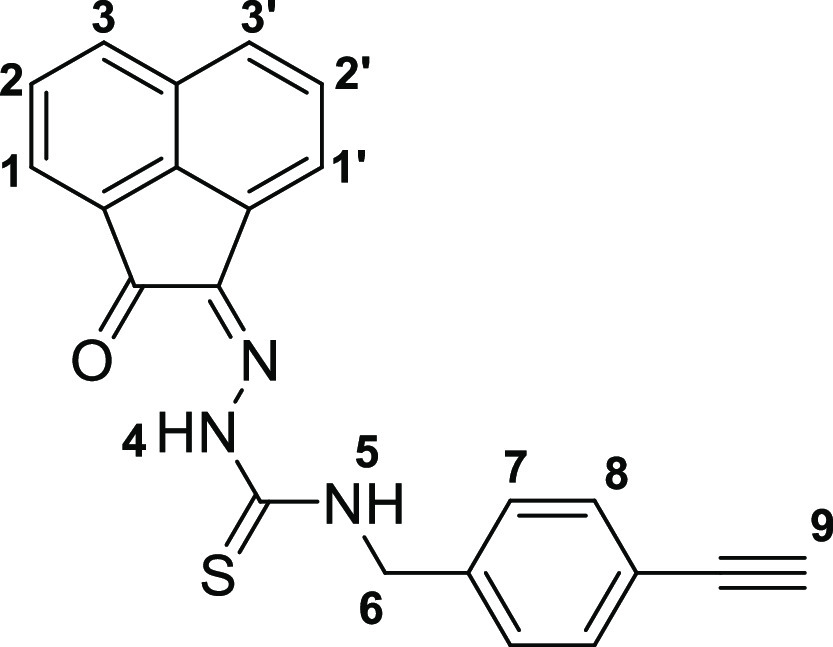


^**1**^**H NMR** (δ, DMSO-*d*_6_, 25 °C): 12.69 (s, 1H, H4), 9.97 (t, 1H, ^3^*J* = 5.8 Hz, H5), 8.35 (d, 1H, ^3^*J* = 8.1 Hz, H1), 8.12 (d, 1H, ^3^*J* = 12.9 Hz, H3′), 8.08 (d, 1H, ^3^*J* = 11.3 Hz, H3), 7.97 (d, 1H, ^3^*J* = 6.9 Hz, H1′), 7.90–7.78 (m, 2H, *J* = 7.6 Hz, H2, H2′), 7.43 (dd, 4H, *J* = 7.7 Hz, 16.1 Hz, H7/7′, H8/8′) 4.91 (d, 2H, ^3^*J* = 5.6 Hz, H6), 4.16 (s, 1H, H9). ^**13**^**C{**^**1**^**H} NMR** (δ, DMSO-*d*_6_, 25 °C): 188.9, 178.4, 139.8, 139.6, 138.0, 133.2, 132.1, 130.8, 130.4, 130.3, 129.2, 129.0, 127.9, 127.5, 122.9, 120.7, 118.7, 83.8, 81.1, 47.4. **Mass spectrum**: ESI-MS calcd for C_22_H_15_N_3_OS [M + H]^+^: 507.1497 found 507.1483. **IR** (ATR, cm^–1^): 3259, 3236, 2950, 1684, 1527, 1452, 1082, 1026. **HPLC (Method C)**: *R*_t_ = 11.37 min.

##### Mono(substituted)-4-Boc-diethylamine-3-thiosemicarbazone-acenaphthenequinone (**4c**)

A microwave tube was filled with acenaphthenoquinone (0.5000 g, 2.74 mmol), 4-Boc-diethylamine thiosemicarbazide (0.640 g, 2.74 mmol), and 15 mL of acetic acid. The mixture was reacted at 90 °C in the microwave for 20 min. The slurry was then allowed to cool, filtered, and washed with Et_2_O. The precipitate was collected to afford 0.9564 g of the desired compound as a yellow solid (88%).
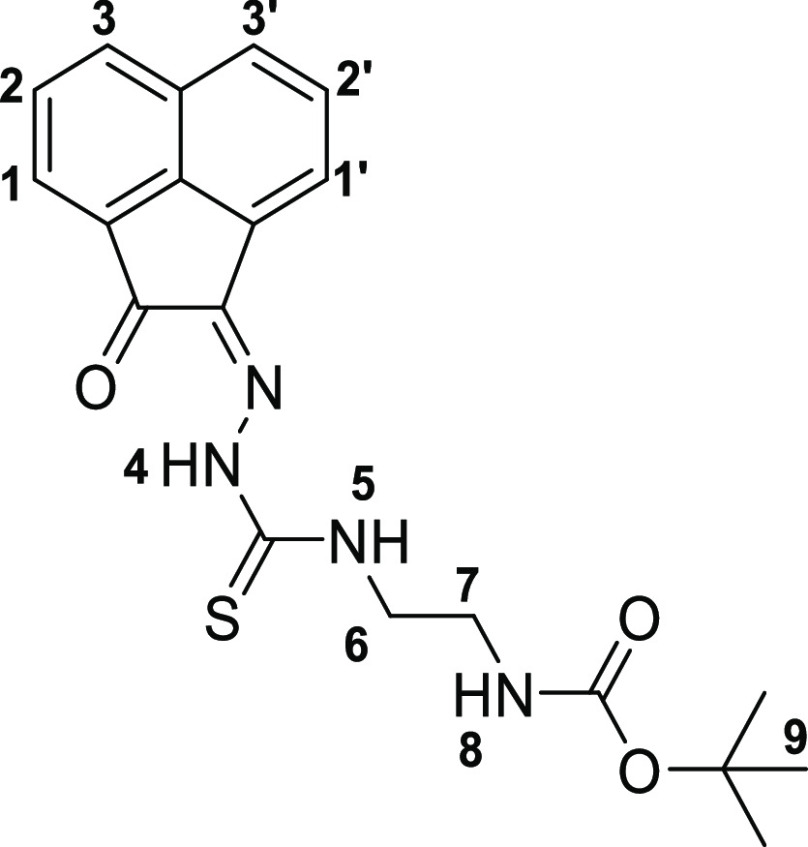


Single crystals suitable for X-ray diffraction were obtained from DMSO after 2 days at room temperature.

^**1**^**H NMR** (δ, DMSO-*d*_6_, 25 °C): 12.61 (s, 1H, H4), 9.41 (t, ^3^*J* = 5.6 Hz, 1H, H5), 8.37 (dd, ^3,4^*J* = 8.2, 0.7 Hz, 1H, H3), 8.13 (dd, ^3,4^*J* = 8.4, 0.7 Hz, 1H, H3′), 8.09 (dd, ^3,4^*J* = 7.1, 0.7 Hz, 1H, H1), 8.01 (d, ^3^*J* = 7.0 Hz, 1H, H1′), 7.87 (dd, ^3,3^*J* = 8.2, 7.0 Hz, 1H, H2), 7.83 (dd, ^3,3^*J* = 8.3, 7.0 Hz, 1H, H2′), 7.06 (t, ^3^*J* = 5.7 Hz, 1H, H8), 3.65 (q, ^3^*J* = 5.9 Hz, 2H, H6), 3.25 (q, ^3^*J* = 6.2 Hz, 2H, H7), 1.38 (s, 9H, H9). ^**13**^**C{**^**1**^**H} NMR** (δ, DMSO-*d*_6_, 25 °C): 188.5, 177.6, 156.2, 139.5, 137.2, 132.8, 130.5, 130.0, 129.9, 128.9, 128.4, 127.1, 122.5, 118.2, 78.0, 44.9, 39.2, 28.2. **Mass spectrum**: ESI-MS calcd for C_20_H_22_N_4_NaO_3_S [M + Na]^+^: 421.1310; found: 421.1329. **IR** (ATR, cm^–1^): 3384, 3326, 3257, 2980, 1719, 1685, 1670, 1512, 1480. **HPLC (Method C)**: *R*_t_ = 10.04 min.

##### Mono-(4-(2-aminoethyl)-3-thiosemicarbazone)-acenaphthenequinone (4c*)

A suspension of the compound **4c** (1.63g, 4.1 mmol) in 80 mL of formic acid was stirred at room temperature for 3 h. The solvent was removed under reduced pressure, and the compound was washed with toluene. The desired product was obtained as a yellow powder in 91% yield.
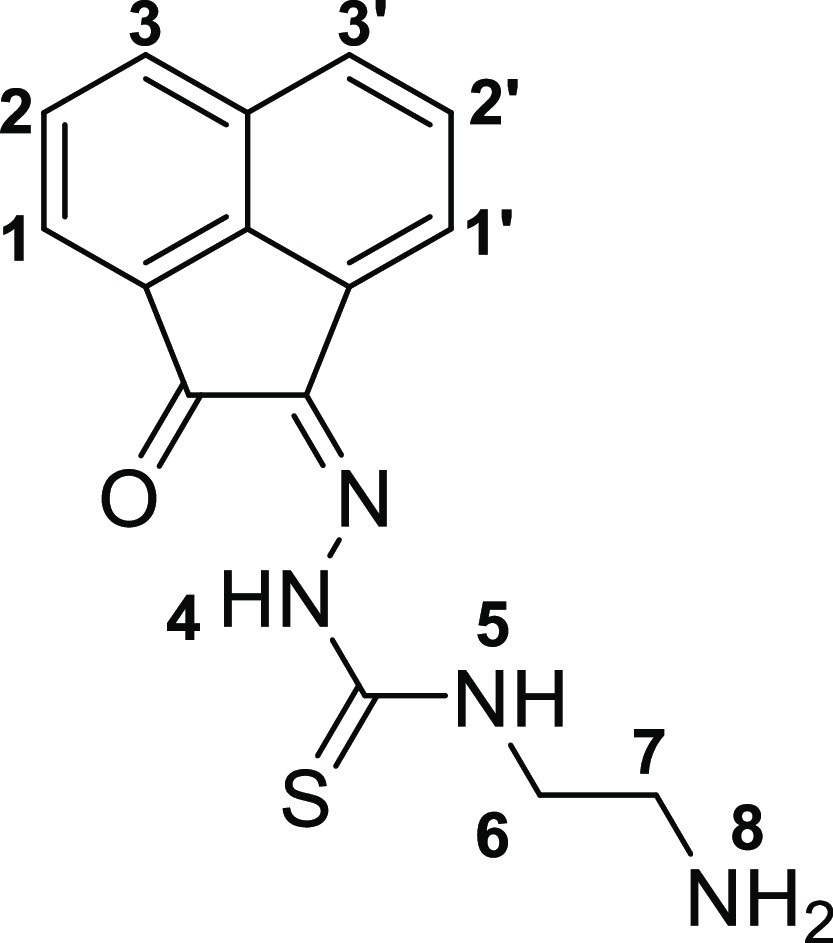


The desired product was obtained as a yellow powder in 91% yield. ^**1**^**H NMR** (δ, DMSO-*d*_6_, 25 °C): 12.72 (s, 1H, H4), 9.68 (t, ^3^*J* = 5.8 Hz, 1H, H5), 8.39 (dd, ^3,4^*J* = 8.2, 0.7 Hz, 1H, H3), 8.29–8.19 (m, 2H, H8), 8.16 (dd, ^3,4^*J* = 8.5, 0.7 Hz, 1H, H3′), 8.11 (dd, ^3,4^*J* = 7.0, 0.7 Hz, 1H, H1), 8.10 (dd, ^3,4^*J* = 7.0, 0.7 Hz, 1H, H1′), 7.89 (dd, ^3,3^*J* = 8.2, 7.1 Hz, 1H, H2), 7.85 (dd, ^3,3^*J* = 8.4, 7.0 Hz, 1H, H2′), 3.95 (q, ^3^*J* = 6.3 Hz, 2H, H6), 3.11 (s, 2H, H7). ^**13**^**C{**^**1**^**H} NMR** (δ, DMSO-*d*_6_, 25 °C): 188.6, 178.0, 139.3, 137.6, 132.9, 130.4, 129.9, 129.9, 128.9, 128.6, 127.2, 122.5, 118.6, 41.8, 37.7. **Mass spectrum**: ESI-MS calcd for C_15_H_15_N_4_OS [M + H]^+^: 299.0967; found: 299.0959. **IR** (ATR, cm^–1^): 3330, 3255, 2836, 1693, 1523, 1467, 1452, 1050. **HPLC** (**Method C**): *R*_t_ = 7.42 min.

##### Mono-(2-(4-fluorobenzylidene)aminoethyl)-3-thiosemicarbazone-acenaphthenequinone (**4d**)

A suspension of the compound 4c* (1.50 g, 5.03 mmol), 1 equiv of 4-(fluorobenzaldehyde) (539.30 μL, 5.03 mmol) in MeOH (20 mL), and three drops of triethylamine was placed in a 20 mL microwave tube, and the mixture was reacted at 90 °C in the microwave for 20 min. The reaction mixture was allowed to cool to room temperature without stirring and then the precipitate was filtrated and washed with Et_2_O and hexane. The solvent was removed under reduced pressure affording the desired compound. The product was obtained as a yellow powder in 62% yield. ^**1**^**H NMR** (δ, DMSO-*d*_6_, 25 °C): 12.15 (s, 1H, H4), 9.37 (bt, 1H, H5), 8.44 (s, 1H, H8), 8.37 (d, ^3,4^*J* = 8.2 Hz, 1H, H3), 8.13 (d, ^3,4^*J* = 8.2 Hz, 1H, H3′), 8.09 (d, ^3,4^*J* = 7.0 Hz, 1H, H1), 7.91–7.83 (m, 4H, H1′, H2, H9, H9′), 7.80 (t, ^3,4^*J* = 7.6 Hz, 1H, H2′), 7.29 (t, ^3,3^*J* = 8.8 Hz, 2H, H10, H10′), 3.94 (d, ^3^*J* = 5.7 Hz, 2H, H6), 3.91 (s, 2H, H7). ^**13**^**C{**^**1**^**H} NMR** (δ, DMSO-*d*_6_, 25 °C): 189.0, 178.0, 164.1 (d, *J*_C–F_ = 248.1 Hz), 161.6, 139.6, 137.8, 133.3, 133.1 (d, *J* = 2.8 Hz), 130.9, 130.7 (d, *J* = 8.8 Hz), 130.4, 130.3, 129.3, 129.1, 127.6, 123.0, 118.6, 116.1 (d, *J* = 21.8 Hz), 59.0, 45.3. ^**19**^**F{**^**1**^**H} NMR** (δ, DMSO-*d*_6_, 25 °C): −109.86. **Mass spectrum**: ESI-MS calcd for C_22_H_17_F_1_N_4_OS [M + H]^+^: 405.1185; found:405.1193. **IR** (ATR, cm^–1^): 3318, 3250, 1681, 1602, 1527, 1481, 1178, 1028, 937, 825, 791, 773. **HPLC** (**Method C**): *R*_t_ = 7.68 min.
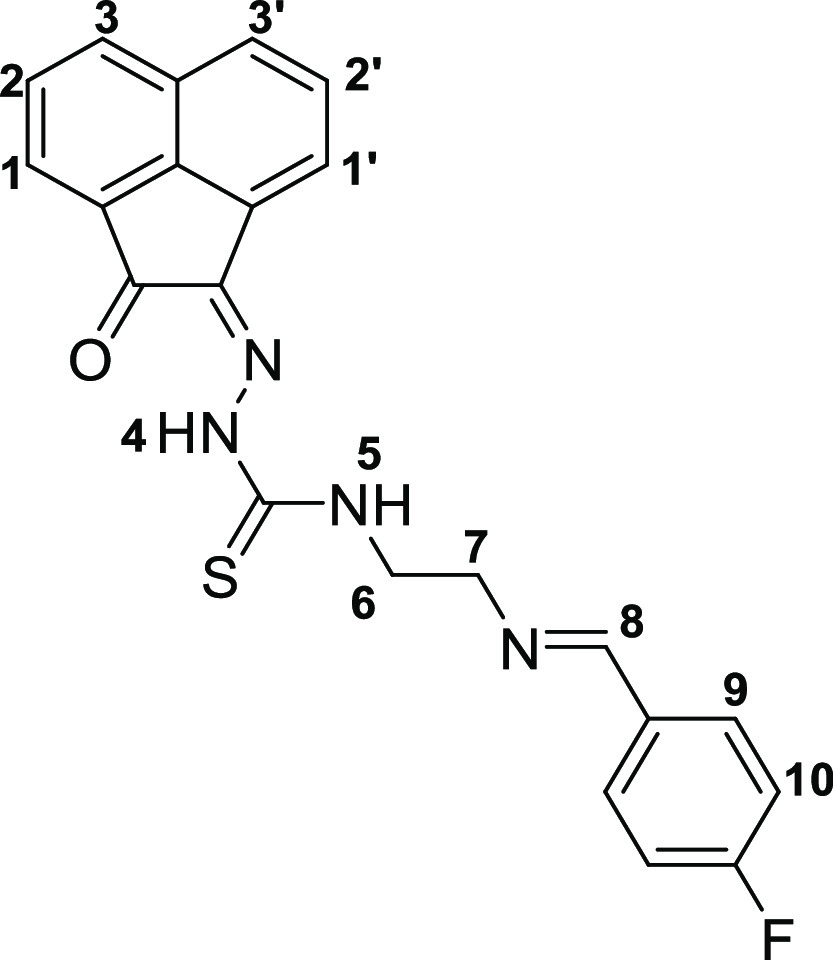


##### Mono-(2-(4-ethynylbenzylidene)aminoethyl)-3-thiosemicarbazone-acenaphthenequinone (**4e**)

A suspension of the compound 4c* (1.50 g, 5.03 mmol), 1 equiv of 4-(ethynylbenzaldehyde) (654.30 mg, 5.03 mmol) in MeOH (20 mL) was placed in a 20 mL microwave tube, and the mixture was reacted at 90 °C in the microwave for 20 min. The reaction mixture was allowed to cool to room temperature without stirring, and then the precipitate was filtrated and washed with Et_2_O and hexane. The solvent was removed under reduced pressure affording the desired compound. The product was obtained as a yellow powder in 59% yield. ^**1**^**H NMR** (δ, DMSO-*d*_6_, 25 °C): 12.64 (s, 1H, H4), 9.37 (bt, 1H, H5), 8.46 (s, 1H, H8), 8.37 (d, ^3,4^*J* = 8.2 Hz, 1H, H3), 8.13 (d, ^3,4^*J* = 8.1 Hz, 1H, H3′), 8.09 (d, ^3,4^*J* = 7.0 Hz, 1H, H1), 7.88 (t, ^3,4^*J* = 7.6 Hz, 1H, H2), 7.85 (d, ^3,4^*J* = 6.9 Hz, 1H, H1′), 7.82–7.78 (m, 3H, H2′, H9, H9′), 7.56 (d, ^3,4^*J* = 8.0 Hz, H10, H10′), 4.35 (s, 1H, H8), 3.99–3.89 (m, 4H, H6, H7). ^**13**^**C{**^**1**^**H} NMR** (δ, DMSO-*d*_6_, 25 °C): 188.9, 178.0, 162.2, 139.6, 137.9, 136.6, 133.3, 132.5, 130.9, 130.4, 130.3, 129.3, 129.1, 128.6, 127.6, 124.4, 123.0, 118.6, 83.6, 83.12, 59.2, 45.3. **Mass spectrum**: ESI-MS calcd for C_24_H_18_N_4_OS [M + H]^+^: 411.1280; found: 411.1291. **IR** (ATR, cm^–1^): 3279, 3213, 1685, 1606, 1532, 1476, 1180, 1024, 935, 828, 777. **HPLC (Method C)**: *R*_t_ = 7.683 min.
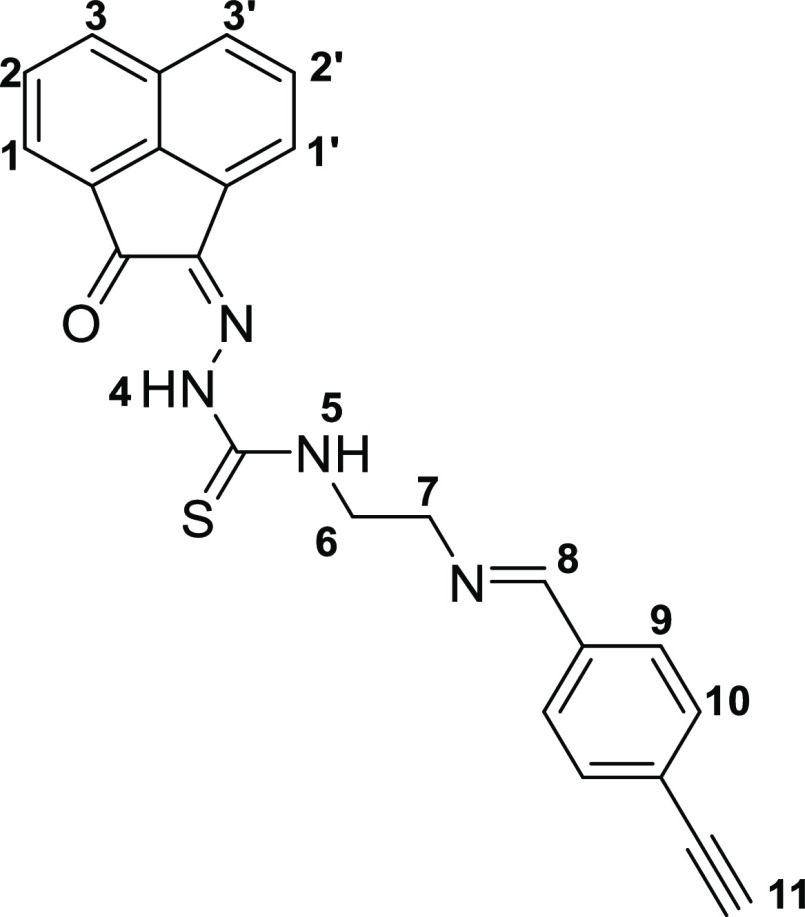


##### Mono(substituted)-3-ethyl-4-thiosemicarbazone-acenaphthenequinone (**4f**)

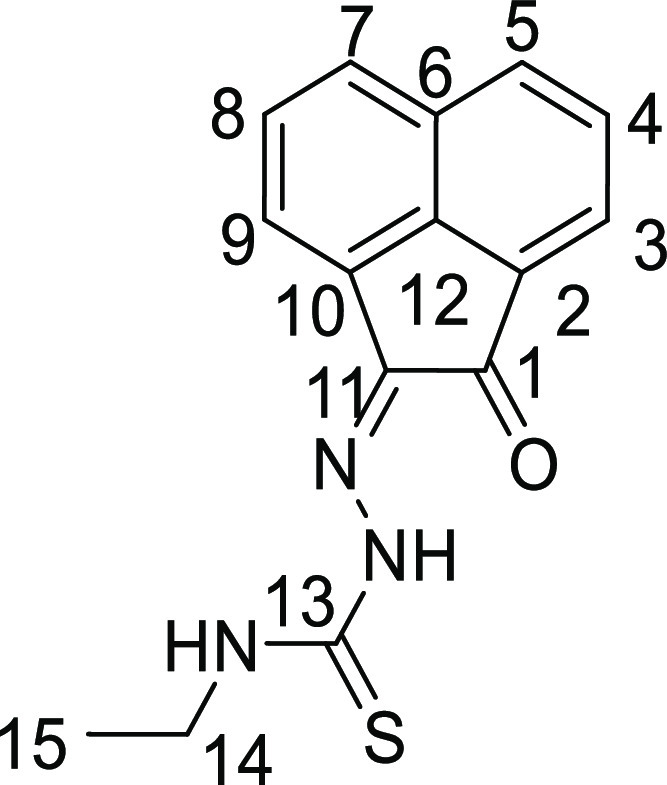



This microwave irradiation method was adapted from ref (26), and optimization protocols are given in the SI.

A microwave tube was filled with acenaphthenoquinone (0.5000 g, 2.74 mmol), 4-ethylthiosemicarbazide (0.3259 g, 2.74 mmol), 15 mL of EtOH, and 0.1 mL of concentrated HCl. The mixture was reacted at 90 °C in the microwave for 10 min. The slurry was then allowed to cool, filtrated, and washed with Et_2_O. The precipitate was collected to afford the desired compound (**4f**) as an orange solid (84%). No further purification was necessary. Crystals suitable for X-ray diffraction were obtained from DMSO after 2 days at room temperature.

^**1**^**H NMR** (300 MHz, DMSO-*d*_6_) δ_H_: 12.59 (s, 1H, N-N***H***); 9.44 (t, 1H, ^3^*J* = 5.44 Hz, N***H***-Et); 8.36 (d, 1H, ^3^*J* = 8.27 Hz, ***H***^***9***^); 8.12 (overlapping d, 1H, ^3^*J* = 13.7 Hz, ***H***^***5***^), 8.08 (overlapping d, 1H, ^3^*J* = 12.1 Hz, ***H***^***7***^); 7.99 (d, 1H, ^3^*J* = 7.0 Hz, ***H***^**3**^); 7.84 (m, 2H, ***H***^***8***^,***H***^***4***^); 3.66 (q, 2H, NH-C***H***_***2***_-CH_3_); 1.20 (t, H, ^3^*J* = 6.9 Hz, R-C***H***_**3**_).

^**13**^**C NMR** (75 MHz, DMSO-*d*_6_) δ_C_: 188.9 (***C***=O); 177.2 (NHR-***C***S–NHEt); 139.4 (***C***=N-R); 137.53 (***C***^***12***^); (***C***^***2***^); 133.1 (***C***^***9***^); 130.9 (***C***^***6***^); 130.5 (***C***^***2***^); 130.3 (***C***^***10***^); 129.3 (***C***^***4***^); 129.0 (***C***^***8***^); 127.41 (***C***^***7***^); 122.84 (***C***^***5***^); 118.63 (***C***^**3**^); 14.4 (RCH_2_-***C***H_3_); ppm.

**Mass spectrometry:** ASAP for C_15_H_13_N_3_OS, calcd for ([M + H]^+^) 284.0858 found 284.0855.

**IR** (solid): ν (cm^–1^) 3298, 3278, 2976, 1682, 1605, 1533, 1471, 1056, 1027 cm^–1^.

**HPLC** (Method C): *R*_f_ = 10.71 min (or Method B: *R*_f_ = 17.50 min).

##### Mono(substituted)-3-allyl-4-thiosemicarbazone-acenaphthenequinone (**4g**)

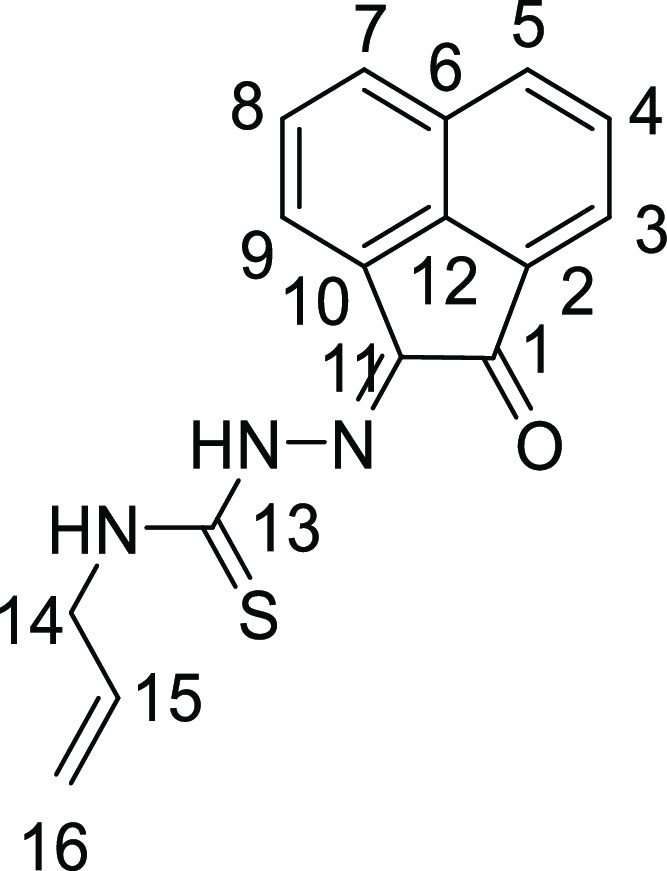



This microwave irradiation method was adapted from ref (26), and optimization protocols are given in the SI.

A microwave tube was filled with acenaphthenoquinone (0.5000 g, 2.74 mmol), 4-phenylthiosemicarbazid (0.3595 g, 2.74 mmol), 15 mL of EtOH, and 0.1 mL of concentrated HCl. The mixture was reacted at 90 °C in the microwave for 10 min. The slurry was then allowed to cool, filtrated, and washed with Et_2_O. The precipitate was collected to afford the desired compound (**4g**) as an orange solid (58%). No further purification was necessary. Crystals suitable for X-ray diffraction were obtained from DMSO after 2 days at room temperature.

^**1**^**H NMR** (300 MHz, DMSO-*d*_6_) δ_H_: 12.63 (s, 1H, N-N*H*); 9.59 (t, 1H, ^3^*J* = 5.7 Hz, N**H**-Allyl); 8.36 (d, 1H, ^3^*J* = 8.0 Hz, ***H***^***9***^); 8.11 (overlapping d, 1H, ^3^*J* = 14.7 Hz, ***H***^***7***^); 8.08 (overlapping d, 1H, ^3^*J* = 13.4 Hz, ***H***^***5***^); 7.99 (d, 1H, ^3^*J* = 7.0 Hz, ***H***^**3**^); 7.84 (m, 2H, ***H***^***4***^, ***H***^***8***^), 5.96 (m, 1H, R-C***H***-CH_2_), 5.22 (overlapping d, 1H, ^3^*J* = 23.5 Hz, RCH-C***H***_***2***_^***a***^); 5.16 (overlapping d, 1H, ^3^*J* = 16.7 Hz, RCH-C***H***_***2***_^***b***^); 4.30 (t, 1H, ^3^*J* = 4.9 Hz, NHC*H*_***2***_-**C**H-CH_2_) ppm

^**13**^**C NMR** (125 MHz, DMSO-*d*_6_) δ_C_: 188.5 (***C***=O); 177.5 (NHR-***C***S–NHAllyl); 139.15 (***C***^**11**^); 137.3 (***C***^**12**^); 134.0 (R-***C***H-CH_2_); 132.8 (***C***^**9**^); 130.5 (***C***^**6**^); 130.1 (***C***^**2**^); 129.9 (***C***=N-R); 128.9 (***C***^**4**^); 128.6 (***C***^**8**^); 127.1 (***C***^**7**^); 122.5 (***C***^**5**^); 118.4 (***C***^**3**^); 116.3 (RCH-***C***H_2_); 46.5 (NH-***C***H_2_-R) ppm.

**Mass spectrometry:** ASAP for C_16_H_13_N_3_OS, calcd for ([M – H]^+^) 294.0691 found 294.0696.

**IR** (solid): ν (cm^–1^) 3296, 3265, 2951, 1685, 1597, 1533, 1477, 1051, 1027 cm^–1^.

**HPLC** (Method C): *R*_f_ = 10.82 min.

##### Mono(substituted)-4-phenyl-3-thiosemicarbazone-acenaphthenequinone (**4h**)

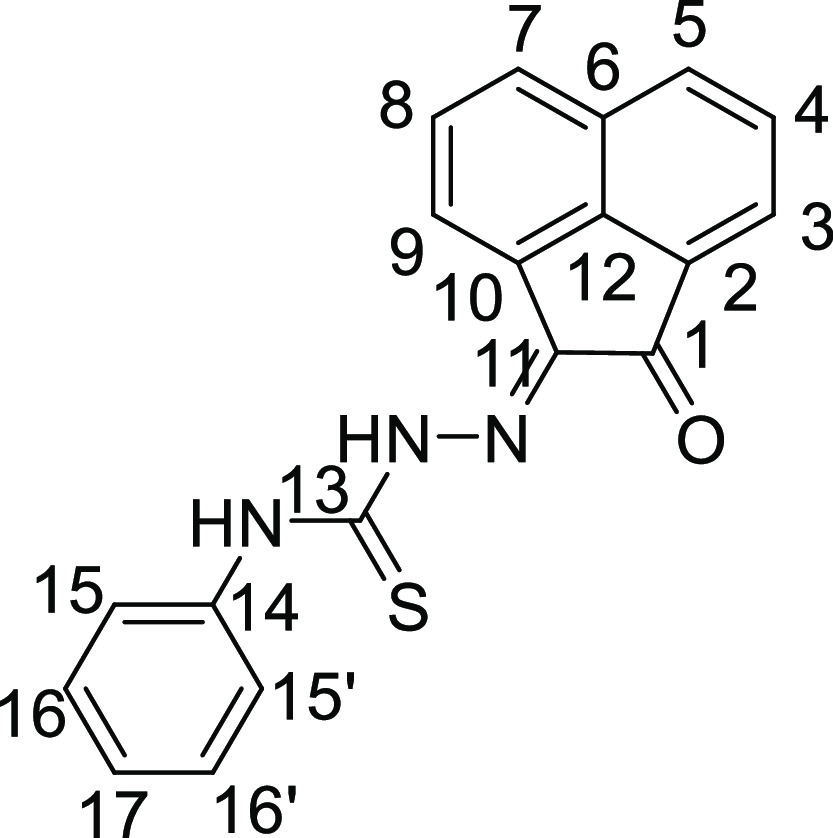



This microwave irradiation method was adapted from ref (20), and optimization protocols are given in the SI.

A microwave tube was filled with acenaphthenoquinone (0.2500 g, 1.37 mmol), 4-phenylthiosemicarbazid (0.2210 g, 1.37 mmol), 10 mL of EtOH, and two drops of concentrated HCl. The mixture was reacted at 90 °C in the microwave for 10 min, under stirring. The resulting mixture was then allowed to cool, filtrated, and washed with Et_2_O. The precipitate was collected to afford the desired compound (**4h**) as an orange solid (58%). No further purification was necessary. Crystals suitable for X-ray diffraction were obtained from DMSO after 2 days at room temperature.

^**1**^**H NMR** (500 MHz, DMSO-*d*_6_) δ_H_: 12.85 (s, 1H, N-N***H***); 11.01 (s, 1H, N***H***-Ph); 8.42 (d, 1H, ^3^*J* = 8.3 Hz, ***H***^**9**^); 8.16 (m, 3H, ***H***^**7**^, ***H***^**5**^, ***H***^**3**^); 7.89 (m, 2H, ***H***^**8**^, ***H***^**4**^), 7.65 (d, 2H, ^3^*J* = 7.7 Hz, ***H***^**15**^***H***^**15′**^), 7.47 (t, 2H, ^3^*J* = 7.6 Hz, ***H***^**16**^***H***^**16′**^), 7.31 (t, 1H, ^3^*J* = 7.6 Hz, ***H***^**17**^) ppm.

^**13**^**C NMR** (125 MHz, DMSO-*d*_6_) δ_C_: 188.6 (***C***=O); 176.6 (NHR-***C***S–NHPh); 139.4 (***C***=N-R); 138.5 (***C***^**14**^); 137.7 (***C***^**12**^); 132.8 (***C***^**9**^); 130.4 (***C***^**2**^); 129.9 (***C***^**6**^); 129.8 (***C***^**12**^); 128.9 (***C***^**4**^); 128.6 (***C***^**8**^); 128.4 (***C***^**16**^); 127.2 (***C***^**5**^); 126.2 (***C***^**17**^); 125.7 (***C***^**15**^); 122.5 (***C***^**7**^); 118.8 (***C***^**3**^) ppm.

**Mass spectrometry:** ASAP for C_19_H_13_N_3_OS, calcd for ([M – H]^+^) 330.0696 found 330.0699.

**IR** (solid): ν (cm^–1^) 3296, 3265, 3058, 1684, 1595, 1540, 1472, 1046, 1023 cm^–1^.

**HPLC** (Method C): *R*_f_ = 11.41 min.

##### Zn(II)[mono(F-benzyl thiosemicarbazonato)-acenaphthenequinone]_2_ (**4a-Zn**)

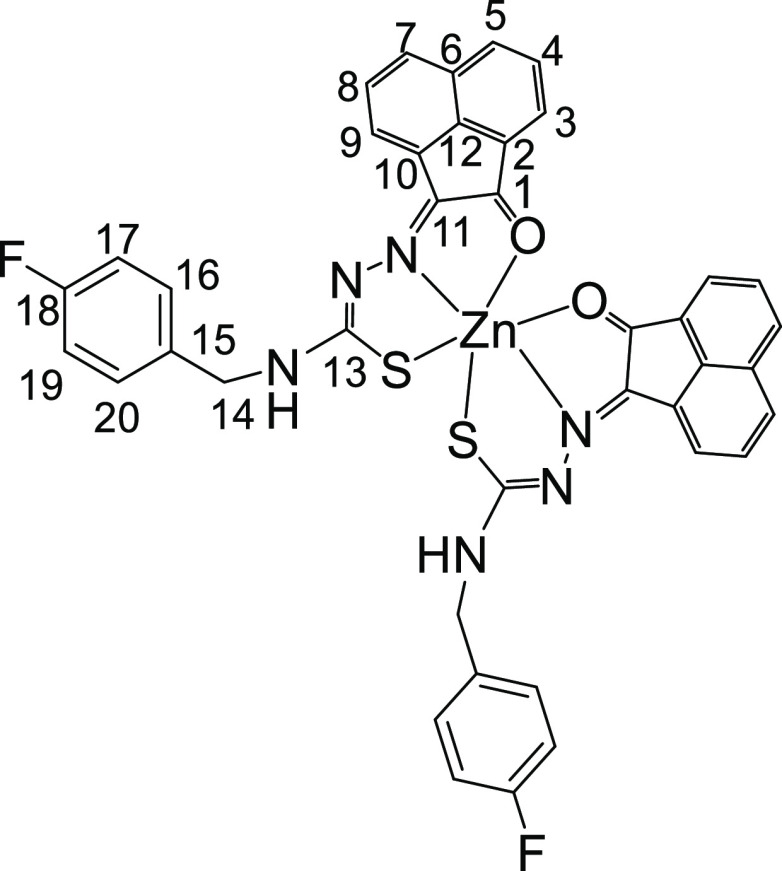



Mono(substituted) 4-F-benzyl-3-thiosemicarbazone-acenaphthenequinone (**4a**) (0.2000 g, 0.55 mmol) and anhydrous zinc acetate (0.2020 g, 1.10 mmol) were suspended in 5 mL of EtOH. The mixture was reacted at 90 °C under microwave irradiation for 60 min. The slurry was then filtrated and washed with Et_2_O. The precipitate was collected to afford ca. 0.96 g of the desired compound as an orange solid (88%). HPLC analysis using Methods A–D indicated that no further purification was necessary.

^**1**^**H NMR** (500 MHz, DMSO-*d*_6_) δ: 8.96 (d, 2H, ^3^*J* = 5.4 Hz, N***H***-bnzF); 8.56 (t, 2H, ^3^*J* = 7.5 Hz, ***H***^**9**^); 8.34 (appt, 2H, ^3^*J* = 7.9 Hz, ***H***^**3**^); 8.12 (t, 4H, ^3^*J* = 7.3 Hz, ***H***^**5**^***H***^**7**^); 7.89 (t, 4H, ^3^*J* = 6.6 Hz, ***H***^**4**^***H***^**8**^); 7.77 (m, 8H, ***H***^**16**^***H***^**17**^***H***^**19**^***H***^**20**^); 4.89 (t, 4H, ^3^*J* = 5.46 Hz, ***H***^**14**^) ppm.

^**13**^**C NMR** (125 MHz, DMSO) δ: 189.9 (***C***=O); 178.2 (NHR-***C***S–NHR) 163.3 (***C***^**18**^); 160.1 (***C***^**11**^); 139.6 (***C***^**12**^); 134.9 (***C***^**3**^); 133.1 (***C***^**6**^); 130.8 (***C***^**2**^); 130.3 (d, *J*_C–C_ = 8.4 Hz ***C***^**16**^); 129.8 (d, *J*_C–C_ = 8.4 Hz ***C***^**20**^); 129.7 (***C***^**4**^); 129.2 (***C***^**8**^); 127.6 (***C***^**5**^); 122.9 (***C***^**7**^); 128.9 (***C***^**10**^); 118.9 (***C***^**9**^); 115.3 (***C***^**17**^***C***^**19**^); 47.5 (***C***^**14**^) ppm.

^**19**^**F NMR** (470 MHz, DMSO-*d*_6_) δ_F_: −116.44 ppm.

**Mass spectrometry:** ASAP for C_40_H_26_F_2_N_6_O_2_S_2_Zn, calcd for ([M + H]^+^) 789.0896 found 789.0912.

**IR** (solid): ν (cm^–1^) 3243, 2936, 1538, 1449, 1392, 1081, 1022, 773 cm^–1^.

**HPLC** (Method C): *R*_f_ = 11.43, 14.47 min.

##### Zn(II)[mono(ethyl thiosemicarbazonato)-acenaphthenequinone]_2_ (**4f-Zn**)

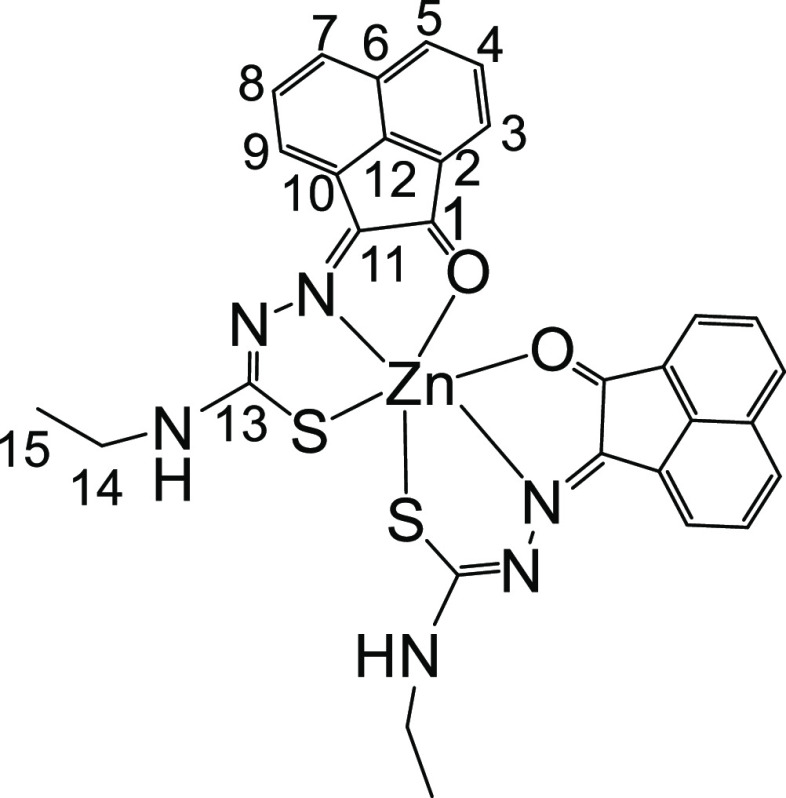



Mono(substituted) 3-ethyl-4-thiosemicarbazone-acenaphthenequinone (**4f**) (0.1844 g, 0.65 mmol) and anhydrous zinc acetate (0.1195 g, 0.65 mmol) were suspended in 10 mL of EtOH. The mixture was reacted at 90 °C under microwave irradiation for 60 min. The slurry was then filtrated and washed with Et_2_O. The precipitate was collected to afford 0.1508 g of the desired compound as an orange solid (37%). HPLC analysis using Methods A, B, and C, each indicating that no further purification was necessary.

^**1**^**H NMR** (500 MHz, DMSO-*d*_6_) δ_H_: 9.52 (q, 2H, ^3^*J* = 6.5, 8.5 Hz, N**H**-Allyl); 8.47 (overlapping d, 2H, ^3^*J* = 7.1 Hz, ***H***^**9**^); 8.32 (overlapping d, 2H, ^3^*J* = 8.4 Hz, ***H***^**5**^), 8.08 (overlapping d, 1H, ^3^*J* = 8.4 Hz, ***H***^**7**^), 7.84 (m, 4H, ***H***^**4**^, ***H***^**8**^), 3.76 (multiplet, 4H, 2H, ^3^*J* = 8.4 Hz, NH-C***H***_***2***_-CH_3_), 1.35 (t, 6H, ^3^*J* = 7.1 Hz, RCH_2_-C***H***_**3**_).

^**13**^**C NMR** (125 MHz, DMSO-*d*_6_) δ_C_: 188.91 (***C***=O); 177.21 (NHR-***C***S–NHEt); 139.44 (***C***^**12**^); 137.53 (***C***^**2**^); 133.2 (***C***^**7**^); 130.8 (***C***^**10**^); 130.5 (***C***=N-R); 130.3 (***C***^**6**^); 129.3 (***C***^**11**^); 127.9 (***C***^**3**^); 124.2 (***C***^**9**^); 123.4 (***C***^**4**^, ***C***^**8**^); 39.1 (R-**C**H_2_-*C*H_3_); 14.9 (RCH_2_-**C**H_3_) ppm.

**Mass spectrometry:** ESI (pos. mode) C_30_H_24_N_6_O_2_S_2_Zn, calcd for ([M + H]^+^) 629.0772 found 629.0765.

**IR** (solid): ν (cm^–1^) 3281, 2918, 1683, 1574, 1442,1394 1078, 1022 cm^–1^.

**HPLC** (Method C): *R*_f_ = 13.01, 13.55 min (or Method B: 21. 3 min).

##### Zn(II)[mono(allyl thiosemicarbazonato)-acenaphthenequinone]_2_ (**4g-Zn**)

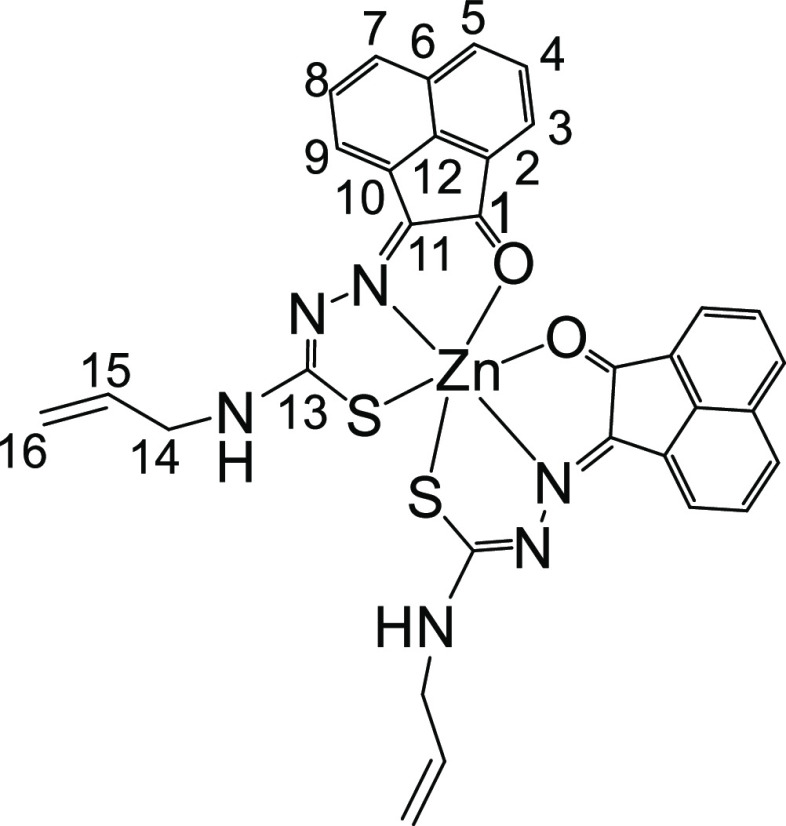



Mono(substituted) 3-allyl-4-thiosemicarbazone-acenaphthenequinone (**4g**) (0.1000 g, 0.34 mmol) and anhydrous zinc acetate (0.1242 g, 0.68 mmol) were suspended in 10 mL of EtOH. The mixture was reacted at 90 °C under microwave irradiation for 60 min. The slurry was then filtrated and washed with Et_2_O. The precipitate was collected to afford 0.1160 g of the desired compound as an orange solid (53%). HPLC analysis using Methods A–C indicated that no further purification was necessary.

^**1**^**H NMR** (500 MHz, DMSO-*d*_6_) δ_H_: 9.51 (t, 2H, ^3^*J* = 5.4 Hz, N***H***-Et); 8.56 (d, 2H, ^3^*J* = 6.8 Hz, ***H***^**3**^); 8.47 (d, ^3^*J* = 6.8 Hz, 2H, ***H***^**9**^); 8.34 (m, 4H, ***H***^**7**^***H***^**5**^), 7.83 (m, 4H, ***H***^**4**^, ***H***^**8**^), 6.0 (m, 4H, NH-C***H***_**2**_-CHCH_2_), 5.17 (overlapping dd, 4H, ^3^*J* = 17.2, 17.5 Hz, RCH-C***H***_**2**_); 4.28 (multiplet, 2H, ^3^*J* = 5.6 Hz, NHC*H*_**2**_-**C**H-CH_2_) ppm.

^**13**^**C NMR** (125 MHz, DMSO-*d*_6_) δ_C_: 188.6 (***C***=O); 177.6 (NHR-***C***S–NHAllyl); 139.2 (***C***^**12**^); 137.6 (***C***^**2**^); 134.6 (R-***C***H-CH_2_); 133.2 (***C***^**7**^); 130.46 (***C***^**10**^); 130.06 (***C***=N-R); 129.9 (***C***^**6**^); 128.6 (***C***^**11**^); 127.9 (***C***^**4**^); 127.2 (***C***^**8**^); 124.3 (***C***^**9**^); 124.1 (***C***^**5**^); 123.8 (***C***^**3**^); 116.7 (RCH-***C***H_2_); 46.1 (NH-***C***H_2_-R) ppm.

**Mass spectrometry:** ASAP for C_32_H_24_N_6_O_2_S_2_Zn, calcd for ([M + H]^+^) 653.0772 found 653.0764.

**IR** (solid): ν (cm^–1^) 3295, 3055, 1685, 1594, 1536, 1048, 1023 cm^–1^.

**HPLC** (Method C): *R*_f_ = 13.11 min.

##### Zn(II)[Mono(phenyl thiosemicarbazonato)-acenaphthenequinone]_2_ (**4h-Zn**)

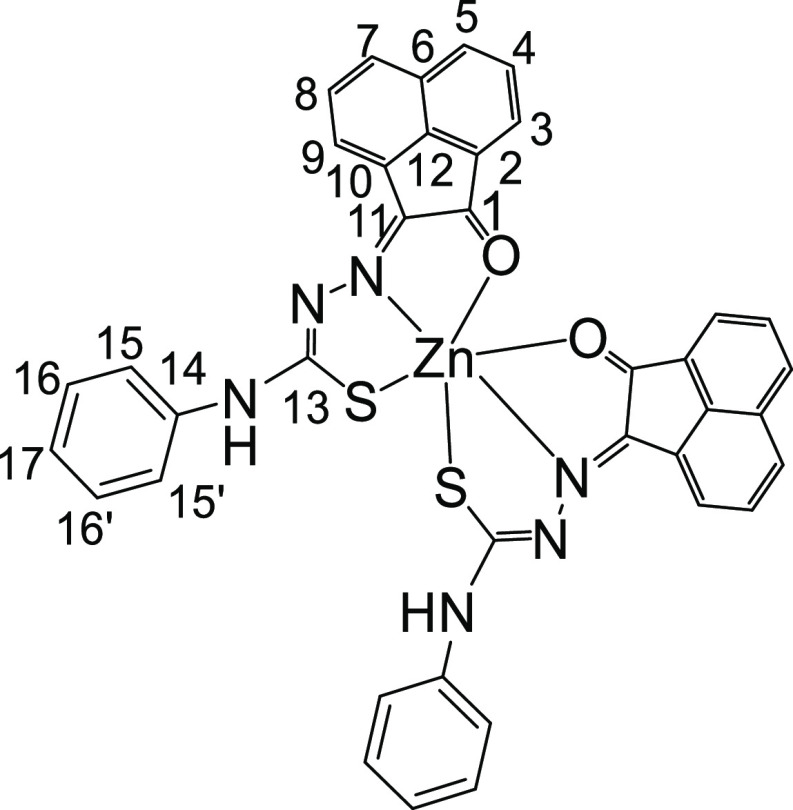



Mono(substituted) 3-phenyl-4-thiosemicarbazone-acenaphthenequinone (**4h**) (0.5000 g, 2.74 mmol) and anhydrous zinc acetate (0.5450 g, 2.74 mmol) were suspended in 5 mL of EtOH. The mixture was reacted at 90 °C under microwave irradiation for 60 min. The slurry was then filtrated and washed with Et_2_O. The precipitate was collected to afford 0.9564 g of the desired compound as a yellow solid (88%). HPLC analysis using Methods A–C indicated that no further purification was necessary.

^**1**^**H NMR** (500 MHz, DMSO-*d*_6_) δ_H_: 10.9 (s, 2H, N***H***-Ph); 8.37 (d, 2H, ^3^*J* = 8.1 Hz, ***H***^**9**^); 8.19 (d, 4H, ^3^*J* = 8.2 Hz, ***H***^**5**^***H***^**7**^); 8.19 (d, 2H, ^3^*J* = 8.1 Hz, ***H***^**3**^); 7.83 (d, 4H, ^3^*J* = 8.1 Hz, ***H***^**8**^, ***H***^**4**^), 7.78 (overlapping d, 2H, ^3^*J* = 7.0 Hz, ***H***^**15**^***H***^**15′**^), 7.47 (t, 2H, ^3^*J* = 6.6 Hz, ***H***^**16**^***H***^**16′**^), 7.31 (t, 1H, ^3^*J* = 6.0 Hz, ***H***^**17**^) ppm.

^**13**^**C NMR** (125 MHz, DMSO-*d*_6_) δ_C_: 189.9 (***C***=O); 177.1 (NHR-***C***S–NHPh); 139.9 (***C***=N-R); 138.2 (***C***^**12**^); 137.4 (***C***^**2**^); 133.6 (***C***^**9**^); 130.9 (***C***^**14**^); 130.4 (***C***^**3**^); 130.4 (***C***^**6**^); 129.4 (***C***^**8**^); 129.1 (***C***^**4**^); 128.9 (***C***^**16**^); 127.7 (***C***^**3**^); 126.6 (***C***^**17**^); 126.2 (***C***^**15**^); 123.0 (***C***^**7**^); 119.3 (***C***^**5**^).

**Mass spectrometry:** ASAP for C_19_H_13_N_3_OS, calcd for ([M – H]^+^) 330.0696 found 330.0699.

**IR** (solid): ν (cm^–1^) 3294, 1684, 1593, 1540, 1376, 1042, 1025 cm^–1^.

**HPLC** (Method C): *R*_f_ = 11.55 min.

### Radiolabeling Assays

#### Treatment of Monosubstituted Thiosemicarbazones with Aqueous [^68^Ga]Ga(III)

A SnO_2_-based column matrix ^68^Ge/^68^Ga generator was used to elute 10 mL of 0.6 M HCl, ca. 178 MBq (4.81mCi) of gallium-68, which was trapped on a Strata x-c 33 μm Polymeric Strong Cation Cartridge from Phenomenex and eluted with 700 μL of 0.02 M HCl (98% THF). This was subsequently dried for 7–10 min under a nitrogen stream at 95 °C. Next, 30 μL of the monosubstituted compound in dry DMSO (2 mg/mL) was added along with 0.6 mL of injectable MeOH. This was heated under microwave radiation at 95 °C for 30 min. Analysis by reversed-phase HPLC (Method C) gave two different retention times for each compound, which in comparison with the HPLC trace of the precursors suggests the presence of isomerism or mixtures of products. Remaining traces of [^68^Ga]Ga ions were observed, indicating that radiolabeling of the mono(substituted) ligands had not gone to completion or that the decomposition of the desired product under the radiolabeling and purification conditions occurs.

#### Treatment of Compound 4c* with [^18^F]FBA

In a sealed reaction vial, compound 4c* (1.20 mg, 0.0042 mmol) was diluted in 0.5 mL of DMF and mixed with 25 μL of the SPE purified solution of compound [^18^F]-FBA in MeCN (5.44 MBq, 147 μCi). The slurry was heated to 120 °C for 25 min. Analysis by reversed-phase HPLC (Method D) gave a retention time of 7.08 min which in comparison with the reference HPLC trace suggested the F-18 incorporation. The extent of conversion to the product was measured as 30%.

### Crystal Structure Determination by X-ray Diffraction and Computational Chemistry Details

Crystallization of compounds to give rise to single crystals suitable for analysis by X-ray diffraction was pursued using several different methods, as follows. The first method involved dissolving the compound of interest in the minimum of THF in a small glass vial and placing this inside a larger vial. A small amount of pentane was placed in the larger vial, and the system was sealed from the outside atmosphere. This was then kept in a still place, allowing the crystals to grow slowly over the subsequent weeks. In the alternative method, the compound of choice was dissolved in the minimum of THF in a vial, and the pentane was layered on top (THF:pentane ratio, 1:2). Additionally, crystals suitable for X-ray diffraction were allowed to grow slowly over several weeks from concentrated solutions of DMSO or *d*_6_-DMSO in NMR tubes. Crystals were selected using the oil drop technique, in perfluoropolyether oil and mounted at 150(2) K with an Oxford Cryostream N_2_ open-flow cooling device.

Intensity data were collected on a Nonius Kappa CCD single-crystal diffractometer using graphite monochromated Mo Kα radiation (λ = 0.71073 Å), whereby data were processed using the Nonius Software, or at Diamond using Synchrotron radiation (λ = 0.68890 Å) on a CrystalLogic Kappa (3 circle), Rigaku Saturn724 at 150 K, whereby data were processed using the Rikagu software package (CrystalClear-SM Expert 2.0 r5). Alternative data collection was at 150(2) K on a Rigaku Xcalibur, EosS2 single-crystal diffractometer using graphite monochromated Mo Kα radiation (λ = 0.71073 Å) on a Rigaku SuperNova Dual EosS2 single-crystal diffractometer using monochromated Cu Kα radiation (λ = 1.54184 Å), in which case the unit cell determination, data collection, data reduction, and absorption correction were performed using the CrysAlisPro software. For all structures, a symmetry-related (multiscan) absorption correction had been applied. The structures were solved by direct methods using the programs SIR97 or SHELXS-97 followed by full-matrix least-squares refinement on F^2^ using SHELXL-97 implemented in the WINGX-1.80 suite of programs throughout. Additional programs used for analyzing and graphically handling data included SHELXle, SHELXL-2018/3, PLATON, and ORTEP3 for Windows and Mercury.^45−56^

Hydrogen atoms were placed onto calculated positions and isotropically refined using a riding model. All nonhydrogen atoms were refined anisotropically. Where possible, heteroatom-bound hydrogen atoms have been located in the difference Fourier map and were refined freely or with bond length restraints.

Crystallography data were deposited to CCDC, and selected information is given in the SI and uploaded as CIF files. Deposition numbers are: 2130526 (precursor), 2130525 (**2a**), 2130524, 2149602 (two different polymorphs of **3a**), 2130523 (**4a**), 2130521 (**4c**), 2130516 (**4b**), 2130510 (**4h**-DMSO adduct), 2130508 (**4h**), 2130507 (**4g**), 2131107 (**4f**), 2130502 (**4f-Zn**, Oh), and 2130501 (**4f-Zn**, Td).

Density functional theory (DFT) calculations were performed using the Amsterdam Density Functional (ADF) suite.^57−59^ All calculations were performed in the gas phase. The generalized gradient approximation (GGA) functional BLYP was employed along with the TZ2P basis set. Geometries were optimized and analytical frequencies calculated. Numerical quality was set to “good”. No frozen cores were applied.

## Supporting Information Available

DFT optimization data (ZIP)
Crystallography data (ZIP)
General experiment, alternative synthesis methods and optimization protocols, detailed NMR and mass spectrometry, HLPC traces, UV–vis, radiochemistry, cellular assays, and DFT optimization results (PDF)

## Notes

Data access statement: The raw data that support the findings of this study are available upon request from the corresponding author.
