# Supplementary figures and images for: Functional, Aromatic, and Fluorinated Monothiosemicarbazones: Investigations into Their Structures and Activity toward the Gallium-68 Incorporation by Microwave Irradiation
Source: ACS Omega. 2022 Apr 11;7(16):13750–77. doi: 10.1021/acsomega.1c07396 (PMC9088960; doi:10.1021/acsomega.1c07396)

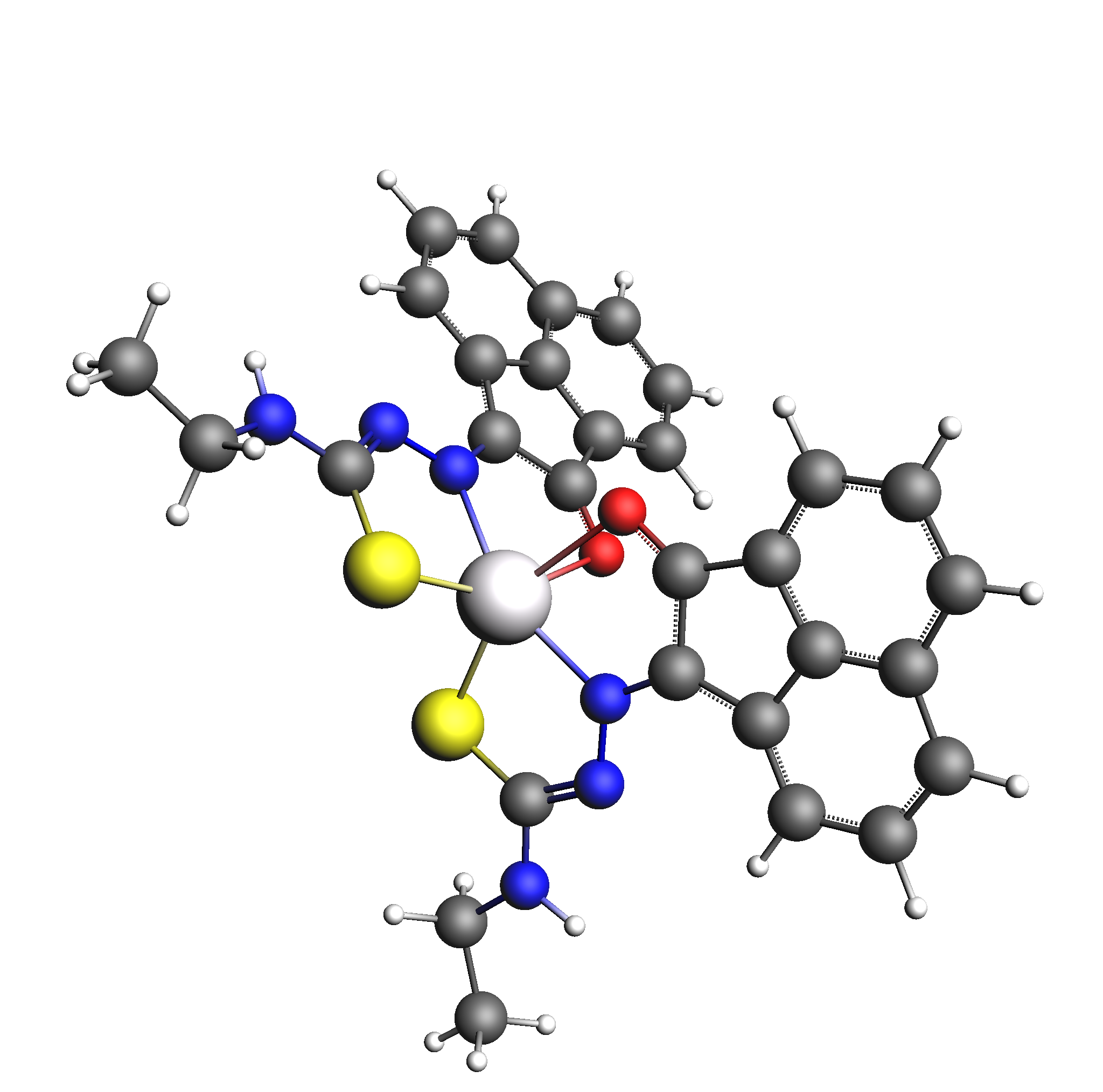

Supplement: Supplementary file 1 — ao1c07396_si_001.zip [file ao1c07396_si_001.zip › DFT_files/Mono TSCEt Zn complex isomer Orientation 2.png]

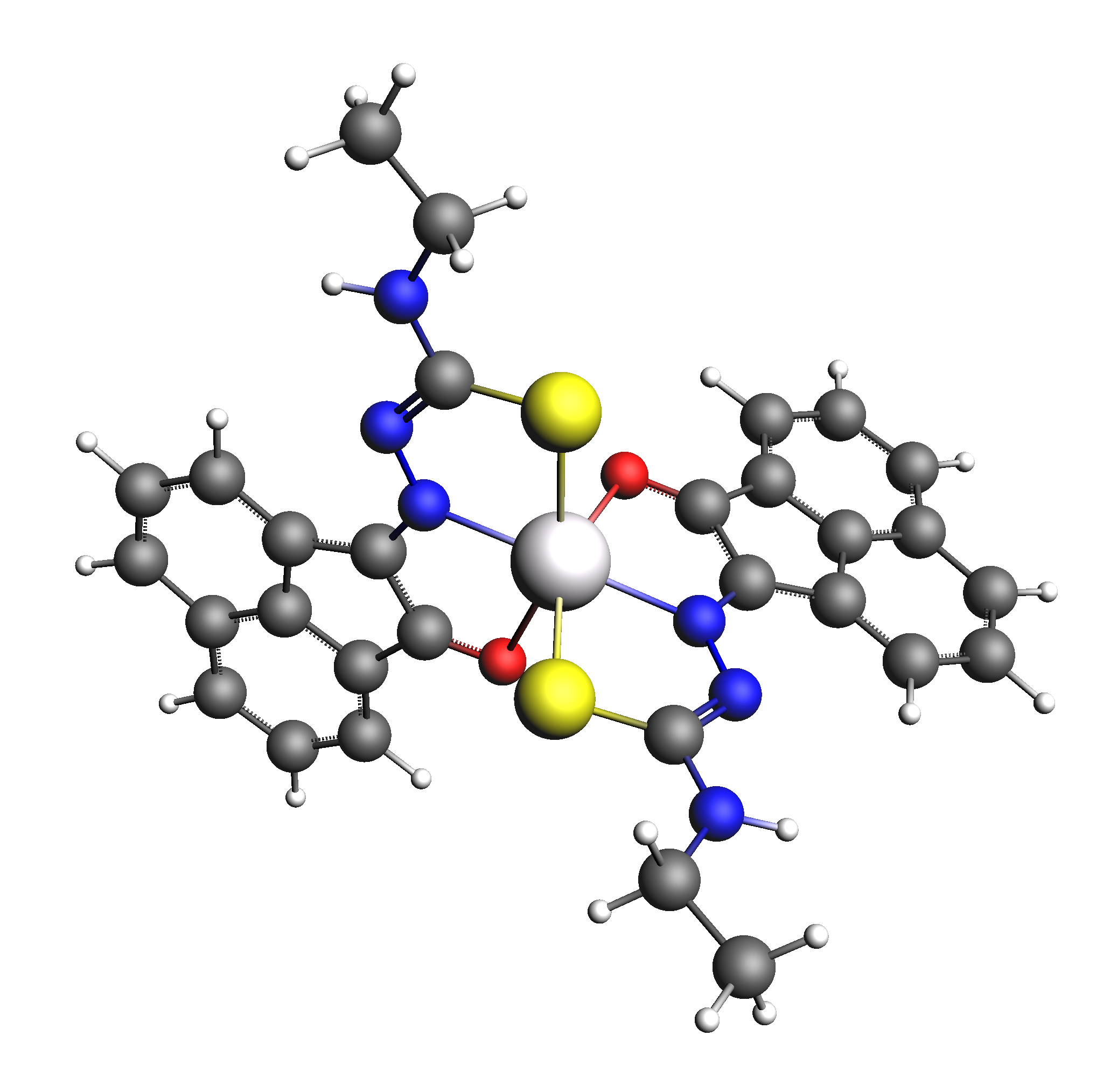

Supplement: Supplementary file 1 — ao1c07396_si_001.zip [file ao1c07396_si_001.zip › DFT_files/Mono TSCEt Zn complex isomer.png]

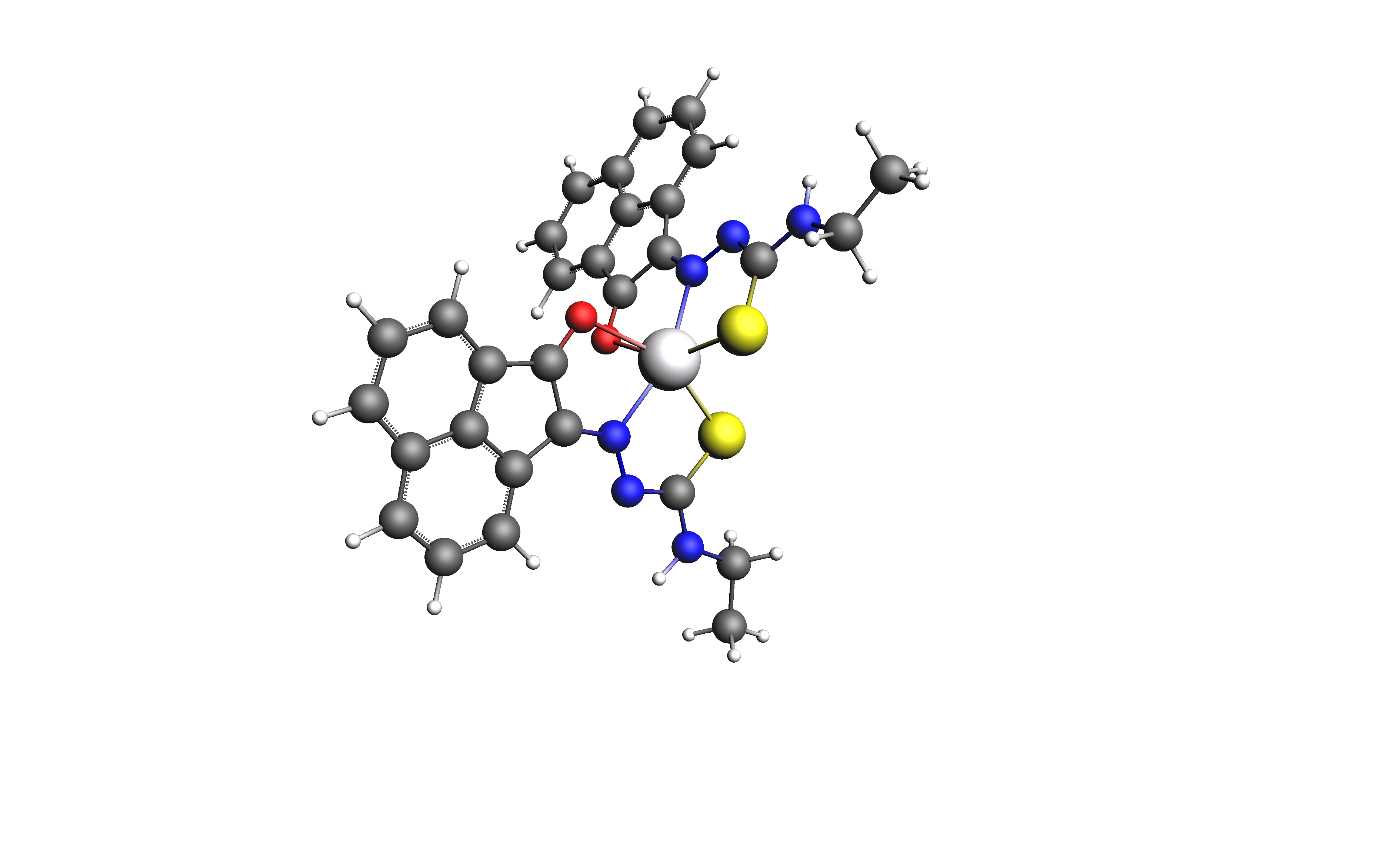

Supplement: Supplementary file 1 — ao1c07396_si_001.zip [file ao1c07396_si_001.zip › DFT_files/Mono TSCEt Zn complex Opt orientation 2.png]

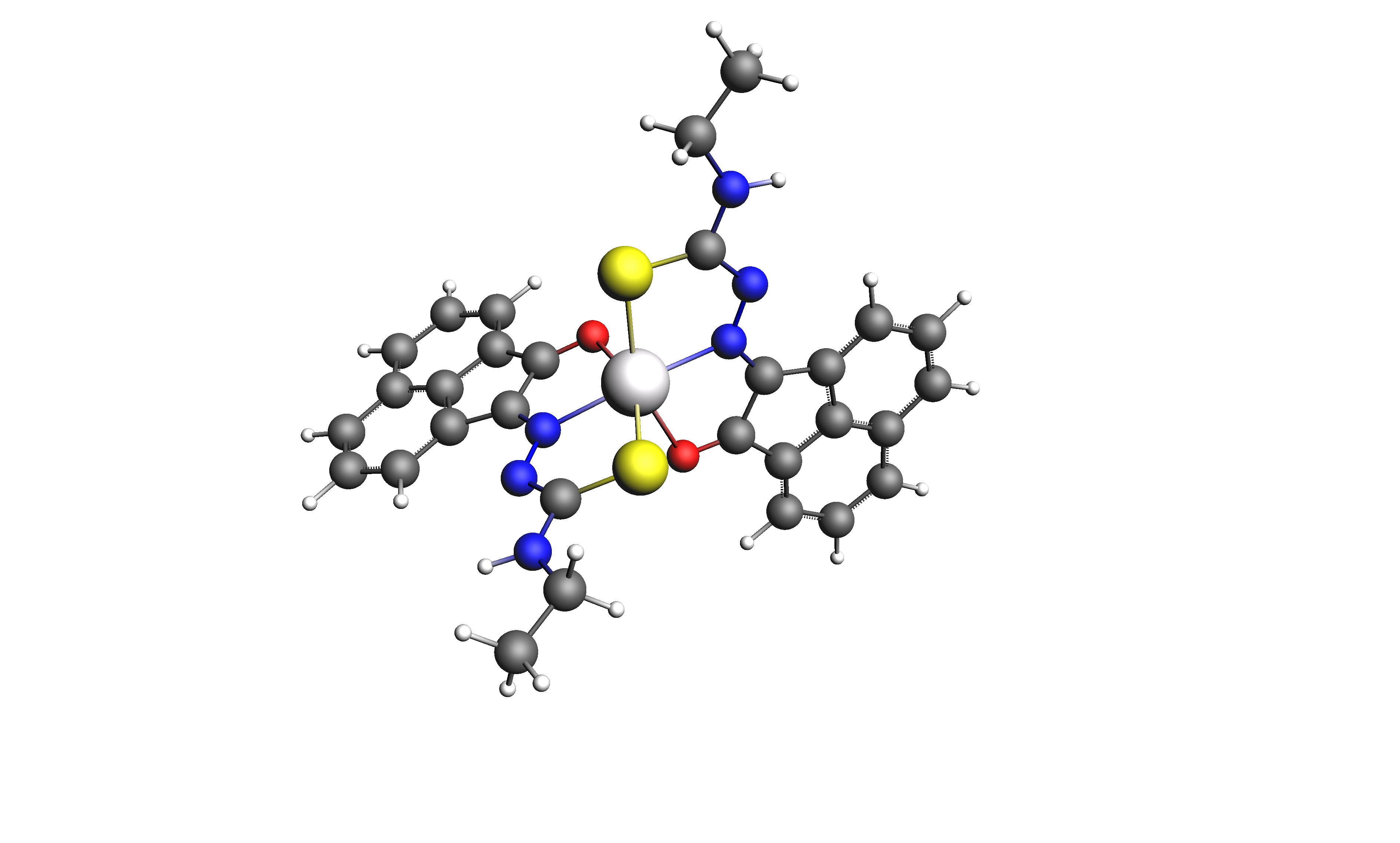

Supplement: Supplementary file 1 — ao1c07396_si_001.zip [file ao1c07396_si_001.zip › DFT_files/Mono TSCEt Zn complex.png]
