# Supplementary material for: Functional, Aromatic, and Fluorinated Monothiosemicarbazones: Investigations into Their Structures and Activity toward the Gallium-68 Incorporation by Microwave Irradiation
Source: ACS Omega. 2022 Apr 11;7(16):13750–77. doi: 10.1021/acsomega.1c07396 (PMC9088960; doi:10.1021/acsomega.1c07396)
Supplement: Supplementary file 3 — ao1c07396_si_016.pdf [file ao1c07396_si_016.pdf]

Supporting Information

Functional, Aromatic and Fluorinated Monothiosemicarbazones: Investigations into their Structures and Activity towards the Gallium-68 incorporation by Microwave Irradiation

Dr Sophia Sarpaki[1= &], Dr Fernando Cortezon-Tamarit[1= $], Rüdiger Maria Exner[1=], Kexin Song[1=], Dr Sara Raquel Mota Merelo de Aguiar[1], Dr Haobo Ge[1], Dr Charareh Pourzand[2,3], Dr Stephen James Paisey[4], Dr Gabriele Kociok-Köhn[1], Prof Jonathan Robin Dilworth[1,5], Dr Laurence Carroll [6,7]* and Prof Sofia Ioana Pascu[1,3]*

[1] Department of Chemistry, University of Bath, Claverton Down, Bath, BA2 7AY, United Kingdom
[2] Department of Pharmacy and Pharmacology, University of Bath, Claverton Down, Bath, BA2 7AY, United Kingdom
[3] Centre of Therapeutic Innovations, University of Bath, Claverton Down, Bath, BA2 7AY, United Kingdom
[4] Wales Research and Diagnostic PET Imaging Centre, School of Medicine, University of Cardiff, Cardiff, UK
[5] Department of Chemistry, Chemistry Research Laboratory, University of Oxford, Oxford, OX2 3TA, United Kingdom
[6] Department of Medicine, Imperial College, Du Cane Road, W12 0NN London, United Kingdom
[7] Russell H. Morgan Department of Radiology and Radiological Sciences, Johns Hopkins Medical Institutions, Baltimore, Maryland, USA

[=] *These authors contributed equally to the work*

Current Address: [$]Oxford University, Radiobiology Research Institute, Oxford, UK OX3 7LE;
[&] *BIOEMTECH Alexandras Avenue 116, Athens, GR 11472; 6987779417*

Keywords: Monothiosemicarbazones, Gallium-68, Fluorine-18, Radiochemistry, Zn(II) complexes, synchrotron X-ray diffraction

Content:

1.General experimental methods
2.Synthetic procedures with alternative routes and selected analytical data
3.Optical spectroscopy of selected monothiosemicarbazones and kinetic stability tests
4.Cellular imaging and viability assays
5.Radiochemistry assays
6.Cellular uptake of the Gallium-68 radiolabeled compound **4a**
7. Experiments for the formation of 'cold' Gallium (III) complexes
8.Additional mass spectrometry data for key compounds and intermediates
9.Infrared spectroscopy for representative compounds
10.Selected X-Ray Crystallographic Data
11.X-ray diffraction data for **2a**, **3a**, **4a**, **4b and 4c**
12. CCDC Deposition Information
13.Estimation of logP
14.Gas Phase DFT Calculations

1. General experimental methods

All chemicals and solvents were reagent grade and used as received unless otherwise specified. Microwave reactions were performed in a Biotage Initiator 2.5 system in 20 mL glass capped vials. The reaction mixture was pre-stirred for 30 s and then heated for the selected time. If the irradiation power is not set, it reaches its maximum (300 W from magnetron at 2.45 GHz) at the start of the reaction until the target temperature is reached, decreasing to lower values afterwards.

The deuterated solvents were purchased from Aldrich and dried over 4 Å molecular sieves. ^{1}H, $^{13}C\{^{1}H\}$, and $^{19}F\{^{1}H\}$ NMR spectra were recorded on a Bruker Avance spectrometer (300 MHz, 400 MHz) or a Bruker Avance II+ (500 MHz) spectrometer at 298 K. ^{1}H and $^{13}C\{^{1}H\}$ NMR spectra were referenced internally to residual protio-solvent, and solvent resonances, respectively, and are reported relative to tetramethylsilane (δ = 0 ppm). $^{19}F\{^{1}H\}$ NMR spectra were referenced to $CFCl_3$ (-120.4 ppm).

Mass spectrometry was performed in a Bruker Micromass LCT TOF spectrometer under conditions of electrospray ionization. Accurate masses are reported to the fourth decimal place using tetraoctylammonium bromide (466.5352 Da) as an internal standard.

HPLC was carried out using a Agilent 1100 series HPLC system (Agilent Technologies, Stockport, UK), equipped with a UV detector (254 nm) and a Lab-Logic Flow-count radio-detector, using a Phenomenex Gemini C_{18} or a Waters Symmetry C_{18} column (250 mm × 4.6 mm, 110 Å or 100 Å, respectively) and Laura 3 software (LabLogic, Sheffield, UK) at a flow rate of 1mL/min. The gradient elution was 0.1% TFA in milli-Q water as solvent A and 0.1% TFA in MeCN as solvent B. A reverse gradient was applied starting with A at 95% for 2 minutes, going up to 5% A at 12 minutes, then an isocratic step until 14 minutes and gradient until 95% A at 16 minutes, then hold to 25 minutes (Method A; variations of this methods were also applied, as specified).

The IR spectra were recorded on a Perkin Elmer (Waltham, Massachusetts) Frontier FTIR spectrometer, in the range between 650 and 4000 cm^{-1} with a resolution of 4 cm^{-1}.
UV-visible spectra were obtained using a Lamda 650 PerkinElmer Spectrometer and processed using UV Winlab 3 software. Samples were dissolved in DMSO and diluted to the desired concentration, prior to measurement in quartz cuvettes with a path-length of 1.00 cm.

Fluorescence spectra were measured in a LS55 Perkin-Elmer luminescence spectrophotometer using a 1.00 cm quartz cuvette. A scan from 250–750 nm with increments of 50 nm was initially carried out to determine excitation wavelength of maximum emission (λex-max).

Confocal microscopy images were acquired in a Nikon Eclipse Ti-E instrument equipped with 405 nm, 488 nm and 561 nm excitation lasers. The images obtained were processed using the Nikon NIS elements-AR Analysis 4.30.02 software. Further confocal microscopy images were acquired in a Zeiss (Oberkochen, Germany) LSM 880 instrument equipped with 405 nm / 488 nm / 594 nm excitation lasers. The images obtained were processed using the ZEN software platform. The working distance of a 60x objective lens used was 0.17 mm (40x = 0.2 mm); therefore, cells were seeded onto single well plates where the coverslip was the bottom of the well.

1.1. Cell culturing and cell plate preparation

Cells were cultured at 37 °C in a humidified atmosphere in air and harvested once >70% confluence had been reached. Both PC3 (prostate cancer cells) and EMT6 (breast cancer cells) were cultured in RPMI 1640 serum medium. The media contained 10% fetal calf serum (FCS), 0.5% penicillin/streptomycin (10,000 IU mL-1/10,000 mg mL-1) and 1% 200 mM L-Glutamine. All steps were performed in the absence of phenol red. Supernatant containing dead cell matter and excess protein was aspirated. The live adherent cells were then washed with 10 mL of phosphate buffer saline (PBS) solution twice to remove any remaining media containing FCS. Cells were incubated in 3 mL of trypsin solution (0.25% trypsin) for 5 to 7 minutes at 37 °C. After trypsinisation, 6 mL of medium containing 10% serum was added to inactivate the trypsin and the solution was centrifuged for 5

minutes (1000 rpm, 25 ºC) to precipitate cells. The supernatant was aspirated and 5 mL of serum medium (10% FCS) was added to the cell matter left behind. Cells were counted using a haemocytometer and then seeded in glass bottom dishes or 96 well plates as appropriate.

1.2. Radiochemistry methods

Radio-HPLC was performed on an Agilent 1100 series HPLC system (Agilent Technologies, Stockport, UK) equipped with a γ-RAM Model 3 gamma-detector (IN/US Systems Inc, Florida, USA) and Laura 3 software (LabLogic, Sheffield, UK). The gradient elution was 0.1% TFA in milli-Q water as solvent A and 0.1% TFA in MeCN as solvent B. A reverse gradient was applied starting with A at 95% for 2 minutes, going up to 5% A at 12 minutes, isocratic level until 14 minutes and gradient until 95% A at 16 minutes, then hold to 25 minutes (**Method B**).

Radio-TLC was performed on a LabLogic PET/SPECT radio-TLC Scanner system (LabLogic, Sheffield, UK) and a Laura software (LabLogic, Sheffield, UK). The radio-TLC was developed on Whatman 3MM with 0.35 M ethylenediaminetetraacetic acid (EDTA) as the mobile phase.

The positron emitting radiotracer gallium-68 was extracted either from a SnO_2-based column matrix $^{68}Ge/^{68}Ga$ generator (Department of Surgery and Cancer of Imperial College in London) using a 0.6 M HCl solution or produced through a cyclotron (PETIC, Cardiff, UK) using a 0.1 M HCl solution. In optimised protocols when $^{68}Ga^{+3}$ was produced through the generator, this was trapped in a strata x-x 33 μμ Polymeric Strong Cation Catridge from Phenomenex and eluted from there using an acidic solution of THF (generally THF/HCl 0.02M 98%). Specifically, the eluted $^{68}Ga(III)$ was purified as follows: the activity was trapped in a SXC cartridge, which was already activated with 1 mL of HCl solution 0.1 M and washed with 10 mL of water. Then the $[^{68}Ga]GaCl_3$ was eluted from the cartridge with 0.8 mL of a THF/HCl (0.02 M) solution (98%) or acetone/HCl (0.02 M) solution (98%) and the resulting $^{68}Ga^{+3}$ stock was then subjected to a flow of nitrogen for 7-10 min before use.
The as-extracted $^{68}Ga^{+3}$ stock measured ca. 148 - 165 MBq. All stock solution of the ligands were prepared using 2 mg of compound per 1 mL of DMSO. For each radioreaction, 25 microL ligand in DMSO was diluted with
500 μL of MeOH in 0.5-2 ml μW vials. To this, 250 μL of $^{68}Ga^{+3}$ stock solution (measuring ca. 7-8 MBq per reaction) was addend and the mixture was pH adjusted (to pH 4-6). The radio-syntheses were carried out both under microwave radiation and conventional heating, as described in the main manuscript and given in Section 5. The reaction progress was monitored by radioHPLC whereby 20 microL aliquots were injected and the general HPLC conditions used were as stated above (e.g. reverse phase column c18 gradient run 95% water (0.1% TFA) and 5% acetonitrile (0.1% TFA)). Generally, it was consistently observed that the microwave reactions resulted in higher ROI w.r.t conventional heating protocols.

The positron emitting radiotracer $[^{18}F]$fluoride was produced through a cyclotron (^{18}O (p,n) ^{18}F, Imanova, London, UK). Synthesis of $[^{18}F]$4-fluorobenzaldehyde was performed, on the FASTlabTM *via* an automated procedure. The $[^{18}F]$fluoride was first dried and then was trapped on a Sep-Pak QMA-carbonate Light Cartridge. Then it was eluted into the reactor using an eluent consisting of Kryptofix K222 and $KHCO_3$ in acetonitrile: water (4:1). The content of the reactor was evaporated at 120 ºC under reduced pressure followed by drying under a low flow of nitrogen. The anhydrous fluoride was then dissolved with 600μl of anhydrous acetonitrile before being transferred to a Wheaton vial. The fluorination step was then performed manually. 400 μL of the anhydrous fluoride was added by syringe to a v-bottom vial containing 3 mg of the precursor. The vial was then heated at 90 °C for 15 minutes resulting in consistently >98% radiochemical purity according to radio-HPLC, and it was then used to further labelling reactions.

Microwave reactions were conducted in a Biotage (Uppsala, Sweden) Initiator 2.5 reactor (0-450 depending on T) in stirred capped vials. The reaction mixtures were pre-stirred for 30 s and heated to the desired temperature by applying maximum power of 400 W that was reduced and kept constant once the target temperature was reached. All the radiolabelling experiments stated were repeated minimum twice.

2. Synthetic procedures with alternative routes and selected analytical data

2.1. General synthetic procedure for the synthesis of the thiosemicarbazides ligands

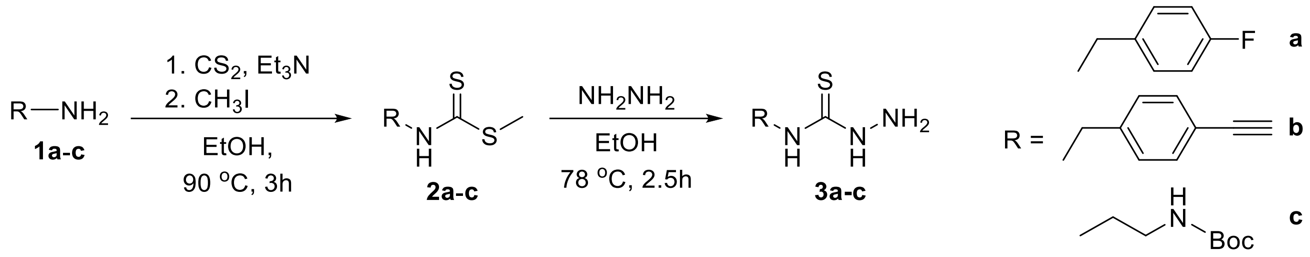

Scheme S1 Synthetic pathway for the preparation of different thiosemicarbazides.

Part A: Carbon disulfide (52.56 mmol) was added dropwise to a solution of triethylamine (52.56 mmol) and the correspondent amine (4-fluorobenzylamine (**1a**), (4-ethynylphenyl)methanamine (**1b**) or N-Boc-ethylenediamine (**1c**) (43.75 mmol) in EtOH (60 mL) under stirring. The mixture was allowed to react for 1.5 h at 25 °C, and then iodomethane (52.56 mmol) was added into the mixture and stirred for another 1.45 h. Afterwards, the solvent was removed under *vacuum*, and the product was dissolved in EtOAc (50 mL), and 1M HCl (100 mL), saturated $NaHCO_3$ solution (200 mL) and distilled H_2O (300 mL) were added to the solution. The organic phase was dried over magnesium sulfate anhydrous and the excess of solvent was removed under reduced pressure to afford the desirable product.

Methyl (4-fluorobenzyl)carbamodithioate (2a):

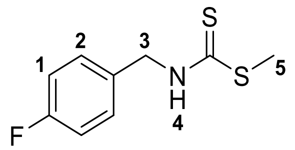

The product was obtained as a yellowish powder in 67 % yield. **^{1}H NMR** (500 MHz, DMSO-d_6, 25 ºC): 10.40 (s, 1H, H4) 7.35-7.27 (m, 2H, H2/2'), 7.15 (appt, 2H, ^3J= 8.9 Hz, H1/1'), 4.79 (d, 2H, ^3J= 3.3 Hz, H3), 2.51 (s, 3H, H5). **^{13}C{^{1}H} NMR** (75 MHz, DMSO-d_6, 25 ºC): 198.3, 161.7 (d, $J_{C\text{-}F}$ = 242.8 Hz), 134.0 (d, $J_{C\text{-}F}$ = 3.1 Hz), 130.0 (d, $J_{C\text{-}F}$ = 8.2 Hz), 115.5 (d, $J_{C\text{-}F}$ = 21.3 Hz), 49.1, 17.8. **^{19}F{^{1}H} NMR** (500 MHz, DMSO-d_6, 25 ºC): -118.05. **Mass spectrum**: ESI-MS calc. for $C_9H_{10}FNS_2$ $[M+H]^+$: 216.0311 found 216.0312.

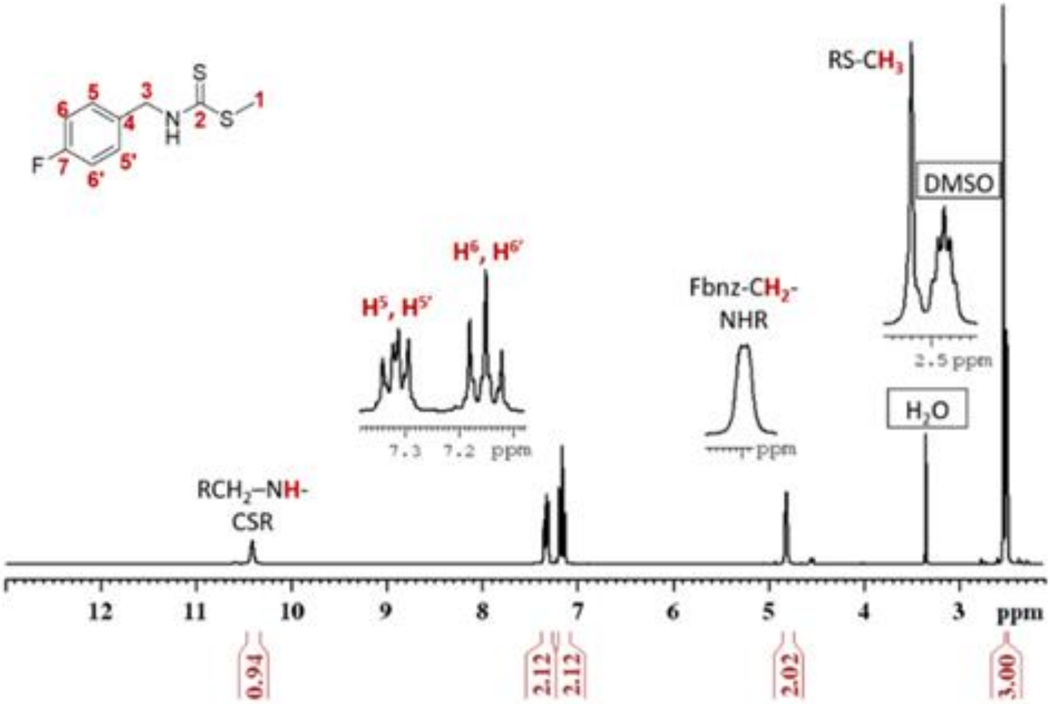

Figure S1 ^{1}H NMR of compound **2a** (500 MHz, 298 K, DMSO-d_6)

Methyl (4-ethynylbenzyl)carbamodithioate (2b):

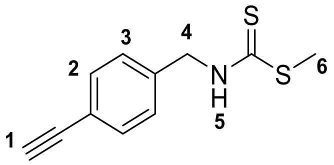

The product was obtained as a yellowish powder in 73 % yield. **^{1}H NMR** (300 MHz, DMSO-d$_6$, 25 ºC): 10.42 (s, 1H, H5) 7.43 (d, 2H, ^3J = 8.2 Hz, H3/3'), 7.26 (d, 2H, ^3J = 8.4 Hz, H2/2'), 4.84 (d, 2H, ^3J= 4.8 Hz, H4), 4.16 (s, 1H, H1), 2.53 (s, 3H, H6). **^{13}C{^{1}H} NMR** (125 MHz, DMSO-d$_6$, 25 ºC): 198.3, 137.8, 133.2, 127.6, 121.1, 85.4, 80.2, 52.0, 18.1. **Mass spectrum**: ESI-MS calc. for $C_{11}H_{11}NS_2$ $[M+H]^+$: 222.0406 found 222.040.

Methyl-N-(2-tert-butoxycarbonylaminoethyl)dithiocarbamate (2c):

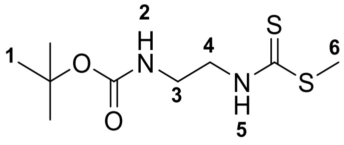

The product was obtained as a yellowish powder in 81 % yield. **^{1}H NMR** (300 MHz, CDCl$_3$, 25 ºC): 8.43 (s, 1H, H5), 5.16 (s, 1H, H2), 3.76 (d, ^3J = 5.0 Hz, 2H, H4), 3.39 (d, ^3J = 5.0 Hz, 2H, H3), 2.55 (s, 3H, H6), 1.41 (s, 9H, H1). **^{13}C{^{1}H} NMR** (75 MHz, CDCl$_3$, 25 ºC): 199.2, 157.8, 80.4, 49.4, 39.1, 28.4, 18.0. **Mass spectrum**: ESI-MS calc. for $C_9H_{19}N_2O_2S_2$ $[M+H]^+$: 251.0888; found: 251.0875.

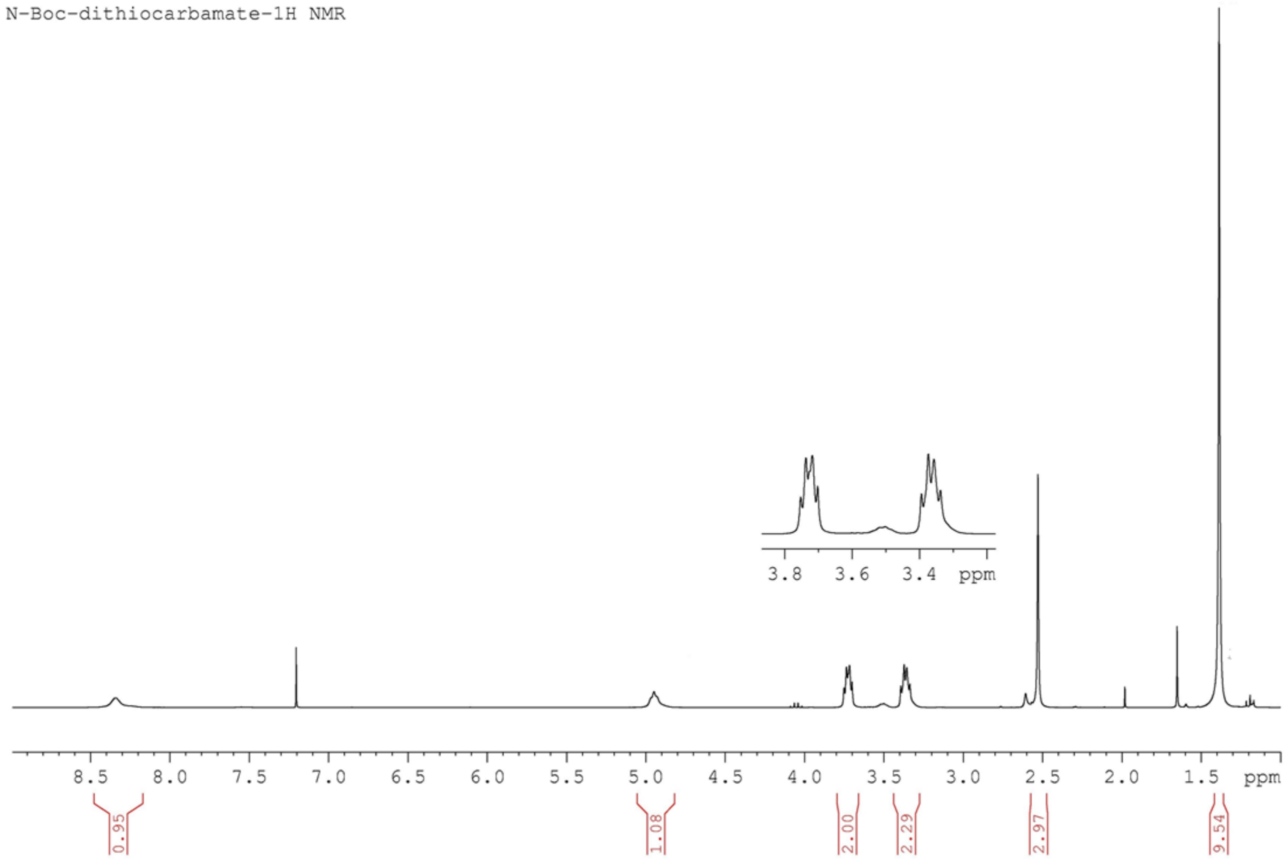

Figure S2 ^{1}H NMR of compound **2c** (300 MHz, 298 K, CDCl$_3$). Inset: The expansion of the 3.8 -3.2 ppm range. Assignments are given above.

Part B: Hydrazine monohydrate (8.09 mmol) was added dropwise to a stirring solution of the corresponding precursor **2a**, **2b** or **2c** (6.47 mmol, in EtOH, 15 mL) and then was allowed to react for 2.5 h under reflux conditions. Afterwards, the excess of solvent was removed under reduced pressure and the product was purified using a silica plug with CHCl$_3$ (50 mL) and MeOH (100 mL). The methanolic fraction was collected and

concentrated under *vacuum* to yield the desire compound. In the case of compound **3a**, the purified product was obtained by recrystallization in MeOH.

N-(4-fluorobenzyl)hydrazinecarbothioamide (3a):

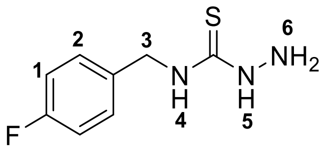

The product was obtained as white crystals in 71 % yield. **^{1}H NMR** (300 MHz , DMSO-d_6, 25 ºC): 8.76 (s, 1H, H5), 8.35 (brs, 1H, H4), 7.39-7.30 (m, 2H, H2/2'), 7.16-7.06 (m, 2H, H1/1'), 4.67 (d, 2H, ^3J = 6.1 Hz, H3), 4.49 (appq, 2H, H6). **^{13}C{^{1}H} NMR** (75 MHz, DMSO-d_6, 25 ºC): 181.8, 161.4 (d, $J_{C\text{-}F}$ = 241.7 Hz), 136.5 (d, $J_{C\text{-}F}$ = 3.0 Hz), 129.7 (d, $J_{C\text{-}F}$ = 8.0 Hz), 115.1 (d, $J_{C\text{-}F}$ = 21.1 Hz), 45.7. **^{19}F{^{1}H} NMR** (500 MHz, DMSO-d_6, 25 ºC): -113.85. **Mass spectrum**: ESI-MS calc. for $C_8H_{10}FN_3S$ $[M+H]^+$: 200.0652 found 200.0650.

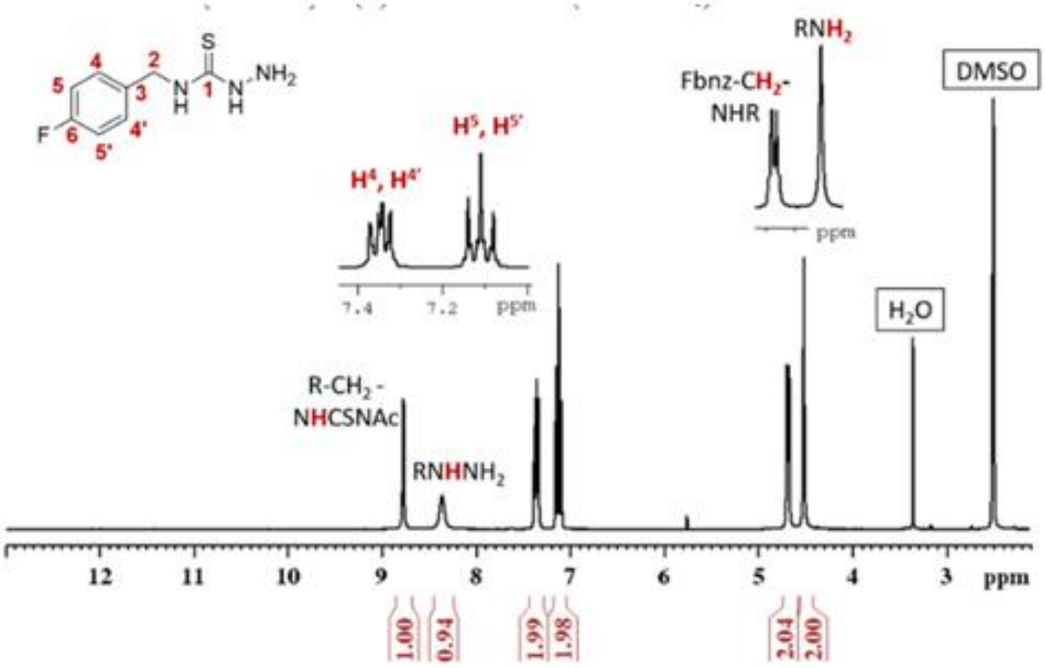

Figure S3 ^{1}H NMR of compound **3a** (300 MHz, 298 K, DMSO-d_6)

N-(4-ethynyl benzyl)hydrazinecarbothioamide (3b):

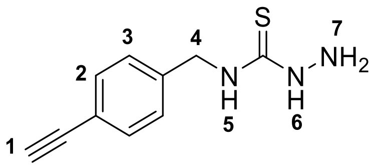

The product was obtained as white powder in 80 % yield. **^{1}H NMR** (300 MHz, DMSO-d_6, 25 ºC): 8.81 (s, 1H, H6), 8.41 (brs, 1H, H5), 7.42 (appd, 2H, ^3J = 8.1 Hz, H3/3'), 7.30 (appd, 2H, ^3J = 8.1 Hz, H2/2'), 4.72 (d, 2H, ^3J = 6.0 Hz, H4), 4.53 (bs, 2H, H7), 4.14 (s, 1H, H1). **^{13}C{^{1}H} NMR** (75 MHz, DMSO-d_6, 25 ºC): 181.9, 141.6, 127.9, 126.2, 120.2, 83.9, 80.9, 46.2. **Mass spectrum**: ESI-MS calc. for $C_{10}H_{11}N_3S$ $[M+H]^+$: 206.0752 found 206.0749.

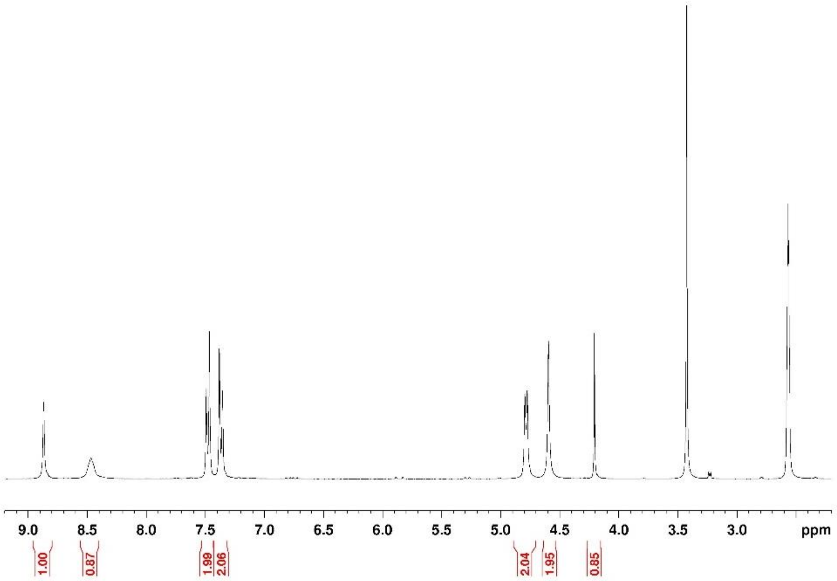

Figure S4 ^{1}H NMR of compound **3b** (300 MHz, 298 K, DMSO-d_6)

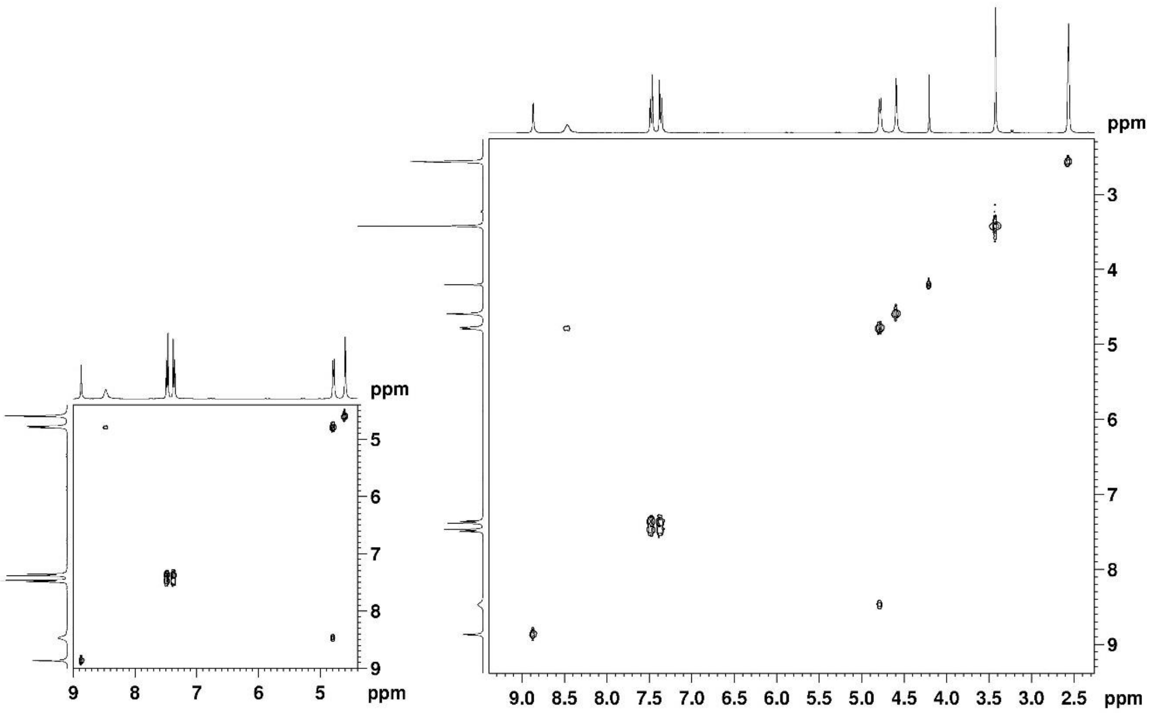

Figure S5 COSY NMR of compound **3b** (300 MHz, 298 K, DMSO-d_6)

4-N-(2-tert-butoxycarbonylaminoethyl)-3-dithiocarbamate (3c):

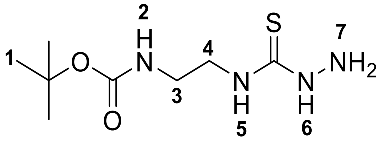

The product was obtained as a white solid in 68 % yield. **^{1}H NMR** (500 MHz, DMSO-d_6, 25 ºC): 8.68 (brs, 1H, H6), 7.94 (brs, 1H, H5), 4.99 (brs, 1H, H2), 4.44 (s, 2H, H7), 3.49 (appq, 2H, ^{3}J = 6.0 Hz, H3), 3.38 (appq, 2H, ^{3}J = 5.9 Hz, H4), 1.38 (s, 9H, H1). **^{13}C{^{1}H} NMR** (125 MHz, DMSO-d_6, 25 ºC): 197.9, 156.1, 78.2, 46.4, 38.5, 28.6. **Mass spectrum**: ESI-MS calc. for $C_8H_{18}N_4O_2S$ $[M+H]^+$: 235.1221 found 235.1223.

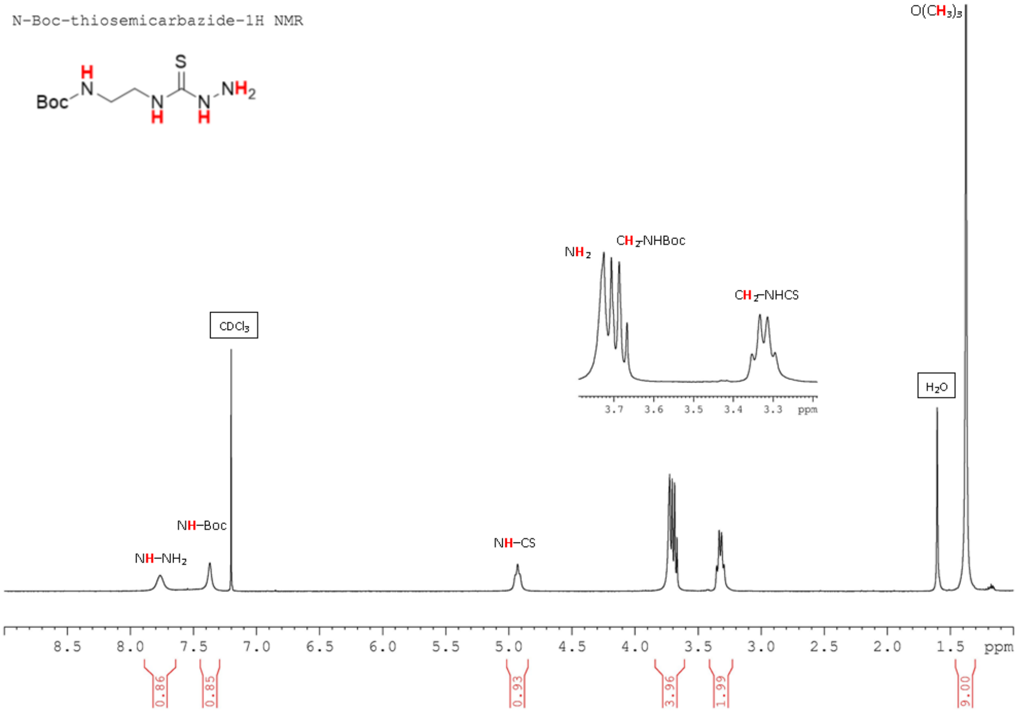

Figure S6 ^{1}H NMR of compound **3c** (500 MHz, 298 K, DMSO-d6)

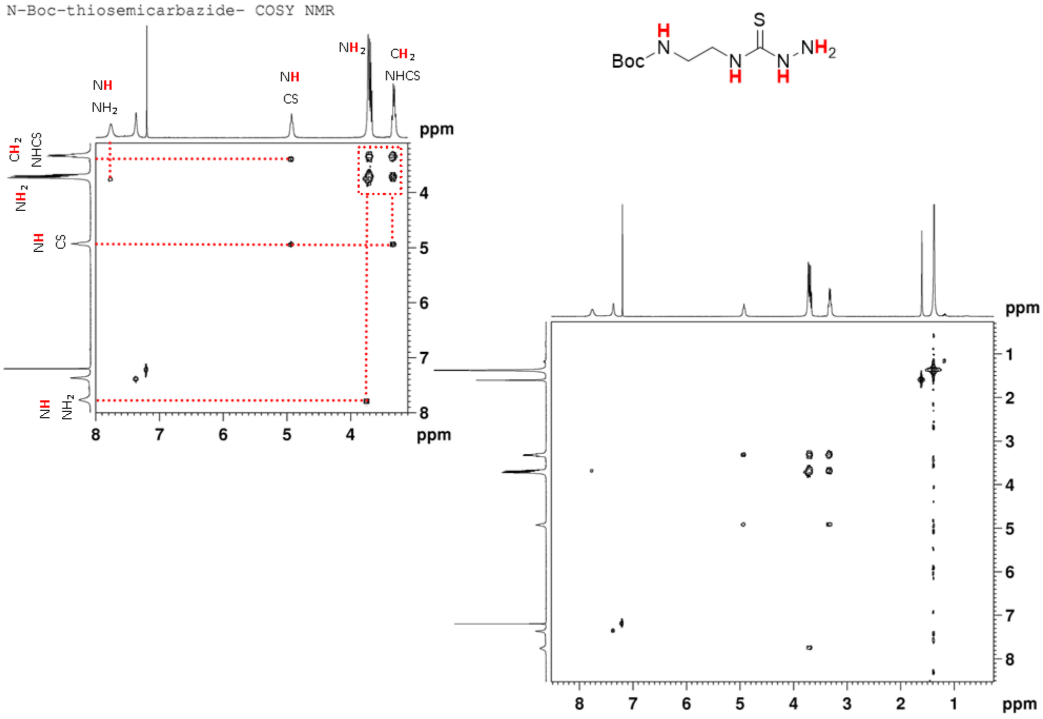

Figure S7 COSY NMR of compound **3c** (500 MHz, 298 K, DMSO-d_6)

2.2. General synthetic procedure for the synthesis of the acenaphthenequinone mono(thiosemicarbazones) ligands (4a-4c)

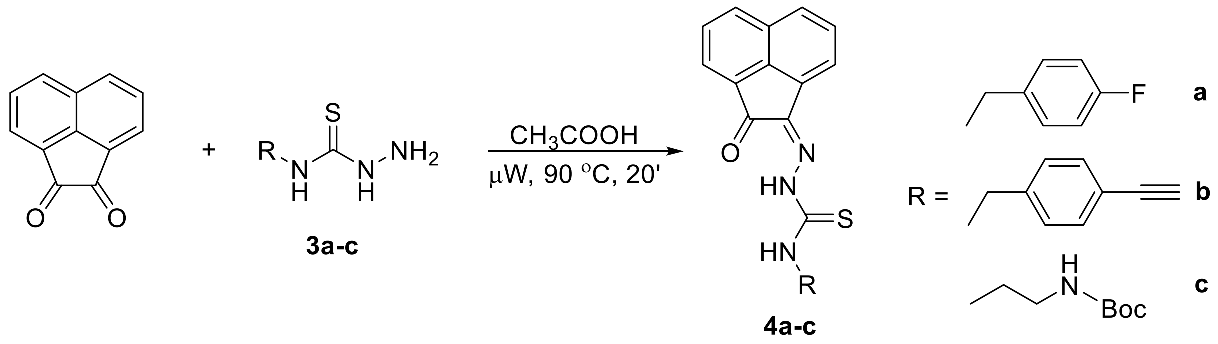

Scheme S2 Synthesis of mono(thiosemicarbazones) from acenaphthenequinone by microwave reaction.

A suspension of the acenaphthenoquinone (2.74 mmol) and 1 equiv. of the correspondent thiosemicarbazide (**3a-c**) (2.74 mmol) in 15 mL of acetic acid was placed in a 20 mL microwave tube and the mixture was reacted at 90 °C in the microwave for 20 min. The reaction mixture was allowed to cool to room temperature without stirring and then the precipitate was filtrated and washed with Et_2O. The solvent was removed under reduced pressure affording the desired compound.

Mono (4-fluorobenzyl)-3-thiosemicarbazone acenaphthenequinone (4a):

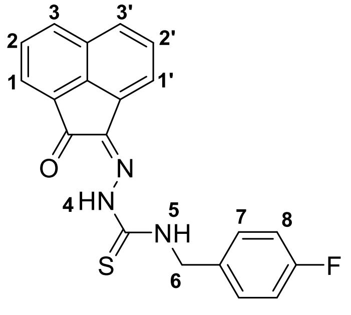

The product was obtained as a yellow powder in 88 % yield. **^{1}H NMR** (500 MHz, DMSO-d_6, 25 ºC): 12.68 (s, 1H, H4), 9.93 (t, 1H, ^3J = 6.3 Hz, H5), 8.36 (d, 1H, ^3J = 8.3 Hz, H3), 8.12 (d, 1H, ^3J = 8.3 Hz, H3'), 8.08 (d, 1H, ^3J = 6.9 Hz, H1), 7.98 (d, 1H, ^3J = 6.9 Hz, H1'), 7.87 (t, 1H, J = 7.6 Hz, H2), 7.81 (t, 1H, J = 7.7 Hz, H2'), 7.43 (dd, 2H, J = 8.4 Hz, 5.5 Hz, H7/7'), 7.17 (t, 2H, J = 8.8 Hz, H8/8'), 4.88 (d, ^{3}J = 6.3 Hz, H6). **^{13}C{^{1}H} NMR** (500 MHz, DMSO-d_6, 25 ºC): 189.0, 178.4, 161.8 (d, J = 242.5 Hz), 139.7, 138.0, 135.0 (d, J = 2.9 Hz), 133.2, 130.9, 130.5, 130.4, 129.9 (d, J = 8.1 Hz), 129.3, 129.1, 127.6, 123.0, 118.8, 115.6 (d, J = 21.4 Hz), 47.0. **^{19}F{^{1}H} NMR** (500 MHz, DMSO-d_6, 25 ºC): -115.74. **Mass spectrum**: ESI-MS calc. for $C_{20}H_{14}FN_3OS$ $[M+H]^+$: 364.0920 found 364.0915. **IR** (ATR, cm^{-1}): 3320, 3269, 1692, 1607, 1520, 1453, 1082, 1026, 853, 775. **HPLC (Method A)**: Rt = 11.33 min

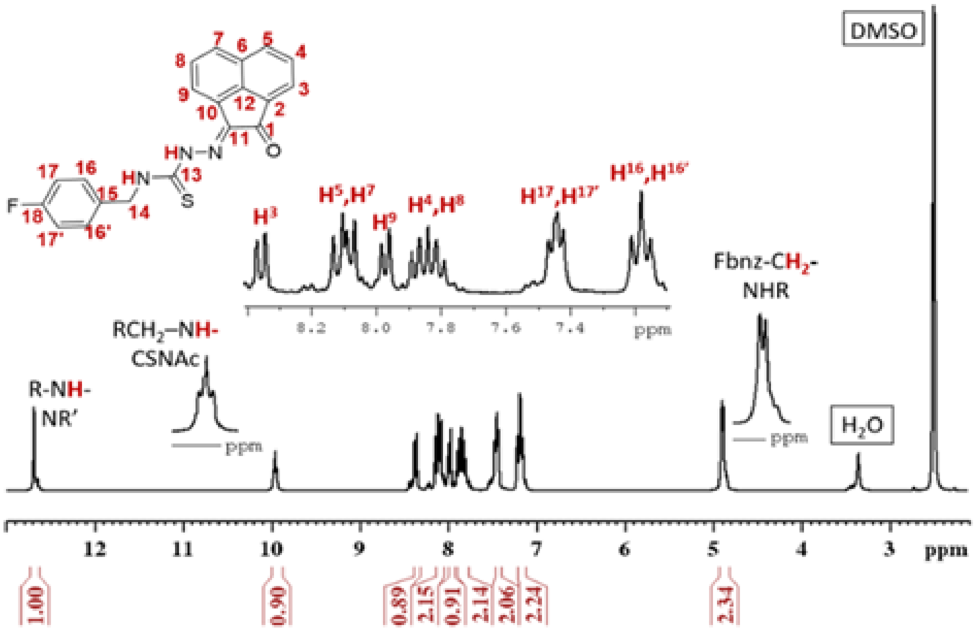

Figure S8 ¹H NMR of compound **4a** (500 MHz, 298 K, DMSO-d6)

Mono (4-ethynylbenzyl)-3-thiosemicarbazone acenaphthenequinone (4b):

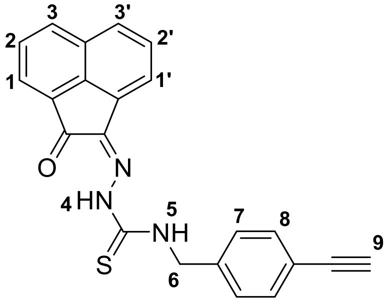

The product was obtained as a yellow powder in 86 % yield. **¹H NMR** (300 MHz, DMSO-d6, 25 ºC): 12.69 (s, 1H, H4), 9.97 (t, 1H, ^3J = 5.8 Hz, H5), 8.35 (d, 1H, ^3J = 8.1 Hz, H1), 8.12 (d, 1H, ^3J = 12.9 Hz, H3'), 8.08 (d, 1H, ^3J = 11.3 Hz, H3), 7.97 (d, 1H, ^3J = 6.9 Hz, H1'), 7.90-7.78 (m, 2H, J = 7.6 Hz, H2, H2'), 7.43 (dd, 4H, J = 7.7 Hz, 16.1 Hz, H7/7', H8/8') 4.91 (d, 2H, ^3J = 5.6 Hz, H6), 4.16 (s, 1H, H9). **$^{13}C\{^1H\}$ NMR** (125 MHz, DMSO-d6, 25 ºC): 188.9, 178.4, 139.8, 139.6, 138.0, 133.2, 132.1, 130.8, 130.4, 130.3, 129.2, 129.0, 127.9, 127.5, 122.9, 120.7, 118.7, 83.8, 81.1, 47.4. **Mass spectrum**: ESI-MS calc. for $C_{22}H_{15}N_3OS$ $[M+H]^+$: 507.1497 found 507.1483. **IR** (ATR, cm^{-1}): 3259, 3236, 2950, 1684, 1527, 1452, 1082, 1026. **HPLC (Method A)**: Rt = 11.37 min.

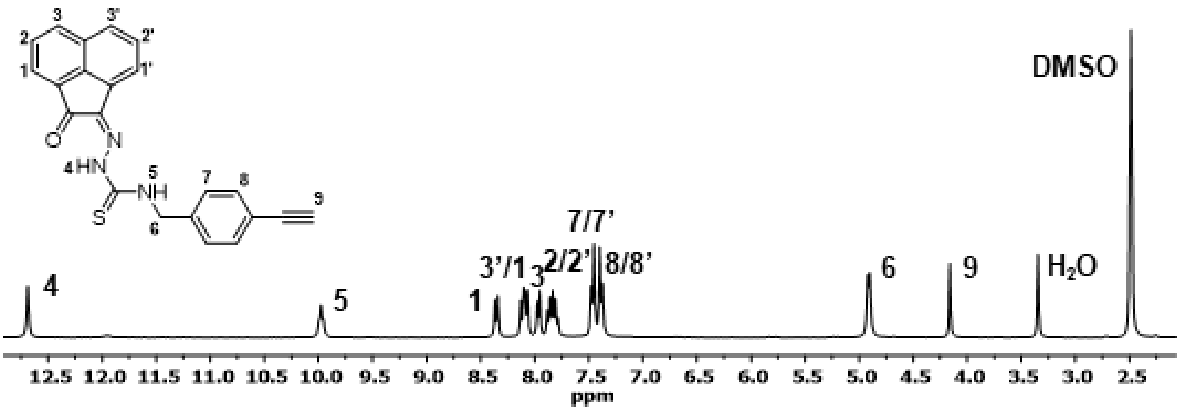

Figure S9 ¹H NMR of compound **4b** (300 MHz, 298 K, DMSO-d6)

Mono (4-tertbutyl-(2-aminoethyl)carbamate)-3-thiosemicarbazone acenaphthenequinone (4c):

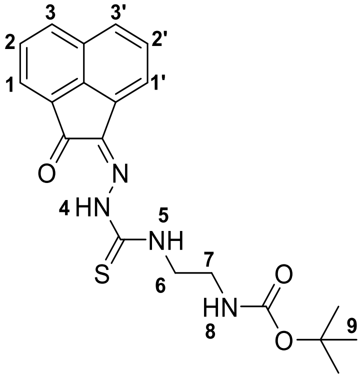

The product was obtained as a yellow powder in 88 % yield. **^{1}H NMR** (300 MHz, DMSO-d$_6$, 25 ºC): 12.61 (s, 1H, H4), 9.41 (t, ^3J = 5.6 Hz, 1H, H5), 8.37 (dd, $^{3,4}J$ = 8.2, 0.7 Hz, 1H, H3), 8.13 (dd, $^{3,4}J$ = 8.4, 0.7 Hz, 1H, H3'), 8.09 (dd, $^{3,4}J$ = 7.1, 0.7 Hz, 1H, H1), 8.01 (d, ^3J = 7.0 Hz, 1H, H1'), 7.87 (dd, $^{3,3}J$ = 8.2, 7.0 Hz, 1H, H2), 7.83 (dd, $^{3,3}J$ = 8.3, 7.0 Hz, 1H, H2'), 7.06 (t, ^3J = 5.7 Hz, 1H, H8), 3.65 (q, ^3J = 5.9 Hz, 2H, H6), 3.25 (q, ^3J = 6.2 Hz, 2H, H7), 1.38 (s, 9H, H9). **^{13}C{^{1}H} NMR** (125 MHz, DMSO-d$_6$, 25 ºC): 188.5, 177.6, 156.2, 139.5, 137.2, 132.8, 130.5, 130.0, 129.9, 128.9, 128.4, 127.1, 122.5, 118.2, 78.0, 44.9, 39.2, 28.2. **Mass spectrum**: ESI-MS calc. for $C_{20}H_{22}N_4NaO_3S$ [M+Na]$^+$: 421.1310; found: 421.1329. **IR** (ATR, cm^{-1}): 3384, 3326, 3257, 2980, 1719, 1685, 1670, 1512, 1480. **HPLC (Method A)**: Rt = 10.04 min.

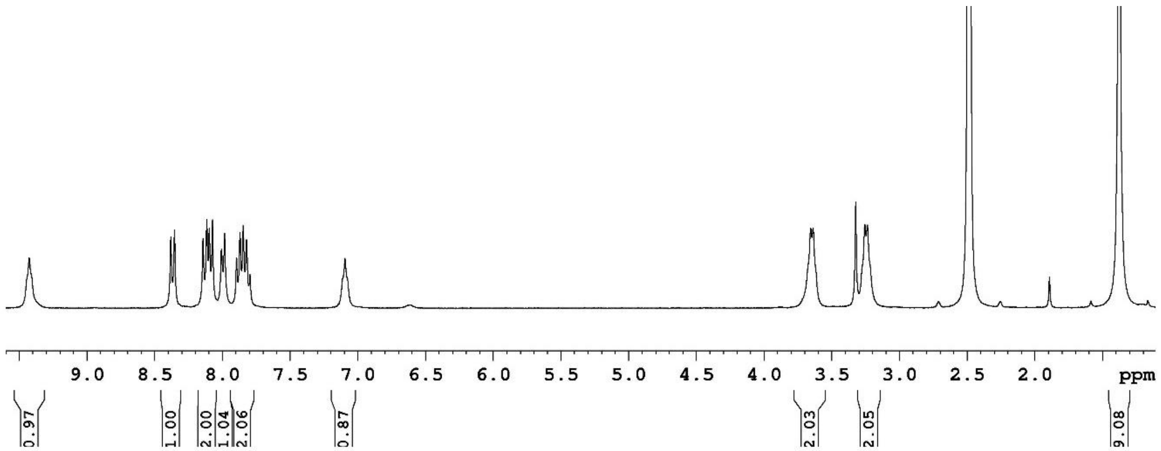

Figure S10 ^{1}H NMR of compound **4c** (300 MHz, 298 K, DMSO-d6)

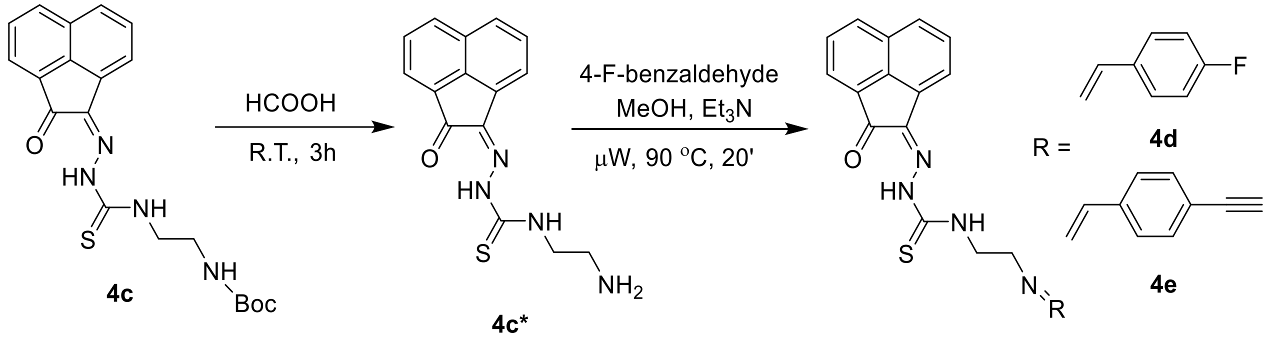

Scheme S3 Synthetic pathway for the formation of different aminoethyl thiosemicarbazone ligands.

Mono (4-(2-aminoethyl)-3-thiosemicarbazone) acenaphthenequinone (4c*):

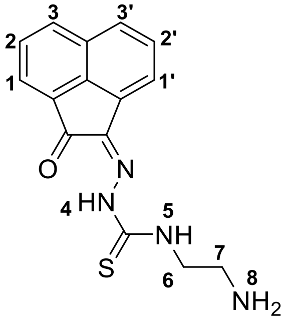

A suspension of the compound **4c** (1.63g, 4.1 mmol) in 80 mL of formic acid was stirred at room temperature for 3h. The solvent was removed under reduced pressure and the compound washed with toluene. The desired product was obtained as a yellow powder in 91 % yield. **^{1}H NMR** (300 MHz, DMSO-d_6, 25 ºC): 12.72 (s, 1H, H4), 9.68 (t, ^{3}J = 5.8 Hz, 1H, H5), 8.39 (dd, $^{3,4}J$ = 8.2, 0.7 Hz, 1H, H3), 8.29 – 8.19 (m, 2H, H8), 8.16 (dd, $^{3,4}J$ = 8.5, 0.7 Hz, 1H, H3'), 8.11 (dd, $^{3,4}J$ = 7.0, 0.7 Hz, 1H, H1), 8.10 (dd, $^{3,4}J$ = 7.0, 0.7 Hz, 1H, H1'), 7.89 (dd, $^{3,3}J$ = 8.2, 7.1 Hz, 1H, H2), 7.85 (dd, $^{3,3}J$ = 8.4, 7.0 Hz, 1H, H2'), 3.95 (q, ^{3}J = 6.3 Hz, 2H, H6), 3.11 (s, 2H, H7). **^{13}C{^{1}H} NMR** (125 MHz, DMSO-d_6, 25 ºC): 188.6, 178.0, 139.3, 137.6, 132.9, 130.4, 129.9, 129.9, 128.9, 128.6, 127.2, 122.5, 118.6, 41.8, 37.7. **Mass spectrum**: ESI-MS calc. for $C_{15}H_{15}N_4OS$ $[M+H]^+$: 299.0967; found: 299.0959. **IR** (ATR, cm^{-1}): 3330, 3255, 2836, 1693, 1523, 1467, 1452, 1050. **HPLC** (**Method A**): Rt = 7.42 min.

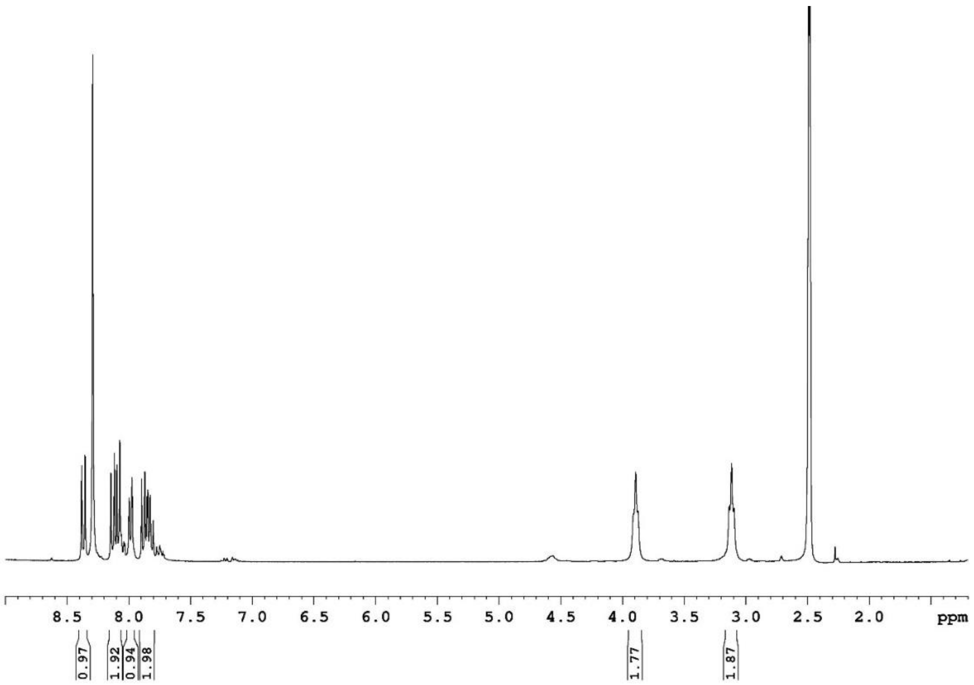

Figure S11 ^{1}H NMR of compound 4**c*** (300 MHz, 298 K, DMSO-d_6)

Mono(2-(4-fluorobenzylidene)aminoethyl)-3-thiosemicarbazone) acenaphthenequinone (4d):

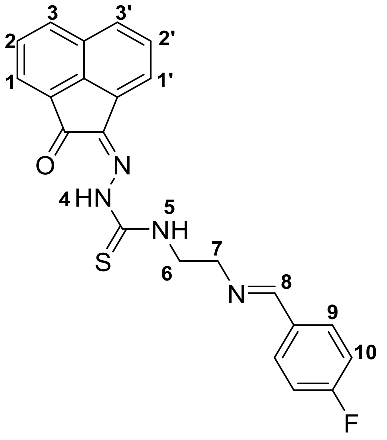

A suspension of the compound **4c*** (1.50g, 5.03 mmol), 1 equiv of 4-(fluorobenzaldehyde) (539.30µL, 5.03 mmol) in MeOH (20mL) and 3 drops of triethylamine was placed in a 20 mL microwave tube and the mixture was reacted at 90 °C in the microwave for 20 min. The reaction mixture was allowed to cool to room temperature without stirring and then the precipitate was filtrated and washed with Et_2O and hexane. The solvent was removed under reduced pressure affording the desired compound. The product was obtained as a yellow powder in 62 % yield. **^{1}H NMR** (300 MHz, DMSO-d_6, 25 ºC): 12.15 (s, 1H, H4), 9.37 (bt, 1H, H5), 8.44 (s, 1H, H8), 8.37 (d, $^{3,4}J$ = 8.2 Hz, 1H, H3), 8.13 (d, $^{3,4}J$ = 8.2 Hz, 1H, H3'), 8.09 (d, $^{3,4}J$ = 7.0 Hz, 1H, H1), 7.91-7.83 (m, 4H, H1', H2, H9, H9'), 7.80 (t, $^{3,4}J$ = 7.6 Hz, 1H, H2'), 7.29 (t, $^{3,3}J$ = 8.8 Hz, 2H, H10,H10'), 3.94 (d, ^3J = 5.7 Hz, 2H, H6), 3.91 (s, 2H, H7). **^{13}C{^{1}H} NMR** (75.5 MHz, DMSO-d_6, 25 ºC): 189.0, 178.0, 164.1 (d, J_{C-F} = 248.1 Hz), 161.6, 139.6, 137.8, 133.3, 133.1 (d, J = 2.8 Hz), 130.9, 130.7 (d, J = 8.8Hz), 130.4, 130.3, 129.3, 129.1, 127.6, 123.0, 118.6, 116.1 (d, J = 21.8 Hz), 59.0, 45.3. **^{19}F{^{1}H} NMR** (500 MHz, DMSO-d_6, 25 ºC):-109.86. **Mass spectrum**: ESI-MS calc. for $C_{22}H_{17}F_1N_4OS$ [M+H]$^+$: 405.1185; found:405.1193. **IR** (ATR, cm^{-1}): 3318, 3250, 1681, 1602, 1527, 1481, 1178, 1028, 937, 825, 791, 773. **HPLC** (**Method A**): Rt = 7.68 min.

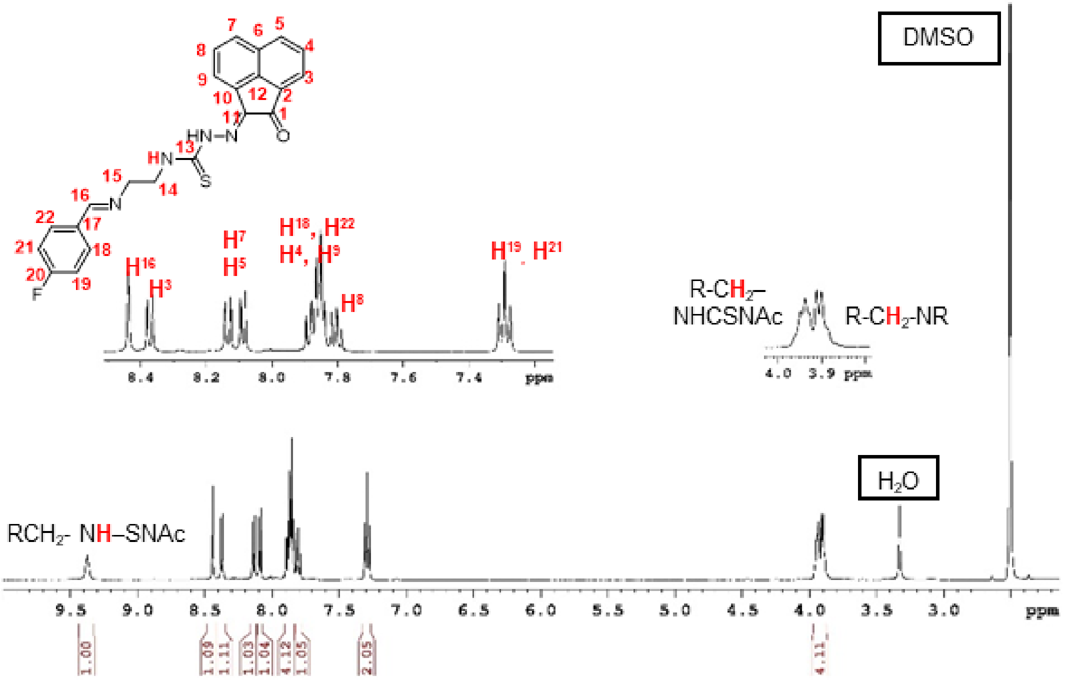

Figure S12 ^{1}H NMR spectroscopy of compound **4c*** (300 MHz, 298 K, DMSO-d_6)

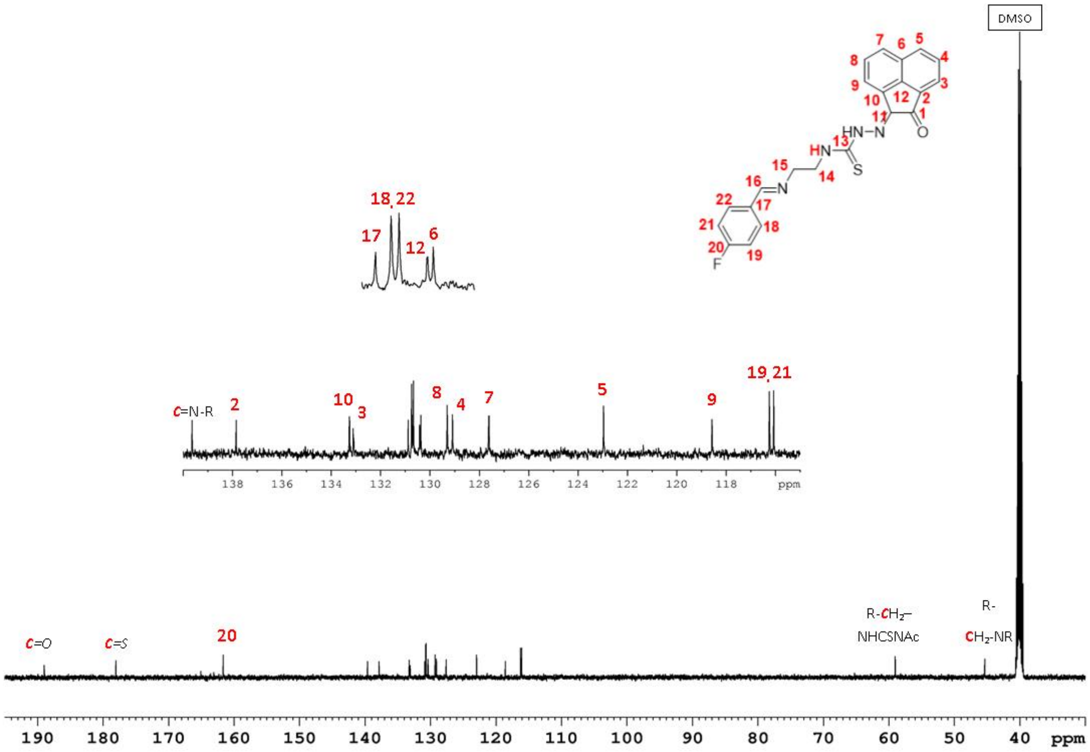

Figure S13 ^{13}C NMR of compound **4c*** (75.5 MHz, 298 K, DMSO-d_6)

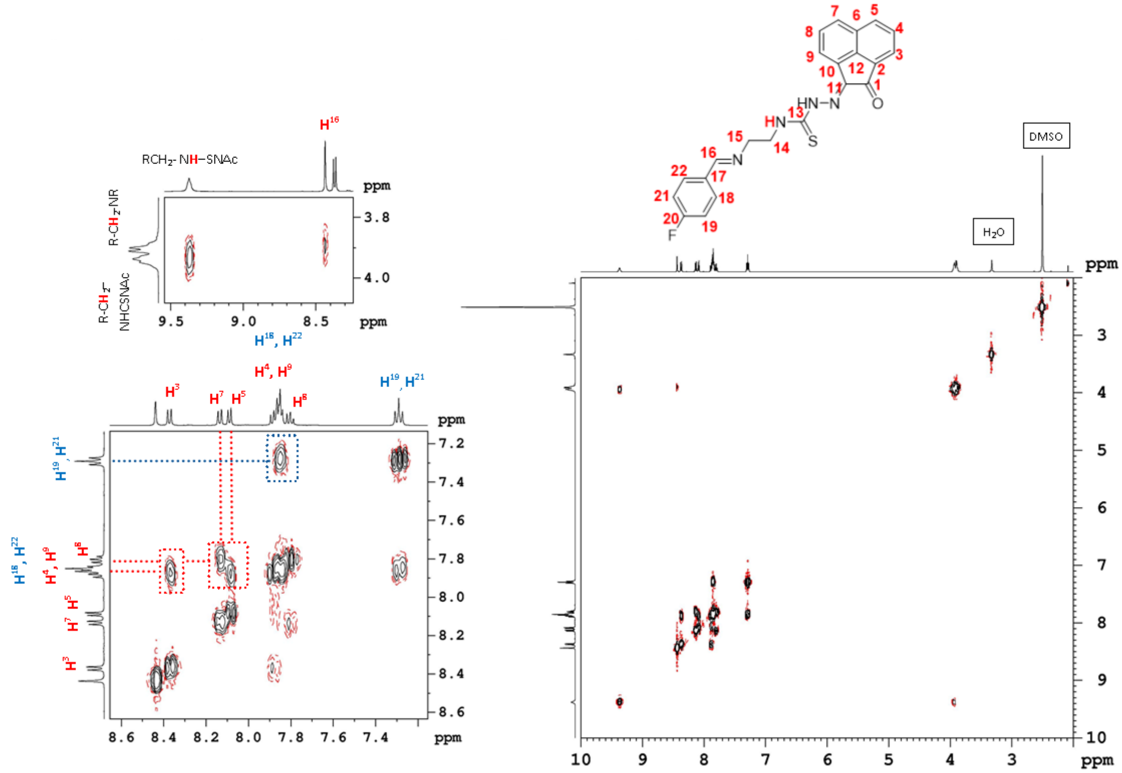

Figure S14 COSY NMR of compound **4c*** (300 MHz, 298 K, DMSO-d_6)

Mono(2-(4-ethynylbenzylidene)aminoethyl)-3-thiosemicarbazone) acenaphthenequinone (4e):

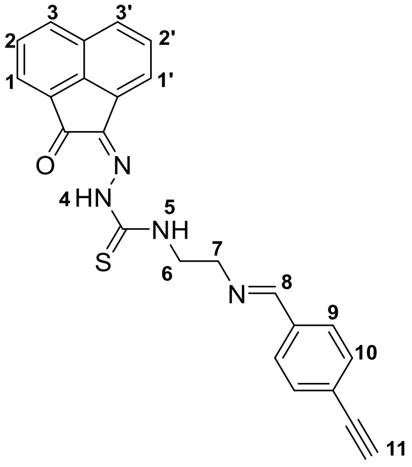

A suspension of the compound **4c*** (1.50g, 5.03 mmol), 1 equiv of 4-(ethynylbenzaldehyde) (654.30 mg, 5.03 mmol) in MeOH (20mL) was placed in a 20 mL microwave tube and the mixture was reacted at 90 °C in the microwave for 20 min. The reaction mixture was allowed to cool to room temperature without stirring and then the precipitate was filtrated and washed with Et_2O and hexane. The solvent was removed under reduced pressure affording the desired compound. The product was obtained as a yellow powder in 59 % yield. **^{1}H NMR** (300 MHz, DMSO-d_6, 25 ºC): 12.64 (s, 1H, H4), 9.37 (bt, 1H, H5), 8.46 (s, 1H, H8), 8.37 (d, $^{3,4}J$ = 8.2 Hz, 1H, H3), 8.13 (d, $^{3,4}J$ = 8.1 Hz, 1H, H3'), 8.09 (d, $^{3,4}J$ = 7.0 Hz, 1H, H1), 7.88 (t, $^{3,4}J$ = 7.6 Hz, 1H, H2), 7.85 (d, $^{3,4}J$ = 6.9 Hz, 1H, H1'), 7.82-7.78 (m, 3H, H2', H9, H9'), 7.56 (d, $^{3,4}J$ = 8.0 Hz, H10, H10'), 4.35 (s, 1H, H8), 3.99-3.89 (m, 4H, H6, H7). **^{13}C{^{1}H} NMR** (75.5 MHz, DMSO-d_6, 25 ºC): 188.9, 178.0, 162.2, 139.6, 137.9, 136.6, 133.3, 132.5, 130.9, 130.4, 130.3, 129.3, 129.1, 128.6, 127.6, 124.4, 123.0, 118.6, 83.6, 83.12, 59.2, 45.3. **Mass spectrum**: ESI-MS calc. for $C_{24}H_{18}N_4OS$ $[M+H]^+$: 411.1280; found:411.1291. **IR** (ATR, cm^{-1}): 3279, 3213, 1685, 1606, 1532, 1476, 1180, 1024, 935, 828, 777. **HPLC (Method A)**: Rt = 7.683min.

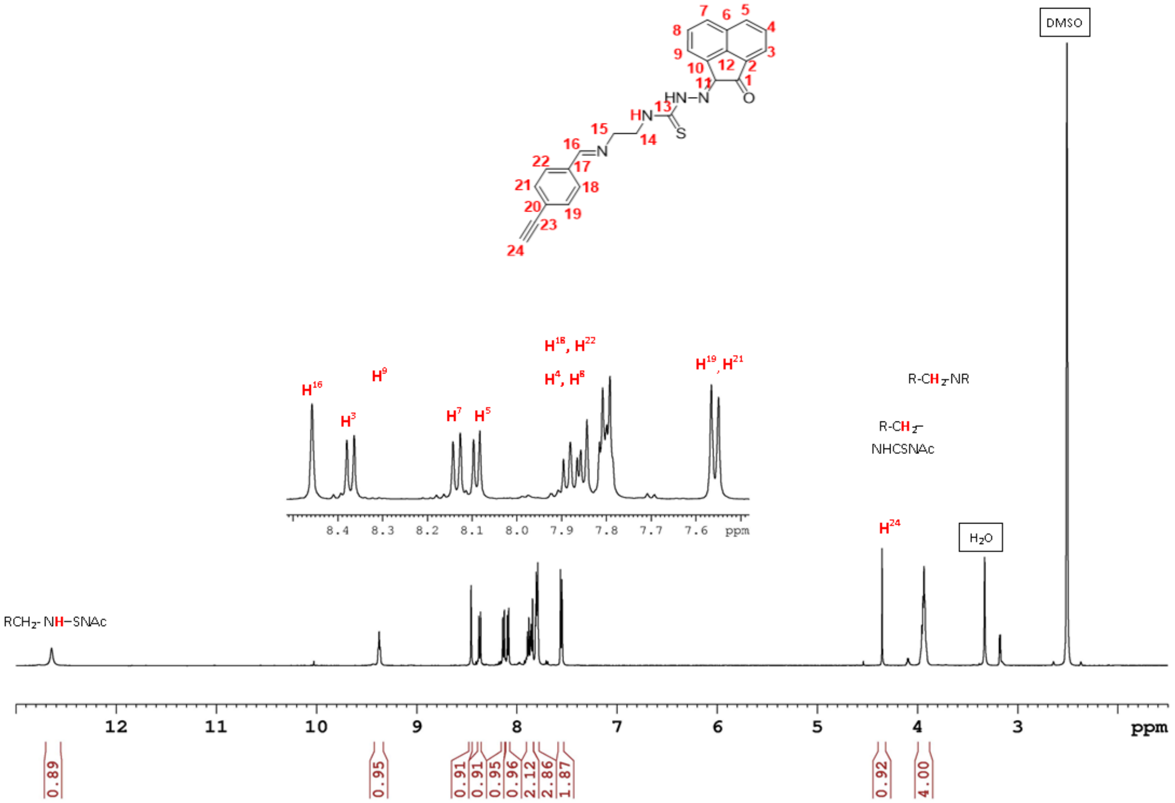

Figure S15 ^{1}H NMR of compound **4e** (300 MHz, 298 K, DMSO-d_6)

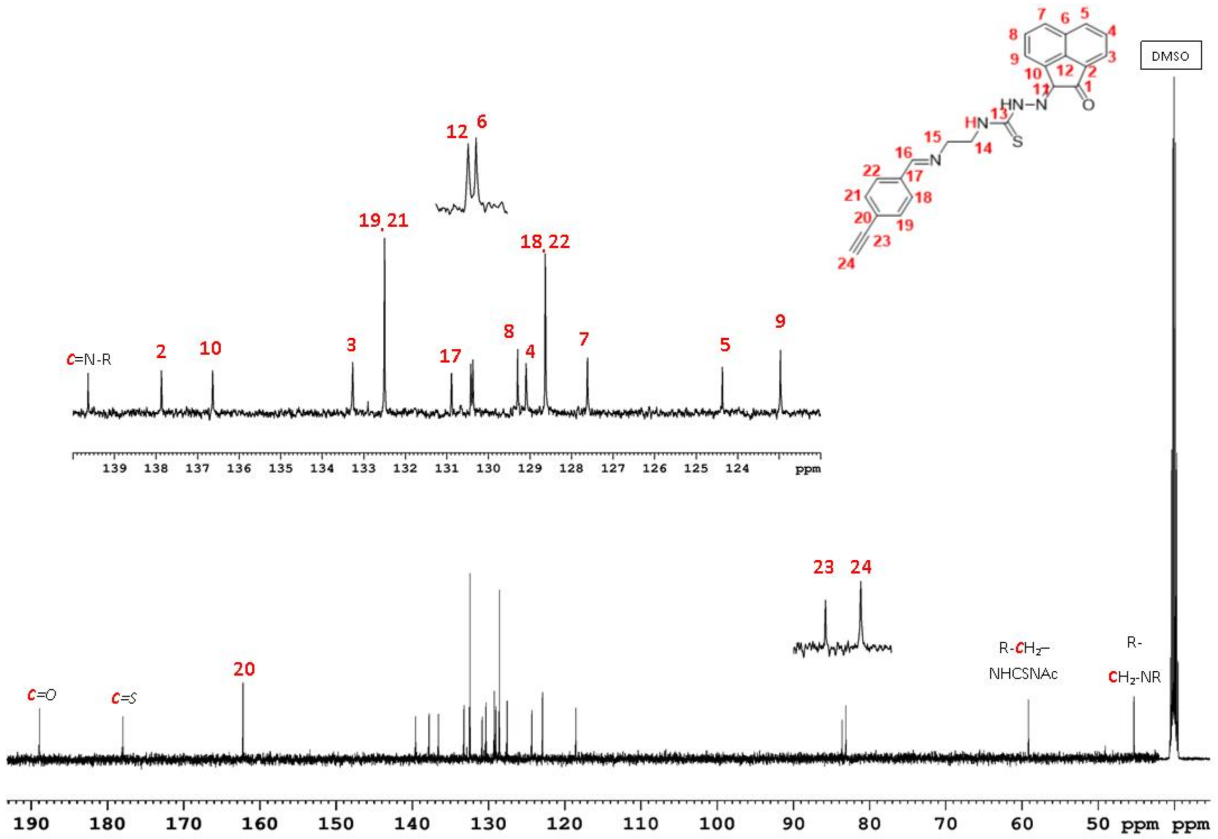

Figure S16 $^{13}C\{^{1}H\}$ NMR of compound **4e** (75.5 MHz, 298 K, DMSO-d_6)

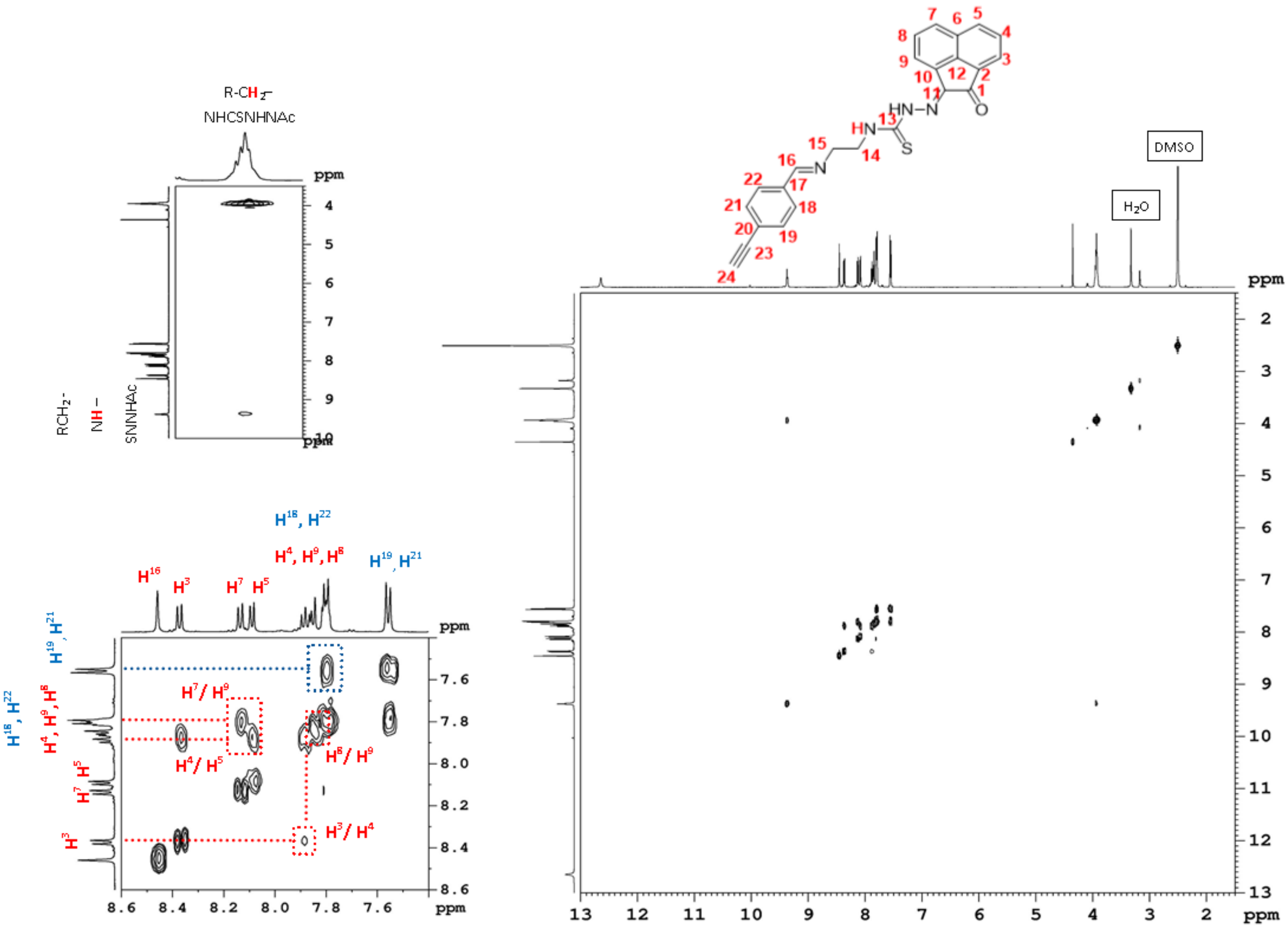

Figure S17 COSY NMR of compound **4e** (300 MHz, 298 K, DMSO-d6)

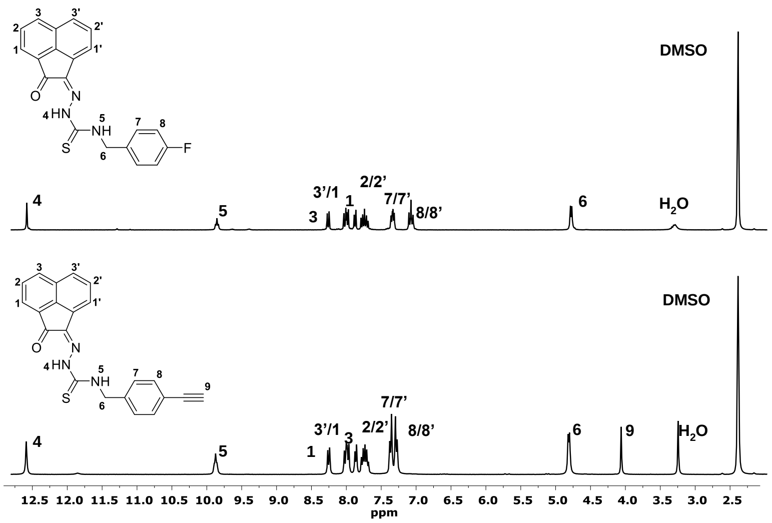

Figure S18 Comparative ^{1}H NMR spectroscopy in DMSO-d6 of compounds **4a** and **4b**

Mono (4-ethyl-3-thiosemicarbazone) acenaphthenequinone (4f)

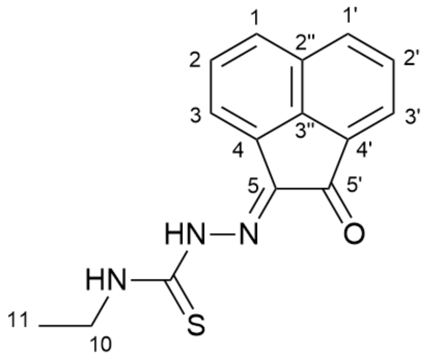

Acenaphthenequinone (0.20 g, 1.10 mmol) and 4-ethyl-3-thiosemicarbazide (0.12 g, 1.00 mmol) were mixed in 10 ml EtOH. Then 150 µl concd. HCl was added. The mixture was irradiated at 90 ℃ for 10 min in a microwave reactor. The precipitate was filtered off whilst hot, washed with MeOH and Et_2O, dried to obtain a yellow solid (0.20 g, 0.70 mmol, 70 %).

^{1}H NMR (400 MHz, DMSO-d_6, 25℃) δ 12.62 (s, 1H, H-7), 9.44 (t, J = 5.9 Hz, 1H, H-9), 8.40 (d, J = 0.7 Hz, 1H, H-1'), 8.16 (d, J = 0.7 Hz, 1H, H-1), 8.12 (d, J = 0.7 Hz, 1H, H-3'), 8.03 (d, J = 0.7 Hz, 1H, H-3), 7.95 – 7.82 (m, 2H, H-2, H-2'), 3.75 – 3.64 (m, 2H, H-10), 1.28 – 1.17 (m, 3H, H-11).

^{13}C NMR (101 MHz, DMSO-d_6, 25℃) δ 189.04 (C-5'), 177.40 (C-8), 139.58 (C-3''), 137.67 (C-5), 133.28 (C-1'), 130.98 (C-4'), 130.60 (C-4), 130.41 (C-2), 129.39 (C-2'), 129.11 (C-2''), 127.55 (C-1), 122.97 (C-3'), 118.77 (C-3), 39.1 (C-10), 14.55 (C-11).

ESI-MS calculated for $C_{15}H_{14}N_3OS^+$ $[M+H]^+$ 284.0855, found 284.0854.

Alternative methods for the preparation of mono(4-ethyl-thiosemicarbazone)acenaphthenequinone (4f)

The ligand was obtained as reported in the literature from a 1:1 molar ratio of acenaphthenequinone and thiosemicarbazide in absolute ethanol. Hereby, the reaction were carried out using acenaphthenequinone (0.5 g, 2.74mmol) and 4-ethyl-3-thiosemicarbazide (0.358 g, 3.00 mmol), which were suspended in absolute ethanol (15ml) and heated under reflux for 2 hours. The solid was isolated by filtration whilst hot then re-suspended in hot methanol (10ml) and stirred for 15 minutes before filtering and washing with further methanol. The resulting solid was then dried under vacuum purity checked using HPLC under various conditions. Yield = 0.659 g, 2.329 mmol, 85%.

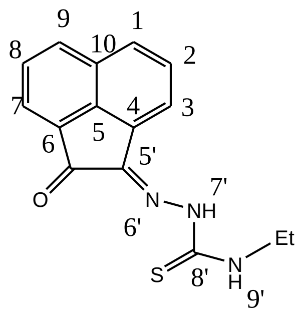

^{1}H NMR (300 MHz, d_6 –DMSO, 25°C): δ 12.45 (s, 1H, N-N(H)), 9.45 (t, 1H, N(H)Et), 8.35 (d, 1H, H7), 8.10 (d, 1H, H3), 8.05 (d, 1H, H9), 7.95 (d, 1H, H1), 7.80 (t+t, 2H, H2 H8), 3.65 (m, 2H, $(CH_2)CH_3$), 1.25 (t, 3H, $CH_2(CH_3)$). **^{13}C NMR** (300 MHz, d_6 –DMSO, 25°C): δ 188.9 (CO), 177.0 C-8'), 139.5(C-5'), 137.5 (C-5), 133.2(C-3), 130.9(C-4), 130.5(C-10), 130.3(C-8), 129.4(C-2), 129.3(C-6), 129.0(C-9), 122.9(C-7), 118.7(C-1), 39.6(CH_2), 14.5(CH_3). **IR**: 3279 w υ(N-H), 3059 m υ(N-H), 1686 s υ(C=O), 1607 s υ(C=N), 1536 s υ(C=N), 1475 sb υ(ring), 1452 s υ(ring), 1140 m υ(C-S). **ES-MS**: m/z = 284.1 $[M + H]^+$. **HPLC** R_f = 17.3 mins (Method B). **Elemental analysis**: Calculated N(14.84 %), C(63.60 %), H(4.59 %). Found N(14.52 %), C(63.12 %), H(4.52 %).

Selected analytical data for 4f

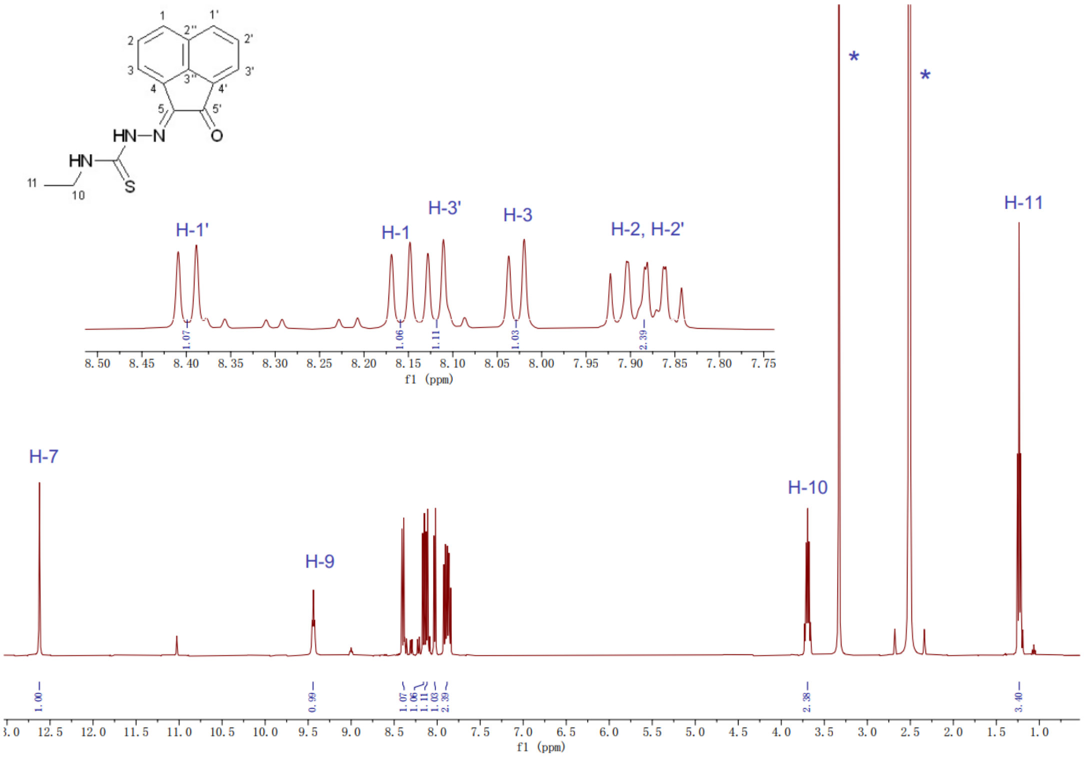

Figure S19 ^{1}H NMR spectrum (400 MHz, 298 K, DMSO-d_6) of compound **4f** (* correspond to the residual solvents, acetic acid, DMSO-d_6 and H_2O).

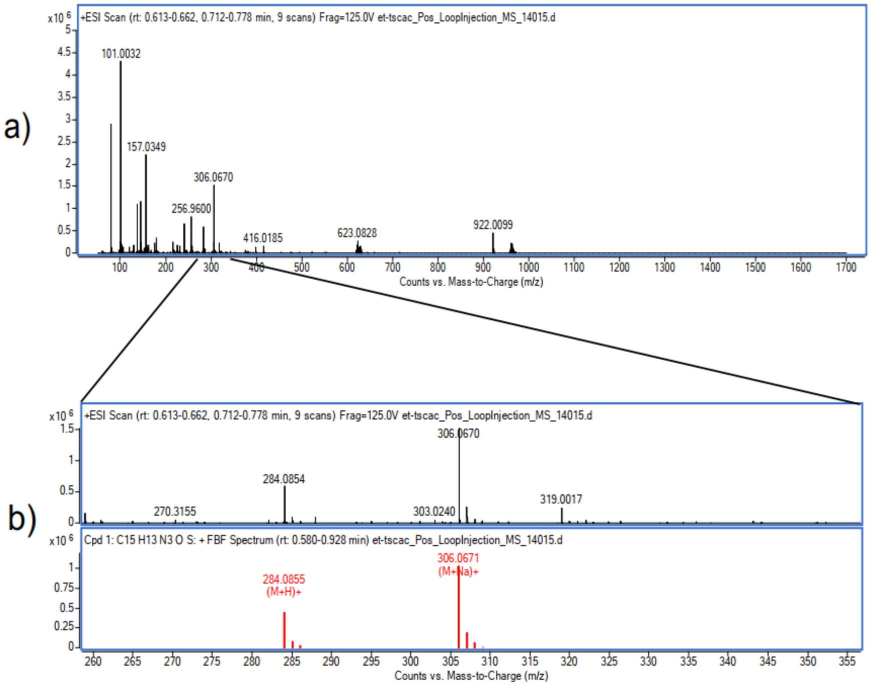

Figure S20 ESI-TOF Mass spectrum of compound **4f**. a). Observed data of isotope model of $[M+H]^+$; b) Zoomed in version of a): the top row shows the observed data of isotope model of $[M+H]^+$, the bottom row shows the theoretical isotope model of $[M+H]^+$ and $[M+Na]^+$.

Mono (4-allyl-3-thiosemicarbazone) acenaphthenequinone (4g)

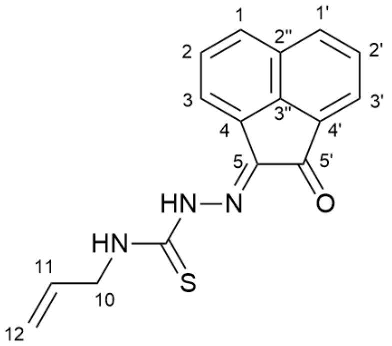

Acenaphthenequinone (0.20 g, 1.10 mmol) and 4-allyl-3-thiosemicarbazide (0.13 g, 1.00 mmol) were mixed in 10 ml EtOH. Then 150 μl concd. HCl was added. The mixture was irradiated at 90 ℃ for 10 min in a microwave reactor. The precipitate was filtered off whilst hot, washed with MeOH and Et_2O, dried to obtain a yellow solid (0.26 g, 0.86 mmol, 87 %).

^{1}H NMR (400 MHz, DMSO-d_6, 25℃) δ 12.68 (s, 1H, H-7), 9.60 (t, *J* = 6.0 Hz, 1H, H-9), 8.40 (d, *J* = 8.1 Hz, 1H, H-1'), 8.16 (d, *J* = 8.3 Hz, 1H, H-1), 8.12 (d, *J* = 6.9 Hz, 1H, H-3'), 8.04 (d, *J* = 6.9 Hz, 1H, H-3), 7.91 (dd, *J* = 8.2, 7.0 Hz, 1H, H-2'), 7.86 (dd, *J* = 8.4, 7.0 Hz, 1H, H-2), 6.02 – 5.91 (m, 1H, H-11), 5.25 (dd, *J* = 17.2, 1.7 Hz, 1H, H-12_{trans}), 5.18 (dd, *J* = 10.3, 1.6 Hz, 1H, H-12_{cis}), 4.34 – 4.29 (m, 2H, H-10).

ESI-MS calculated for $C_{16}H_{14}N_3OS^+$ $[M+H]^+$ 296.0852, found 296.0828.

Synthesis of mono (4-allyl-3-thiosemicarbazone) acenaphthenequinone (**4g**)

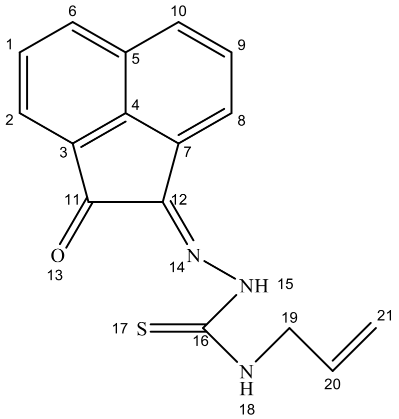

Compound **4g** was prepared as a result of the reaction between acenaphthenequinone (0.1 g, 0.5489 mMol) and 4-allyl-3-thiosemicarbazone (0.0864 g, 0.6587 mMol) in a 1:2 ratio. Both compounds were dissolved in 40ml of ethanol and a few drops of glacial acetic acid were added. The reaction was refluxed at a maximum temperature of 100°C for 4 hours. Following the removal of methanol with evaporation, a dark yellow solid was isolated by filtration and washing with diethyl ether. Yield = 94%

^{1}H NMR (300MHz, d^6-DMSO, 25°C): δ 12.98 (s, 1H, H15), 9.52 (s, 1H, H18), 8.56 (d, 1H, H2), 8.42 (d, 1H, H8), 8.37 (d, 1H, H6), 8.05 (d, 1H, H10), 7.96 (t, 1H, H1), 7.84 (t, 1H, H9), 5.84 (m, 1H, H20), 5.32 (dd, 1H, H21(a)), 5.22 (dd, 1H, H21(b)), 4.43 (m, 2H, H19). **ES MS**: M/z = 318.0657 $[M+Na]^+$, M/z = 296 $[M+H]^+$

Mono (4-phenyl-3-thiosemicarbazone) acenaphthenequinone (4h)

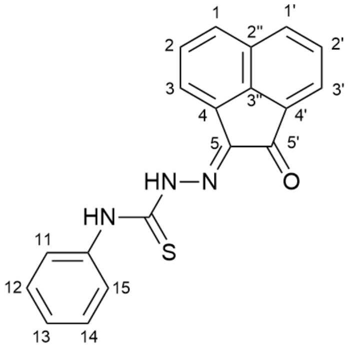

Acenaphthenequinone (0.20 g, 1.10 mmol) and 4-phenylthiosemicarbazide (0.16 g, 1.00 mmol) were mixed in 10 ml EtOH. Then 150 µl concd. HCl was added. The mixture was irradiated at 90 ℃ for 10 min in a microwave reactor. The precipitate was filtered off whilst hot, washed with MeOH and Et_2O, dried to obtain an orange solid (0.20 g, 0.62 mmol, 62 %).

^{1}H NMR (400 MHz, DMSO-d_6, 25℃) δ 12.85 (s, 1H, H-7), 10.98 (s, 1H, H-9), 8.42 (d, *J* = 8.1 Hz, 1H, H-1'), 8.18 (d, *J* = 8.5 Hz, 1H, H-1), 8.16 (dd, *J* = 6.9, 3.6 Hz, 2H, H-3, H-3'), 7.95 – 7.85 (m, 2H, H-2, H-2'), 7.65 (d, *J* = 7.4 Hz, 2H, H-11, H-15), 7.46 (t, *J* = 7.8 Hz, 2H, H-12, H-14), 7.31 (t, *J* = 7.4 Hz, 1H, H-13).

Alternative method for the preparation of mono(4-phenyl-3-thiosemicarbazone)acenaphthenequinone (4h)

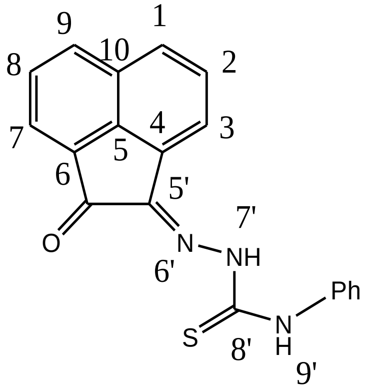

Synthesis as for **4f** using acenapthenequinone (0.50 g, 2.74 mmol) and (0.468 g, 2.80 mmol) 4-phenyl-3-thiosemicarbazide, a yellow solid is obtained. Yield = 0.620g, 1.867 mmol, 68 %.

^{1}H NMR (300 MHz, d_6 –DMSO, 25°C): δ 12.79 (s, 1H, N-N(H)), 10.96 (t, 1H, N(H)Ph), 8.38 (d, 1H, H7), 8.14 (d, 1H, H3), 8.12 (d, 1H, H9), 8.01 (d, 1H, H1), 7.88(t, 2H, H8), 7.84(t, 2H, H2), 7.62 (d, 4H, o-CH), 7.44 (t, 4H, m-CH), 7.29(t, 2H, p-CH). **^{13}C NMR** (300 MHz, d_6 –DMSO, 25°C): δ 188.9 (CO), 177.0(C-8'), 139.7 (C-5'), 138.9 (C-Ph), 138.0 (C-5), 133.2 (C-3), 130.7 (C-), 130.3(C-), 129.3(C-), 129.1(C-), 129.0(C-2), 128.9(C-6), 127.6 (C-9), 126.6(C-Ph), 126.2(C-Ph), 122.9(C-7), 119.3(C-1). **IR**: 3297.83w υ(N-H), 3054.26w υ(N-H), 1682.56s υ(C=O), 1597.14s υ(C=N), 1565.58s υ(C=N), 1475.73m υ(ring), 1445.36 s υ(ring), 1146.85 m υ(C-S). **ES-MS**: m/z = 333.0 $[M + H]^+$**.**

HPLC R_f = 20.5 mins (**Method B**)

Scheme S4: General synthetic procedure for the synthesis of the Zinc acenaphthenequinone thiosemicarbazones complexes using microwave irradiation

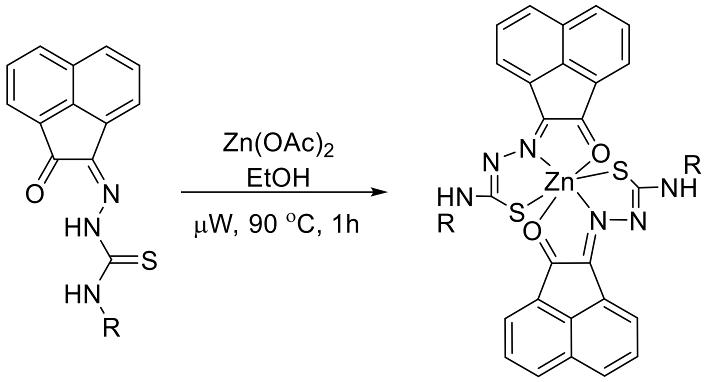

Synthesis of Zn(II) bis [mono (F-benzyl thiosemicarbazonate) acenaphthenequinone] (4a-Zn):

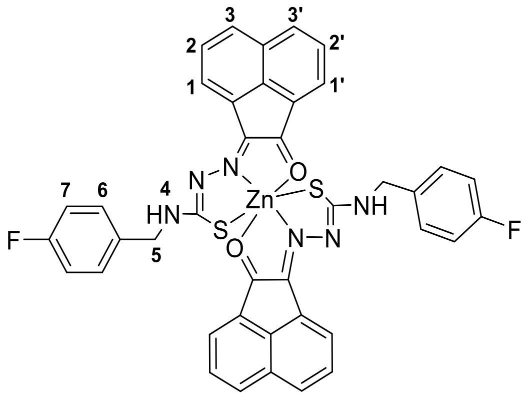

A suspension of anhydrous zinc acetate (201.94 mg, 1.10 mmol) and the respective acenaphthenequinone mono(thiosemicarbazone) ligand (200.0 mg, 0.55 mmol) in EtOH was placed in a sealed microwave glass tube. The mixture was reacted at 90 °C under microwave irradiation for 1 h. The precipitated was then filtrated, washed twice with Et_2O and dried under vacuum. The product was obtained as a yellow powder in 62 % yield **^{1}H NMR** (500 MHz, DMSO-d_6, 25 ºC): 8.96 (appd, 2H, ^3J = 5.4 Hz, N***H***-bnzF); 8.56 (appt, 2H, ^3J = 7.5 Hz, ***H9***); 8.34 (appt, 2H, ^3J = 7.9 Hz, ***H3***); 8.12 (appt, 4H, ^3J = 7.3 Hz, ***H5 H7***); 7.89 (appt, 4H, ^3J = 6.6 Hz, ***H4 H8***); 7.77 (m, 8H, ***H16 H17 H19 H20***); 4.89 (t, 4H, ^3J = 5.46 Hz, ***H14***) ppm. **^{13}C{^{1}H} NMR** (125 MHz, DMSO-d_6, 25 ºC): 189.9, 178.2, 163.3, 160.1, 139.6, 135.0 (d, *J* = 3.01 Hz), 133.1, 130.8, 130.3 (d, *J* = 8.3 Hz), 129.8 (d, *J* = 8.3 Hz), 129.6, 128.9, 129.2, 128.9, 127.5, 122.9, 118.7, 115.3. **^{19}F{^{1}H} NMR** (500 MHz, DMSO-d_6, 25 ºC):-116.44. **Mass spectrum**: ESI-MS calc. for $C_{40}H_{26}F_2N_6O_2S_2Zn$ $[M+H]^+$: 789.0896 found 789.0912. **IR (ATR, cm^{-1}):** 3243, 2936, 1538, 1449, 1392, 1081, 1022, 773. **HPLC (Method A)**: Rt = 11.43, 14.47 min.

Synthesis of Zn(II) bis[mono(4-ethyl-3-thiosemicarbazone) acenaphthenequinone] using conventional heating (4f-Zn)

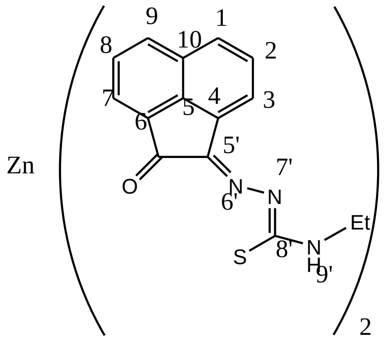

Mono(4-ethyl-thiosemicarbazone)-acenaphthenequinone (0.387 g, 1.366 mmol), and zinc acetate bishydrate (0.150 g, 0.683 mmol) were suspended in ethanol (50ml) with 10 drops of HCl (35 %) and heated under reflux for 24 hours. The red-orange solid is isolated by filtration whilst hot. The suspension is filtered and washed with diethyl ether (50 ml) and the desired compound was isolated as a red-orange solid. Yield = 0.65g, 1.0281 mmol, 75 %.

^{1}H NMR (300 MHz, d_6–DMSO, 25ºC): δ δ 9.52 (m, 1H, N(H)), 9.03 (m, 1H, N(H)), 8.64 (d, 1H, H7), 8.43 (d, 1H, H7), 8.28 (d+d, 2H, H3), 8.09 (d, 1H, H9), 8.06 (d, 1H, H9), 7.96(d+d, 2H, H1), 7.86(t, 1H, H8), 7.82(t, 1H, H8), 7.78(t, 1H, H2), 7.74(t, 1H, H2), 3.73 (m, 2H, $(CH_2)CH_3$), 3.64(m, 2H,

$(CH_2)CH_3$), 1.31(t, 3H, $CH_2(CH3)$), 1.25 (t, 3H, $CH_2(CH3)$). ^{13}**C NMR** (125 MHz, d_6 –DMSO, 25°C): δ 188.3(C-7''), 187.4(C-7''), 182.6(C-8'), 139.7(C-5'), 138.5(C-5'), 137.4 (C-5), 136.6(C-5), 132.8(C-3), 132.6 (C-3), 130.3, 130.2, 129.8, 129.7, 129.1, 128.8, 128.6, 128.3, 128.2, 127.3, 123.6 (C7), 123.5(C7), 122.9(C1), 122.8 (C1), 39.2(CH_2), 14. 3(CH_3). **IR**: 3319 w υ(N-H), 2930 m υ(N-H), 1683 s υ(C=O), 1606 s υ(C=N), 1577 s υ(C=N), 1496 s υ(ring), 1450 s υ(ring), 1171 m υ(C-S), 1139s υ(NN). **ES MS**: m/z = 629.1 $[M + H]^+$

Synthesis of zinc bis[mono (4-allyl-3-thiosemicarbazone) acenaphthenequinone] (4g-Zn)

Mono(4-allyl-3-thiosemicarbazone)acenaphthenequinone, (0.05g, 0.169mMol) was added to zinc acetate (0.0372g, 0.169mMol) on a 1:1 ratio. Approximately 20ml of tetrahydrothiophene, THF, was added dropwise consistently over a period of 1 hour. After the addition the reaction was left for another 2 hours and then the solvent evaporated before filtration and isolation. Diethyl ether was used to wash the orange solid obtained to remove any impurities. Yield = 73%.

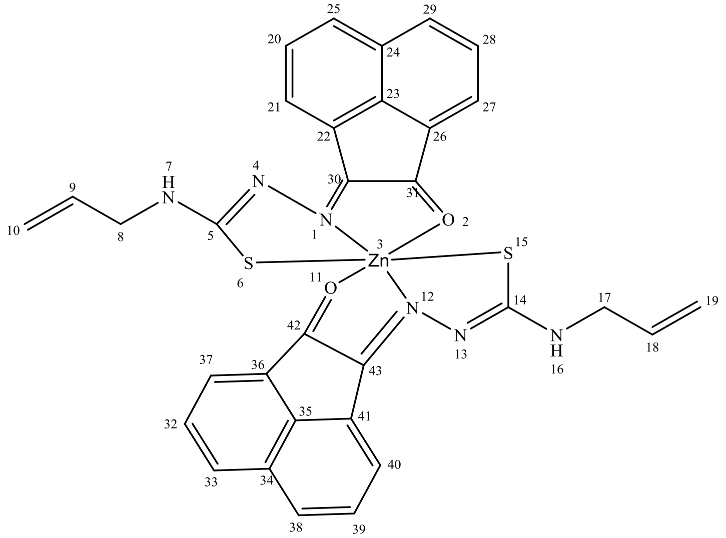

^{1}**H NMR** (300MHz, d^6-DMSO, 25°C): δ 8.16 (d, 2H, H21 + H27), 8.10 (t, 1H, NH), 7.98 (d, 2H, H29 + H25), 7.75 (t, 2H, H28 + H20), 5.98 (m, 1H, H18), 5.25 (dd, 1H, H19(a)), 5.10 (dd, 1H, H19(b)), 4.16 (bm, 2H, H17). **ES MS:** M/z =675 [M+Na]+ with the expected isotopic pattern

3. Optical spectroscopy of selected monothiosemicarbazones and kinetic stability tests

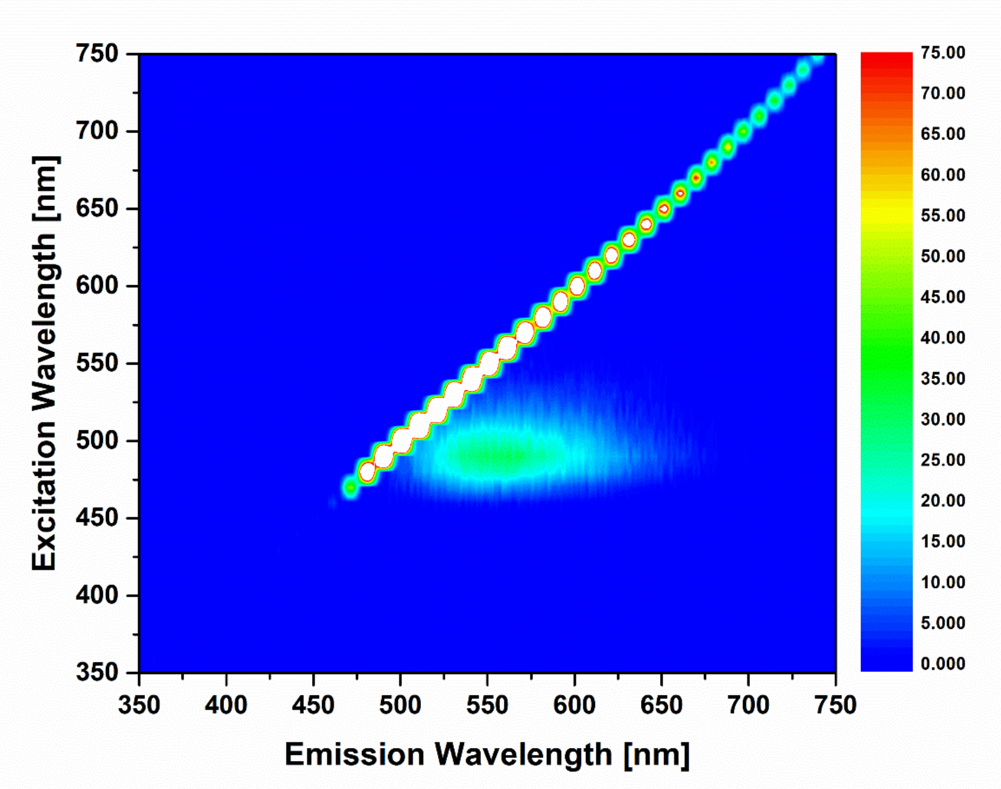

Figure S21 Excitation-Emission map for compound **4a** - two-dimensional fluorescence contour plot (100 uM in DMSO)

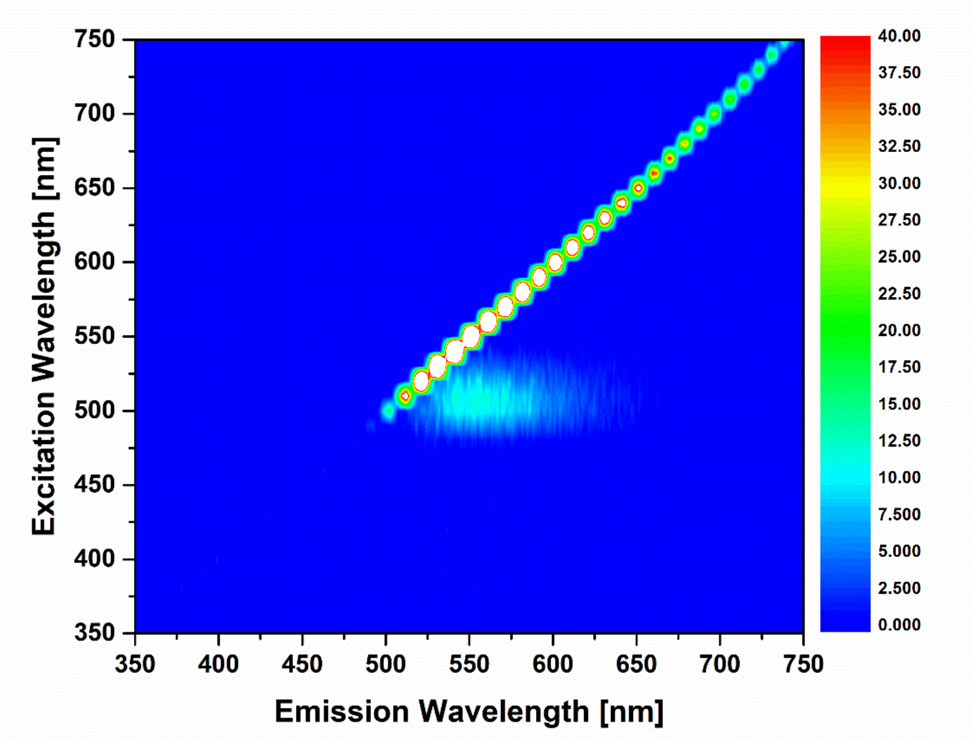

Figure S22. Excitation-Emission map for compound **4a-Zn** - two-dimensional fluorescence contour plot of (100 uM in DMSO).

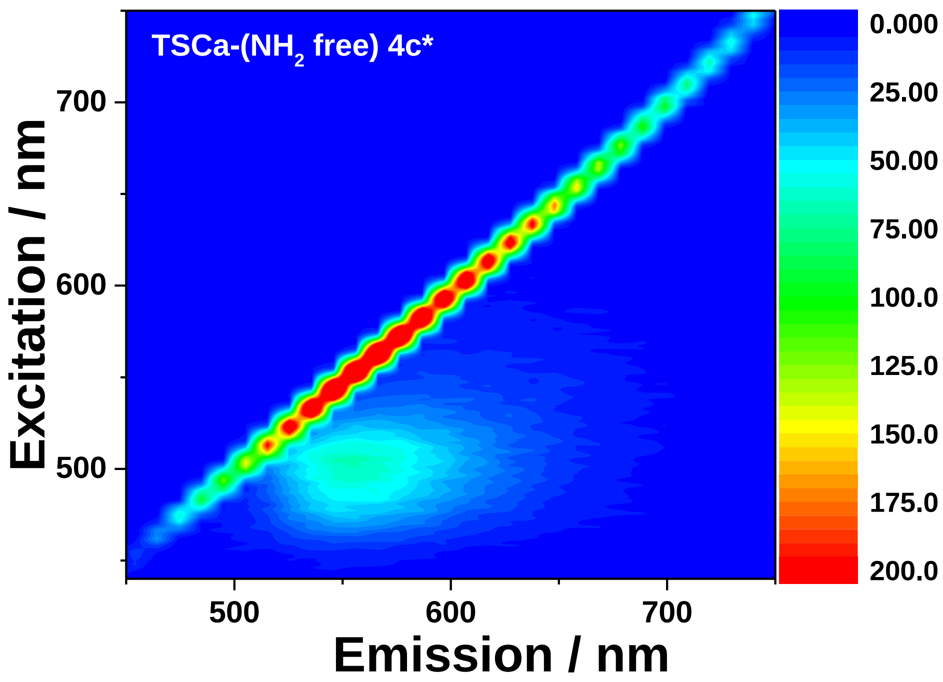

Figure S23 Excitation-Emission map for compound **4c*** - two-dimensional fluorescence contour plot (100 uM in DMSO)

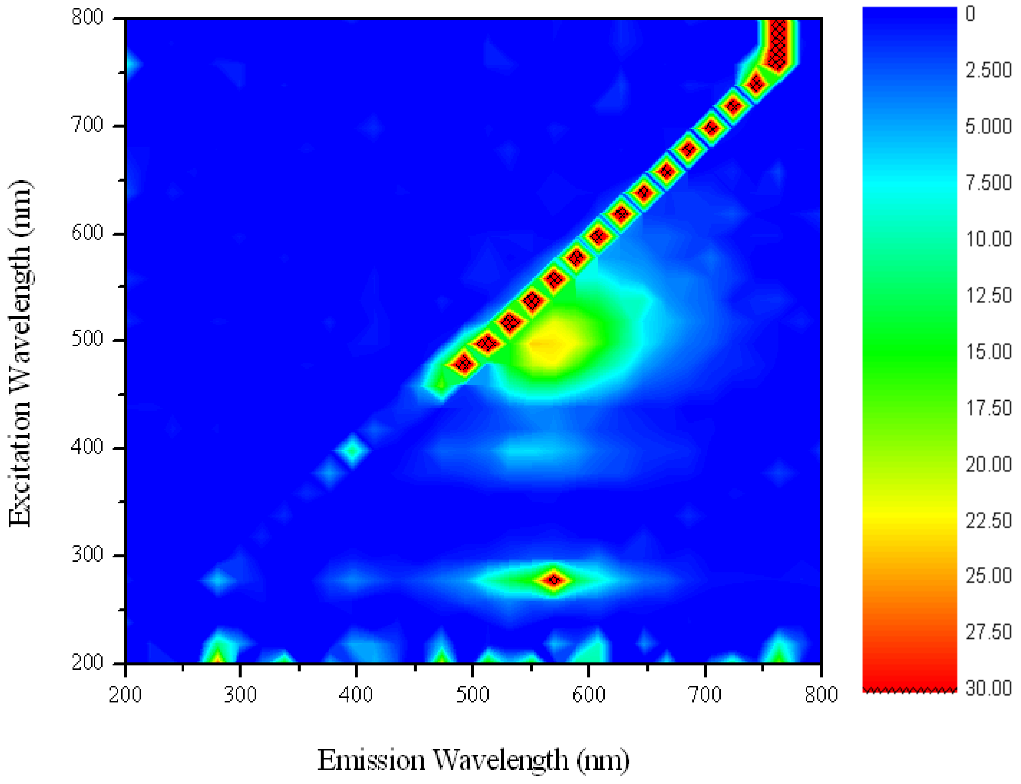

Figure S24

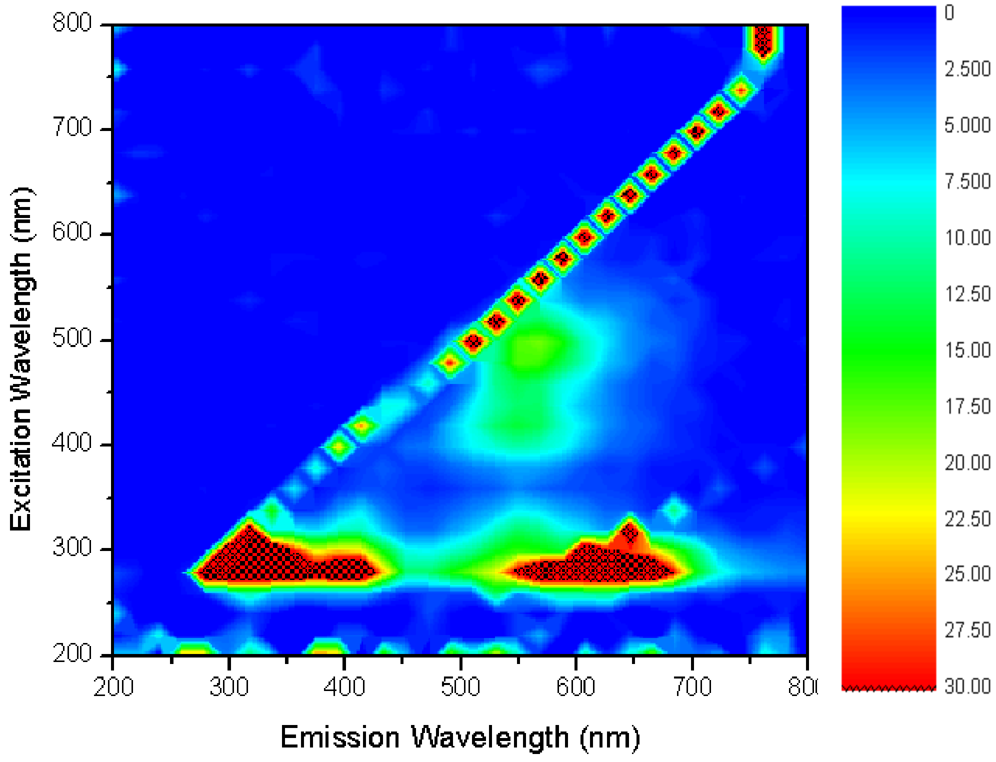

Figure S25

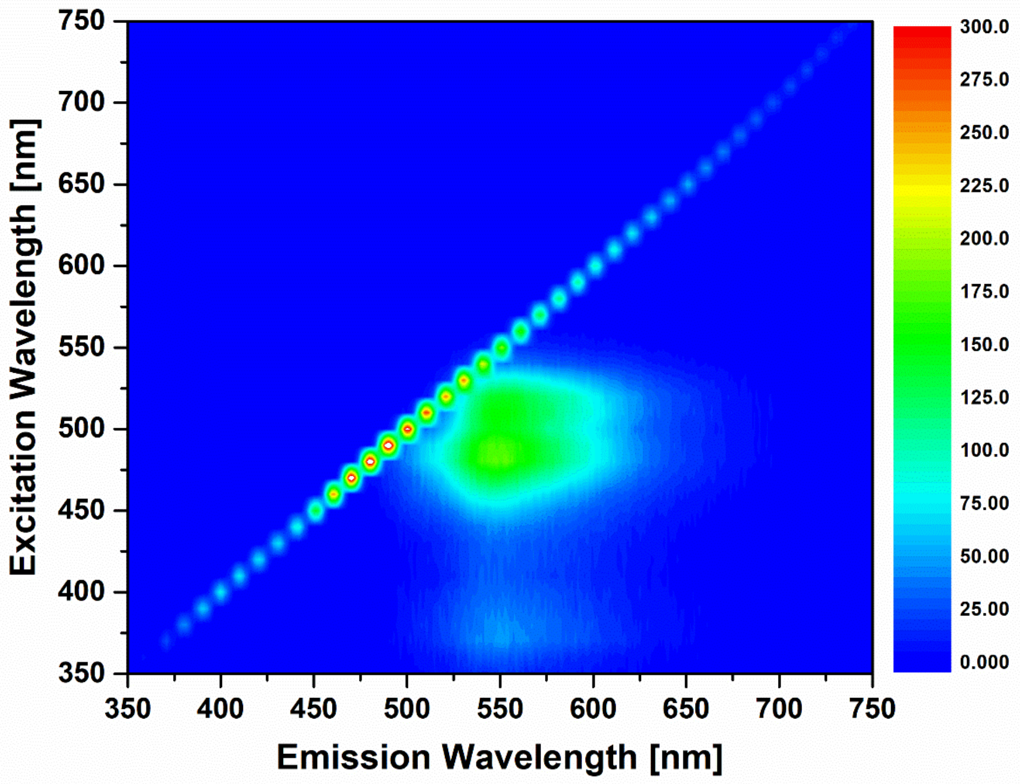

Figure S26

Figures S24 - S26: Excitation-Emission maps for compounds **4g**, **4g-Zn** and **4g-Ga** in concentration of 100 μM in DMSO. Experiment was performed by recording emission spectra corresponding to the excitation at wavelengths 10 nm increments apart between the 200 nm and 800 nm for **4g** and **4g-Zn** and between 350 and 750 nm for **4g-Ga**. Scans were performed over a period of approximately 4 hours, during which time the expected decomposition of **4g-Ga** to **4g** ligand likely occurred. Data was processed in Origin 9.0.

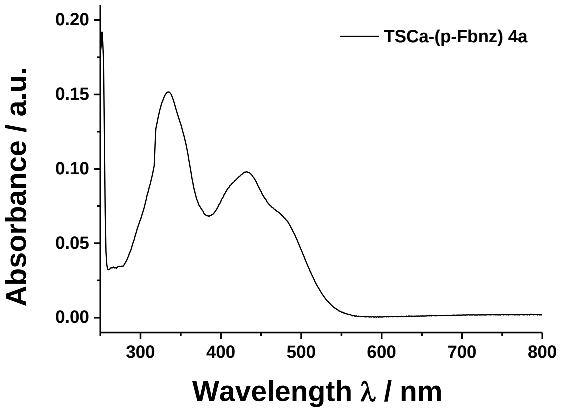

Figure S27 UV-vis absorption spectrum for compound **4a** 0.01mM in DMSO

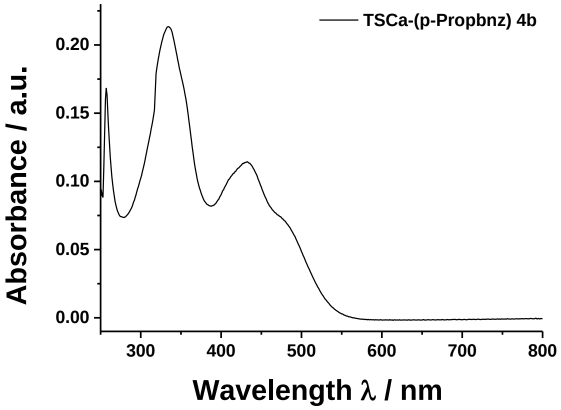

Figure S28 UV-vis absorption spectrum for compound **4b** 0.01mM in DMSO (left)

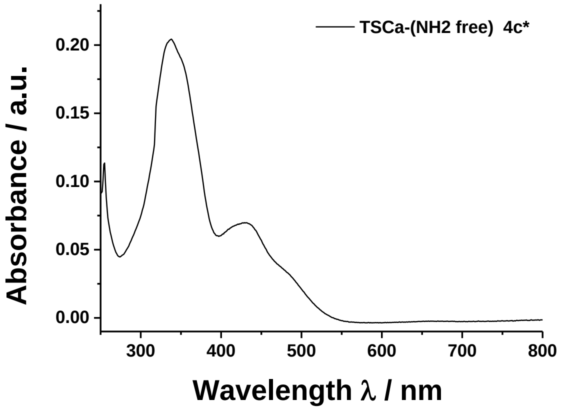

Figure S29 UV-vis absorption spectrum for compound **4c*** 0.01mM in DMSO (left)

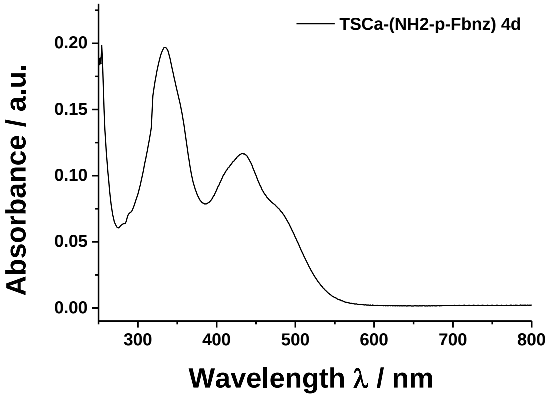

Figure S30 UV-vis absorption spectrum for compound **4d** 0.01mM in DMSO (left) and two-dimensional fluorescence contour plotting of **4d** 1mM in DMSO (right).

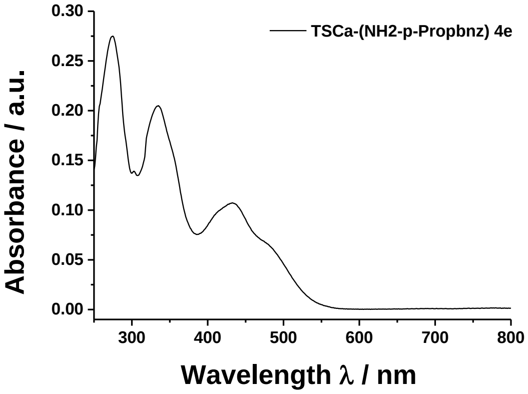

Figure S31 UV-vis absorption spectrum for compound **4e** 0.01mM in DMSO (left) and two-dimensional fluorescence contour plotting of **4e** 1mM in DMSO (right).

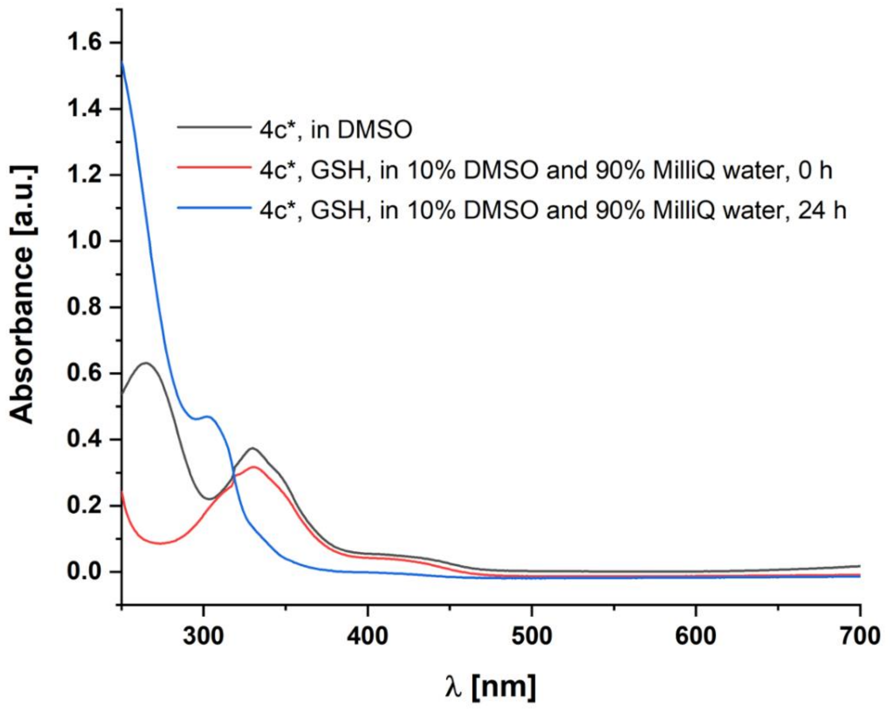

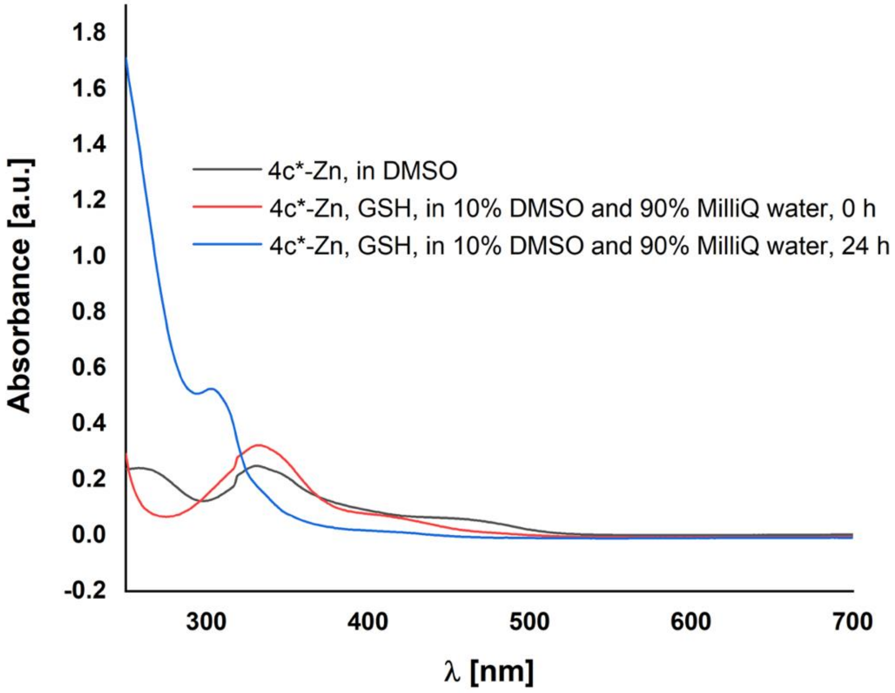

Figure S32. Spectroscopic stability assay (10% DMSO, 90% MilliQ water) for compound **4c* (top)** and **4C*-Zn (bottom)** at a concentration of 15 μM against glutathione (GHS, 10 mM) by UV/Vis spectra monitored at 0 h and 24 h at room temperature.

4. Cellular imaging and viability assays

4.1. Confocal fluorescence microscopy experiments

PC3 cells were cultured under normoxic conditions as previously described, and then seeded in a glass bottom dish at least 48 h prior to the microscopy experiment (10,0000 cell per well plate) and incubated at 37 ºC. Control fluorescence images were recorded before the addition of compound. For confocal microscopy, the compound in a DMSO: RPMI/SFM 0.5:99.5 solution mixture (50 μM), was loaded into the wells and cells were allowed to incubate for 15 min, at 37 °C. They were then carefully washed with PBS and then it was replaced by RPMI/SFM to remove the non-internalised fluorescent dispersion prior to fluorescence imaging.

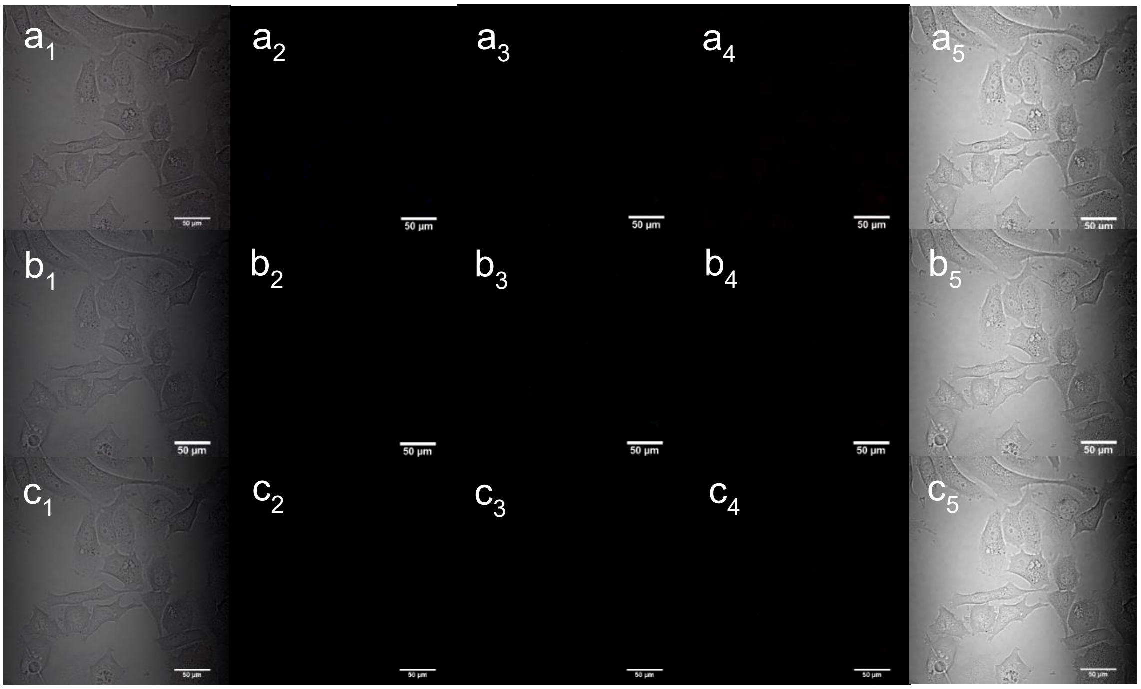

Figure S33 Single photon confocal microscopy images of the control of PC-3 cells at 37 °C after 20 min incubation (50 μM, in 10:990 DMSO:serum free medium), where a) λex = 405.0 nm, b) λex = 488.0 nm, c) λex = 561.0 nm, a_1, b_1 ,c_1) overlay of the channels, a_2, b_2 ,c_2) blue channel (λem = 417-477 nm), a_3, b_3 ,c_3) green channel (λem = 500-550 nm), a_4, b_4 ,c_4) red channel (λem = 570-750 nm, and a_5, b_5 ,c_5) DIC channel. (scale bar 20 μm).

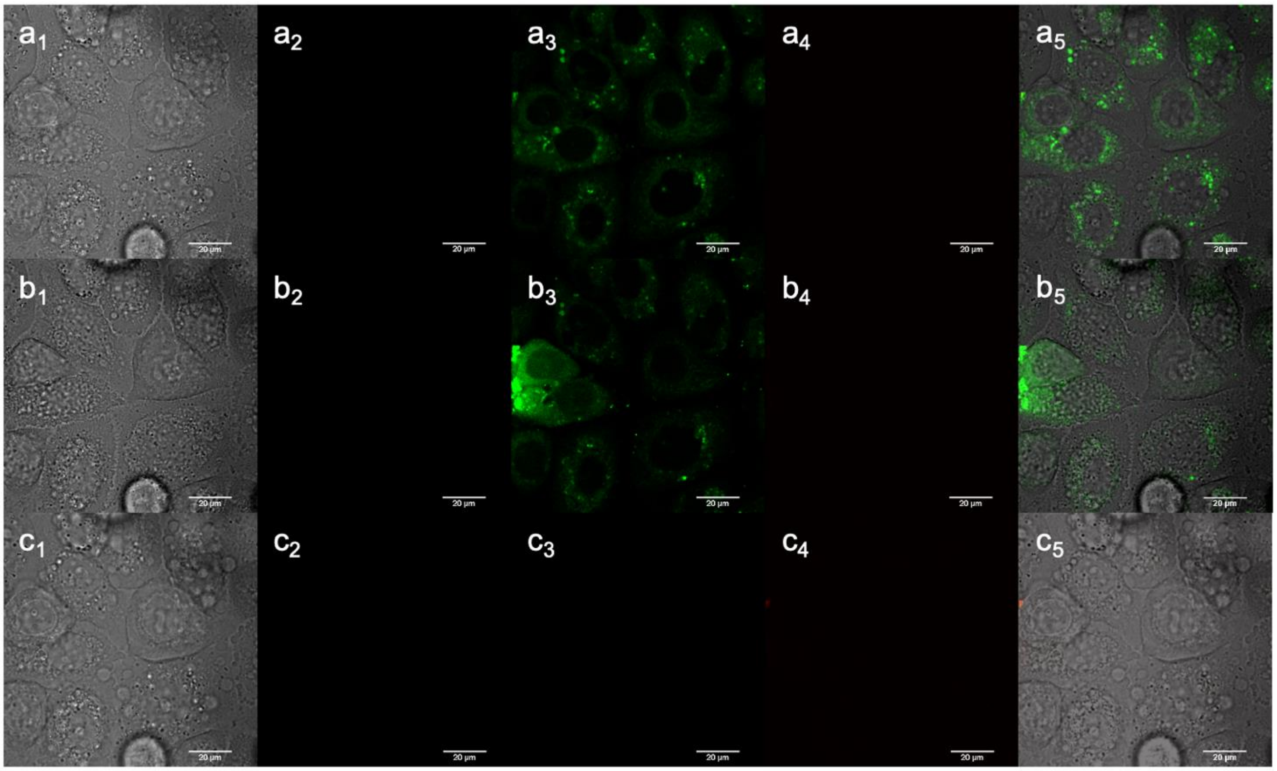

Figure S34 Single photon confocal microscopy images of TSCa-(p-Fbnz) ligand (**4a**) in PC-3 cells at 37 °C after 20 min incubation (50 μM,in 10:990 DMSO:serum free medium), where a) λex = 405 nm, b) λex = 488 nm, c) λex = 561 nm, a_1, b_1 ,c_1) DIC channels, a_2, b_2 ,c_2) blue channel (λem = 417-477 nm), a_3, b_3 ,c_3) green channel (λem = 500-550 nm), a_4, b_4 ,c_4) red channel (λem = 570-750 nm, and a_5, b_5 ,c_5) overlay of all channels. (scale bar 20 μm).

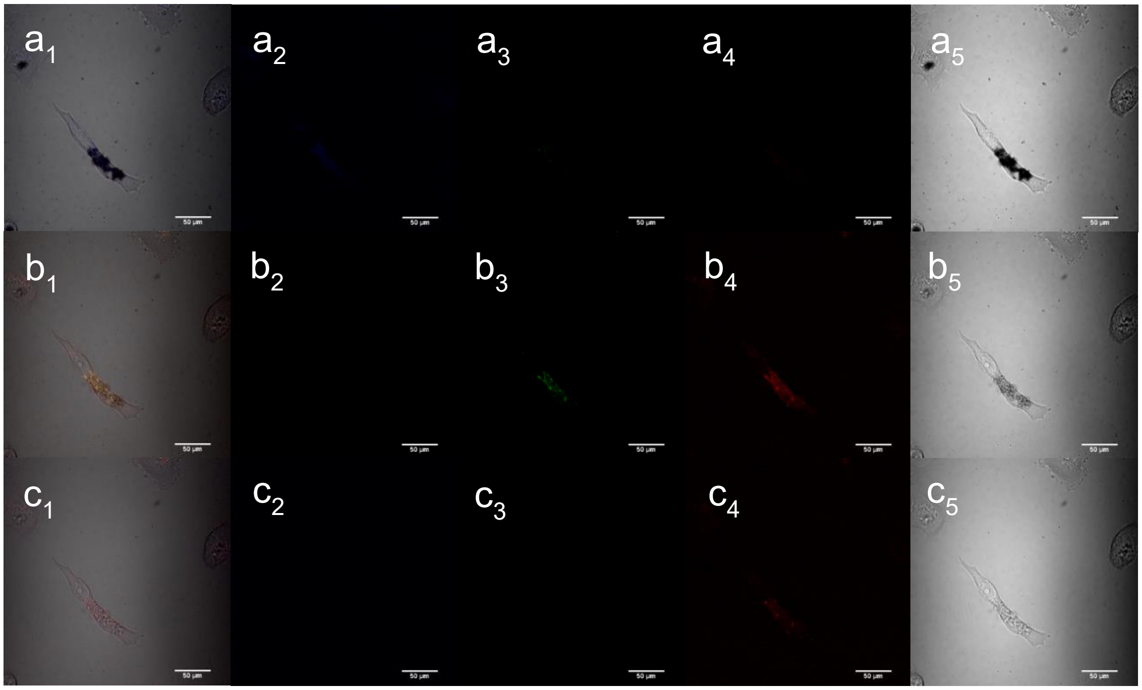

Figure S35 Single photon confocal microscopy images of TSCa-(p-Propbnz) ligand (**4b**) in PC-3 cells at 37 °C after 20 min incubation (50 μM,in 10:990 DMSO:serum free medium), where a) λex = 405.0 nm, b) λex = 488.0 nm, c) λex = 561.0 nm, a_1, b_1 ,c_1) overlay of the channels, a_2, b_2 ,c_2) blue channel (λem = 417-477 nm), a_3, b_3 ,c_3) green channel (λem = 500-550 nm), a_4, b_4 ,c_4) red channel (λem = 570-750 nm, and a_5, b_5 ,c_5) DIC channel. (scale bar 20 μm).

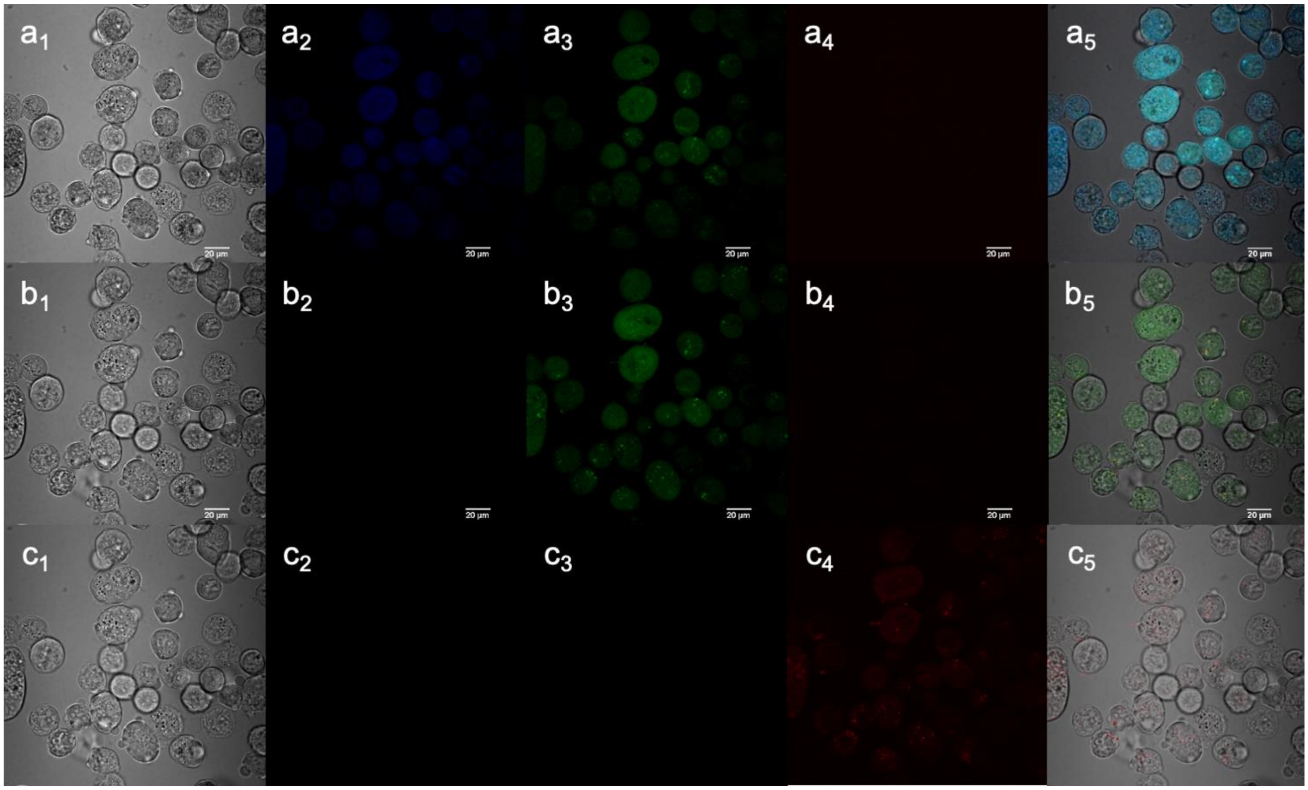

Figure S36 Single photon confocal microscopy images of TSCa-(NH_2 free) ligand (**4c***) in PC-3 cells at 37 °C after 20 min incubation (50 μM,in 10:990 DMSO:serum free medium), where a) λex = 405 nm, b) λex = 488 nm, c) λex = 561 nm, a_1, b_1 ,c_1) DIC channel, a_2, b_2 ,c_2) blue channel (λem = 417-477 nm), a_3, b_3 ,c_3) green channel (λem = 500-550 nm), a_4, b_4 ,c_4) red channel (λem = 570-750 nm, and a_5, b_5 ,c_5) overlay of all channels. (Scale bar 20 μm).

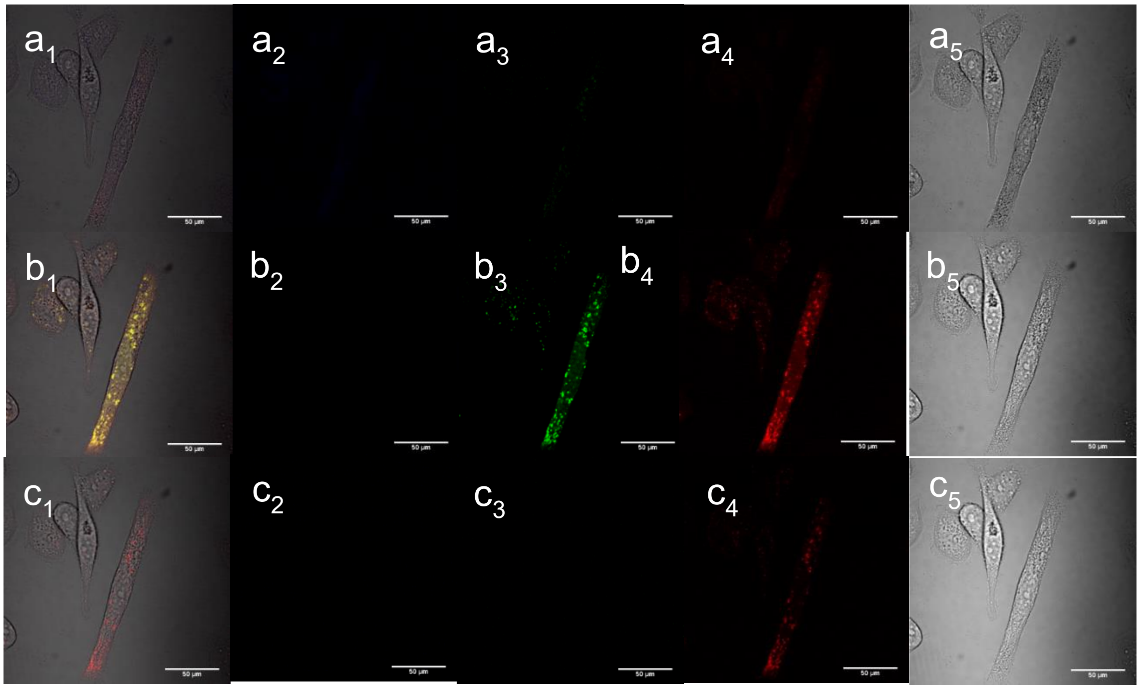

Figure S37 Single photon confocal microscopy images of TSCa-(NH_2-p-Fbnz) ligand (**4d**) in PC-3 cells at 37 °C after 20 min incubation (50 μM,in 10:990 DMSO:serum free medium), where a) λex = 405.0 nm, b) λex = 488.0 nm, c) λex = 561.0 nm, a_1, b_1 ,c_1) overlay of the channels, a_2, b_2 ,c_2) blue channel (λem = 417-477 nm), a_3, b_3 ,c_3) green channel (λem = 500-550 nm), a_4, b_4 ,c_4) red channel (λem = 570-750 nm, and a_5, b_5 ,c_5) DIC channel. (scale bar 20 μm).

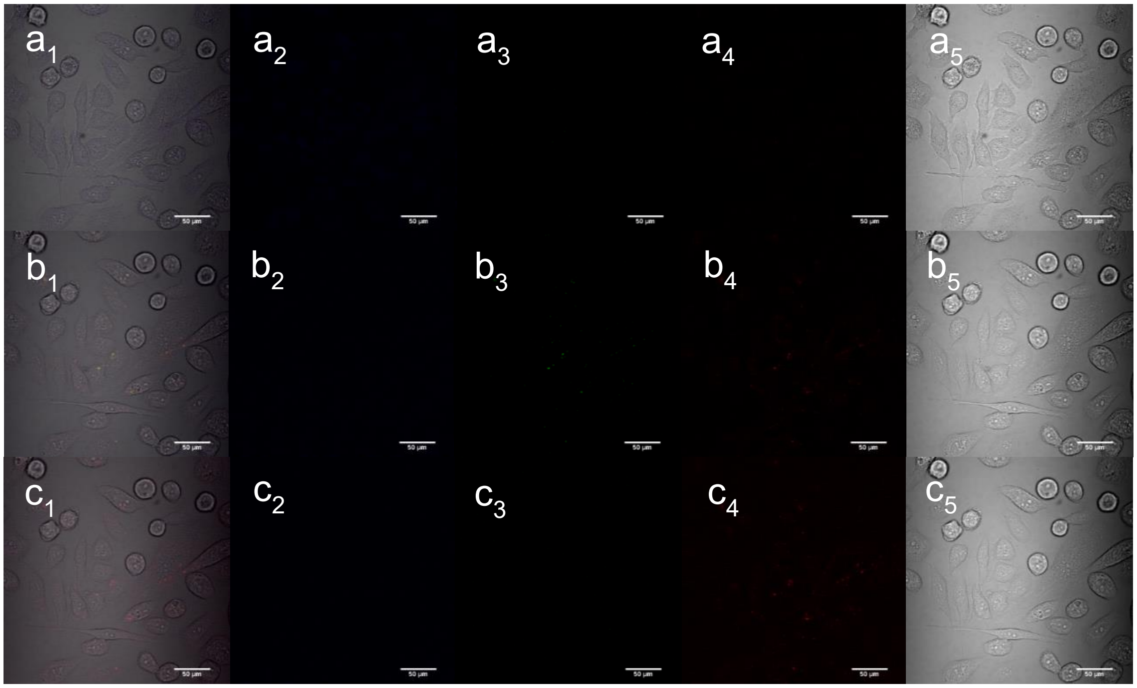

Figure S38 Single photon confocal microscopy images of TSCa-(NH_2-p-Propbnz) ligand (**4e**) in PC-3 cells at 37 °C after 20 min incubation (50 μM,in 10:990 DMSO:serum free medium), where a) λex = 405.0 nm, b) λex = 488.0 nm, c) λex = 561.0 nm, a_1, b_1 ,c_1) overlay of the channels, a_2, b_2 ,c_2) blue channel (λem = 417-477 nm), a_3, b_3 ,c_3) green channel (λem = 500-550 nm), a_4, b_4 ,c_4) red channel (λem = 570-750 nm, and a_5, b_5 ,c_5) DIC channel. (scale bar 20 μm).

4.2. MTT assays

Cells (5-7×10^3 cells per well) were seeded on a sterile 96-well plate and incubated for 48 h to adhere. All the TSCa mono(substituted) ligands were subsequently loaded at different concentration into wells and cultured for another 48 h. Subsequently, cells were washed three times with PBS and 3-(4, 5-dimethylthiazol-2-yl)-2, 5-diphenyltetrazolium bromide (MTT) was added (0.5 mg/ mL, 90% serum-free medium (SFM)) + 10% PBS followed by a two-hour incubation. After aspiration, 100 µL of DMSO was added and the 96-well plates were read by an ELISA plate reader, Molecular Devices Versa Max (BN02877). The absorption wavelength was at 570 nm and 630 nm wavelength was used as a reference. For establishing the correct protocols, a library of known compounds with similar solubility and which also carries the N/S motifs were analysed.

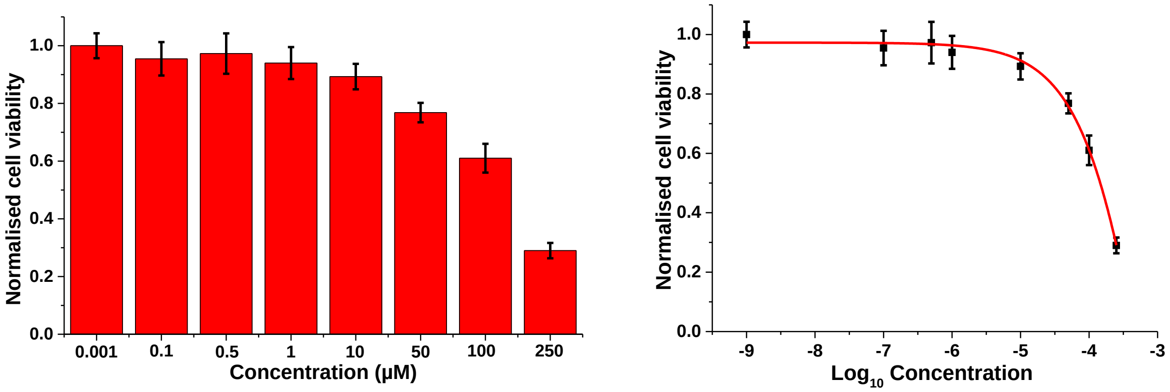

Figure S39 Normalised cell viability test of PC3 cells treated with compound **4a** incubated for 24 h under normoxia (left); curve of cell viability (right). Error bars stands for standard error calculated from the six repeats.

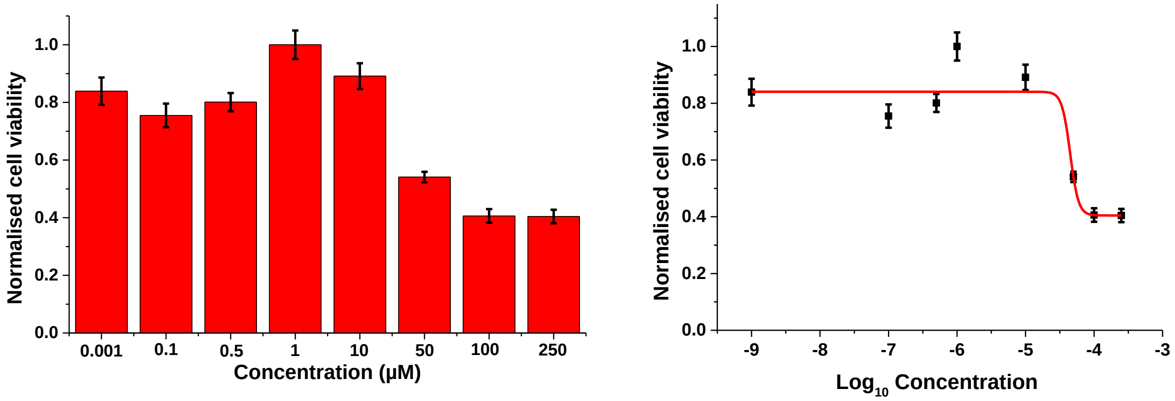

Figure S40 Normalised cell viability test of PC3 cells treated with compound **4a** incubated for 48 h under normoxia (left); curve of cell viability (right). Error bars stands for standard error calculated from the six repeats.

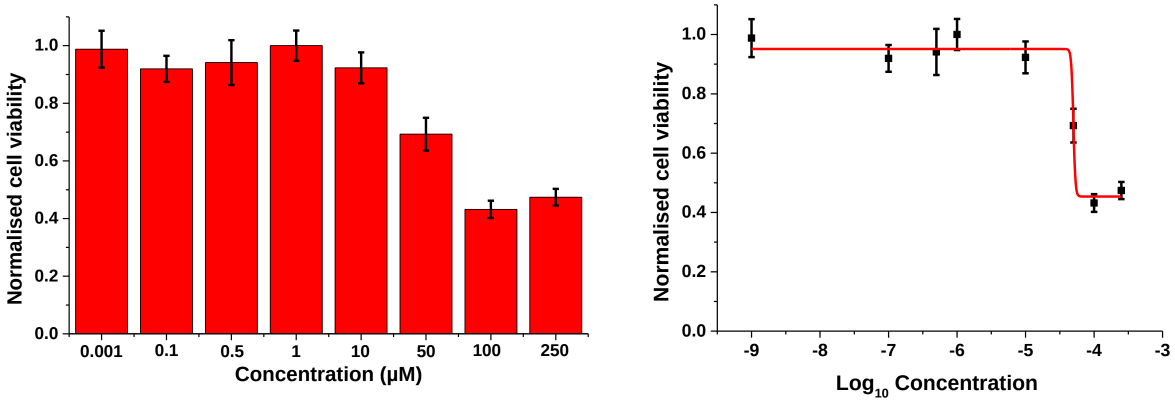

Figure S41 Normalised cell viability test of PC3 cells treated with compound **4a** incubated for 72 h under normoxia (left); curve of cell viability (right). Error bars stands for standard error calculated from the six repeats.

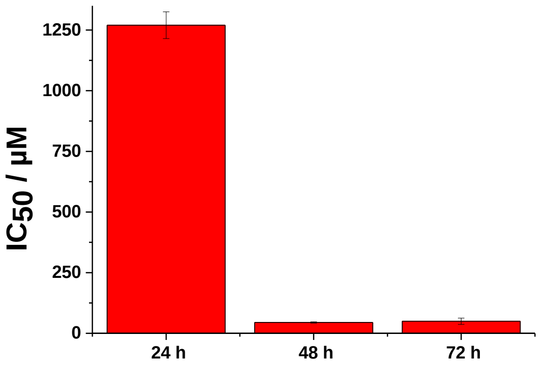

Figure S42 IC_{50} in PC-3 cells after 24, 48 and 72 hours treatment with compound 4a.

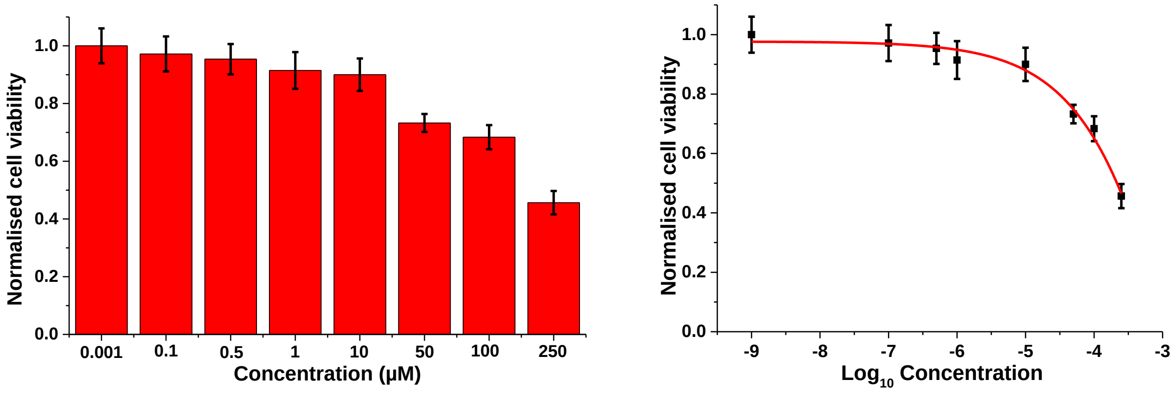

Figure S43 Normalised cell viability test of PC3 cells treated with compound **4b** incubated for 24 h under normoxia (left); curve of cell viability (right). Error bars stands for standard error calculated from the six repeats.

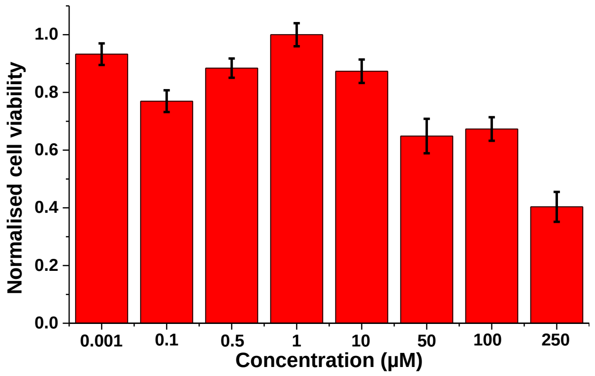

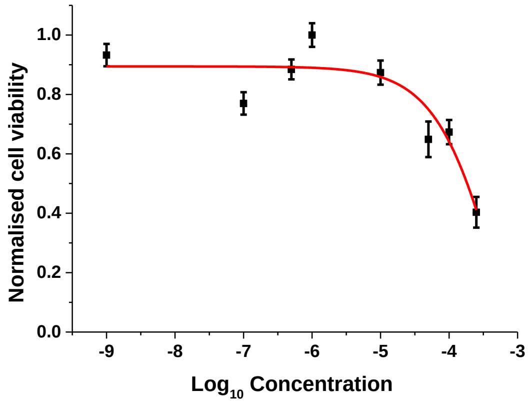

Figure S44 Normalised cell viability test of PC3 cells treated with compound **4b** incubated for 48 h under normoxia (left); curve of cell viability (right). Error bars stands for standard error calculated from the six repeats.

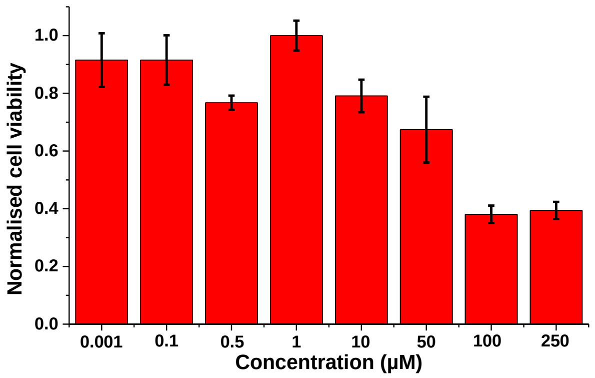

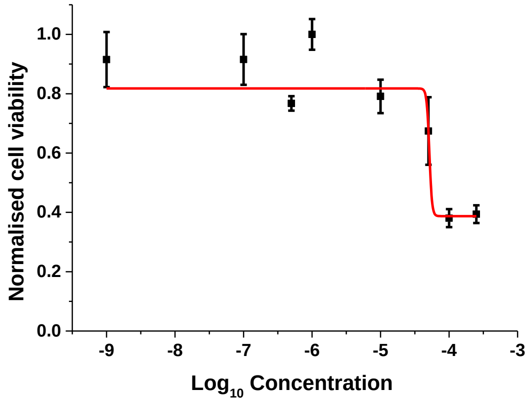

Figure S45 Normalised cell viability test of PC3 cells treated with compound **4b** incubated for 72 h under normoxia (left); curve of cell viability (right). Error bars stands for standard error calculated from the six repeats.

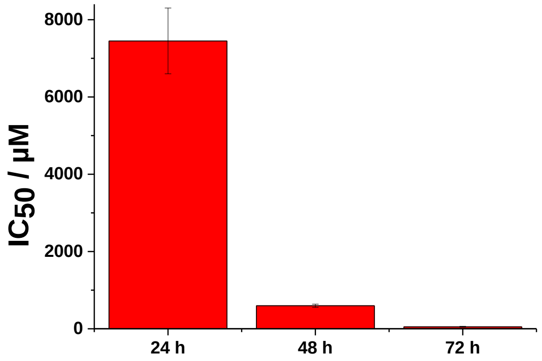

Figure S46 IC_{50} in PC-3 cells after 24, 48 and 72 hours treatment with compound 4b.

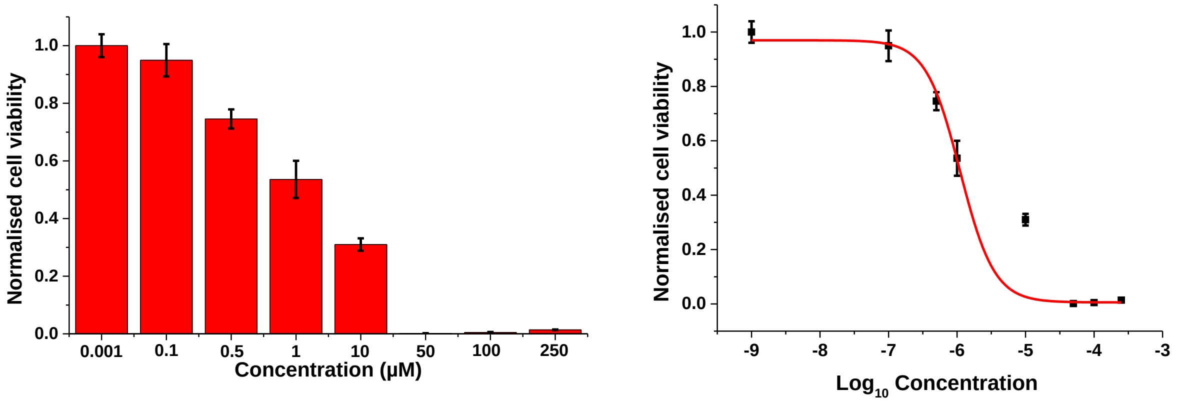

Figure S47 Normalised cell viability test of PC3 cells treated with compound **4c*** incubated for 24 h under normoxia (left); curve of cell viability (right). Error bars stands for standard error calculated from the six repeats.

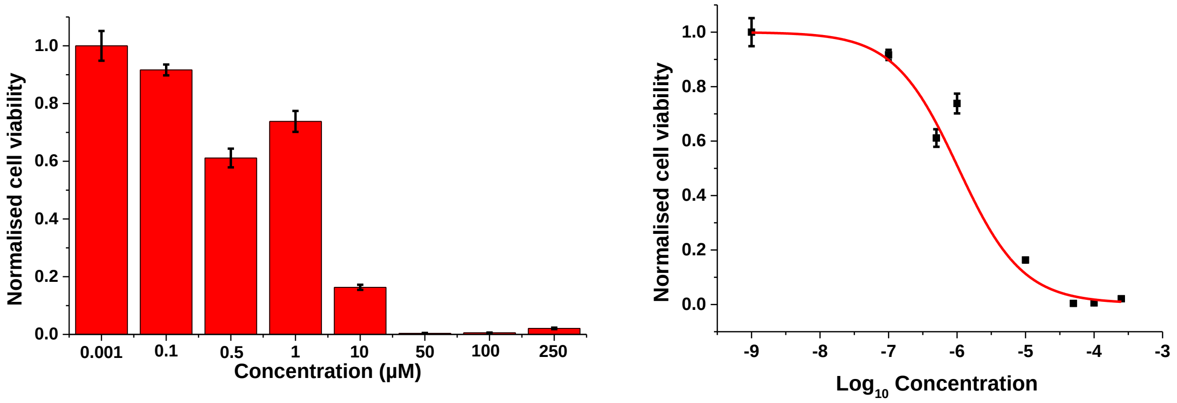

Figure S48 Normalised cell viability test of PC3 cells treated with compound **4c*** incubated for 48 h under normoxia (left); curve of cell viability (right). Error bars stands for standard error calculated from the six repeats.

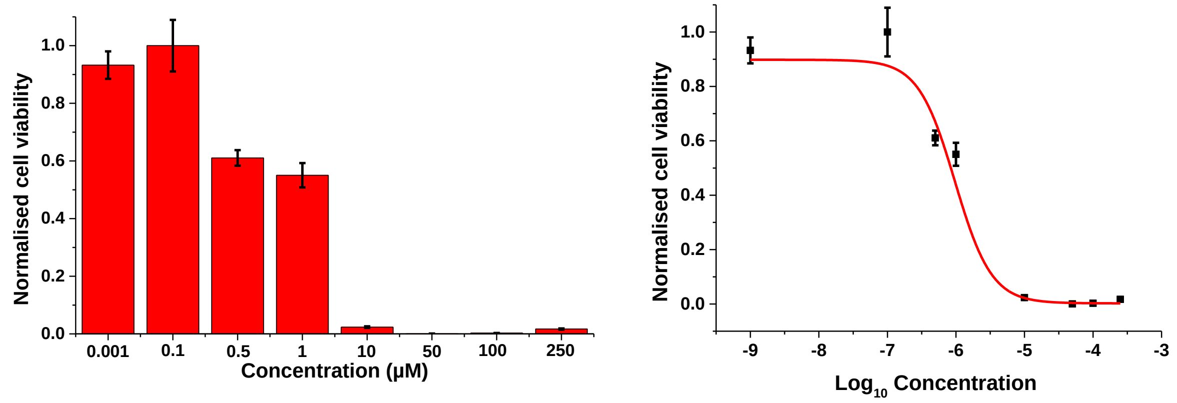

Figure S49 Normalised cell viability test of PC3 cells treated with compound **4c*** incubated for 72 h under normoxia (left); curve of cell viability (right). Error bars stands for standard error calculated from the six repeats.

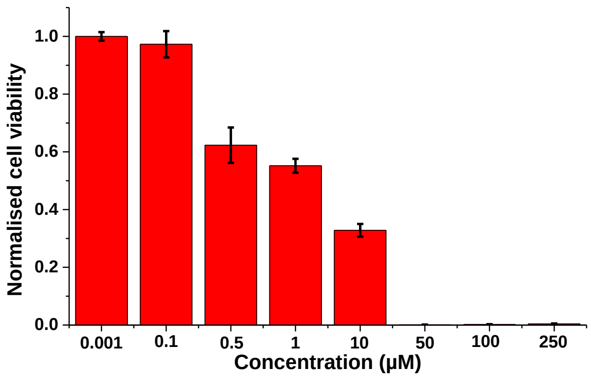

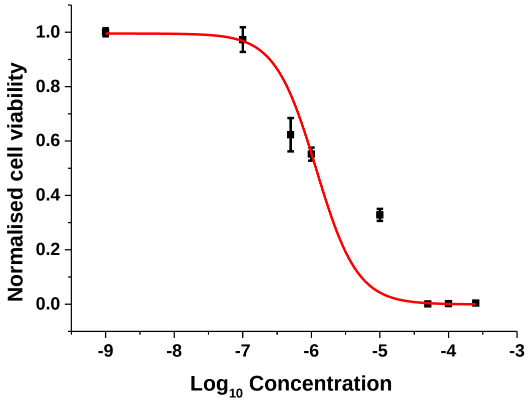

Figure S50 Normalised cell viability test of PC3 cells treated with compound **4d** incubated for 24 h under normoxia (left); curve of cell viability (right). Error bars stands for standard error calculated from the six repeats.

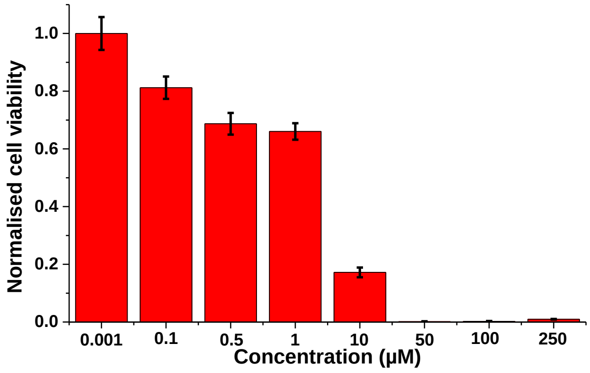

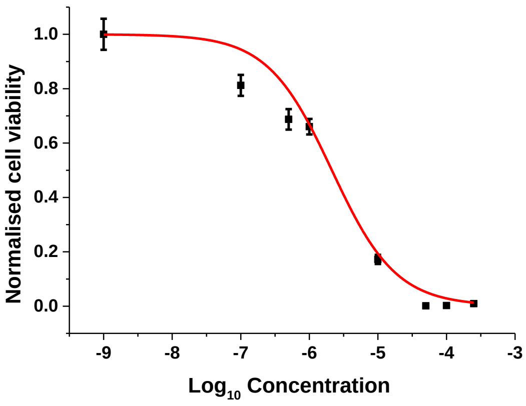

Figure S51 Normalised cell viability test of PC3 cells treated with compound **4d** incubated for 48 h under normoxia (left); curve of cell viability (right). Error bars stands for standard error calculated from the six repeats.

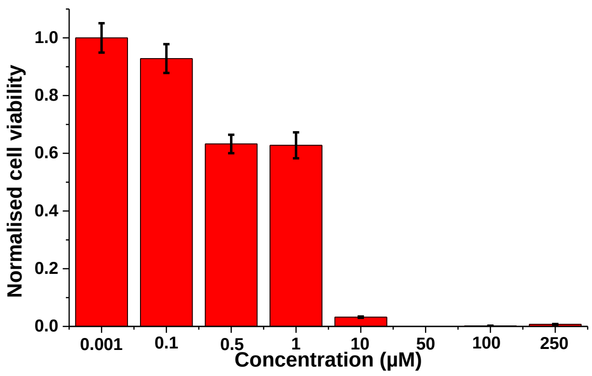

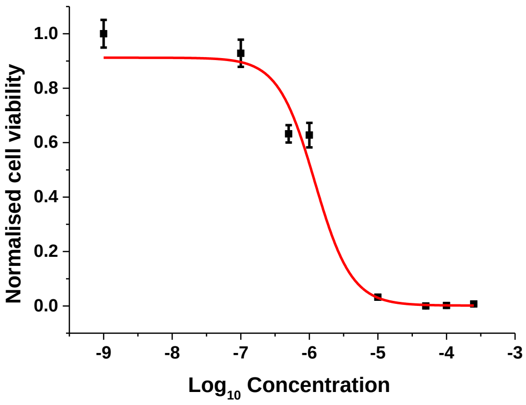

Figure S52 Normalised cell viability test of PC3 cells treated with compound **4d** incubated for 72 h under normoxia (left); curve of cell viability (right). Error bars stands for standard error calculated from the six repeats.

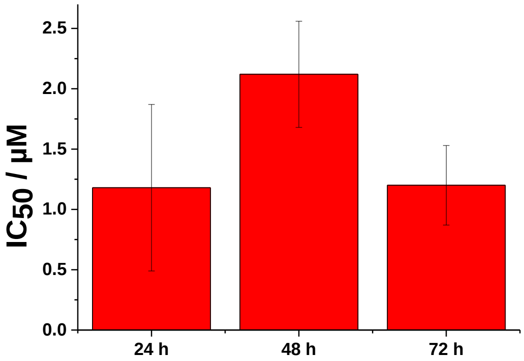

Figure S53 IC_{50} in PC-3 cells after 24, 48 and 72 hours treatment with compound 4d.

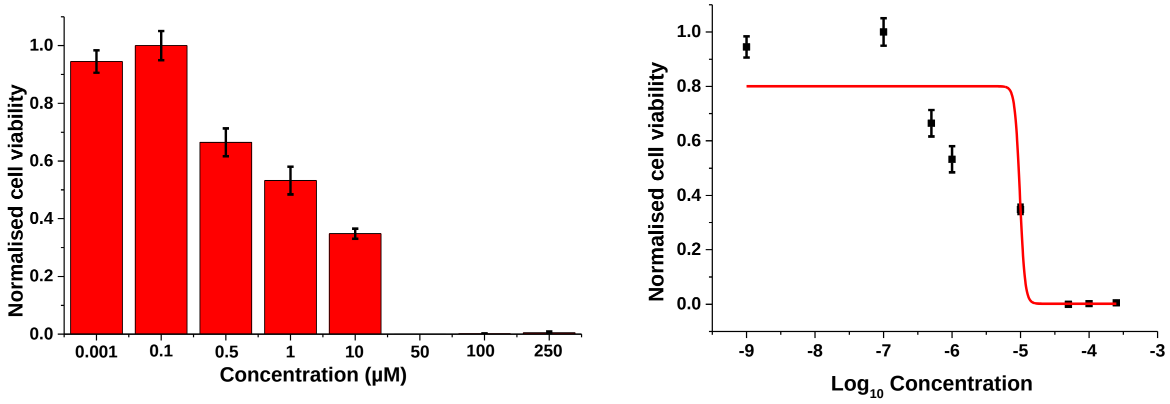

Figure S54 Normalised cell viability test of PC3 cells treated with compound **4e** incubated for 24 h under normoxia (left); curve of cell viability (right). Error bars stands for standard error calculated from the six repeats.

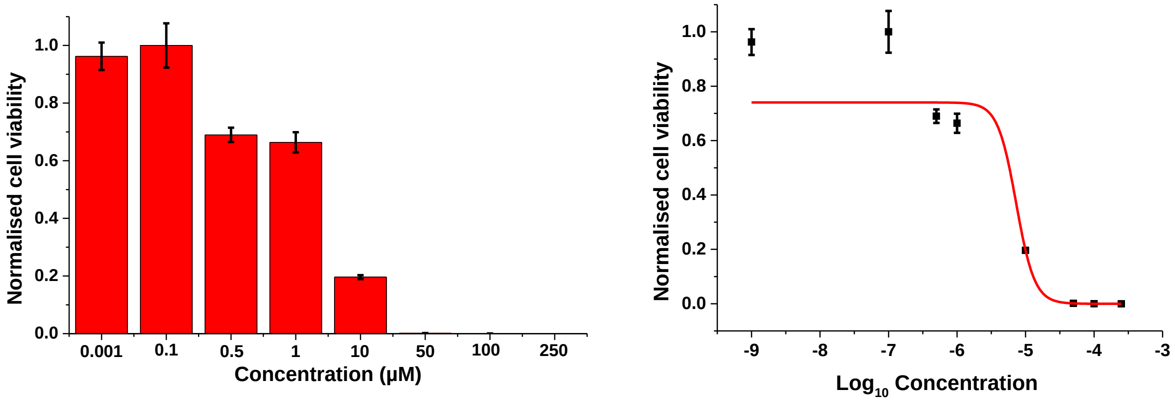

Figure S55 Normalised cell viability test of PC3 cells treated with compound **4e** incubated for 48 h under normoxia (left); curve of cell viability (right). Error bars stands for standard error calculated from the six repeats.

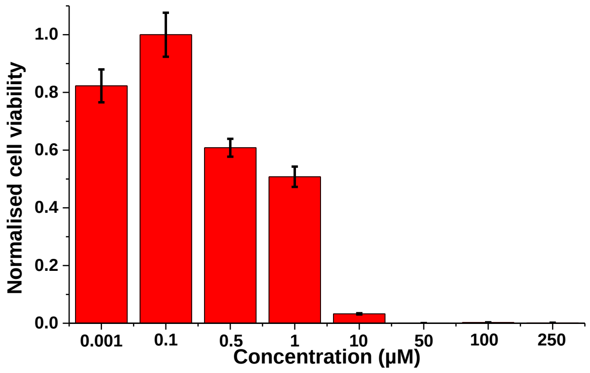

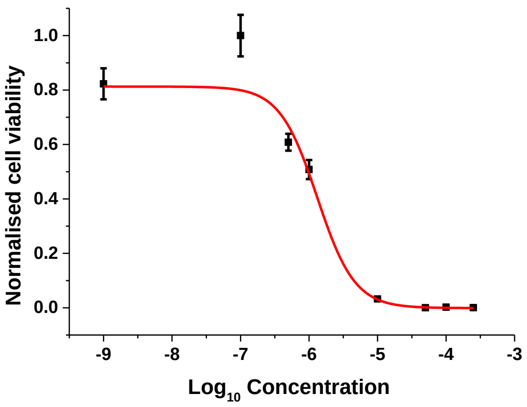

Figure S56 Normalised cell viability test of PC3 cells treated with compound **4e** incubated for 72 h under normoxia (left); curve of cell viability (right). Error bars stands for standard error calculated from the six repeats.

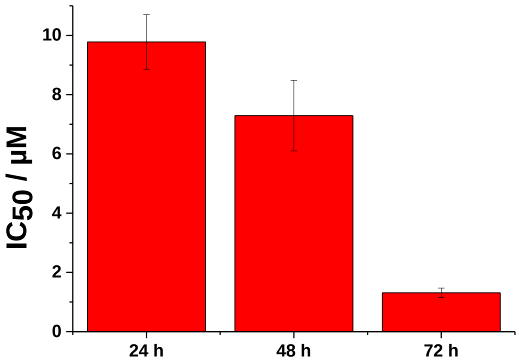

Figure S57 IC_{50} in PC-3 cells after 24, 48 and 72 hours treatment with compound **4e.**

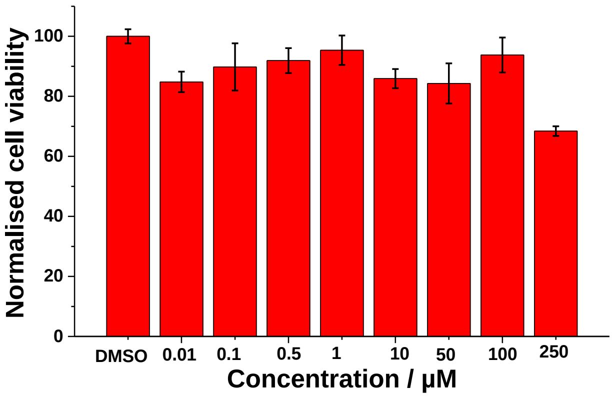

Figure S58 Normalised cell viability test of EMT6 cells treated with compound **4a** (TSCa-(p-Fbnz)) under normoxic condition (left); Experiments were repeated six times and error bars stands for standard error calculated from the repeated measurements.

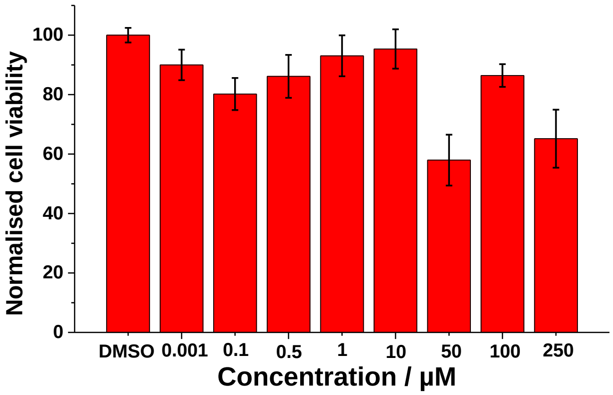

Figure S59 Normalised cell viability test of EMT6 cells treated with compound **4a** (TSCa-(p-Fbnz)) under hypoxic condition (left); Experiments were repeated six times and error bars stands for standard error calculated from the repeated measurements.

5. **Radiochemistry assays**

5.1. General synthetic procedure for the synthesis of the Gallium-68 acenaphthenequinone thiosemicarbazones complexes

A SnO2-based column matrix $^{68}Ge/^{68}Ga$ generator was used to elute 10 mL of 0.6 M HCl, ca. 4 mCi of ^{68}Ga, which was trapped on a strata x-c 33 µm Polymeric Strong Cation Cartridge from Phenomenex and eluted with 700 µL of 0.02M HCl (98% THF). This was subsequently dried for 7-10 min under a nitrogen stream at 95°C. Next 30 µL of the monosubstituted compound in dry DMSO (2mg/mL) was added along with 0.6 mL of injectable MeOH and the pH adjusted to range between 5-7. This was heated under microwave radiation at 95 °C for 30 min. Analysis by reverse-phase HPLC (Method B) gave two different retention times for each compound which in comparison with the HPLC trace of the precursors suggest that this could be isomers of the compound. Remains of the $[^{68}Ga]GaCl_3$ were indicate that radiolabelling of the mono(substituted) ligands had not gone to completion. It was found that if reactions were carried out at pH ca 1-4 only very limited conversions were achieved, either by microwave irradiation or by conventional heating, even under prolonged reaction times (60 min -120 min).

5.2. UV- and radio-HPLC traces of the mono(thiosemicarbazones) ligands and corresponding Gallium-68 complexes

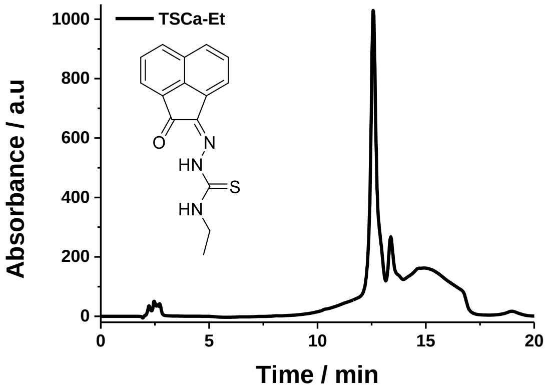

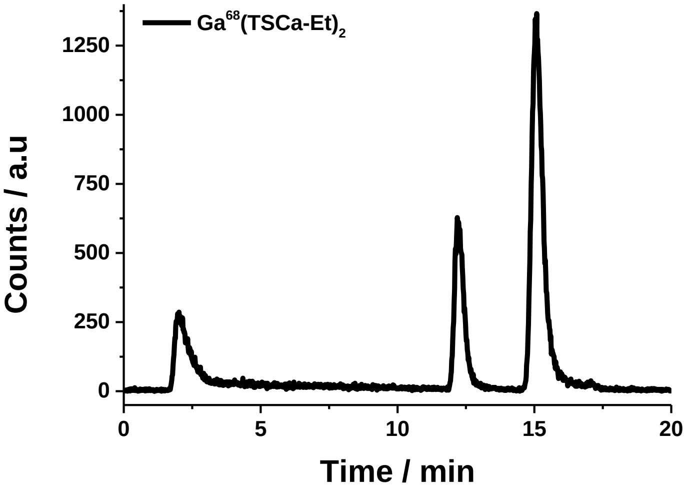

Figure S60 UV-HPLC chromatogram of the TSCa-Et ligand **4f** (top) and radio-HPLC chromatogram of the radiolabelling Gallium-68 TSCa-Et complex (bottom).

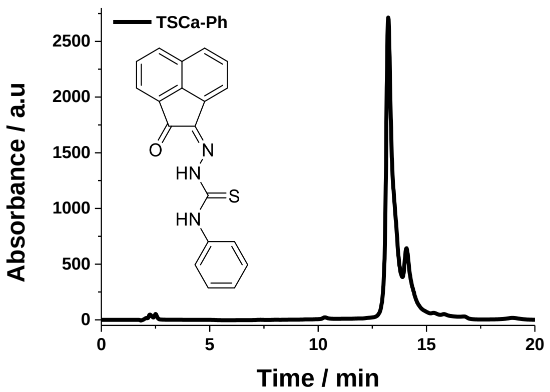

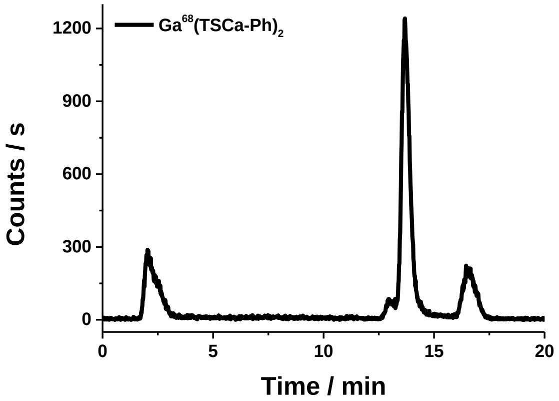

Figure S61 UV-HPLC chromatogram of the TSCa-Ph **4h** ligand (left) and radio-HPLC chromatogram of the radiolabelling gallium-68 TSCa-Ph complex (right). pH 6.8

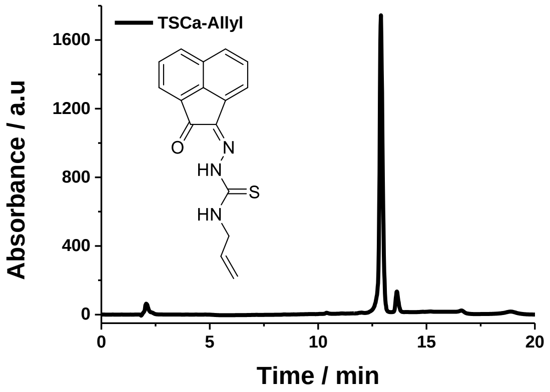

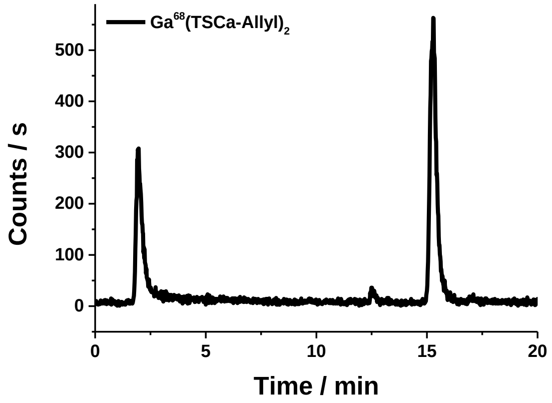

Figure S62 UV-HPLC chromatogram of the TSCa-Allyl ligand **4g** (left) and radio-HPLC chromatogram of the radiolabelling gallium-68 TSCa-Allyl complex (right). pH 6.8

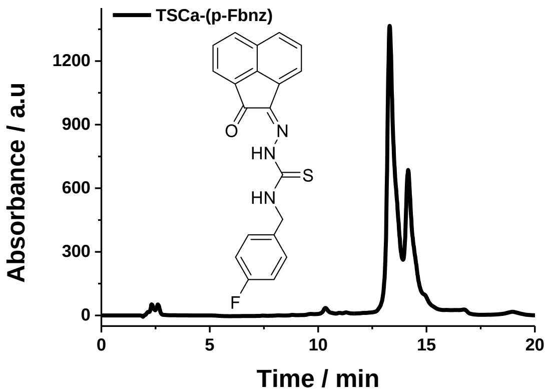

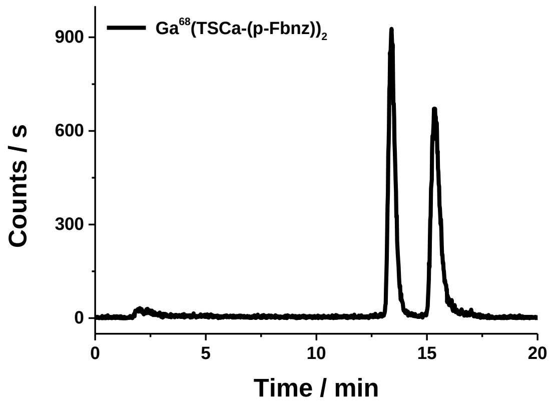

Figure S63 UV-HPLC chromatogram of the TSCa-(p-Fbnz) ligand **4a** (left) and radio-HPLC chromatogram of the radiolabelling gallium-68 TSCa-(p-Fbnz) complex (right). pH 6.8

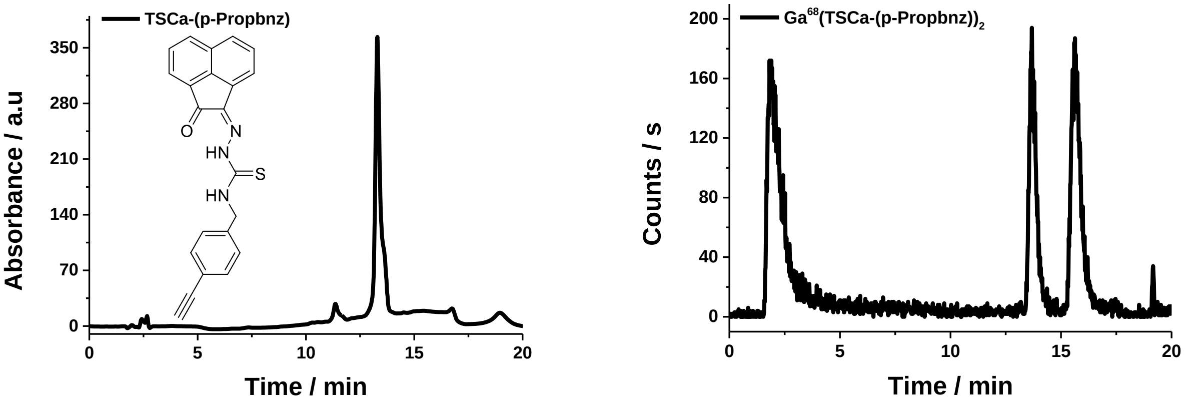

Figure S64 UV-HPLC chromatogram of the TSCa-(p-Propbnz) ligand **4b** (left) and radio-HPLC chromatogram of the gallium-68 TSCa-(p-Propbnz) complex mixture (right). Optimised reaction, pH 6.8.

Radio Thin Layer Chromatography:

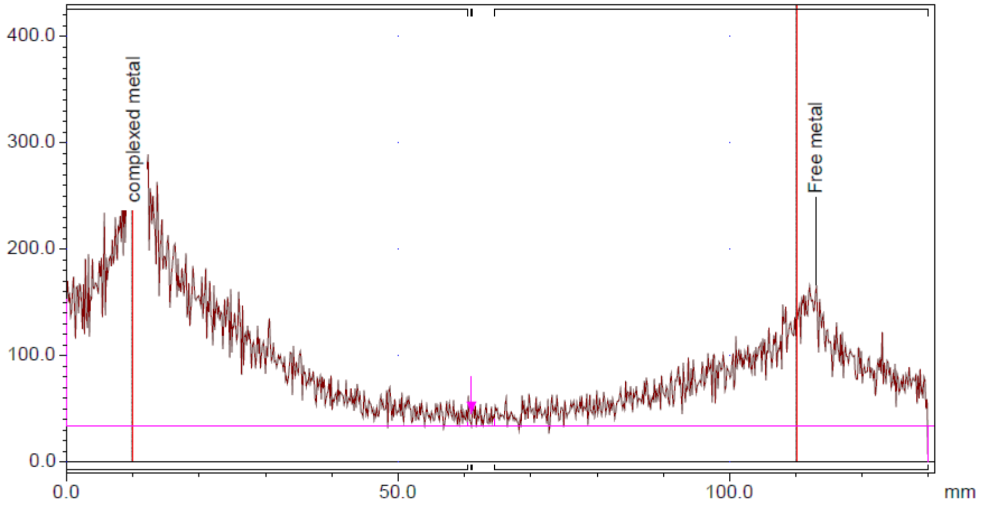

Name	Start (mm)	End (mm)	Retention (RF)	Area (Counts)	%ROI (%)	%Total (%)
complexed metal	0.0	60.5	0.003	34182.0	63.88	63.68
BP 1	61.1	61.2	0.511			
Free metal	64.5	129.9	1.030	19325.0	36.12	36.00
2 Peaks				53507.0	100.00	99.68

Total Area: 53681.0 Counts
Average Background: 34.0 Counts

Figure S65 Radio TLC of crude **[^{68}Ga]-4a** ([^{68}Ga]Ga(FbnzTSCA)$_2$), developed on Whatman 3 MM with 0.35 M ethylenediaminetetraacetic acid (EDTA) as the mobile phase. The radio-TLC shows formation of 36 % of free [^{68}Ga]GaCl$_3$. This TLC trace was obtained after a conventional heating radiolabelling reaction.

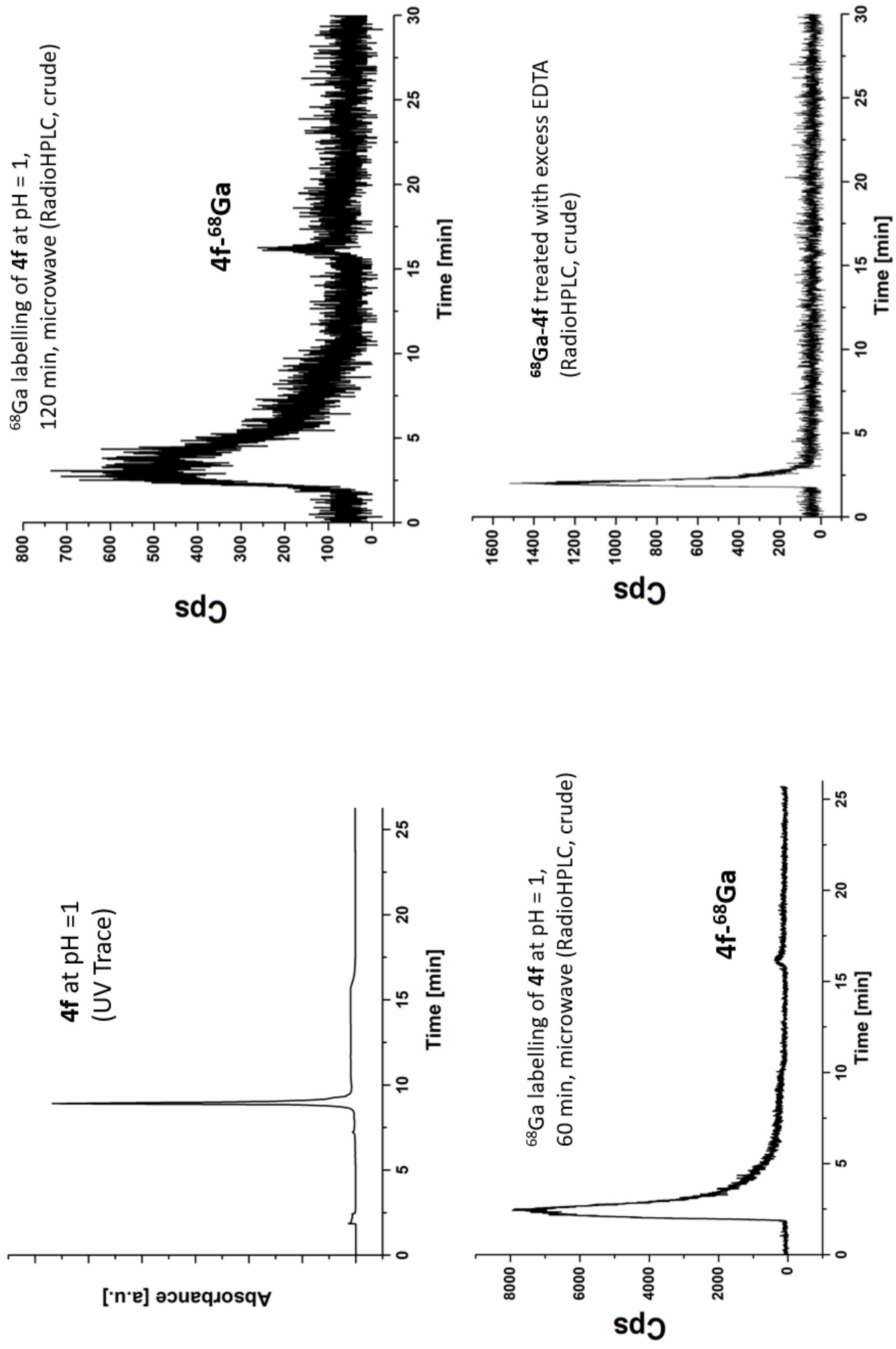

Figure S66 UV-HPLC chromatogram of compound 4f at pH1 ligand and radio-HPLC chromatograms of the attempted radiolabelling with Ga-68 at pH =1. Kinetic stability challenge with EDTA showing decomposition of **Ga-68-4f.**

5.3. Synthesis of the [(2-(4-^{18}Ffluorobenzylidene)aminoethyl)-3-thiosemicarbazone) acenaphthenequinone]

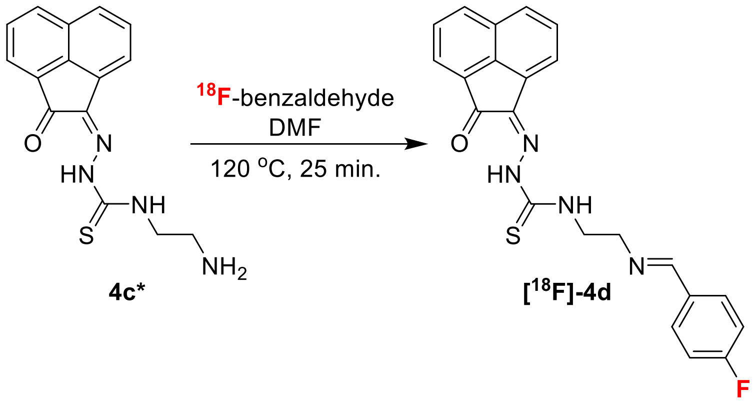

Scheme S5 Synthesis of 2-(4-[^{18}F]fluorobenzylidene)aminoethyl)-3-thiosemicarbazone acenaphthenequinone ligand via a coupling reaction.

In a sealed reaction vial, compound **4c*** (1.20 mg, 0.0042 mmol) was diluted in 0.5 mL of DMF and mixed with 25 μL of the SPE purified solution of compound 4-[^{18}F]fluorobenzaldehyde in MeCN (5.44 MBq). The mixture was heated to 120 °C for 25 min. Analysis by reverse-phase HPLC (**Method C**) gave a retention time of 7.08 min which in comparison with the reference HPLC trace suggested to confirm the synthesis of desirable radiolabelled compound ([^{18}F]-**4c**). The extent of conversion to the product was measured as 30 %.

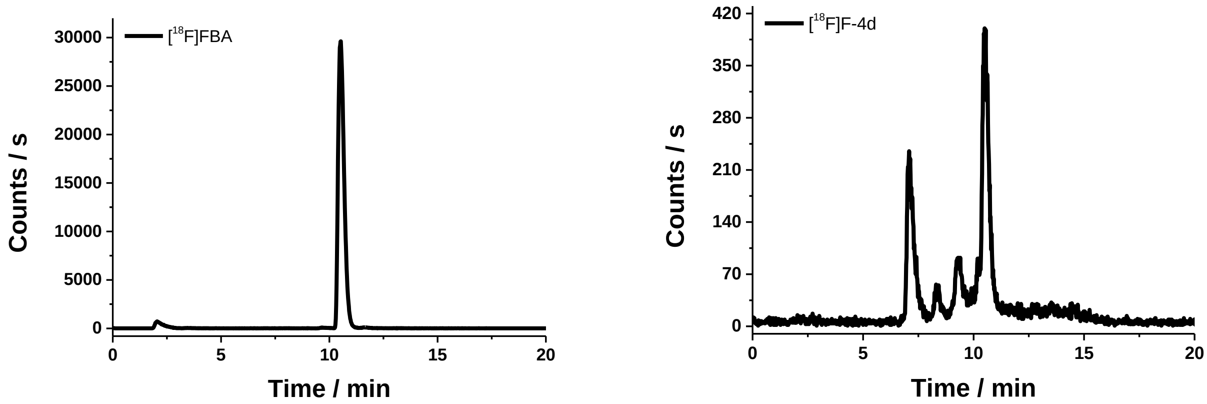

Figure S68 Radio-HPLC traces for radiolabelling of 4-[^{18}F]fluorobenzaldehyde (left) and the 2-(4-[^{18}F]fluorobenzylidene)aminoethyl)-3-thiosemicarbazone acenaphthenequinone ligand ([^{18}F]-**4d**) (right).

6. Cellular uptake of the Gallium-68 radiolabeled compound 4a

PC-3 and EMT-6 cells ($3 \cdot 10^3$ cells) were seeded in 6-well plates and incubated in normoxia and hypoxia environments as described before. After treatments, plates were aspirated and washed twice with warm PBS buffer. Each well plate was then loaded with 1000 µL of Gallium-68 complex in DMSO:PBS solution mixture (0.5:95.5) (3 MBq/ mL) and incubated for 1 h. After incubation, the reaction was stopped by washing wells with ice-cold PBS buffer twice, followed by addition of 1 mL of ice-cold, 0.1 % Triton X-100 and 0.1 M NaOH Lysates. A homogenous mixture was obtained by blending the components with up/down pipetting. 800 µL of each dissolved cell was then transferred and capped into counting tubes for gamma counting. The stock of the gallium-68 complex solution was aliquoted in 3 of 10µL dose and placed to the counting tubes as standards. The intracellular radioactivity was immediately counted using an LKB Wallac 1282 Compugamma Laboratory gamma counter (PerkinElmer, USA). Lastly, protein concentration determination was carried out by BCA assays. The cells were counted at a Sunrise absorbance reader (Tecan Trading AG, Switzerland). This normalisation of decay-corrected radioactivity counts per minute (CPM) to protein concentration, was required in order to give a measure of radiotracer uptake as % ID/mg of protein= CPM in 1 mL/ (standard in mL · protein concentration in mg) · 100 %. All the obtained results were analysed through the scientific 2D graphics and statistics software GraphPad Prism (GraphPad Software, California).

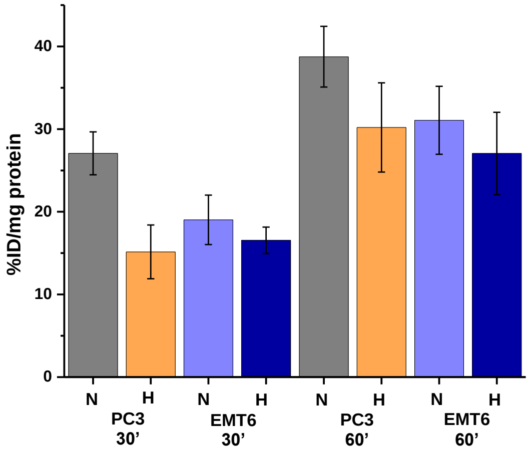

Figure S69. [^{68}Ga]Ga(4-fluorobenzyl)-3-thiosemicarbazonato acenaphthenequinone)$_2$ complex retention in PC3 and EMT6 cells under normoxic (N) and hypoxic (H) conditions measured at 30 and 60 minutes post addition of tracer and expressed in % of total tracer/mg of protein. Error bar stands for standard error (±SE), calculated from six repeated measurements.

7. Investigations into the formation and nature of 'cold' Gallium (III) complexes

Formation of a Ga(III) (F-benzyl thiosemicarbazonate) acenaphthenequinone complex using a microwave protocol either directly, from the free ligand **4a** or through the intermediate formation of the Zn(II) precursor (denoted **4a-Zn**) was performed. However only the formation of an intractable mixture of species was observed and the desired compound with proposed structure as shown below (denoted 6a, or 4a-Ga) could not be isolated in a pure form by semi-prep HPLC. ^{19}F NMR spectroscopy $^{19}F\{^1H\}$ NMR (DMSO-d_6, 25 ºC): showed broad resonance at -69 ppm and -120 ppm, additionally to the singlet peak characteristic to a **4a-type** fluorine resonance (assignable to the free ligand **4a** or Zn-bound **4a**) at -118 ppm.

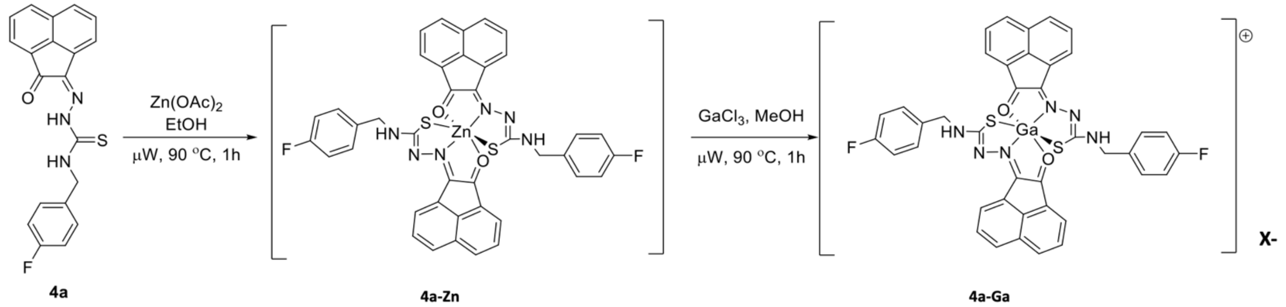

(X = Cl-, or OH- or MeO-)

Scheme S6

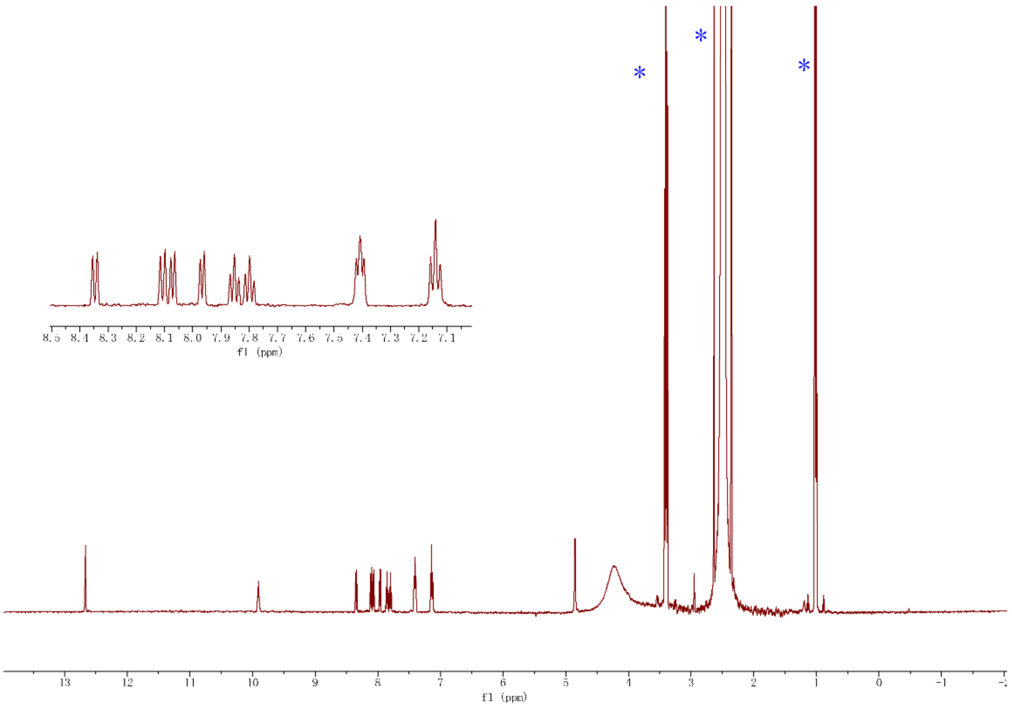

Figure S70 ^1H NMR spectrum (500 MHz, 298 K, DMSO-d_6) of **4a-Ga** which was synthesised by transmetallation. (* correspond to the residual solvents, DMSO-d_6 and Et_2O).

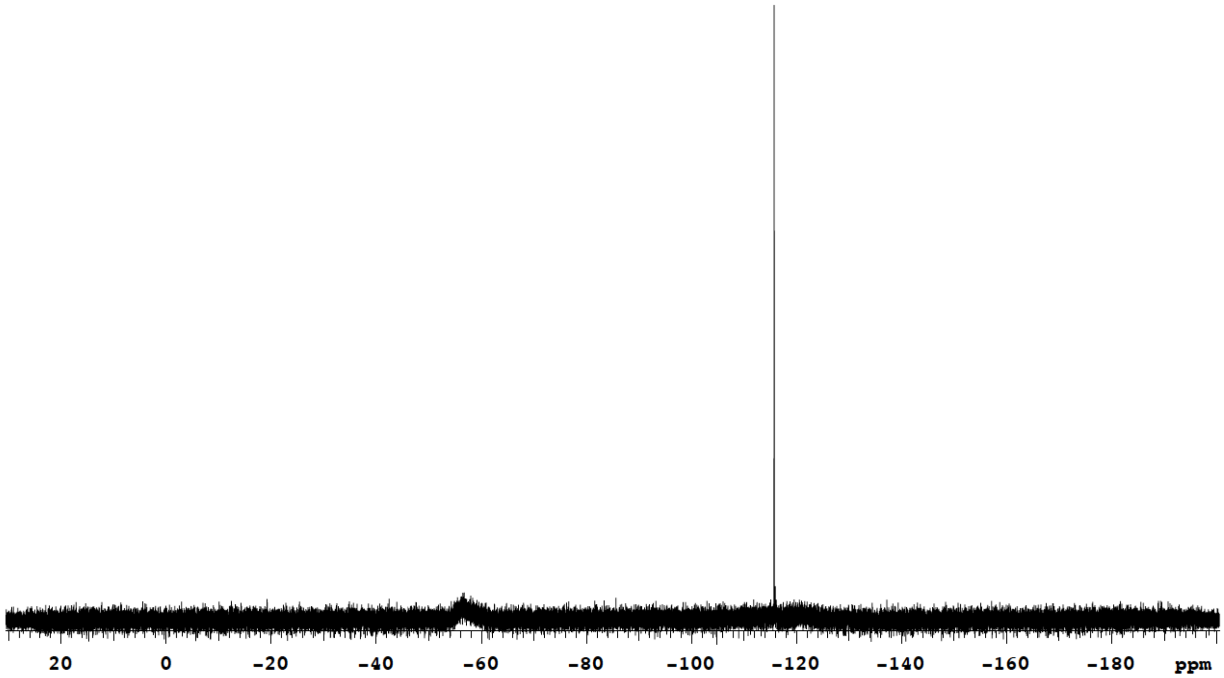

Figure S71. The $^{19}F\{^{1}H\}$ NMR spectroscopy in d^6-DMSO for the transmetallation reaction giving rise to free ligand **4a**, as well as traces of complexes mixtures corresponding to **4a-Ga.**

7.1. Titration experiments of 4a with $GaCl_3$

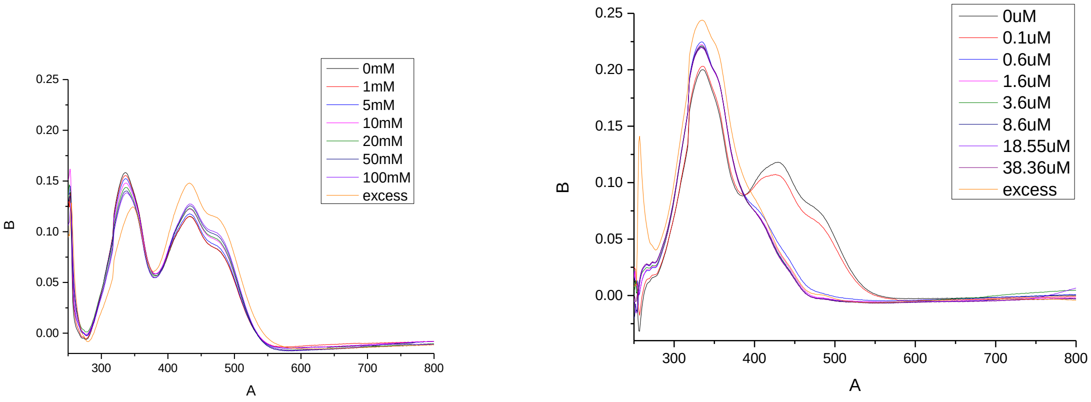

Figure S72. UV-vis absorption spectrum for titration of **4a** ligand (0.01mM in DMSO) with NaOAc (A) and $GaCl_3$ (B) in DMSO.

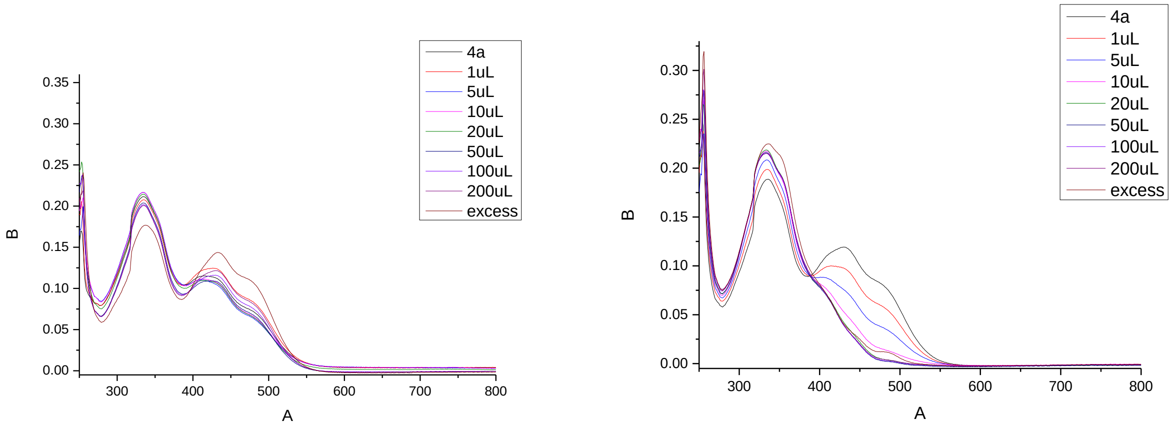

Figure S73. Titration experiment showcasing changes occurring to the UV/Vis spectrum of (FbnzTSCA) compound **4a** upon addition of $GaCl_3$ in DMSO. The spectra were re-run after each addition of aliquots of $GaCl_3$.

7.2 ^{1}H NMR scale reaction for the treatment of 4f with $GaCl_3$ in a 1:1 scale.

Scheme S7:

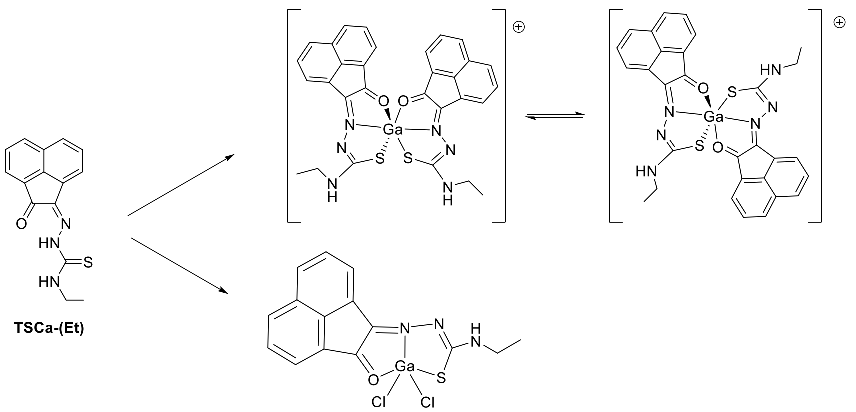

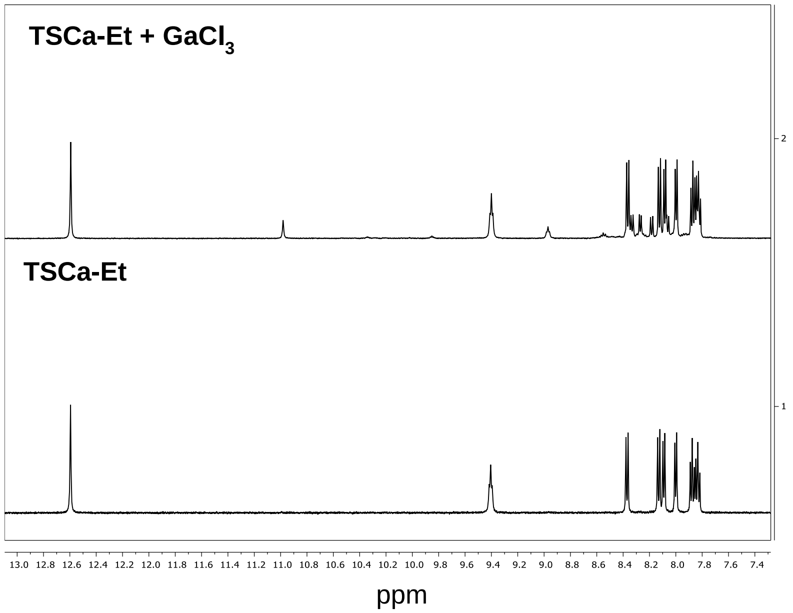

Figure S74 ^{1}H NMR scale reaction for the formation of a small amount **4f-Ga (likely as optical isomers)** from the free ligand **4f**, as well as the presence of unreacted **4f** (proposed pathway shown in the reaction Scheme S7).

7.3 Pathway to the anhydrous synthesis of Ga-4f using NEt_3

In a glove box, 2 equivalent of compound **4f** (0.07 g, 0.25 mmol) was suspended in anhydrous THF (10 ml) and 1 equivalent of anhydrous $GaCl_3$ (0.022 g, 0.125 mmol) was added to the suspension. An instant colour change from yellow to red was observed upon the addition of 0.025 ml triethylamine. The mixture was then stirred for 24 h at room temperature under argon. Pentane (15 ml) was then added to the solution and the mixture was placed in the freezer overnight and a yellow solid crashed out. This solid (fraction A) was removed by filtration. The filtrate had solvent removed by rotary evaporation to leave a brown solid (fraction B). Both fractions were analysed by mass spectrometry which indicated formation of mixtures of Ga-containing products as well as free ligand.

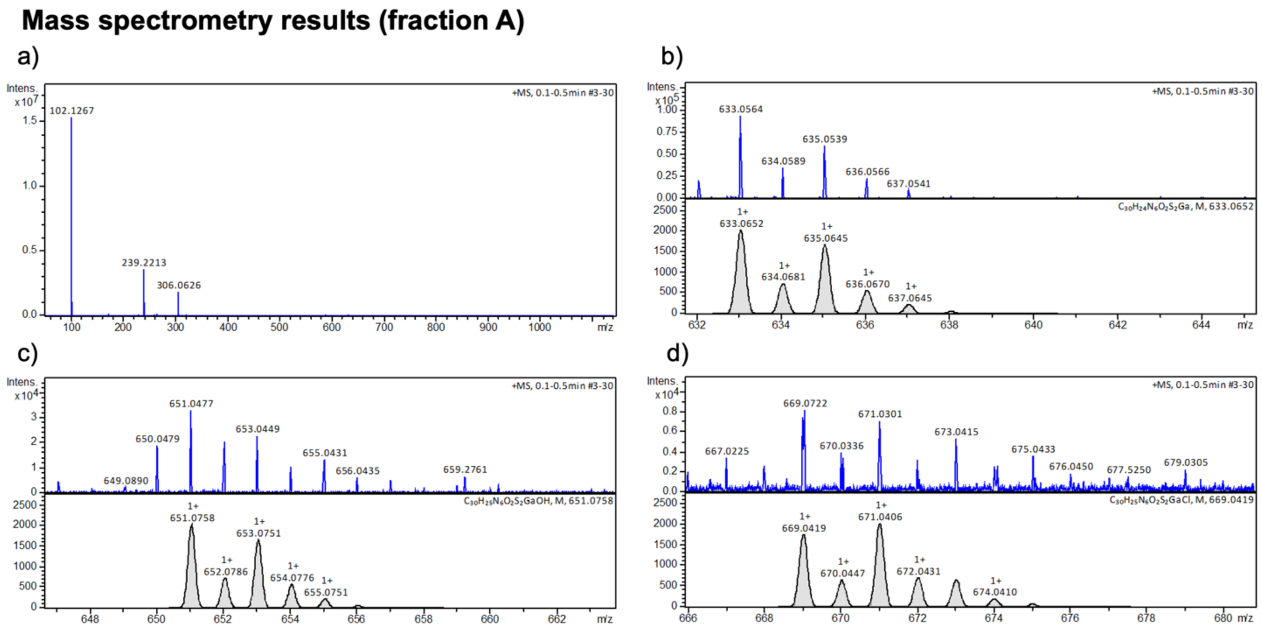

Figure S75. Positive mode ESI MS: a) full spectrum; Expansions onto relevant peaks assignable to **1:2 [Ga]:[4f ligand]** species: b) $[M]^+$, c) $[M+H+OH]^+$, d) $[M+H+Cl]^+$.

Mass spectra results (fraction B)

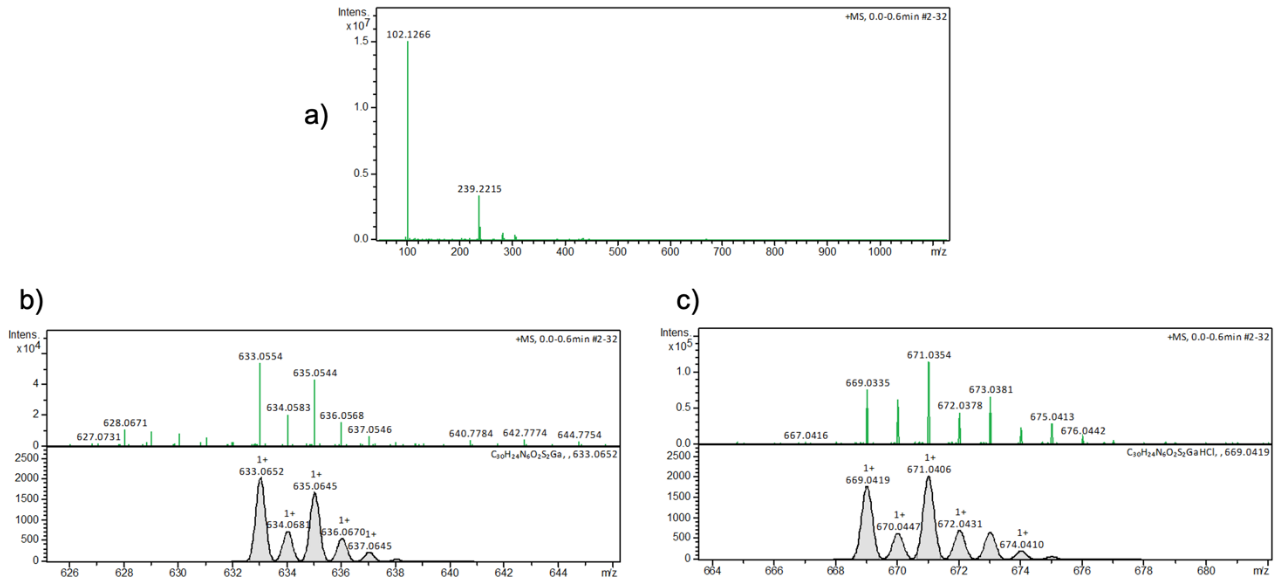

Figure S76. Positive mode ESI MS: a) full spectrum; Expansions onto relevant peaks assignable to **1:2 [Ga]:[4f ligand]** species: b) $[M]^+$, c) $[M+H+Cl]^+$

7.4 Alternative procedure for synthesis of Ga-4f under anhydrous conditions

Compound **4g** (mono Et-TSC (0.07 g, 0.25 mmol) was suspended in anhydrous THF (10 ml) and 1 molar equivalent of anhydrous $GaCl_3$ (0.022 g, 0.125 mmol) was added to the suspension. The mixture was stirred for 24 h at room temperature under argon. Pentane (15 ml) was then added to the solution and the mixture was placed in the freezer overnight and a yellow solid crashed out. This solid (fraction A) was removed by filtration. The filtrate had solvent removed by rotary evaporation to leave a brown solid (fraction B).

Mass spectra results (fraction B)

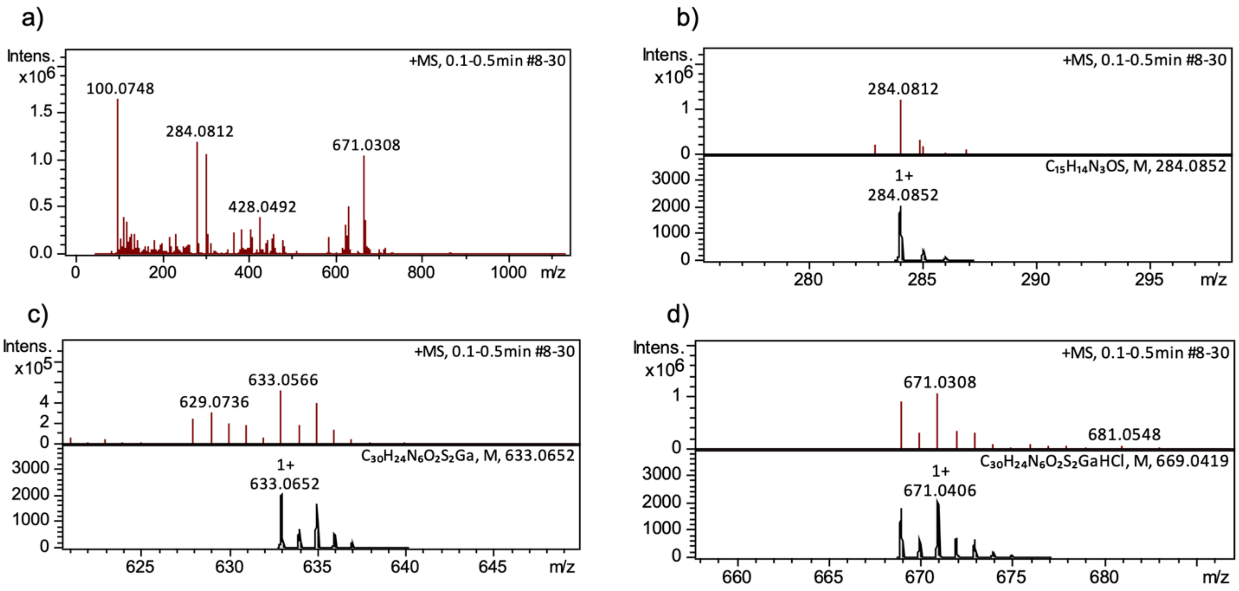

Figure S77. Positive mode ESI MS: a) full spectrum; Expansions onto relevant peaks assignable to **1:2 [Ga]: [4f ligand]** species: b) [mono free ligand]$^+$, c) [M]$^+$, d) [M+H+Cl]$^+$.

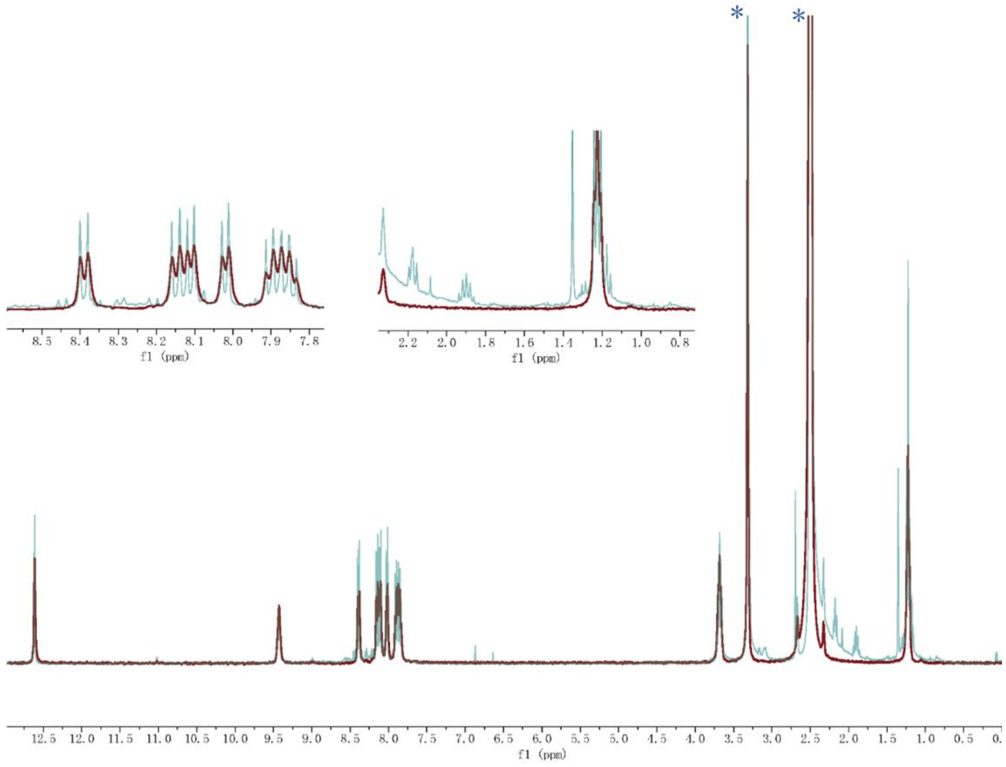

Figure S78. Overlay of the 1H NMR spectrum (400 MHz, 298 K, DMSO-d_6) of the monothiosemicarbazone ligand **4f** (red line) overlaid onto that of the corresponding reaction product (expecting formation of **4f-Ga** complex, blue line). (* correspond to the residual solvents, d_6-DMSO and H_2O).(fraction B, emerging from the anhydrous synthetic procedure). Data suggests that only traces of the desired gallium compounds have been isolated.

7.5 Pathway to the anhydrous synthesis of Ga-4g using NEt_3

In a glove box, the ligand **4g** (mono Allyl-TSC, 0.07g, 0.25 mmol) was suspended in anhydrous THF (10 ml), 1 equivalent of anhydrous $GaCl_3$ (0.022g, 0.125 mmol) was added carefully to the suspension observing a 2:1 ratio of ligand to [Ga]. Then to this mixture, 0.025 ml triethylamine was added and an instant colour change was observed from yellow to a darker orange. The mixture was stirred for 24 h at room temperature under argon. Pentane (15 ml) was then added to the solution and subsequently the mixture was placed in the freezer overnight and an orange solid crashed out. This solid (fraction A) was removed by filtration. The filtrate had solvent removed by rotary evaporation to leave an orange solid (fraction B). Both fractions were analysed by Mass Spectrometry which showed that whilst it is possible to isolate the desired species, additional gallium containing fragments may also form from this reaction, even under the carefully controlled anhydrous conditions utilised.

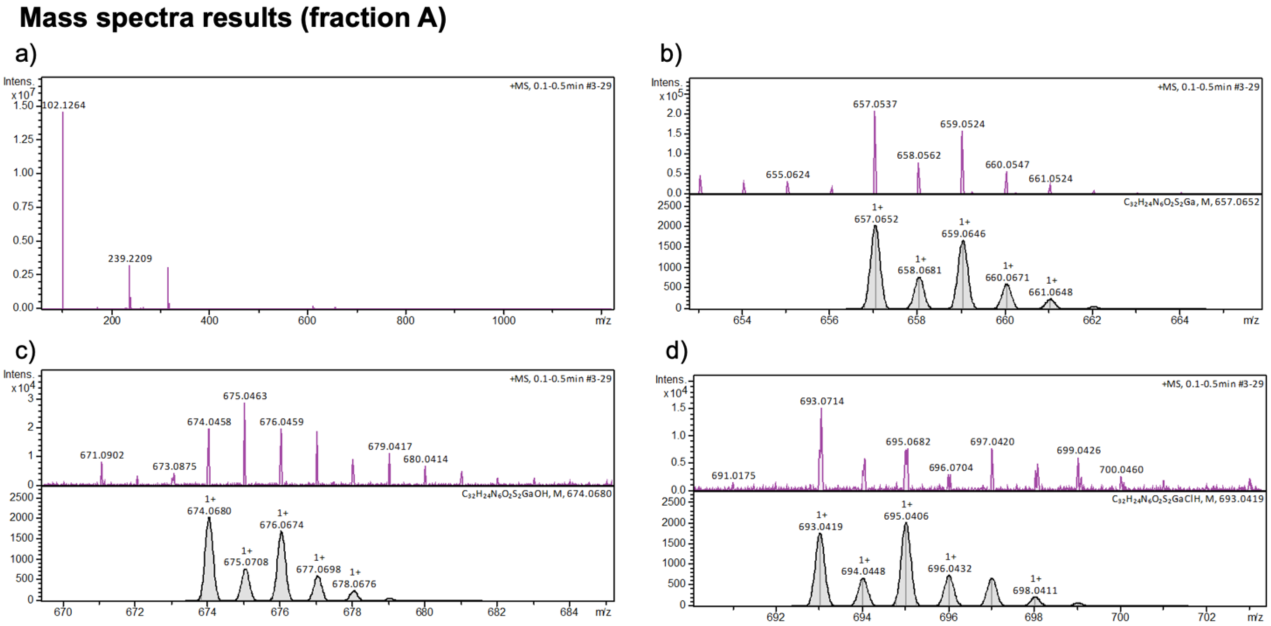

Figure S79. Positive mode ESI MS: a) full spectrum; Expansions onto relevant peaks assignable to **1:2 [Ga]:[4g ligand]** species: b) $[M]^+$, c) $[M+OH]^+$, d) $[M+H+Cl]^+$

Mass spectra results (fraction B)

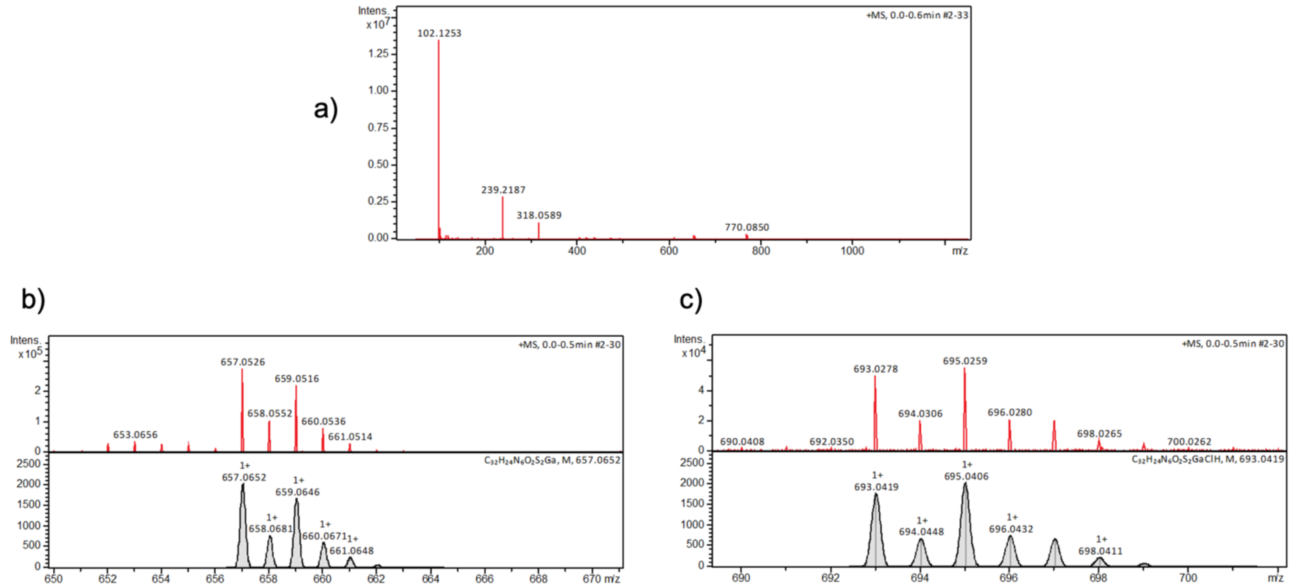

Figure S80. Positive mode ESI MS: a) full spectrum; Expansions onto relevant peaks assignable to **1:2 [Ga]: [4g ligand]** species: b) $[M]^+$, c) $[M+H+Cl]^+$

7.6. Alternative pathway for the synthesis of Ga-4g under anhydrous conditions

2 equivalents of mono Allyl-TSC (0.07 g, 0.25 mmol) were suspended in anhydrous THF (10 ml), 1 equivalent of anhydrous $GaCl_3$ (0.022 g, 0.125 mmol) was added to the suspension. The mixture was stirred for 24 h at room temperature under argon. Pentane (15 ml) was then added to the solution and the mixture was placed in the freezer overnight and an orange solid crashed out. This solid (fraction A) was removed by filtration. The filtrate had solvent removed by rotary evaporation to leave an orange solid (fraction B).

Mass spectra results (fraction A)

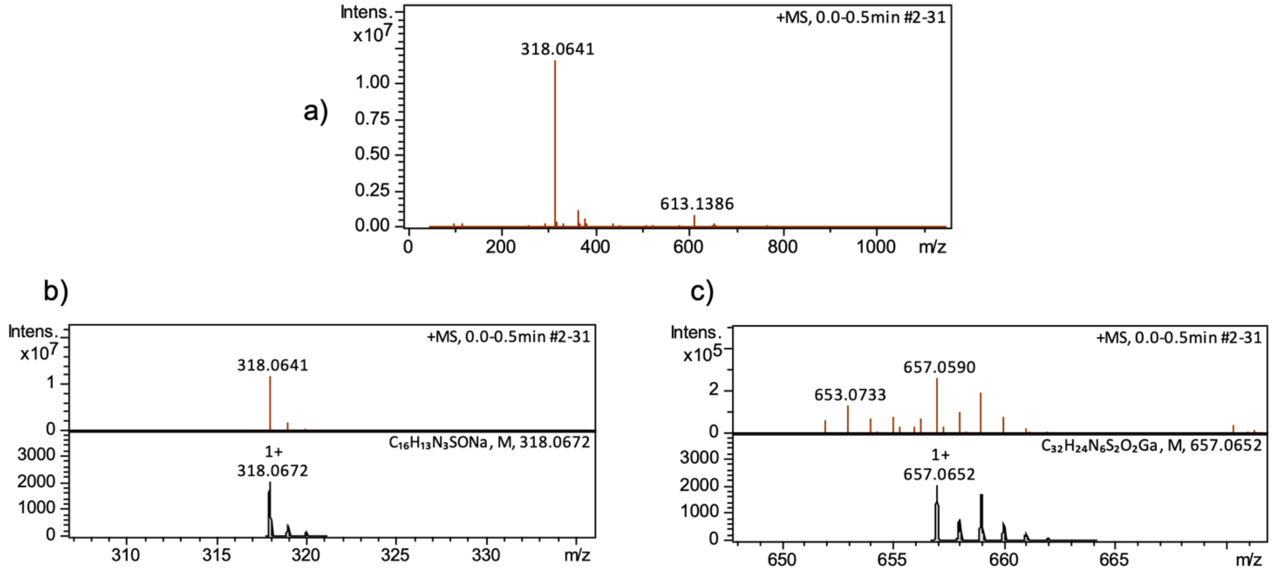

Figure S81. Positive mode ESI MS: a) full spectrum; Expansions onto relevant peaks assignable to b) [free ligand $4g+Na]^+$ and **1:2 [Ga]: [4g ligand]** species: c) $[M]^+$.

Mass spectra results (fraction B)

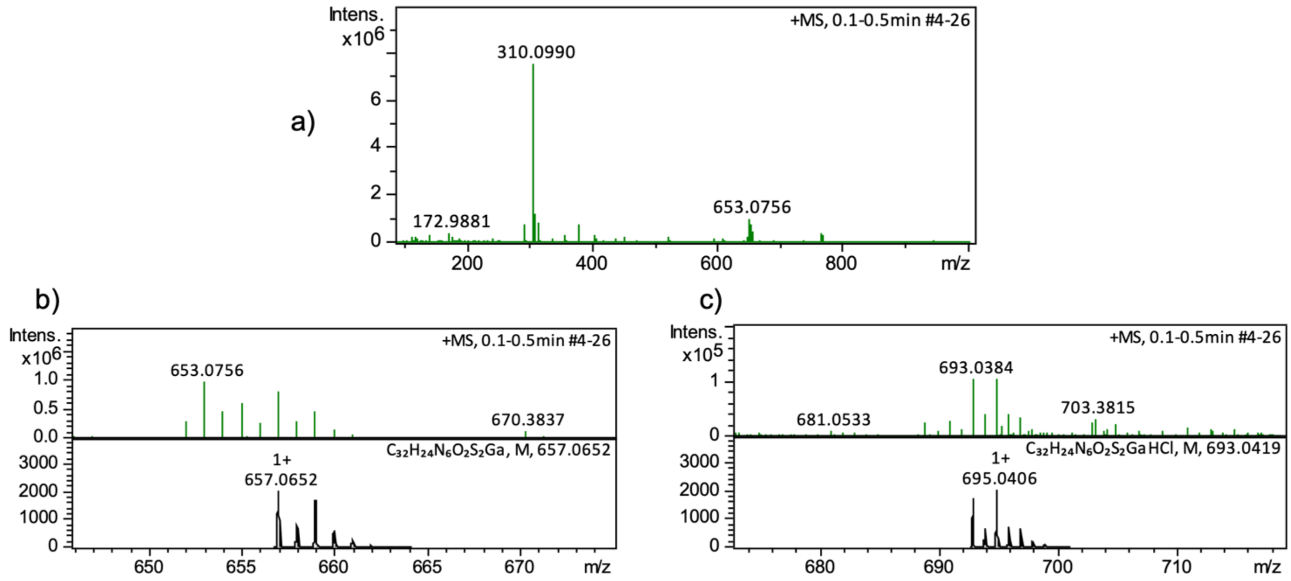

Figure S82. Positive mode ESI MS: a) full spectrum; Expansions onto relevant peaks assignable to b) $[M]^+$, c) $[M+H+Cl]^+$.

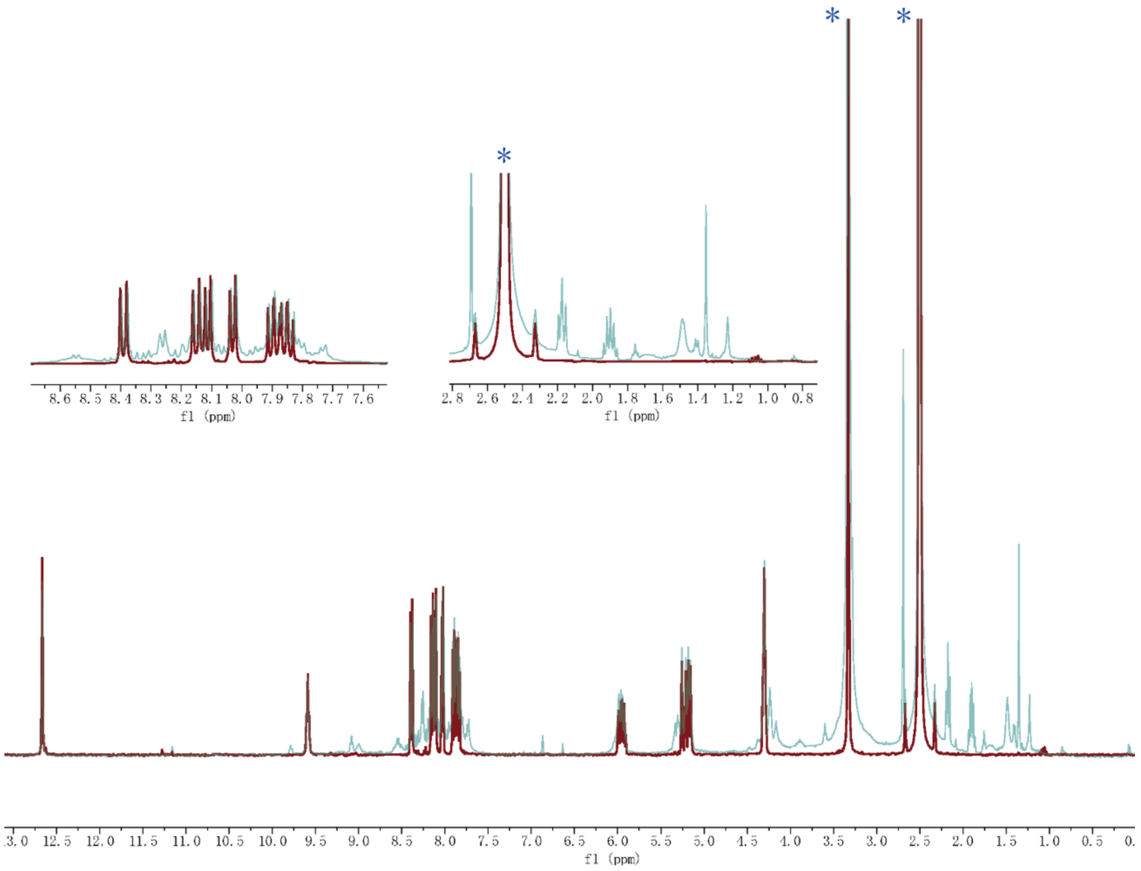

Figure S83. Overlay of the ^{1}H NMR spectrum (400 MHz, 298 K, DMSO-d_6) of the monothiosemicarbazone ligand **4g** (red line) onto that of the corresponding **4g-Ga** complex (blue line). (* correspond to the residual

solvents, d_6-DMSO and H_2O). (fraction B, made from the anhydrous synthesis reaction). Data suggests that only traces of the desired gallium compounds have been isolated.

7.6 Alternative procedure towards the formation of Ga-4g

Compound **4g**, mono Allyl-TSC (0.07 g, 0.25 mmol, 2 molar equivalent) was suspended in THF (10 ml). 1 equivalent of anhydrous $GaCl_3$ (0.022 g, 0.125 mmol) was dissolved in degassed water (0.5 ml), then THF (2 ml) was added. The $GaCl_3$ solution was added dropwise to the suspension of mono Allyl-TSC. The mixture was stirred for 24 h at room temperature. Pentane (15 ml) was then added to the solution and the mixture was placed in the freezer overnight and an orange solid crashed out. This solid (fraction A) was removed by filtration. The filtrate had solvent removed by rotary evaporation to leave an orange solid (fraction B).

Mass spectra results (fraction A)

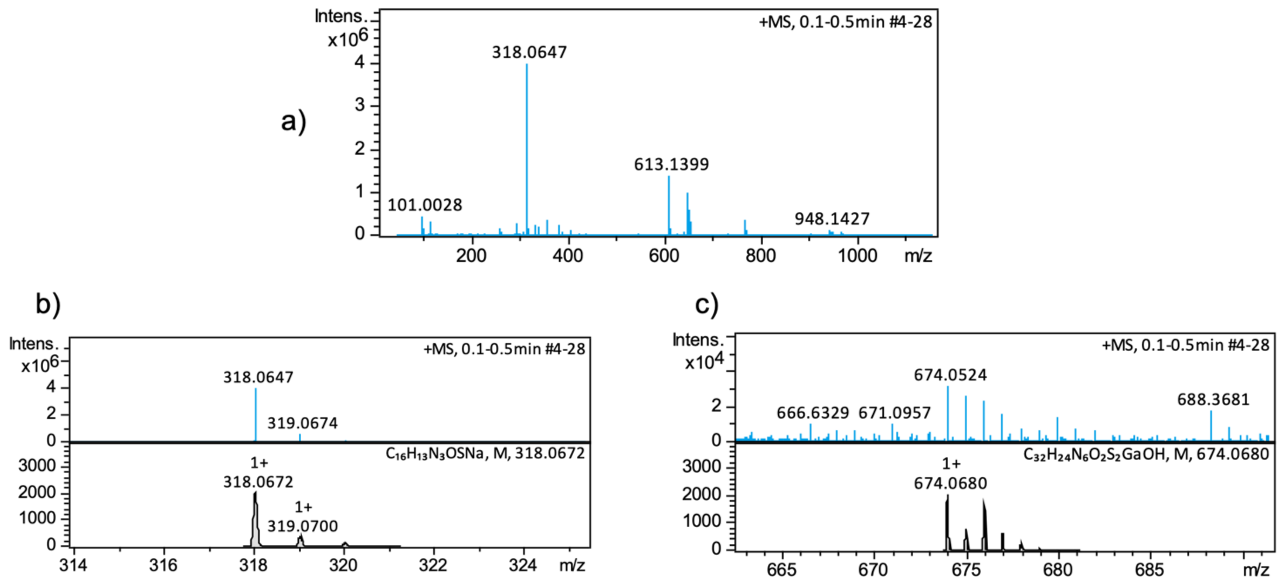

Figure S84. Positive mode ESI MS: a) full spectrum; Expansions onto relevant peaks assignable a) full spectra, b) [mono free ligand+Na]$^+$, c) [M+OH]$^+$

Mass spectra results (fraction B)

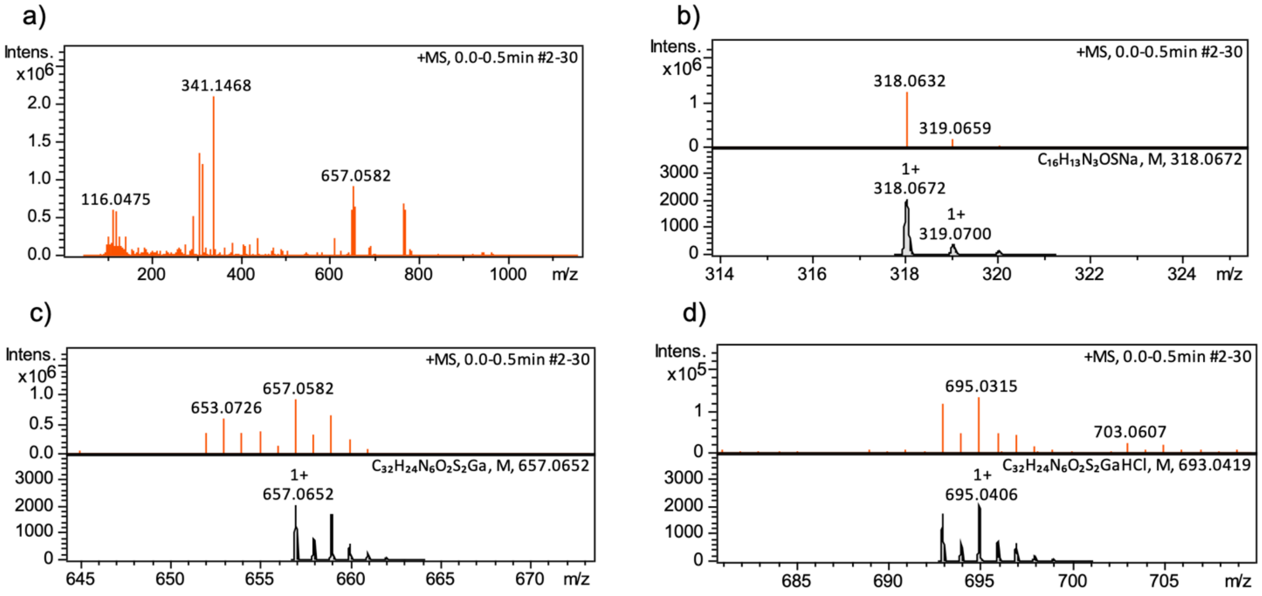

Figure S85. Positive mode ESI MS: a) full spectra, a) full spectrum; Expansions onto relevant peaks assignable to b) [mono free ligand+Na]$^+$, c) [M]$^+$, d) [M+H+Cl]$^+$.

7.8 Reaction between excess aqueous [Ga(III)] and 4g

A solution of aqueous $GaCl_3$ (0.7 mol dm^{-3}) was prepared in degassed water. (Note: It was assumed this solution then contained a mixture of $GaCl_3$ and $Ga(OH)_3$, either as monomers or as oligomers, as well as other solvated Ga(III) species). The ligand **4g** (0.049g, 0.17mmol) was dissolved in 30 ml of THF in a Schlenk flask under N2. Then, 1 ml of the [Ga] stock solution was added by syringe to the THF ligand solution. The mixture was left to stir for 24 hours at the room temperature. Over that period the solution became gradually darker, before becoming dark orange. To this solution, 5 mL of pentane was added under stirring and the resulting slurry was transferred to a round bottom flask. The solution was left in a freezer overnight where a dark orange solid crashed out. This solid was removed by filtration giving rise to a solid denoted **4g-Ga** (**fraction A**), which was analysed by ESI-MS+. From the filtrate fraction, the solvent was removed under reduced pressure and the second fraction (denoted **4g-Ga fraction B**) was also analysed by ESI+. In both cases the ^{1}H NMR spectroscopy showed formation of complex mixtures of products and free ligand. A ESI+ MS analysis was performed on fraction A of **4g-Ga**. A close inspection showed one peak with m/z = 296.1246 corresponded to the free ligand, as well as un-assignable [Ga] containing peaks at higher m/z. The ES+ MS of fraction **4g-Ga B** also indicated the presence of fragments corresponding to the free ligand as well as fragments with high m/z: e.g. one peak of interest, m/z = 729.3620 was assignable to a gallium-ligand complex of type $[GaL_2Cl_2+H^+]$ where the two chlorines seem to remain associated with the complex ion in mass spectrometry.

+MS, -Spectral Bkgrnd, 1.0-1.3min #(94-120)

No of Most Intense MS Peaks Analysed

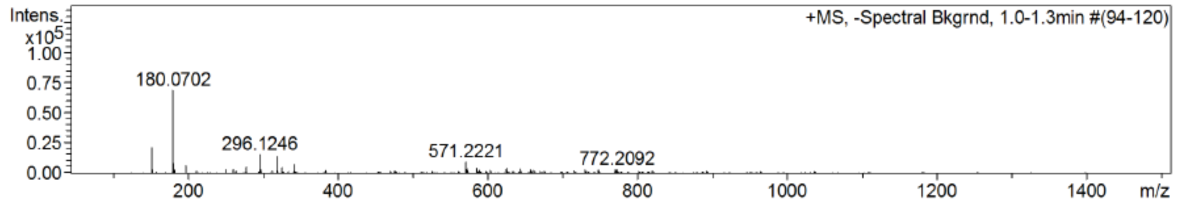

#	m/z	I	I %	Area	S/N
1	152.0717	21251	31.0	443	2499.5
2	180.0702	68610	100.0	1604	5753.5
3	181.0738	8565	12.5	222	710.9
4	197.0955	6613	9.6	116	418.0
5	278.1642	5093	7.4	222	103.8
6	296.1246	15435	22.5	691	300.0
7	318.1100	13992	20.4	647	299.2
8	326.1401	4501	6.6	219	99.9
9	341.1973	7319	10.7	357	174.9
10	571.2221	9377	13.7	1046	86.5

Figure S86. Mass spectrometry (ESI+) of fraction **4g-Ga (fraction A)**

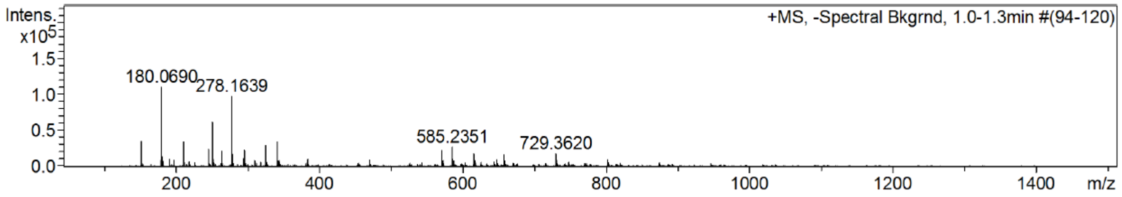

#	m/z	I	I %	Area	S/N
1	152.0708	35151	31.8	701	378.4
2	180.0690	110551	100.0	2583	811.9
3	211.1127	34809	31.5	1057	161.2
4	246.1332	23636	21.4	846	72.8
5	251.1492	62621	56.6	2196	183.9
6	278.1639	97523	88.2	3801	229.8
7	296.1224	22777	20.6	946	52.6
8	326.1378	29767	26.9	1437	85.1
9	341.1951	34794	31.5	1711	113.0
10	585.2351	27039	24.5	2757	360.5

Figure S87. Mass spectrometry (ESI+) of fraction **4g-Ga (fraction B)**

7.9 Procedure towards the formation of Ga-4h

The compound **4h** mono Ph-TSC (0.08 g, 0.25 mmol, 2 equivalent) was suspended in THF (10 ml). 1 equivalent of anhydrous $GaCl_3$ (0.022 g, 0.125 mmol) was dissolved in degassed water (0.5 ml), then THF (2 ml) was added. The GaCl3 solution was added dropwise to the suspension of mono Ph-TSC. The mixture was stirred for 24 h at room temperature. Pentane (15 ml) was then added to the solution and the mixture was placed in the freezer overnight and a dark red solid crashed out. This solid (fraction A) was removed by filtration. The filtrate had solvent removed by rotary evaporation to leave a red solid (fraction B).

Mass spectra results (fraction B)

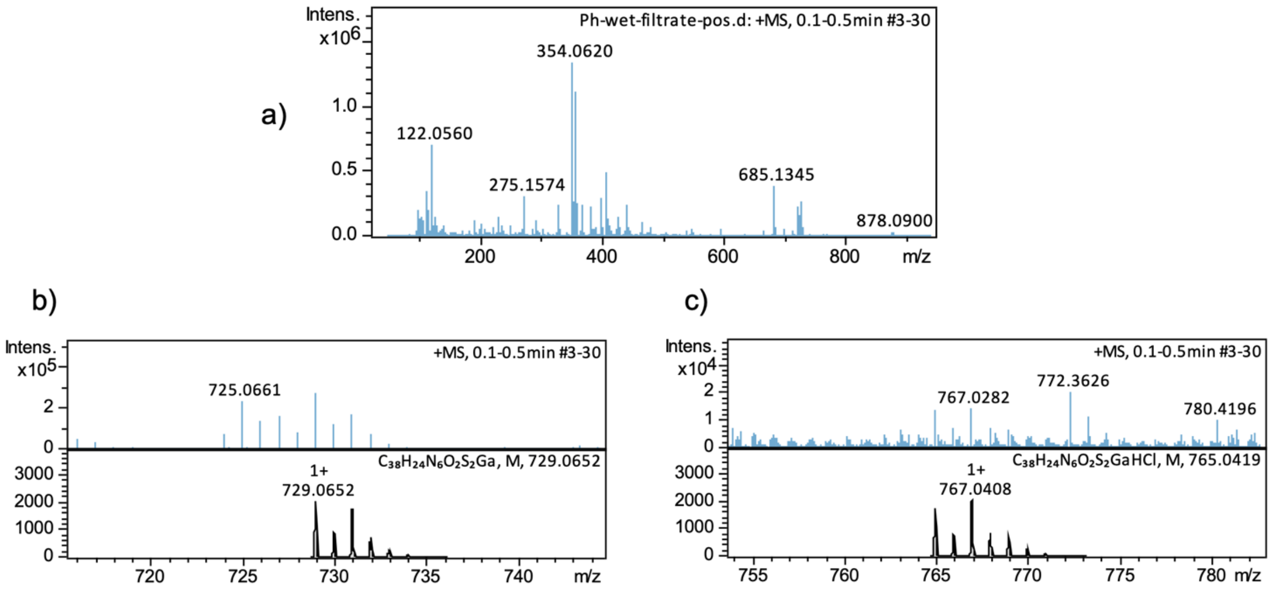

Figure S88 Positive mode ESI MS: a) full spectrum; Expansions onto relevant peaks assignable to Positive mode a) full spectra, b) $[M]^+$, c) $[M+H+Cl]^+$

7.10 Pathway to the anhydrous synthesis of Ga-4h using NEt_3

In a glove box, the ligand **4h** (mono Ph-TSC, 0.08 g, 0.25 mmol) was suspended in anhydrous THF (10 ml) and treated with anhydrous GaCl3 (0.022 g, 0.125 mmol) which was carefully added to the suspension, aiming to closely observe the 2: 1 molar ratio of 4f to $GaCl_3$ added. An instant colour change was observed upon the addition of 0.025 ml triethylamine to this mixture. The resulting mixture was stirred for 24 h at room temperature under argon. Pentane (15 ml) was then added to the solution and the mixture was placed in the freezer overnight and a dark red solid crashed out. This solid (fraction A) was removed by filtration, and the mass spectrometry analysis proved unconclusive. The filtrate had solvent removed by rotary evaporation to leave a red solid (fraction B). Detailed analysis by Mass spectrometry revealed recovery of free ligand 4h, as well as the presence of the desired **Ga-4f** species in Fraction B.

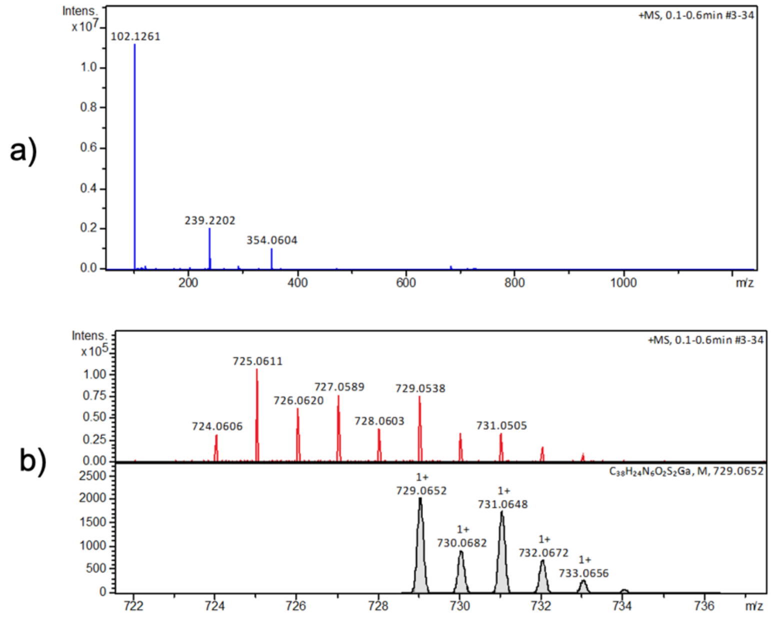

Figure S89 Positive mode ESI MS: a) full spectrum; Expansions onto relevant peaks assignable to **1:2 [Ga]: [4h ligand]** species b) $[M]^+$

7.11 Anhydrous synthesis of Ga-4h

The compound **4h** mono Ph-TSC (0.08 g, 0.25 mmol, 2 molar equiv) was suspended in anhydrous THF (10 ml). To this suspension, anhydrous $GaCl_3$ (0.022 g, 0.125 mmol) was added (1 molar equivalent). The mixture was stirred for 24 h at room temperature under argon. Pentane (15 ml) was then added to the solution and the mixture was placed in the freezer overnight and a dark red solid crashed out. This solid (fraction A) was removed by filtration. The filtrate had solvent removed by rotary evaporation to leave a red solid (fraction B).

Mass spectra results (fraction B)

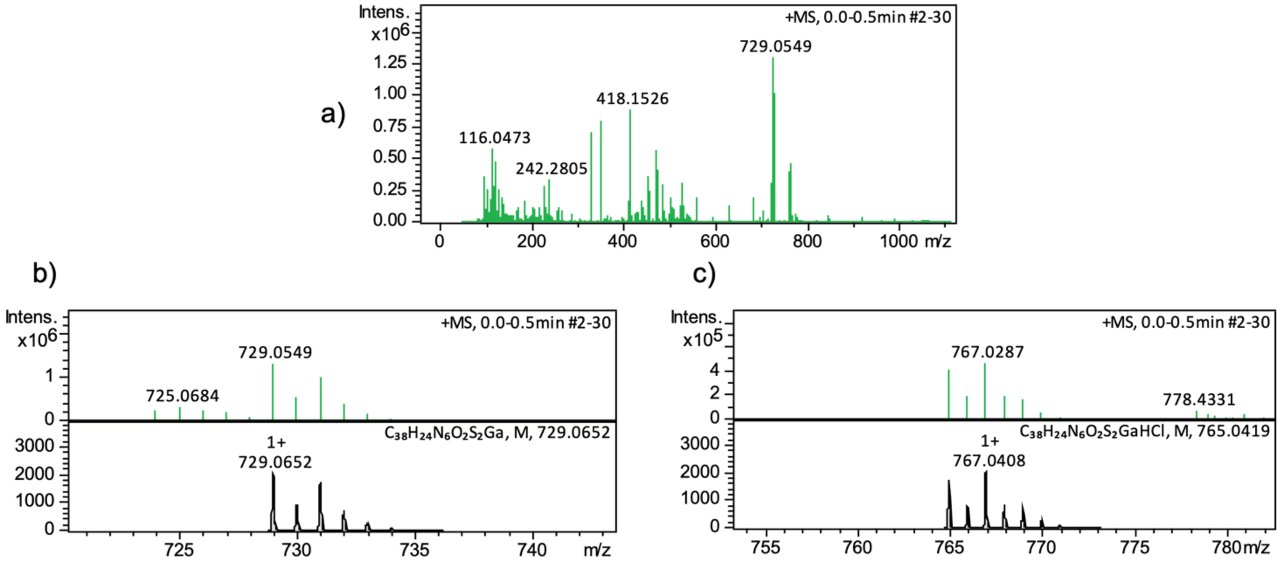

Figure S90. Positive mode ESI MS: a) full spectrum; Expansions onto relevant peaks assignable to b) $[M]^+$, c) $[M+H+Cl]^+$.

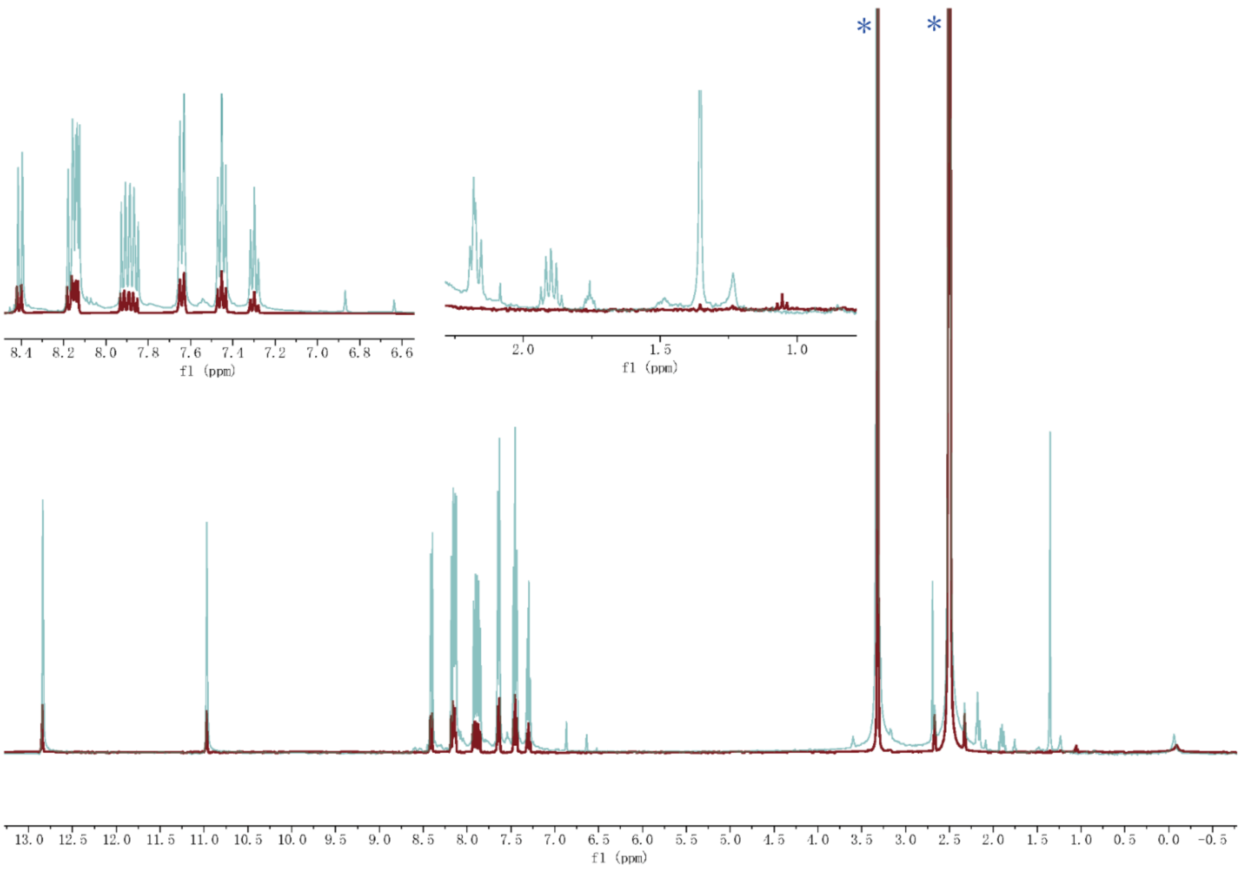

Figure S91. Overlay of the ^{1}H NMR spectrum (400 MHz, 298 K, DMSO-d_6) of the monothiosemicarbazone ligand **4h** (red line) overlaid onto that of the corresponding product, expecting formation of the **4h-Ga** complex (blue line). (* correspond to the residual solvents, d_6-DMSO and H2O). (fraction B, made from the anhydrous synthesis reaction). Data suggests that only traces of the desired gallium compound have been isolated.

7.12 Alternative method for the reaction between $GaCl_3$ and Compound 4h

Compound **4h** (0.051g, 0.15mmol) was added to a solution of dry THF (20 mL) containing $GaCl_3$ whilst the reaction vessel was kept under a nitrogen. (Note: Anhydrous $GaCl_3$ (0.0135g, 0.077mmol) was weighed out and added to a round bottom flask provided with a septum in a glove box). The reaction mixture was left to stir for 24 hours under N_2, then a small amount of pentane was added to the solution and the mixture was placed in the freezer overnight. A red solid crashed out and this solid (denoted **4h-Ga fraction A**) was removed by filtration. The filtrate was concentrated by rotary evaporation to generate a red oily solid residue (denoted **4h-Ga B**). ^{1}H NMR analysis in d^6-DMSO proved inconclusive with formation of protonated ligand species being observed. The HPLC was dominated by the free ligand species, indicating decomposition in wet solvents. The Mass spectrometry (ESI+) of fraction **4h-Ga A** did not show expected molecular ion peak centred at m/z 753 region. Instead, a peak centred at m/z = 354.1161, representing the free ligand associated with Na, was observed together with peaks with higher m/z. The fraction denoted **4h-Ga B** was also analysed by ES+ MS and a small peak for the m/z = 753.2173 was found, most closely assignable to a [Ga(HL)(L)+Na] fragment, where HL corresponds to the protonated ligand (**4h**).

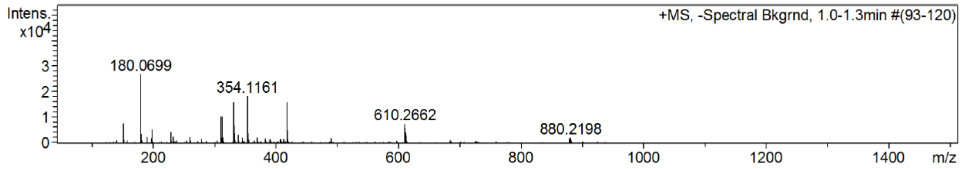

#	m/z	I	I %	Area	S/N
1	152.0716	7509	28.2	153	1709.5
2	180.0699	26664	100.0	636	4467.6
3	199.0475	5274	19.8	53	518.7
4	311.0598	10339	38.8	509	165.7
5	313.0579	10274	38.5	496	161.7
6	332.1308	15812	59.3	817	211.6
7	354.1161	18148	68.1	915	207.2
8	418.2176	15949	59.8	1137	208.2
9	419.2193	5125	19.2	323	67.7
10	610.2662	7445	27.9	350	250.2

Figure S92 Mass spectrometry (ESI+) of fraction **4h-Ga A**

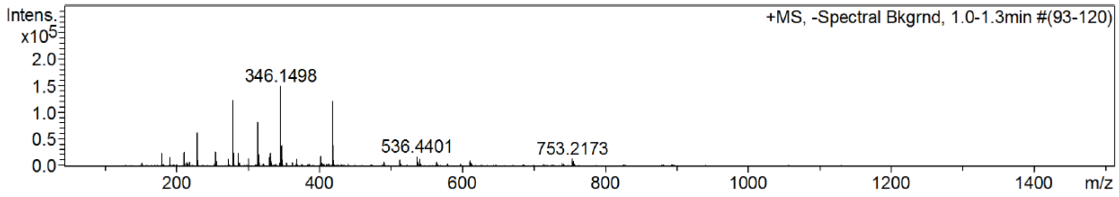

#	m/z	I	I %	Area	S/N
1	211.1131	25320	17.0	759	131.9
2	229.1998	61918	41.6	1809	303.5
3	255.1242	26425	17.8	922	119.4
4	279.1308	122584	82.4	4126	516.2
5	287.1557	24518	16.5	1023	101.0
6	314.1174	82677	55.6	3683	223.4
7	346.1498	148720	100.0	6672	282.6
8	347.1512	38188	25.7	1996	71.9
9	418.2195	120430	81.0	6823	247.5
10	419.2198	39729	26.7	2401	82.8

Figure S93 Mass spectrometry (ESI+) of fraction **4h-Ga B**

7.13 Transmetallation reaction between 4f-Zn and $GaCl_3$.

The complex **Zn-4f** ($Zn(EtTSC)_2$, 0.06g, 0.10 mmol) was suspended in dried MeOH (20 ml), and then added dropwise to a flask containing neat and anhydrous $GaCl_3$ (0.18 g, 0.125 mmol). The mixture was then heated to 65 ℃ over 5 h under argon. The reaction mixture was allowed to reach room temperature, and the slurry was subsequently concentrated under reduced pressure to yield a red oil. To this, a small amount of Et_2O (10 mL) was then added and a red precipitate formed. The precipitate (fraction A) was removed by filtration. The filtrate had solvent removed by rotary evaporation to leave an orange solid (fraction B). Both fractions were analysed by mass spectrometry, to show the presence of the **Zn-4f** precursor, additionally to the expected **Ga-4f** compound.

Mass spectra results (fraction B)

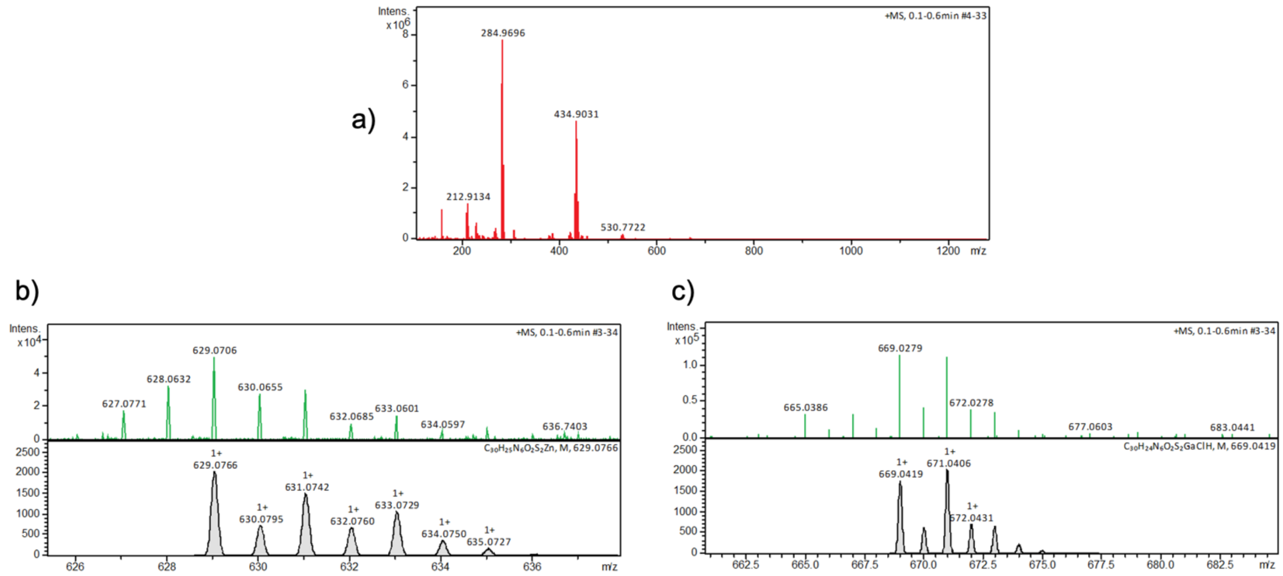

Figure S94. Positive mode ESI MS: a) full spectrum; Expansions onto relevant peaks assignable to: b) [M-Ga+Zn]$^+$, c) [M+H+Cl]$^+$

7.14 Transmetallation reaction between 4g-Zn and excess $GaCl_3$.

$GaCl_3$ (anhydrous) was added to Schlenk tube in the glove box (0.121 g, 6.9 x 10^{-4} mol). To this, the Zn(II) complex **4g-Zn** (0.030 g, 4.6 x 10^{-5} mol, suspended in 5 mL MeOH) was added dropwise under a stream of N_2. The reaction mixture was then heated to reflux over 5 h. Then, the reaction mixture was allowed to reach room temperature, and the resulting slurry concentrated under reduced pressure to yield a red oil. To this, a small amount (2 mL) of Et_2O was added. This resulted in the formation of a red precipitate which was isolated by filtration (18.2 mg). The ^{1}H NMR (d^6-DMSO) showed the presence of the free ligand additionally to a complex mixture of species. By ESI+ main fragment is assignable to a species containing Ga ion as monocoordinated to one 4g ligand and 2 OH groups, [C16H14GaN3O3S+Na] with m/z centred at 419.3482, and the presence of the desired **Ga-4f** species is also indicated by the higher m/z peaks assignable to [M]+ and [M+H+Cl]+. ESI+ and ESI- mass spectrometry support the formation of the GaL_2X_2 as well as $GaLX_2$ species, however the formation of the free ligand **4g** was also observed. Additionally, the presence of the **4g-Zn** starting material cannot be fully discounted by ^{1}H NMR and ESI+ MS.

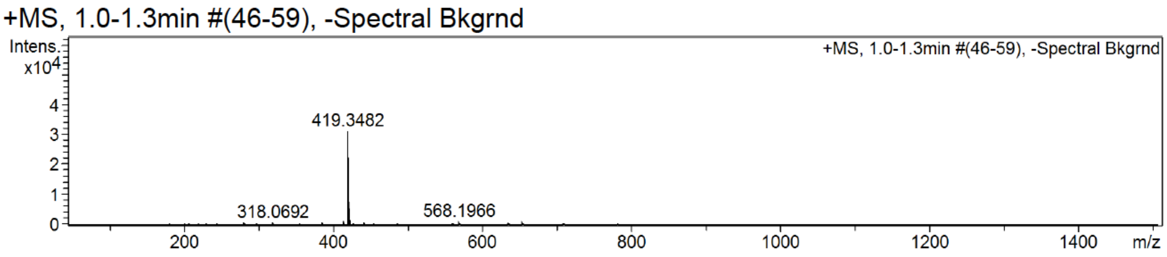

#	m/z	I	I %	Area	S/N
1	318.0692	872	2.8	68	340.3
2	384.3422	723	2.3	44	152.6
3	413.2749	1157	3.7	96	277.6
4	419.3482	31308	100.0	2204	7757.3
5	420.3514	7875	25.2	603	1961.9
6	421.3542	926	3.0	70	231.9
7	441.2968	856	2.7	57	240.7
8	568.1966	1357	4.3	137	356.6
9	633.1594	730	2.3	75	211.0
10	652.2872	1242	4.0	179	379.5

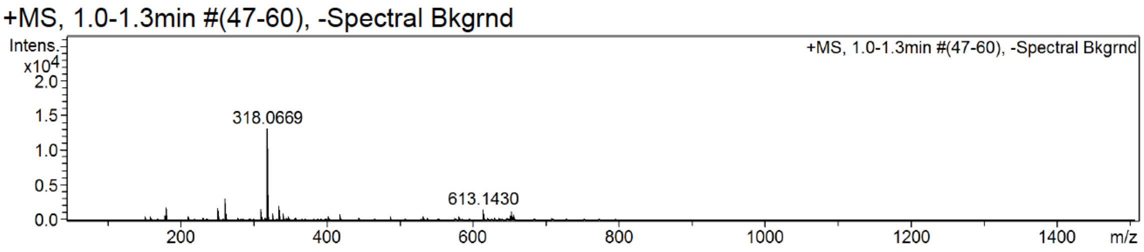

#	m/z	I	I %	Area	S/N
1	180.0462	1919	14.6	68	3635.4
2	251.1191	1767	13.4	97	701.8
3	261.0101	3088	23.4	137	1089.6
4	310.0991	1628	12.4	105	479.0
5	318.0669	13179	100.0	834	4007.4
6	319.0698	2510	19.0	140	766.3
7	334.0370	1692	12.8	146	551.4
8	340.0540	997	7.6	78	334.1
9	613.1430	1626	12.3	215	247.6
10	653.0786	1341	10.2	175	168.2

Figure S95 Mass Spectrometry (ESI+) of the resulting product mixture from the treatment of **4g-Zn** compound with excess Ga(III) recorded after the transmetallation reaction and repeated under a number of injection conditions.

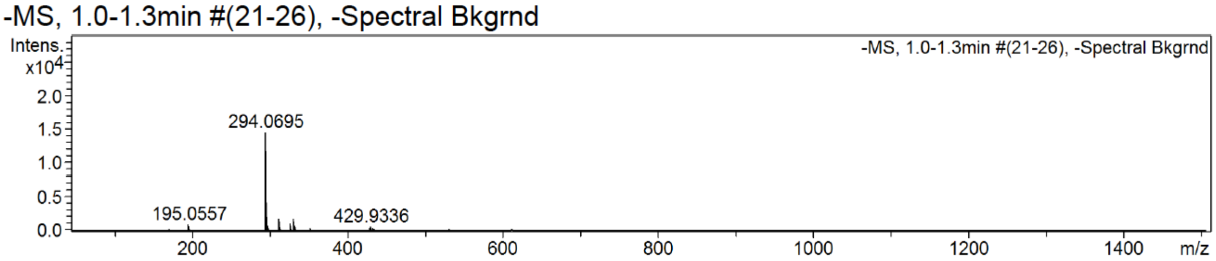

#	m/z	I	I %	Area	S/N
1	195.0557	912	6.3	25	6170.4
2	294.0695	14458	100.0	660	12753.1
3	295.0735	2706	18.7	115	2403.0
4	296.0682	815	5.6	36	728.1
5	297.1495	630	4.4	29	567.2
6	311.1673	1681	11.6	81	1671.4
7	325.1833	1102	7.6	53	1222.3
8	330.0465	1787	12.4	91	2066.5
9	332.0436	669	4.6	33	786.7
10	429.9336	638	4.4	38	2238.1

Figure S96 Mass Spectrometry (ESI-) of the resulting product of **4g-Zn** with excess Ga(II) recorded after the transmetallation reaction.

7.11 Proposed reaction scheme at the formation of 4g-Ga (R = Allyl)

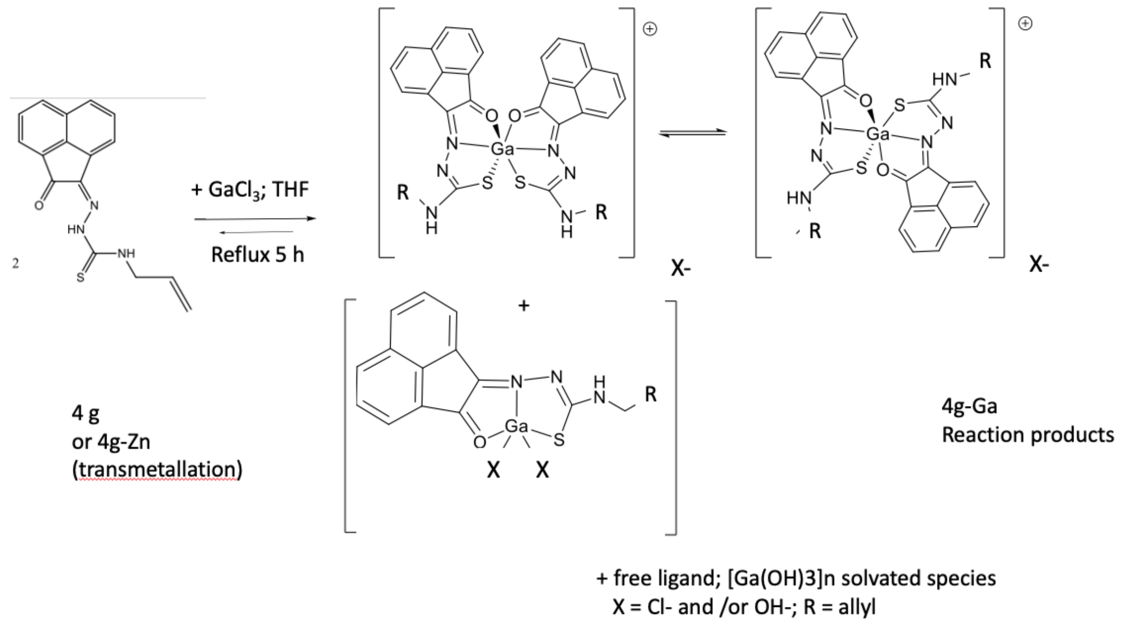

Scheme S8 Proposed formation of 4g-Ga (R = Allyl)

Figure S97. The 1H NMR spectroscopy in d6-DMSO for the transmetallation reaction – a comparison of the ^{1}H NMR for the free ligand **4g** (top spectrum), complex **4g-Zn** (middle spectrum) and the products of the Zn(II) to Ga(III) transmetallation reaction (bottom spectrum; free ligand and **4g-Ga** mixture, see Scheme S8).

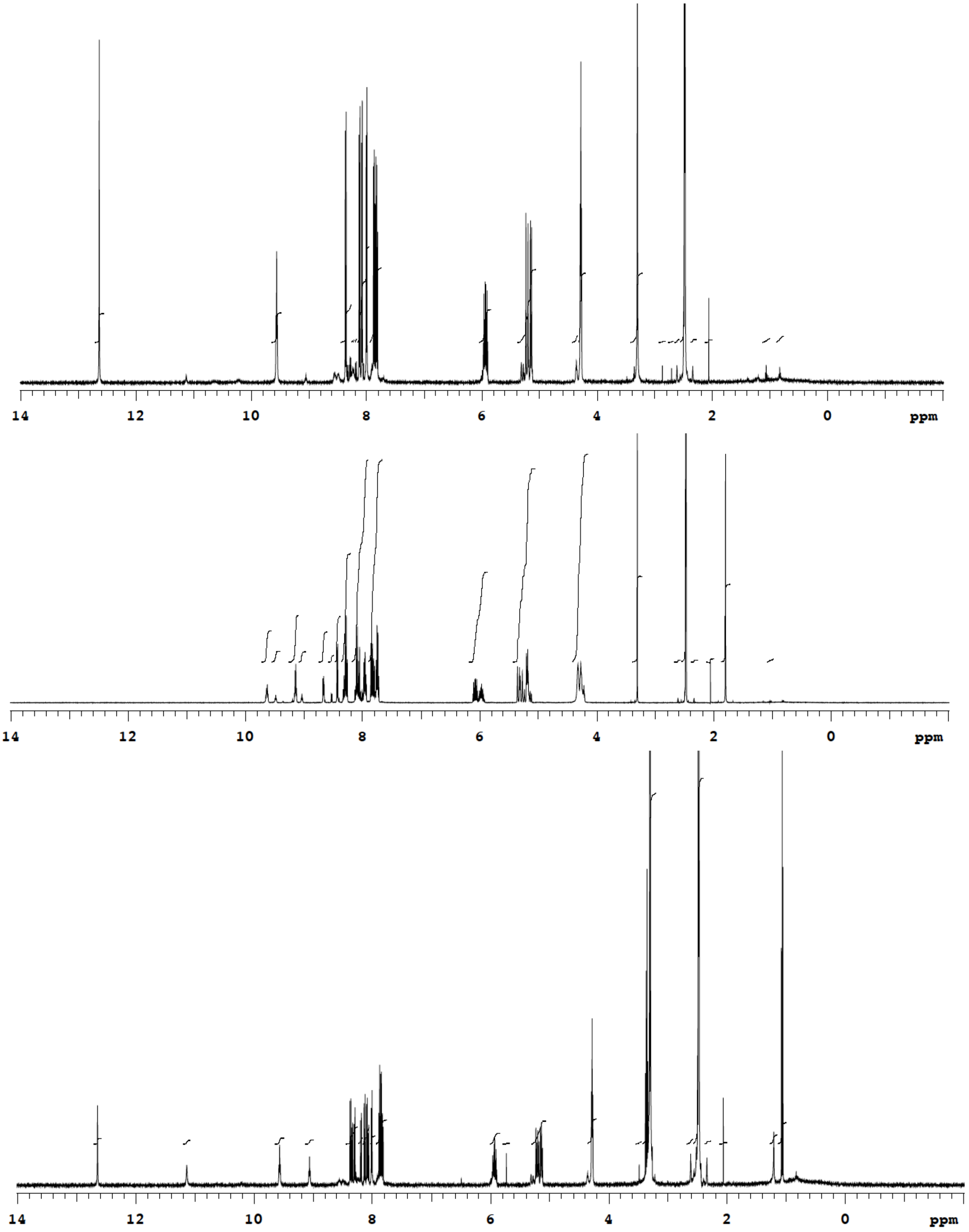

Synthesis of Ga-(Et-TSC)2 by transmetallation reaction (MeOH)

The complex **4f-Zn** (Zn(EtTSC)2, 0.06g, 0.10 mmol, 1 molar equivalent) was suspended in anhydrous MeOH (20 ml) , added dropwise to 10 equivalent of anhydrous $GaCl_3$ (0.18 g, 1 mmol) and refluxed at 65 ℃ for 5 h under argon. The reaction mixture was allowed to reach room temperature, and the slurry was concentrated under reduced pressure to yield a red oil. Et_2O was then added until a red precipitate formed. The precipitate (fraction A) was removed by filtration. The filtrate had solvent removed by rotary evaporation to leave an orange solid (fraction B) which was analysed by mass spectrometry.

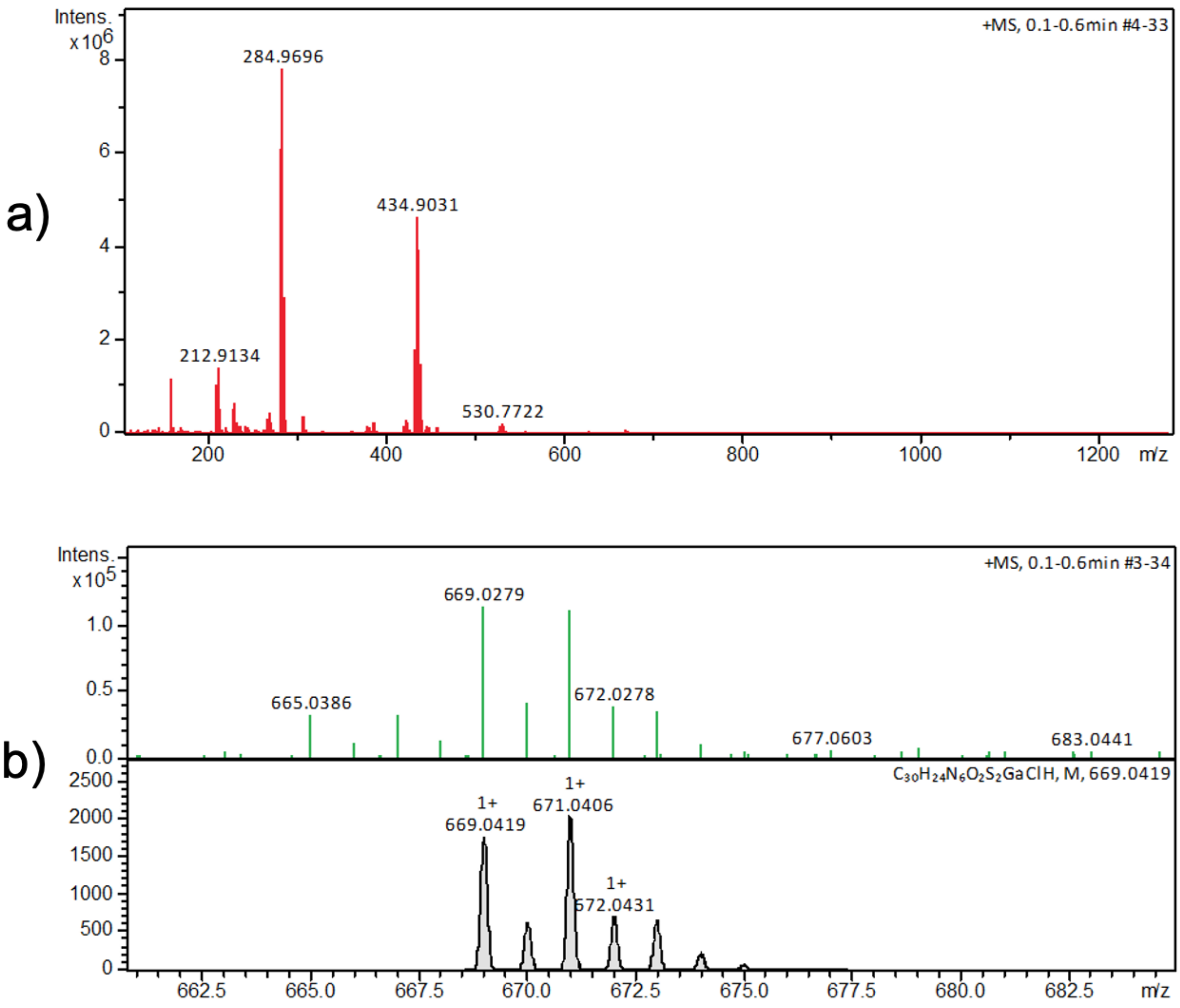

Figure S98. ESI Mass spectrometry results (fraction B): Positive mode a) full spectra, b) $[M+H+Cl]^+$

Alternative synthetic approach towards Ga-(Et-TSC)2 by transmetallation reaction (THF)

The compound **4f**, (Zn(EtTSC)2,0.06g, 0.10 mmol, 1 equivalent) was suspended in anhydrous THF (20 ml) , added dropwise to 10 equivalent of anhydrous $GaCl_3$ (0.18 g, 1 mmol), refluxed at 65 ℃ for 5 h under argon. The reaction mixture was allowed to reach room temperature, and the slurry was concentrated under reduced pressure to yield a red oil. Et_2O was then added until a red precipitate formed. The precipitate (fraction A) was

removed by filtration. The filtrate had solvent removed by rotary evaporation to leave an orange solid (fraction B).

Mass spectra results (fraction A)

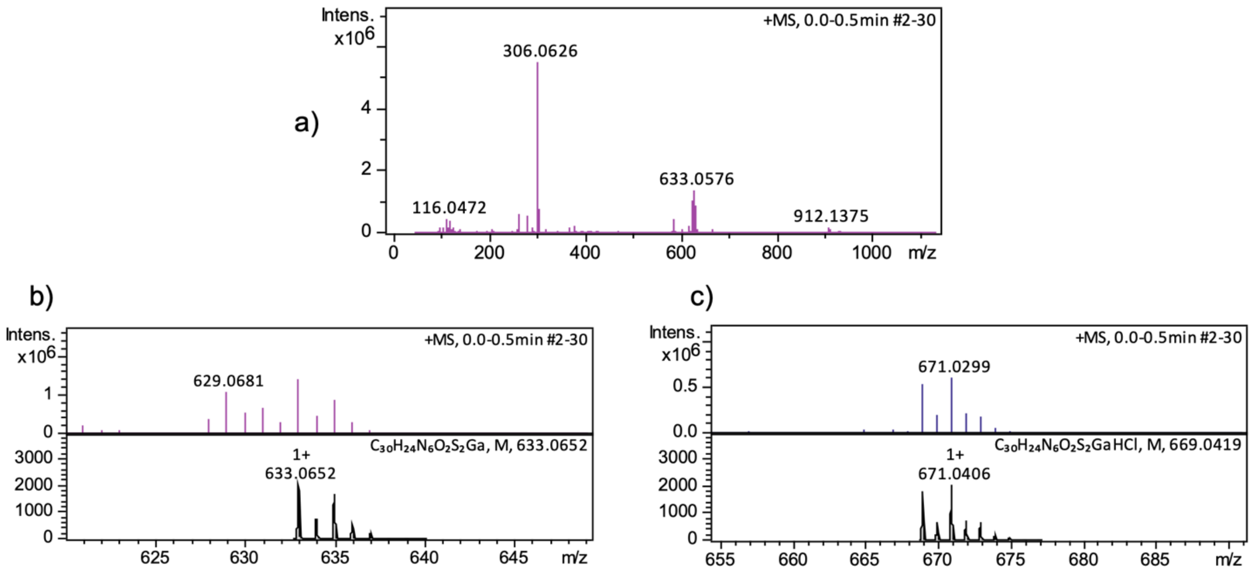

Figure S99. ESI Mass spectrometry results: Positive mode a) full spectra, b) [M]+, c) [M+H+Cl]+

Mass spectra results (fraction B)

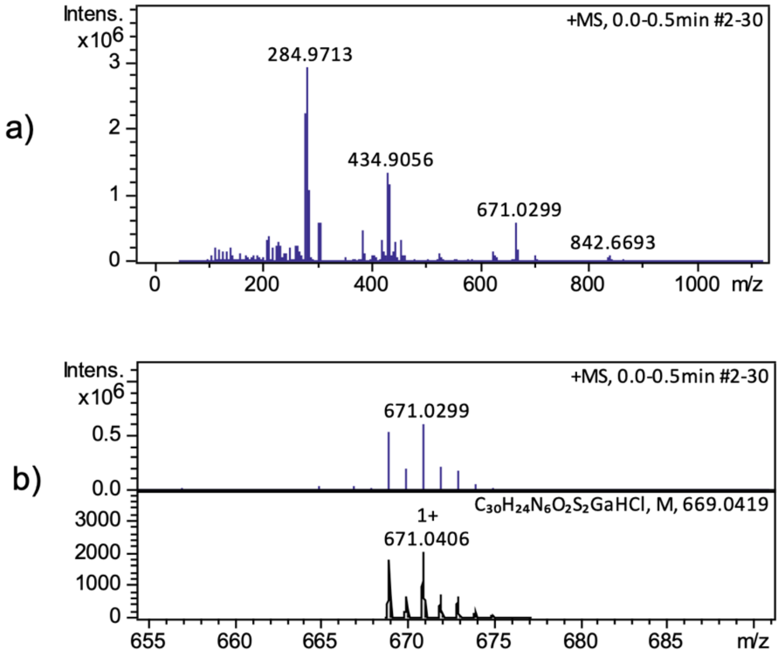

Figure S100. ESI Mass spectrometry results: Positive mode a) full spectra, b) $[M+H+Cl]^+$

8. Additional mass spectrometry data for key compounds and intermediates

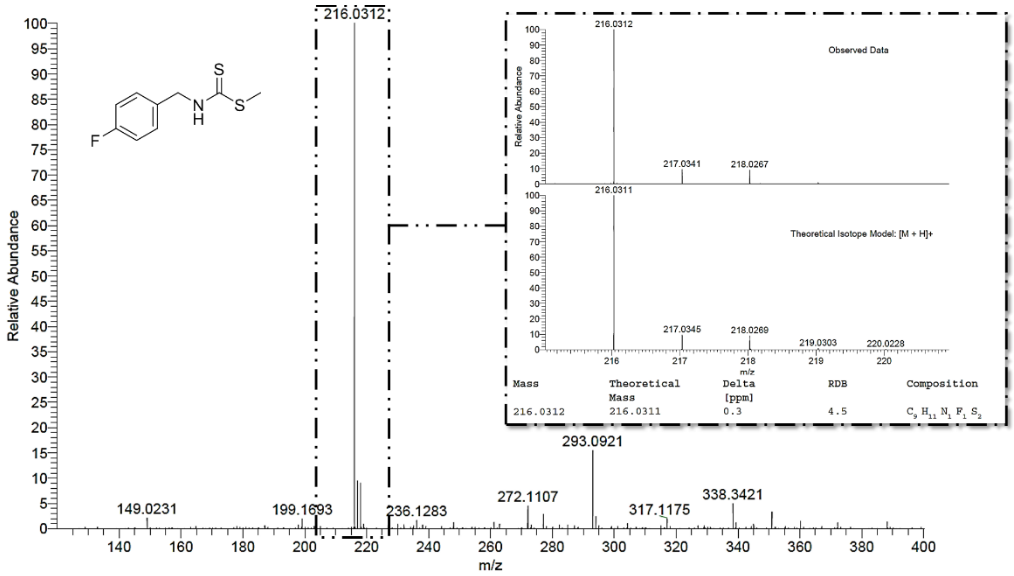

Figure S101. ESI Mass spectrometry results for fluorinated precursor

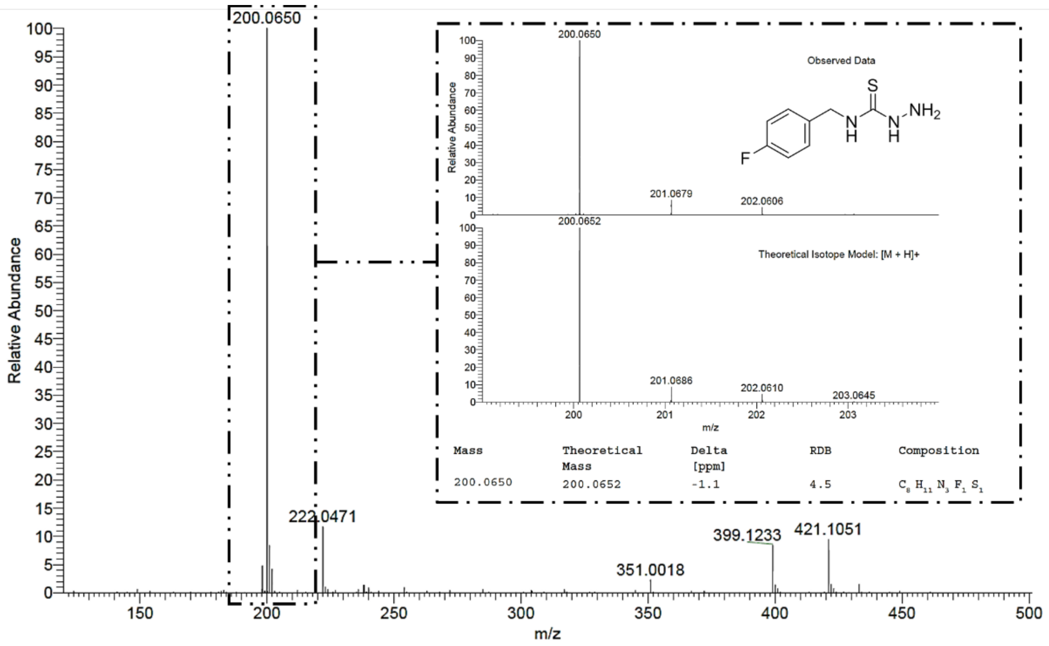

Figure S102 ESI Mass spectrometry results for fluorinated thiosemicarbazone precursor

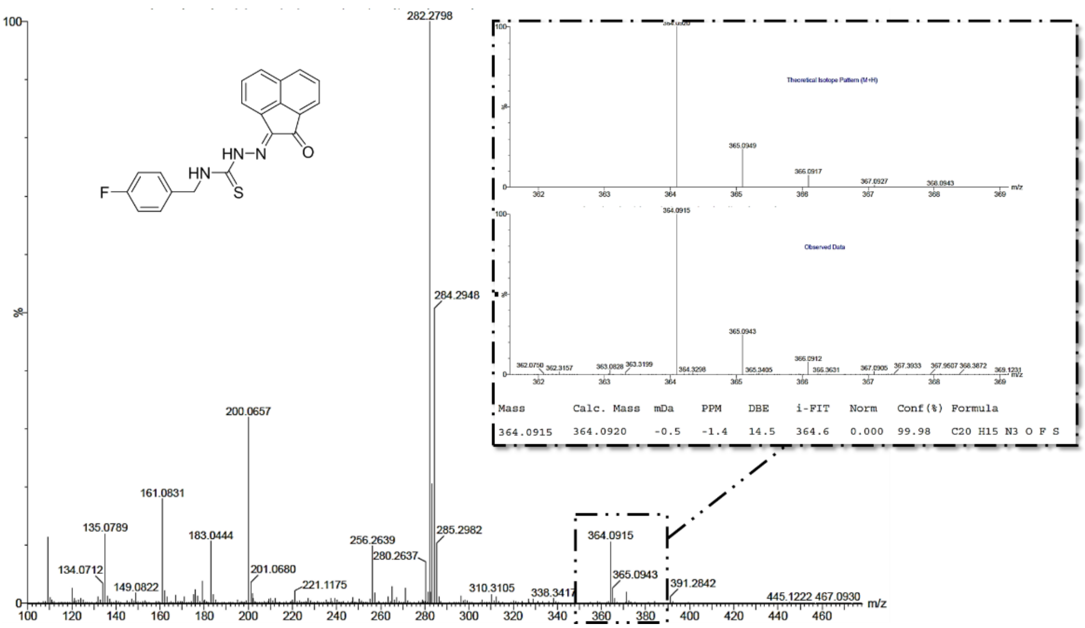

Figure S103. ESI-MS analysis: mass spectrometry of compound **4a**

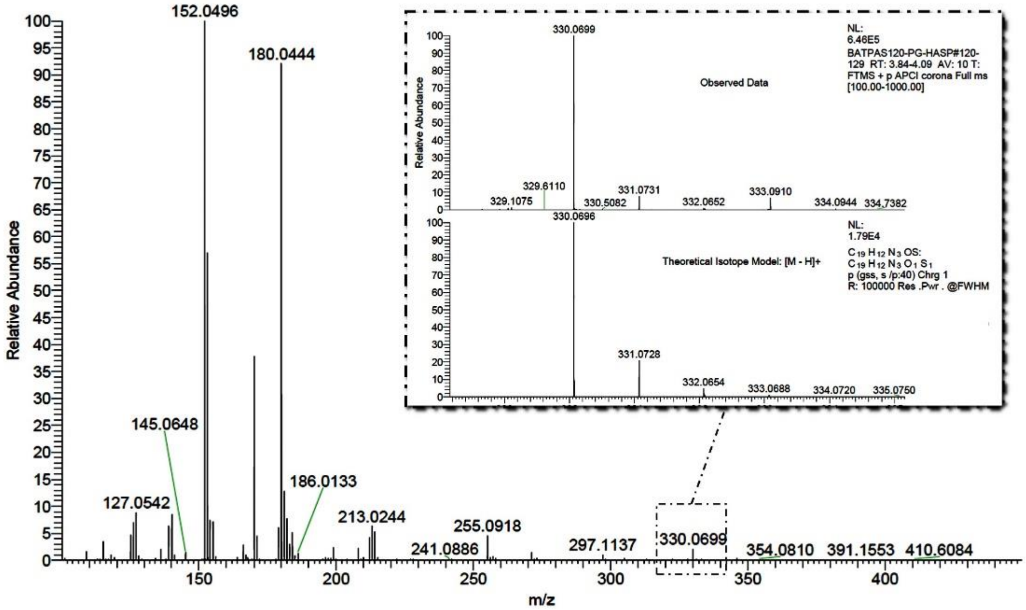

Figure S104. ESI-MS analysis: mass spectrometry of 4-phenyl-3-thiosemicarbazone acenaphthenequinone (PhTSCA, **4h**).

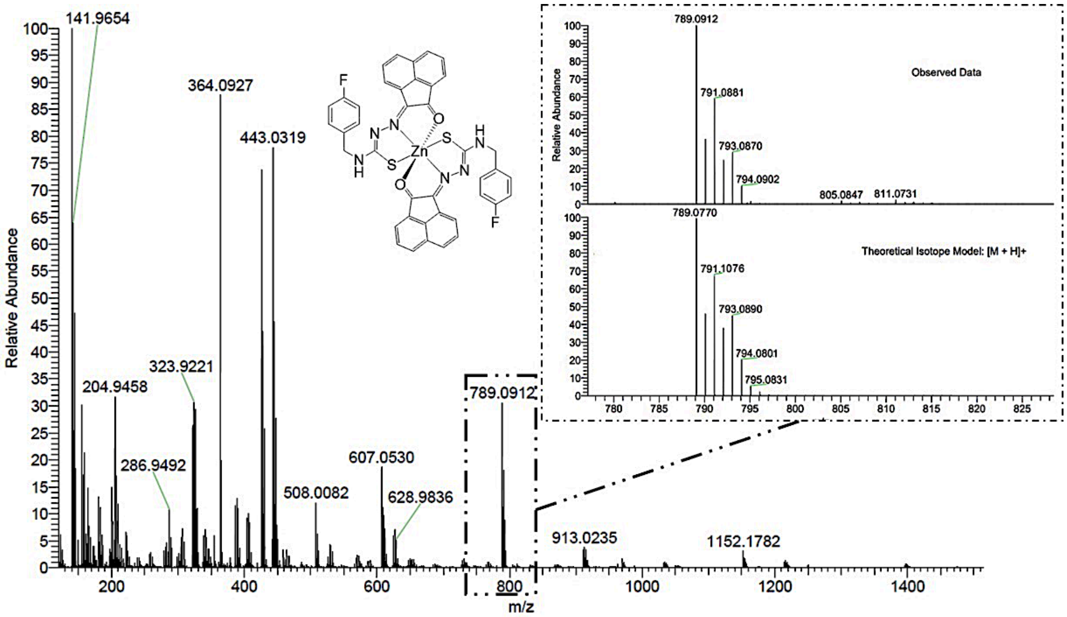

Figure S105. ESI-MS analysis mass spectrometry for the Zinc(II) [mono (F-benzyl thiosemicarbazonate-acenaphthenequinone]$_2$ (**4a-Zn**).

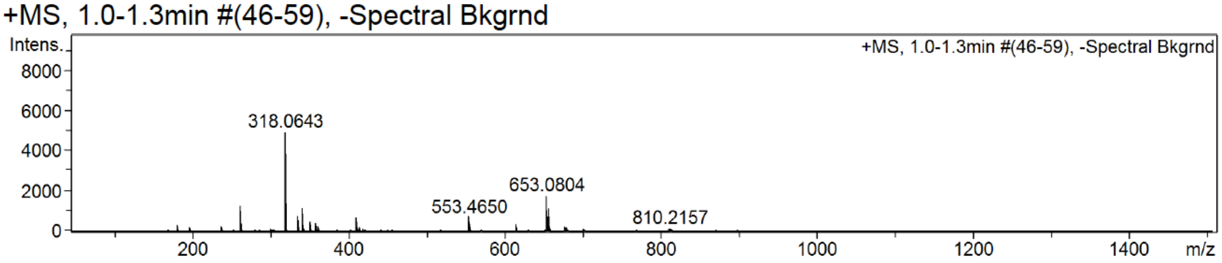

#	m/z	I	I %	Area	S/N
1	261.0100	1243	25.5	57	2605.0
2	318.0643	4884	100.0	288	7670.5
3	319.0705	1008	20.6	50	1584.1
4	334.0512	780	16.0	63	1230.9
5	340.0480	1170	24.0	83	1850.5
6	409.1608	669	13.7	51	1310.5
7	553.4650	694	14.2	51	1265.4
8	653.0804	1768	36.2	190	1385.6
9	654.0836	734	15.0	70	571.8
10	655.0823	1128	23.1	159	872.9

Figure S106. ESI-MS analysis mass spectrometry of **4g-Zn** complex

9. Infrared spectroscopy for representative compounds

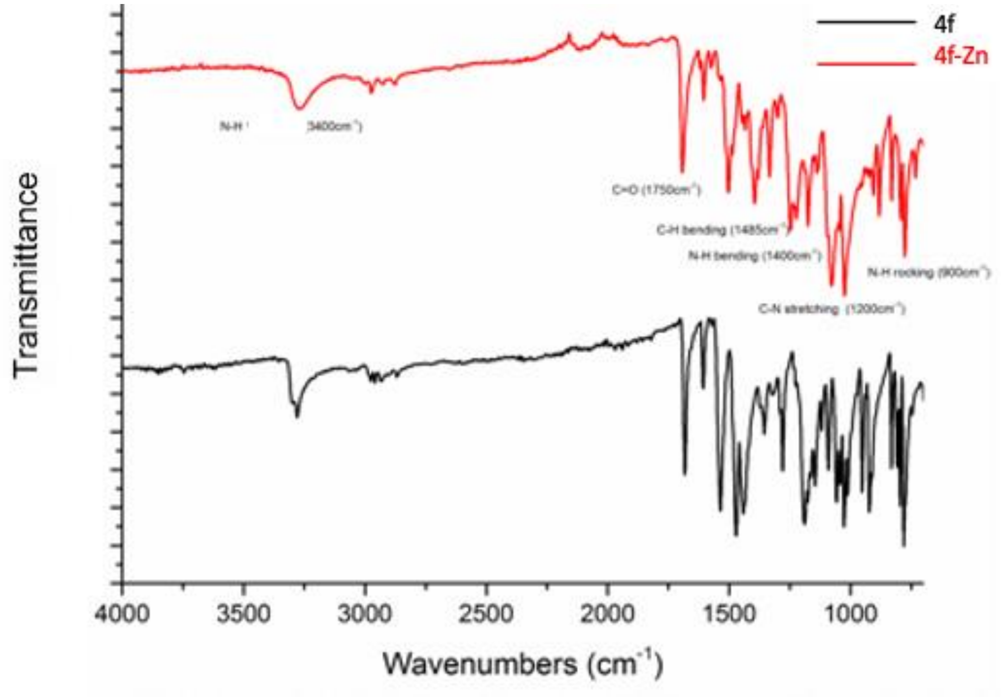

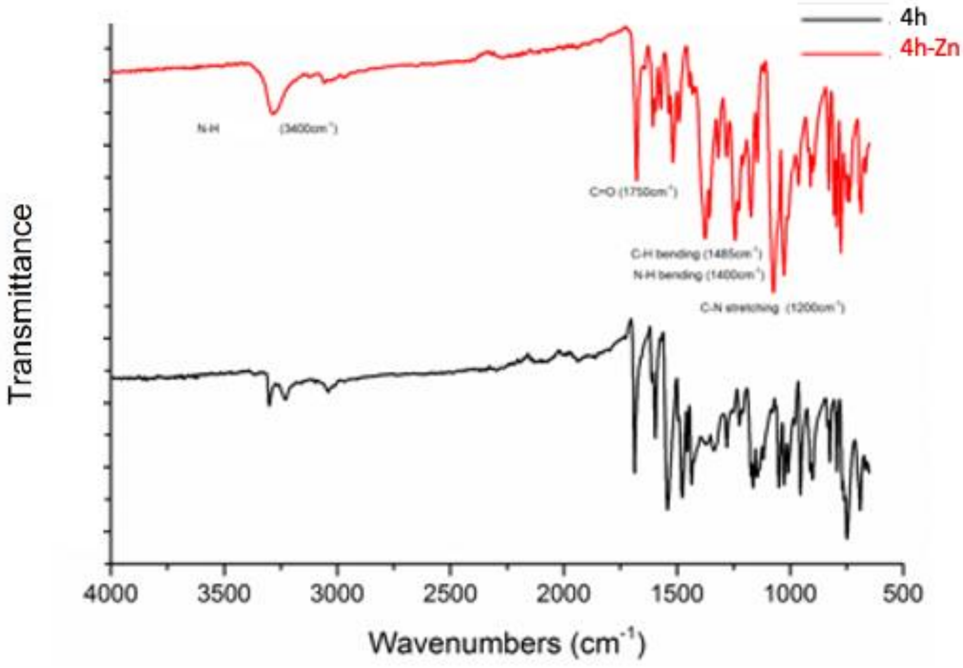

Figure S107. IR spectra for compounds **4f** and **4f-Zn** (overlay, top) as well as for **4h** and **4h-Zn** (overlay, bottom).

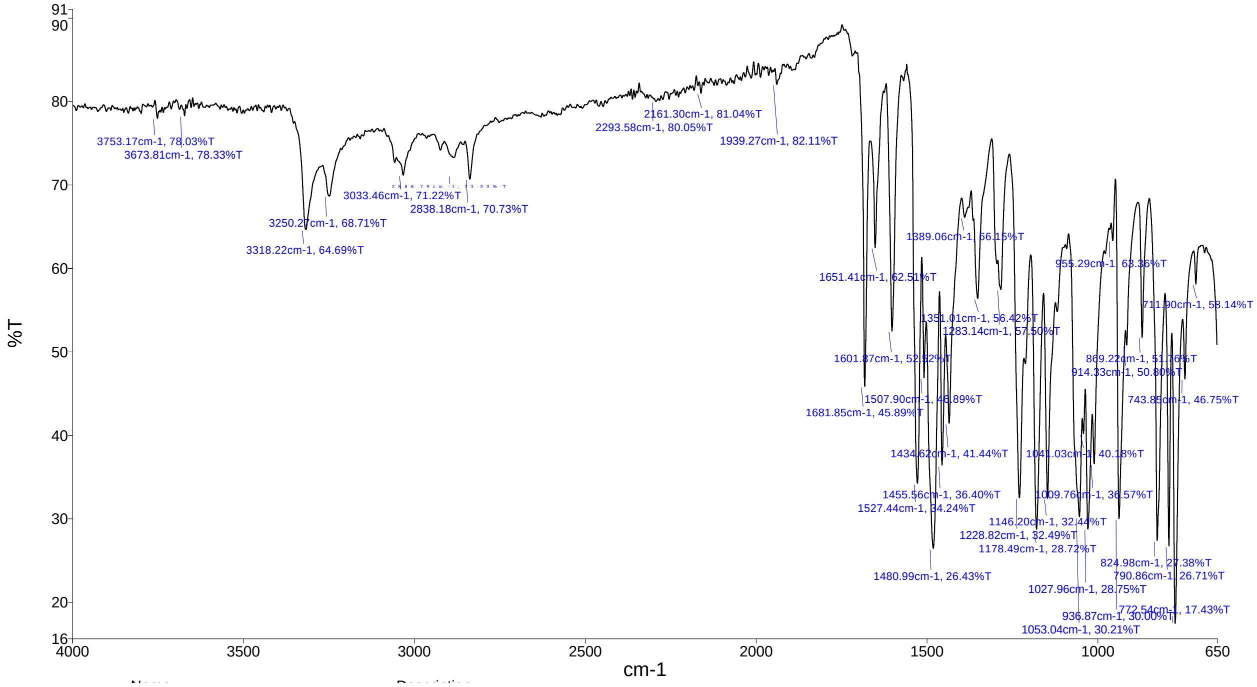

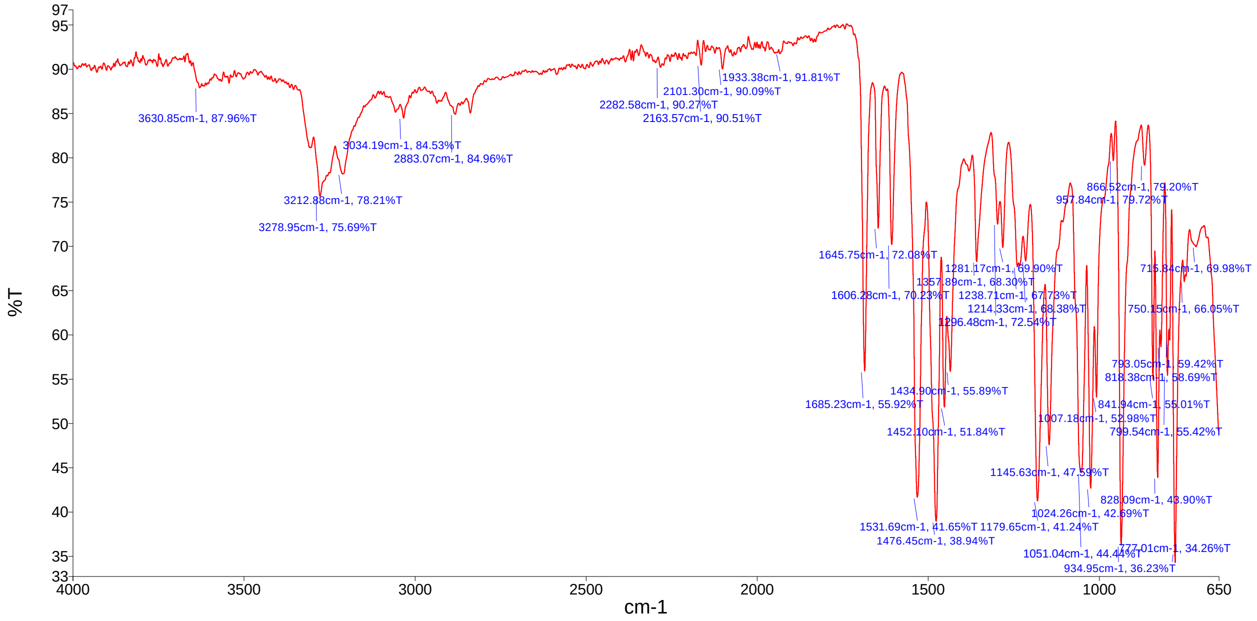

Figure S108. IR spectra for compounds **4d** (top) and **4e** (bottom).

10. Selected X-Ray Crystallographic Data

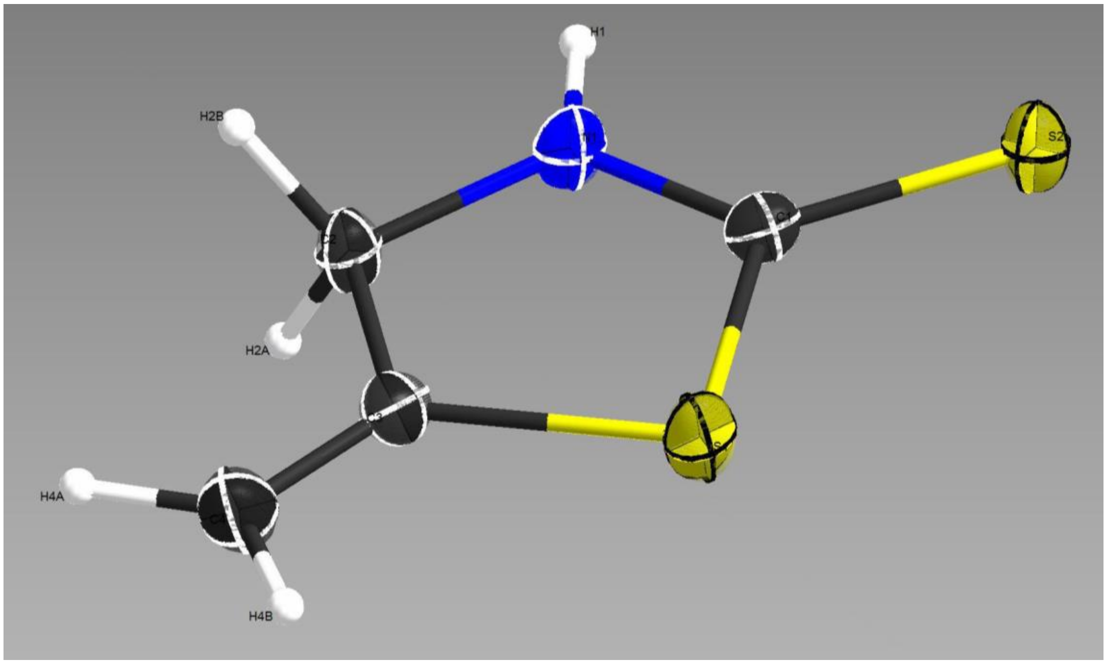

Figure S109. Single crystals X-ray diffraction structure of precursor 5-methylene-2-(l1-sulfanyl)thiazolidine precursor/intermediate. Thermal ellipsoids at 50% probability

Table S1: Crystal data and structure refinement for 5-methylene-2-(l1-sulfanyl)thiazolidine

Identification code	E16SIP12	
Empirical formula	C4 H5 N S2	
Formula weight	131.21	
Temperature	150.0(4) K	
Wavelength	0.71073 Å	
Crystal system	Monoclinic	
Space group	$P2_1/n$	
Unit cell dimensions	a = 6.1770(3) Å	α= 90°.
	b = 6.0691(3) Å	β= 91.868(4)°.
	c = 15.2900(8) Å	γ = 90°.
Volume	572.90(5) $Å^3$	
Z	4	
Density (calculated)	1.521 Mg/m^3	
Absorption coefficient	0.791 mm^{-1}	
F(000)	272	
Crystal size	0.400 x 0.350 x 0.180 mm^3	
Theta range for data collection	3.519 to 30.065°.	
Index ranges	-8<=h<=8, -8<=k<=8, -21<=l<=20	
Reflections collected	4880	
Independent reflections	1525 [R(int) = 0.0302]	
Completeness to theta = 25.242°	99.9 %	
Absorption correction	Semi-empirical from equivalents	
Max. And min. Transmission	1.00000 and 0.96866	
Refinement method	Full-matrix least-squares on F^2	
Data / restraints / parameters	1525 / 0 / 68	
Goodness-of-fit on f^2	1.059	
Final r indices [i>2sigma(i)]	R1 = 0.0307, wR2 = 0.0692	
R indices (all data)	R1 = 0.0381, wR2 = 0.0726	
Extinction coefficient	n/a	
Largest diff. Peak and hole	0.317 and -0.245 e.$Å^{-3}$	

Table S1: Selected Bond lengths [Å] and angles [°] for 5-methylene-2-(l1-sulfanyl)thiazolidine

S(2)-C(1)	1.6686(16)	C(2)-N(1)-H(1)	121.3(13)
S(1)-C(1)	1.7499(16)	N(1)-C(1)-S(2)	127.39(13)
S(1)-C(3)	1.7735(16)	N(1)-C(1)-S(1)	110.95(12)
N(1)-C(1)	1.3252(19)	S(2)-C(1)-S(1)	121.65(9)
N(1)-C(2)	1.452(2)	N(1)-C(2)-C(3)	106.98(12)
N(1)-H(1)	0.84(2)	N(1)-C(2)-H(2A)	110.3
C(2)-C(3)	1.511(2)	C(3)-C(2)-H(2A)	110.3
C(2)-H(2A)	0.9900	N(1)-C(2)-H(2B)	110.3
C(2)-H(2B)	0.9900	C(3)-C(2)-H(2B)	110.3
C(3)-C(4)	1.315(2)	H(2A)-C(2)-H(2B)	108.6
C(4)-H(4A)	0.9500	C(4)-C(3)-C(2)	126.33(14)
C(4)-H(4B)	0.9500	C(4)-C(3)-S(1)	124.53(13)
C(1)-S(1)-C(3)	93.11(7)	C(2)-C(3)-S(1)	109.14(11)
C(1)-N(1)-C(2)	118.94(14)	C(3)-C(4)-H(4AorB)	120.0
C(1)-N(1)-H(1)	119.7(13)	H(4A)-C(4)-H(4B)	120.0

11. **Selected X-ray** diffraction data for **2a**, **3a**, **4a**, **4b and 4c**

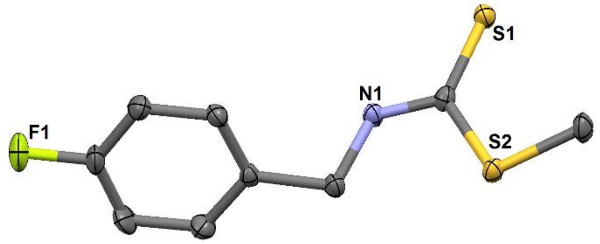

Figure S110. Structural view of methyl (4-fluorobenzyl)carbamodithioate (**2a**) showing 50% thermal ellipsoids (H atoms omitted for clarity).

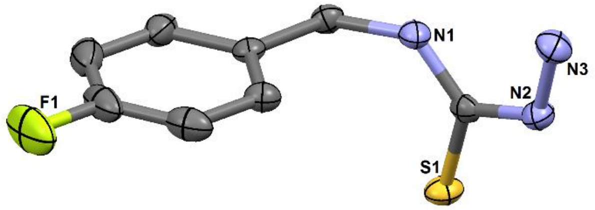

Figure S111. Structural view of N-(4-fluorobenzyl)hydrazinecarbothioamide (**3a**) showing 50% thermal ellipsoids (H atoms omitted for clarity).

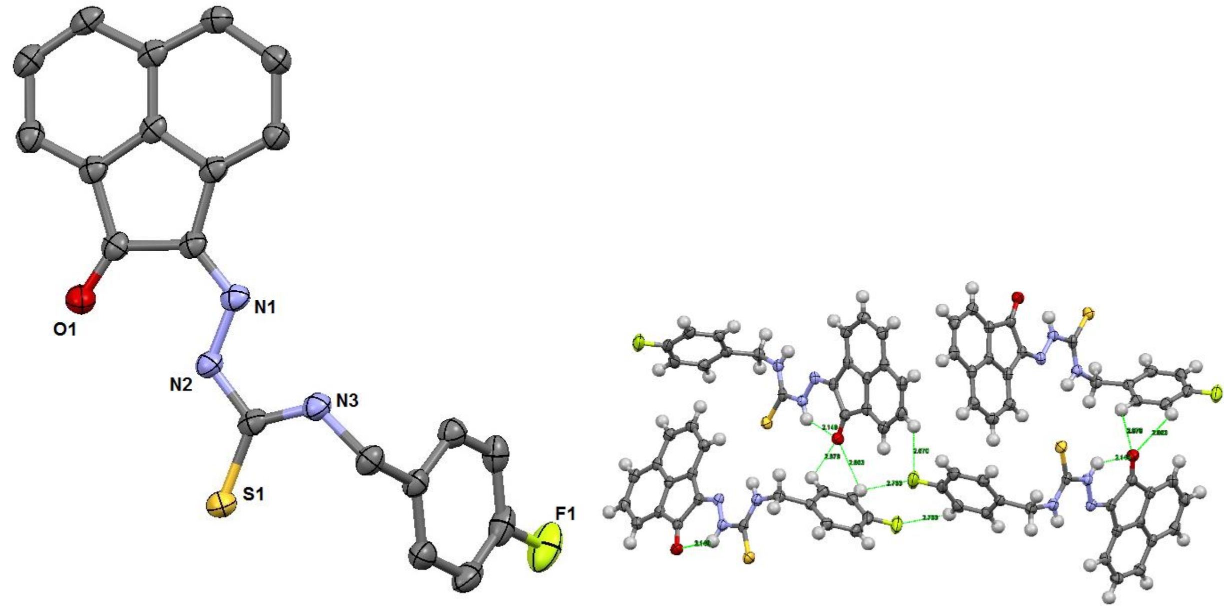

Figure S112. Structural view of the thiosemicarbazone ligand **4a** showing 50% thermal ellipsoids (H atoms omitted for clarity).

Table S3. Crystal and structure refinement data for **2a**, **3a** and **4a**

	**2a**	**3a**	**4a**
Empirical formula	$C_9 H_{10} F_1 N_1 S_2$	$C_8 H_{10} F_1 N_3 S_1$	$C_{20} H_{14} F_1 N_3 O_1 S_1$
Formula weight	215.30	199.25	363.40
Crystal system	Triclinic	Monoclinic	Orthorhombic
Space group	P-1	$P2_1/c$	$P2_12_12_1$
a / Å	5.8689(5)	4.74990(10)	5.11830(10)
a / Å	8.0985(7)	5.6068(2)	12.2027(2)
c/ Å	11.1040(9)	34.4659(13)	26.4044(3)
α/ °	70.418(8)	90	90
β/ °	84.161(7)	93.902(3)	90
γ/ °	87.618(7)	90	90
Volume / $Å^3$	494.65(8)	915.76(5)	1649.14(5)
Z	2	4	4
Density (Calculated)/ Mg/m^3	1.446	1.445	1.464
Absorption coefficient/ mm^{-1}	0.503	2.932	1.964
F(000)	224	416	752
GOF	1.065	1.166	1.041
Reflections collected	4239	9108	19380
Independent reflections	2496 [R(int) = 0.0247]	1809 [R(int) = 0.0196]	3297 [R(int) = 0.0543]
Final R indices [I>2sigma(I)]	R1 = 0.0475, wR2 = 0.0883	R1 = 0.0287, wR2 = 0.0887	R1 = 0.0337, wR2 = 0.0867
Largest diff. peak and hole/ $e.Å^{-3}$	0.423 and -0.294	0.406 and -0.359	0.272 and -0.186

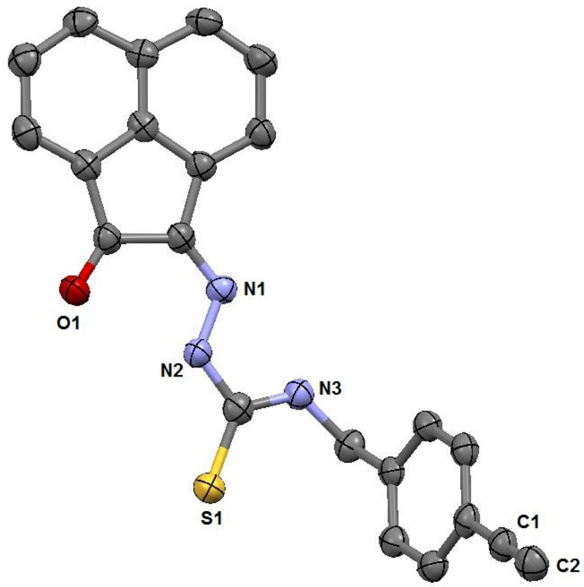

Figure S113. Structural view of the thiosemicarbazone ligand **4b** showing 50% thermal ellipsoids (H atoms omitted for clarity).

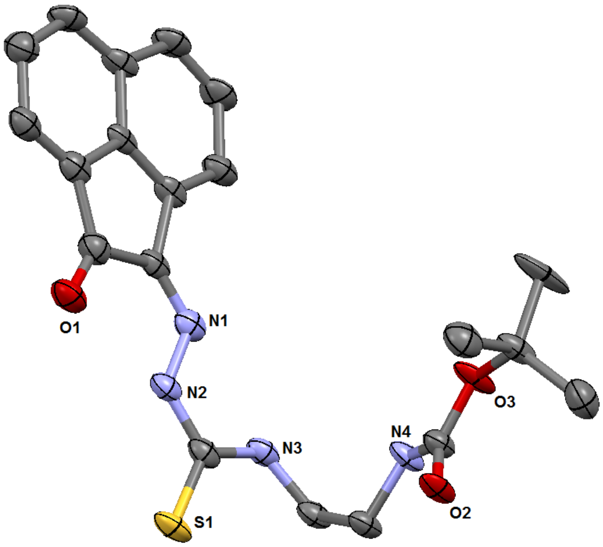

Figure S114. Structural view of the thiosemicarbazone ligand **4c** showing 50% thermal ellipsoids (H atoms omitted for clarity).

Table S4. Crystal and structure refinement data for **4b** and **4c**

	**4b**	**4c**
Empirical formula	C_{22} H_{15} N_3 O_1 S_1	C_{20} H_{21} N_4 O_3 S_1
Formula weight	369.43	397.47
Crystal system	Orthorhombic	Monoclinic
Space group	$P2_12_12_1$	$P2_1/c$
a / Å	5.3336(3)	5.2829(7)
a / Å	12.1295(5)	35.811(9)
c/ Å	27.1119(16)	10.5644(18)
α/ °	90	90
β/ °	90	101.588(16)
γ/ °	90	90
Volume / $Å^3$	1753.97(16)	1957.9(7)
Z	4	4
Density (Calculated)/ Mg/m^3	1.399	1.348
Absorption coefficient/ mm^{-1}	1.774	1.713
F(000)	768	836
GOF	1.070	1.062
Reflections collected	10236	11002
Independent reflections	3461 [R(int) = 0.0590]	3579 [R(int) = 0.0853]
Final R indices [I>2sigma(I)]	R1 = 0.0536, wR2 = 0.1228	R1 = 0.1339, wR2 = 0.2917
Largest diff. peak and hole/ e.$Å^{-3}$	0.258 and -0.168	0.921 and -0.521

12. CCDC Deposition Information

The Xray structures of Compounds listed below are all available free of charge from CCDC (https://www.ccdc.cam.ac.uk/), with the CCDC Deposition Numbers given below.

Table S5.

CCDC Number:	Compound		CCDC CIF Label
2130526	C4 H5 N1 S2	**Precursor 1**	data_e16sip12
2130525	C9 H10 F1 N1 S2	**2a**	data_e16sip13
2130524/ 2149602	C8 H10 F1 N3 S1	**3a (2 polymorphs)**	data_s16sip5a and data_e16sip14
2130523	C20 H14 F1 N3 O1 S1	**4a**	data_s16sip7
2130521	C20 H22 N4 O3 S1	**4c**	data_s17sip1
2130516	C22 H15 N3 O1 S1	**4b**	data_s17sip2
2130510	C19 H13 N3 O1 S1,C2 H6 O1 S1	**4h-DMSO complex**	data_arc1523
2130508	C19 H13 N3 O1 S1	**4h**	data_ox40
2130507	C16 H13 N3 O1 S1	**4g**	data_s17sip3
2131107	C15 H13 N3 O1 S1	**4f**	data_napht
2130502	C30 H24 N6 O2 S2 Zn1,C2 H6 O1 S1	**4f-Zn Oh (DMSO)**	data_zn_mono_et
2130501	C30 H24 N6 O2 S2 Zn1	**4f-Zn Td**	data_ox082

13. Estimation of logP

LogP for the free ligands investigated were evaluated theoretically using SwissADME.[1-3] This approach generates five different calculated logP values, based on different methodologies the consensus logP gives the average of these values.

Table S6

Compound	Consensus LogP [3]
**4a**	3.98
**4b**	4.08
**4c**	2.94
**4c***	1.85
**4d**	4.07
**4e**	3.38
**4f**	2.78
**4g**	3.01
**4h**	3.69

14. Gas-phase DFT Calculations

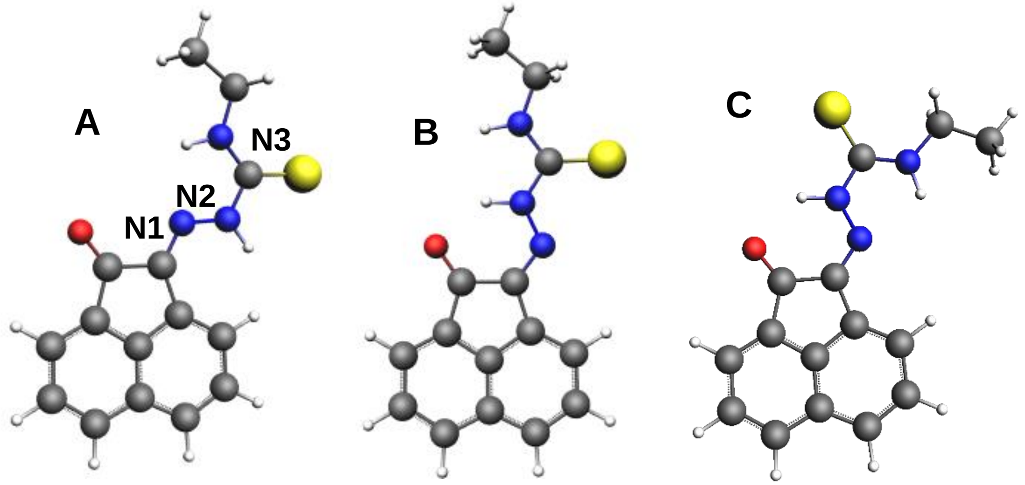

Figure S115. Optimised geometries for the isomers of Compound **4f**

Table S7. Optimised energies for Compound **4f**, Isomers **A, B** and **C**

Geometry	Protocol	Bond Energy [eV]	ZPE [eV]
**4f-I (A)**	BLYP/TZ2P	-205.8204 eV<br>-4746.33 kcal/mol	6.7093 eV<br>154.72 kcal/mol
**4f-II (B)**	BLYP/TZ2P	-205.6919 eV<br>-4743.37 kcal/mol	6.6973 eV<br>154.44 kcal/mol
**4f-III (C)**	BLYP/TZ2P	-206.0110 eV<br>-4750.73 kcal/mol	6.7157 eV<br>154.87 kcal/mol

Table S8: Geometric Parameters:

Bond Distance [pm] /Angle [°]	A	B	C
C-S [pm]	167.9	166.6	167.7
C-O [pm]	122.3	124.3	123.6
C-N1 [pm]	130.3	131.0	131.0
N1-N2 [pm]	134.0	133.7	134.3
C-N1-N2 [°]	121.3	117.2	118.7
N2-C-N3 [°]	114.4	110.7	114.6
N2-C-S [°]	118.4	124.7	119.0
C-N3-C [°]	124.4	124.1	124.3

DFT Geometry optimizations of 4f-Zn and 4f-Ga complexes

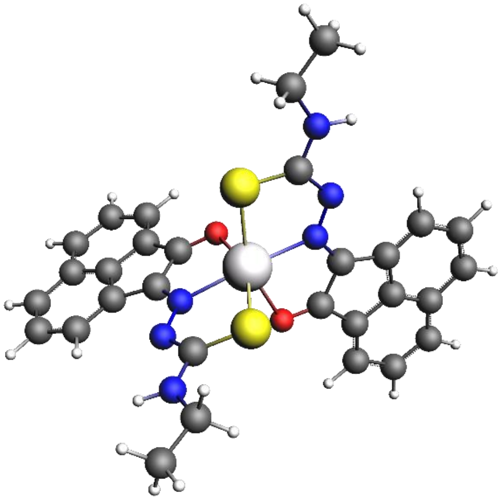

Zn-1

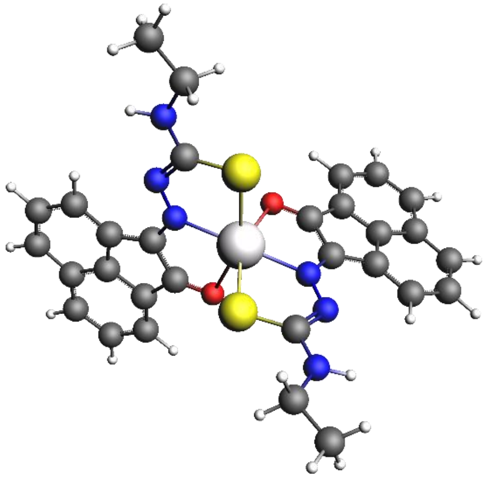

Zn-2

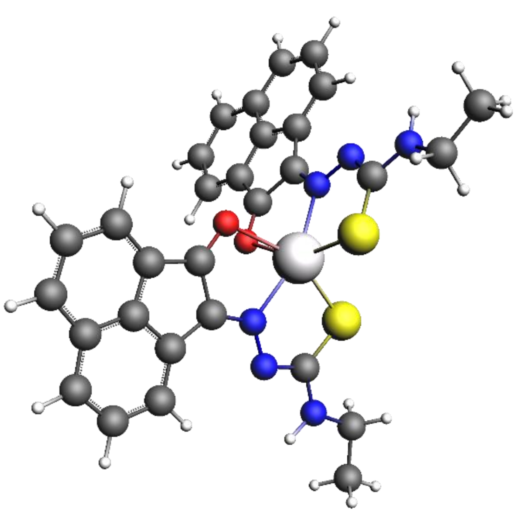

Alternative view Zn-1

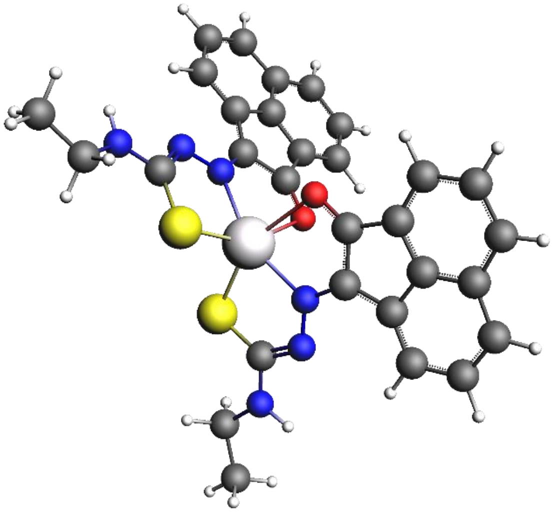

Alternative view Zn-2

Figure S116. Optimised geometries for the isomers of the Zn-complex of ligand **4f**

Table S9. Optimised energies for the two isomers of the Zn(II) complex of ligand **4f.**

**Geometry**	**Protocol**	**Bond Energy [eV]**	**ZPE [eV]**
**Zn – Isomer 1**	BLYP/TZ2P	-406.7023 eV<br>-9378.78 kcal/mol	12.8466 eV<br>296.25 kcal/mol
**Zn – Isomer 2**	BLYP/TZ2P	-406.7019 eV<br>-9378.77 kcal/mol	12.8466 eV<br>296.25 kcal/mol

Table S10. Selected molecular parameters for the Zn(II) complexes of compound **4f.**

**Distance [pm] Angle [°]**	**Zn-1**	**Zn-2**
C-S [pm]	172.3 / 172.4	172.4
S-M [pm]	246.8 / 247.1	246.4 / 246.7
N-M [pm]	215.9 / 216.0	215.6 / 215.7
O-M [pm]	243.9 / 246.7	247.3 / 247.6
S-M-S [°]	104.1	104.3
N-M-N [°]	158.9	157.0
O-M-O [°]	82.0	82.0

DFT investigations into the geometries of the Ga(III) complex isomers of ligand 4f

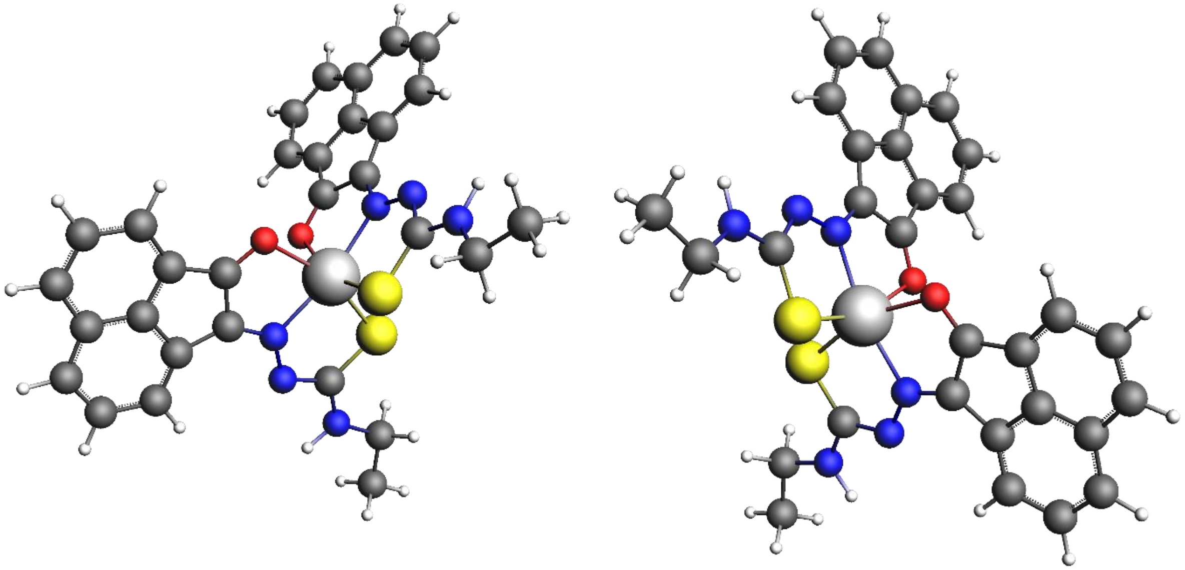

Ga-1 **Ga-2**

Figure S117. Optimised geometries of the two isomers of the monocationic Ga(III) complex ion of ligand **4f.** The Cl^- counterion was not included in the model.

Table S11: Optimised energies for the two isomers of the Ga(III) complex of ligand **4f**

Geometry	Protocol	Bond Energy [eV]	ZPE [eV]
**Ga – Isomer 1**	BLYP/TZ2P	-403.7878 eV<br>-9311.57 kcal/mol	12.9261 eV<br>298.08 kcal/mol
**Ga – Isomer 2**	BLYP/TZ2P	-403.7867 eV<br>-9311.54 kcal/mol	12.9266 eV<br>298.08 kcal/mol

Table S12. Selected molecular parameters for the Ga(III) complexes of compound **4f.**

**Distance [pm]**<br>**Angle [°]**	**Ga-1**	**Ga-2**
C-S [pm]	173.9	173.9
S-M [pm]	243.2 / 243.3	242.9
N-M [pm]	212.4	212.3 / 212.4
O-M [pm]	216.6 / 217.1	216.9 / 217.3
S-M-S [°]	99.8	99.6
N-M-N [°]	168.4	167.5
O-M-O [°]	83.6	83.6

References:

1. Daina, A.; Michielin, O.; Zoete, V., iLOGP: A Simple, Robust, and Efficient Description of n-Octanol/Water Partition Coefficient for Drug Design Using the GB/SA Approach. *J. Chem. Inf. Model.* **2014,** *54* (12), 3284-3301.
2. Daina, A.; Michielin, O.; Zoete, V., SwissADME: a free web tool to evaluate pharmacokinetics, drug-likeness and medicinal chemistry friendliness of small molecules. *Sci. Rep.* **2017,** *7* (1), 42717.
3. http://www.swissadme.ch/ (accessed February 2022)
